# Bis-silylation of internal alkynes enabled by Ni(0) catalysis

**DOI:** 10.1038/s41467-020-20392-w

**Published:** 2021-01-04

**Authors:** Yun Zhang, Xi-Chao Wang, Cheng-Wei Ju, Dongbing Zhao

**Affiliations:** State Key Laboratory and Institute of Elemento-Organic Chemistry, College of Chemistry, Nankai University, 300071 Tianjin, China

**Keywords:** Catalytic mechanisms, Heterogeneous catalysis, Synthetic chemistry methodology, Synthetic chemistry methodology

## Abstract

1,2-Bis-silyl alkenes have exciting synthetic potential for programmable sequential synthesis via manipulation of the two vicinal silyl groups. Transition metal-catalyzed bis-silylation of alkynes with disilanes is the most straightforward strategy to access such useful building blocks. However, this process has some limitations: (1) symmetric disilanes are frequently employed in most of the reactions to assemble two identical silyl groups, which makes chemoselective differentiation for stepwise downstream transformations difficult; (2) the main catalysts are low-valent platinum group transition metal complexes, which are expensive; and (3) internal alkynes remain challenging substrates with low inherent reactivity. Thus, the development of abundant metal-catalyzed bis-silylation of internal alkynes with unsymmetrical disilanes is of significance. Herein, we solve most of the aforementioned limitations in bis-silylation of unsaturated bonds by developing a strongly coordinating disilane reagent and a Ni(0) catalytic system. Importantly, we sufficiently realize the stepwise recognition of the two silyl groups, making this synthetic protocol of wide potential utility.

## Abstract (web summary)

Bis-silylated alkenes offer the advantage of two functional handles with distinguished reactivity for downstream functionalization. Here, the authors report a nickel-catalyzed bis-silylation of internal alkynes to versatile silylated alkene intermediates which can be chemoselectively manipulated.

## Introduction

**Fig. 1:**
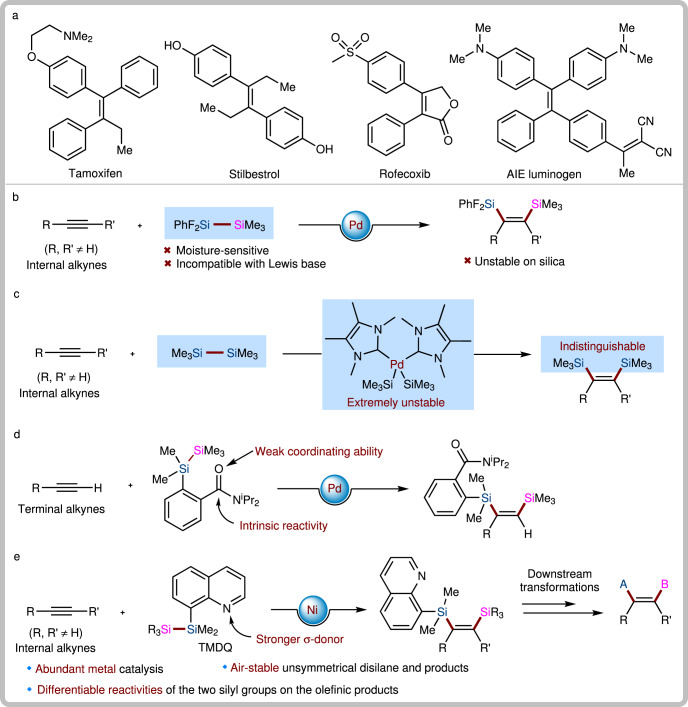
Research background and our study on Ni(0)-catalyzed bis-silylation of internal alkynes with unsymmetric disilanes. **a** Some multi-substituted alkenes as drugs and luminogens. **b** The seminal report on bis-silylation of internal alkynes with acyclic disilanes (Ozawa’s work). **c** The breakthrough on bis-silylation of internal alkynes with stable disilanes (Spencer’s work). **d** Development of directing group-activated air-stable disilane for bis-silylation. **e** Bis-silylation of internal alkynes with air-stable disilane enabled by Ni(0) catalysis (this work).

The numerous applications of organosilicon compounds, ranging from organic synthetic chemistry and materials science to medicinal chemistry, indicate that the development of reliable and inexpensive methods for rapid preparation of silylated synthons is of great demand^1–5^. Within this context, transition metal-catalyzed bis-silylation of alkynes with disilanes, especially acyclic disilanes, has attracted substantial interest, as reflected by the ample body of literature^6–12^. The olefinic products juxtaposing two vicinal silicon atoms have been recognized as versatile building blocks leading to high-value scaffolds, such as densely substituted alkenes (Fig. 1a)^13–17^. Despite significant advances in this reaction, there are still some limitations: (1) symmetric disilanes are frequently employed in most reactions to assemble two identical silyl groups on olefinic products, but are difficult to differentiate chemoselectively for stepwise downstream transformations^18–25^; (2) the main catalysts are low-valent platinum group transition metal complexes, which are expensive; and (3) internal alkynes remain challenging substrates with low inherent reactivity in most catalytic systems.

To date, only two examples of *cis*-bis-silylation of internal alkynes with acyclic disilanes have been reported. Ozawa and coworkers reported seminal work on the bis-silylation of internal alkynes by employing Me_3_SiSiF_2_Ph as the disilane reagent with a Pd catalyst (Fig. 1b)^26^. However, the high toxicity, moisture sensitivity, and intolerance to various Lewis bases of Me_3_SiSiF_2_Ph, as well as the instability of the bis-silylated products (unstable on silica) severely limit the practicality of this method. In 2015, Spencer and coworkers made an important breakthrough in the intermolecular bis-silylation of internal alkynes with air-stable hexamethyldisilane by using the [(NHC)_2_Pd(SiMe_3_)_2_] (NHC = N-heterocyclic carbene) complex as the precatalyst (Fig. 1c)^27^; however, their system has two drawbacks: (1) such a bis(silyl)Pd precatalyst is relatively difficult to access and must be handled in an ampoule due to its extreme instability; (2) the two identical TMS groups on the product are inert and indistinguishable for downstream transformations. Clearly, the development of abundant metal-catalyzed bis-silylation reactions of internal alkynes with unsymmetrical disilanes is of significance and would increase the utility of this process.

Recently, Song and coworkers developed an air-stable unsymmetrical disilane by attaching a coordinating group to one of the two silicon atoms, which was successfully applied to the Pd-catalyzed bis-silylation of terminal alkynes^28^. Such an unsymmetrical disilane is air-stable and offers exciting possibilities to differentiate the reactivities of the two silyl groups introduced. However, the weak coordinating ability and the intrinsic reactivity of the amide group on Song’s reagent have decreased their application scope. We envisioned that practical bis-silylation of internal alkynes with air-stable unsymmetrical disilane could be achieved by tuning the variety of directing groups on the silicon atoms and choosing appropriate transition metal catalytic systems, yielding tetrasubstituted vinylsilanes. In fact, despite the existence of numerous methods to produce vinylsilanes^29–31^, the synthesis of tetrasubstituted vinylsilanes remains challenging^32–35^. Inspired by the strong coordination ability of N-heterocycles that enables the formation of thermodynamically stable metallacycles^36^, we wondered whether the introduction of a quinolyl group on a disilane would change the coordinating ability of the metal center and result in higher reactivity.

Herein, we achieve the bis-silylation of internal alkynes with an air-stable unsymmetrical disilane by developing a strongly coordinating disilane reagent 8-(2-substituted-1,1,2,2-tetramethyldisilanyl)quinoline (TMDQ) and a Ni(0)-catalytic system (Fig. 1c). Notably, Ni(0) catalysis has never been utilized in the bis-silylation of π-bonds with acyclic disilanes before^37,38^. Moreover, both the disilane reagent and the products in our study are stable on silica, and the reactivities of the two nonequivalent silyl groups on the olefinic products can be distinguished sufficiently, making this synthetic protocol of great potential utility.

## Results

### Preparation of strongly coordinating disilane TMDQ reagents

**Fig. 2:**
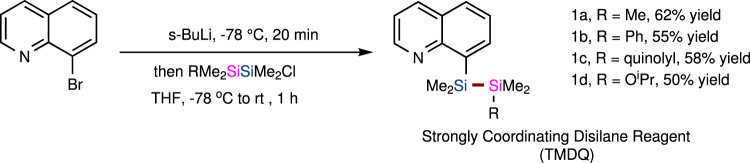
The synthetic route for producing the strongly coordinating disilane TMDQ reagent. The detailed procedures for preparation of these disilane TMDQ reagents have been presented in Supplementary Information (SI).

TMDQ reagents were easily synthesized in one step, as shown in Fig. 2. With commercially available, inexpensive 8-bromoquinoline as the starting material, the lithium halogen exchange reaction with *sec*-butyllithium followed by the treatment with Me_2_RSiSiMe_2_Cl yielded the strongly coordinating disilane TMDQ reagents **1a–d**. Notably, our TMDQ reagents are air-stable, colorless liquids that can be synthesized practically on the gram scale and can be easily purified by column chromatography.

### Condition screening

With the unsymmetrical disilane TMDQ reagent in hand, we conducted the reaction of disilane **1a** and diphenyl acetylene **2a** under the Pd-catalyzed conditions reported by Song and coworkers for the bis-silylation of terminal alkynes^28^, but these reagents were found to be completely unreactive under these conditions (Table 1, entry 1). After numerous attempts to optimize the reaction conditions (for more details, see Supplementary Table 1 in Supplementary Information), we found that the bis-silylation of unsymmetrical disilane **1a** and diphenyl acetylene **2a** occurred smoothly under Ni(0)-catalytic conditions, and the optimized conditions were 10 mol % Ni(COD)_2_, SIPr (12 mol%), and toluene as the solvent to produce *cis*-bis-silylated olefinic product **3aa** in 98% yield at 100 °C for 24 h (Table 1, entry 2). To confirm the structure of **3aa**, structural analysis of a single crystal was carried out by X-ray diffraction. Control experiments were subsequently carried out to investigate the effect of each component. The use of SIPr generated in situ from SIPr·HCl incorporation of *t-*BuOK led to inferior yield (entry 3). Switching the SIPr·HCl to ICy·HBF_4_ dramatically decreased the yield of this reaction (entry 4). Reducing the amount of the Ni catalyst to 5 mol% resulted in a slightly decreased yield (entry 5). Interestingly, the bis-silylation reaction also proceeded smoothly, with 92% yield, when Ni(COD)_2_/PPh_3_ was used as the catalytic system (entry 6). We also investigated the effects of disilane reagents on this bis-silylation reaction under the optimized conditions. We found that no reaction occurred if unsymmetric disilane **1a** was replaced with Me_3_SiSiMe_3_ (**1e**) under the optimized conditions (entry 7). No desired bis-silylated product was detected if the 8-quinolinyl group was replaced with a 1-naphthalenyl group in unsymmetric disilane **1a** (entry 8). Notably, the reaction occurred, albeit at a low yield, when Song’s disilane reagent **1g** was used during the reaction (entry 9). These results indicate that the introduction of the directing group on the unsymmetric disilane was vital for ensuring this bis-silylation reaction.

**Table 1:**
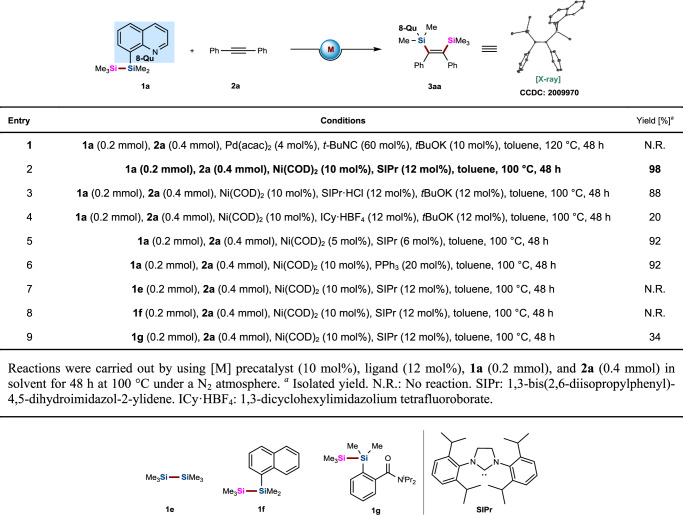
Control experiments for the screening of reaction condition.

### Substrate scope

We next investigated the scope of unsaturated substrates and TMDQ reagents for this bis-silylation (Table 2). We found that PPh_3_ as the ligand in the model reaction resulted in good yield, and the other internal alkynes generally gave the bis-silylated products in low to moderate yields. To our delight, the reaction of TMDQ reagent **1a** and a wide variety of symmetric *para-* or *meta*-substituted diaryl alkynes with different electronic properties afforded the desired products in good to excellent yields with exclusive *cis*-selectivity (**3ab**–**al**) by use of SIPr as the ligand. Multi-substituted diaryl alkynes also provided the corresponding bis-silylated products in high yields (**3am**−**an**). Naphthalene-substituted alkynes could also successfully react with the TMDQ reagent to deliver bis-silylated olefins (**3ao**−**ap**). Importantly, this method was remarkably tolerant of various important functional groups on the phenyl ring of diaryl alkynes, such as F, CF_3_, COOEt, and OMe groups. Next, various symmetrical aliphatic alkynes were evaluated. We found that using 2-dicyclohexylphosphino-2′,6′-dimethoxybiphenyl (Sphos) as the ligand resulted in high yields with a low catalyst loading under milder reaction conditions (**3aq**–**at**). Furthermore, the electron-withdrawing internal alkyne dimethyl acetylenedicarboxylate as the substrate was also subjected to Ni-catalytic conditions. Unfortunately, it proved unreactive. In addition, the other TMDQ reagents produced from 8-bromoquinoline were also competent coupling partners, enabling access to the corresponding bis-silylated olefinic products (**3ba**−**da**). In addition to symmetrical alkynes, unsymmetrical internal alkynes were also investigated. Unfortunately, 1-phenylpropyne was inert under Ni-catalyzed standard conditions. To ensure reactivity and the regioisomeric ratio, we further screened a large set of reaction parameters, such as ligands, solvents, and disilane reagents, as shown in Supplementary Tables 2 and 3 in the Supplementary Information. Finally, disilylated product **3au** was obtained in 82% yield at 83:17 r.r., when BrettPhos was used as the ligand and DMF was used as the solvent. Moreover, we found that employment of TMDQ **1d** as the disilane reagent during the reaction further increased the regioisomeric ratio to 86:14 (**3du** in Table 2). Then, diverse aryl methyl acetylenes were evaluated under the modified conditions. In general, the aryl substituent with different electronic properties did not influence the yield or the regioisomeric ratio, generally giving the corresponding products (**3du**–**dz**) in good yields with good regioselectivities (up to 9:1). Phenyl propyl acetylene was also a suitable substrate, affording **3dä** in 65% yield with 73% regioselectivity. Notably, these two regioisomers were separable by column chromatography, which increases the practicality of this method. The structure of the major isomer was confirmed by X-ray crystal diffraction analysis of compound **3dz**.

**Table 2:**
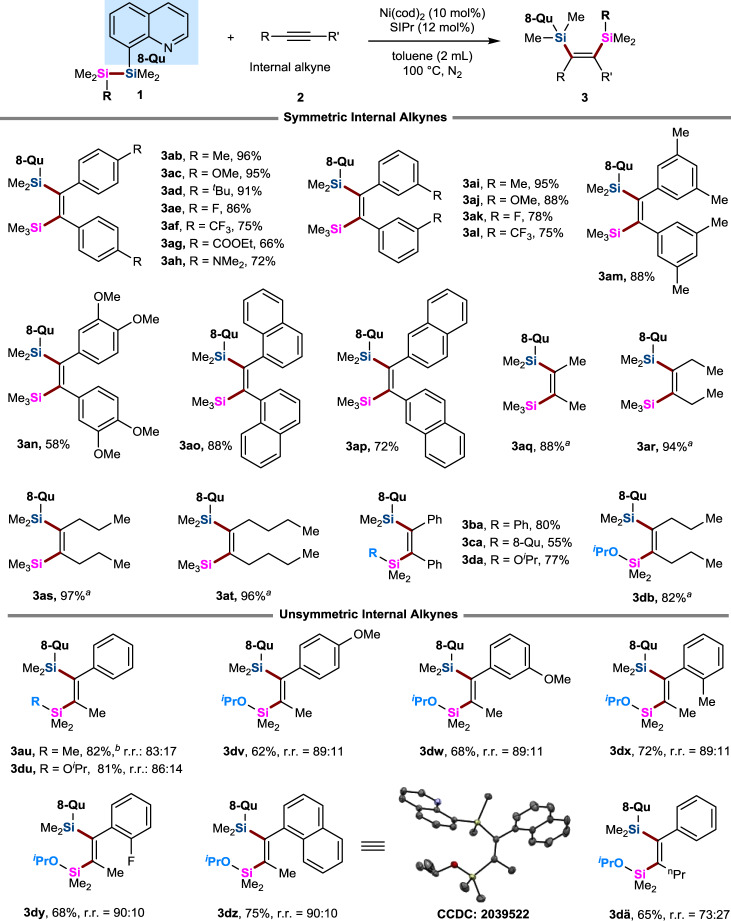
Substrate scope.

Reactions were carried out by using Ni(COD)_2_ (5.6 mg, 0.02 mmol), SIPr (9.4 mg, 0.024 mmol), **1** (0.2 mmol), alkyne **2** (0.4 mmol) in toluene (2 mL) at 100 °C under an N_2_ atmosphere.

^a^The reaction was carried out with Sphos (8.2 mg, 0.02 mmol), Ni(COD)_2_ (2.8 mg, 0.01 mmol) in toluene for 36 h at 100 °C.

^b^BrettPhos (0.04 mmol) was used as the ligand. Regioisomeric ratio (r.r.) were determined by 1H NMR.

**Fig. 3:**
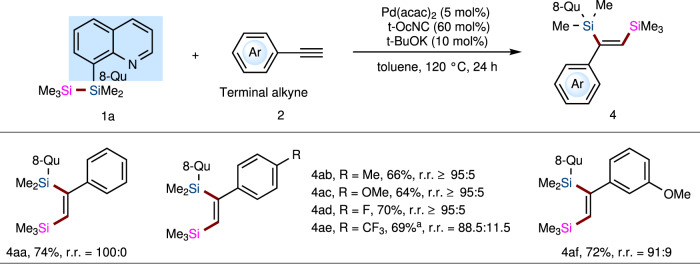
The bis-silylation reaction of TMDQ reagent 1 with terminal alkyne 2. Standard reaction conditions: disilane **1a** (52 mg, 0.2 mmol), terminal alkyne **2** (41 mg, 44 μL, 0.4 mmol), Pd(acac)_2_ (3 mg, 0.01 mmol), *t*-OcNC (17 mg, 0.12 mmol), and *t*-BuOK (2.3 mg, 0.02 mmol) in toluene (2 mL) at 120 °C under N_2_ atmosphere for 24 h. See Supplementary Information (SI) for details.

Following the success of Ni-catalyzed bis-silylation of internal alkynes with diverse TMDQ reagents, we proceeded to examine the terminal alkyne as the coupling partner. Unfortunately, in the optimized Ni-catalytic system, the reaction of TMDQ reagent **1a** and phenylacetylene did not proceed. Presumably, it might be because terminal alkynes are generally more active than internal alkynes and feasible to coordinate with the metal center more tightly by forming the Ni/alkyne (1:2) complex, leading to catalyst deactivation. Inspired by the Pd-catalytic system developed by Song, we replaced the Ni catalyst with a Pd-catalytic system for this reaction. After extensive screening, we found that the reaction of unsymmetric disilane **1a** with terminal phenylacetylene proceeded smoothly with Pd(acac)_2_ (5 mol%), *tert*-octylisocyanide (*t*-OcNC, 6 mol%), and *t-*BuOK (10 mol%) in toluene for 24 h at 120 °C, affording desired bis-silylated product **4aa** in 74% yield with complete regioselectivity (100:0 r.r.). Then, several aryl acetylenes were further tested for the bis-silylation reaction with TMDQ reagent **1a**, affording the corresponding products **4ab**–**af** (Fig. 3). Alkyl acetylene was unreactive. In general, our TMDQ reagent led to low yields and comparable regioselectivity relative to Song’s system for the bis-silylation reaction of terminal alkynes.

**Fig. 4:**
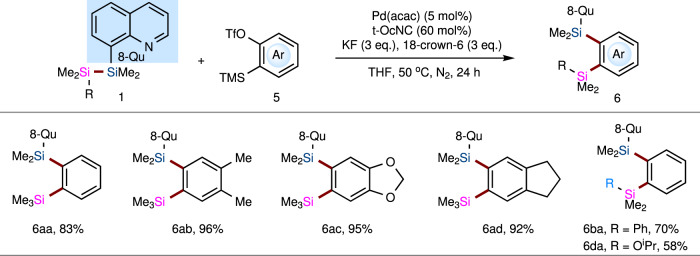
The bis-silylation reaction of TMDQ reagent 1 with aryne 5. Standard reaction conditions: TMDQ reagent **1** (0.2 mmol), aryne precursor **5** (0.4 mmol), Pd(acac)_2_ (3 mg, 0.01 mmol), *t*-OcNC (17 mg, 0.12 mmol), KF (35 mg, 0.6 mmol), and 18-crown-6 (159 mg, 0.6 mmol) in THF (2 mL) at 50 °C under N_2_ atmosphere for 24 h. See Supplementary Information (SI) for details.

The importance of arynes as useful reactive intermediates undergoing a wide variety of reactions has been well illustrated^39–41^. For example, in 2003, Yoshida and Kunai disclosed the palladium-catalyzed bis-silylation of arynes with strained cyclic disilanes^42^, which afforded diverse benzoannulated disilacarbocycles. However, until now, no report has been published on the addition of an acyclic disilane to arynes, which is an excellent and straightforward way to synthesize 1,2-disilyl-substituted benzenes that have been widely employed as starting materials for preparing diverse reagents and π-building blocks^43–46^. Following the success of the bis-silylation of internal and terminal alkynes with the TMDQ reagent, we set out to explore the application of this reagent in the bis-silylation of arynes to access 1,2-disilylbenzene derivatives that are usually obtained via the reaction of 1,2-dichlorobenzenes or 1,2-dibromobenzenes with chlorosilanes^47–49^. We investigated the reaction of TMDQ **1a** and an aryne generated in situ from 2-(trimethylsilyl)aryl triflate (**5a**) and a fluoride ion (KF/18-crown-6). However, no desired product was detected under the Ni-catalytic system. To our delight, the screening results showed that desired product **6aa** was obtained in 83% yield when the Pd-catalytic system was used (Supplementary Table 5 in Supplementary Information). We continued our studies by investigating the scope with respect to the aryne components (Fig. 4). The reaction of symmetric arynes with TMDQ reagent **1a** provided desired products **6aa**–**ad** in excellent yields. Moreover, TMDQ reagent **1d** also reacted smoothly with benzyne to produce 1,2-disilyl-substituted benzene **6da** in 58% yield.

**Fig. 5:**
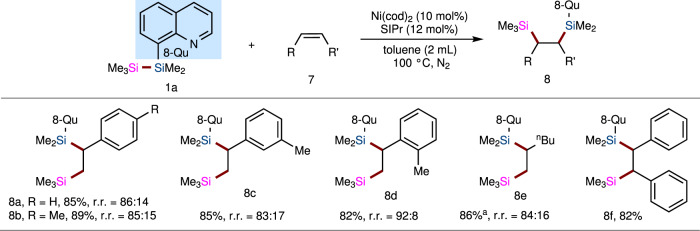
The bis-silylation reaction of TMDQ reagent 1a with alkenes 7. Standard reaction conditions: the mixture of Ni(COD)_2_ (5.6 mg, 0.02 mmol) and SIPr (9.5 mg, 0.024 mmol) in toluene (2 mL) was stirred for 30 min, then disilane reagent **1** (0.2 mmol) and specific alkene **7** (0.4–2 mmol) were added. The reaction was heated to 100 °C with vigorous stirring for 48 h under N_2_ atmosphere. See Supplementary Information (SI) for details. ^a^10.0 equiv alkene was used.

In addition to diverse alkynes, alkenes as substrates for bis-silylation with our disilane reagent **1a** have also been tested under Ni-catalyzed and Pd-catalyzed conditions. In fact, transition metal-catalyzed intermolecular bis-silylation is still a challenge. Generally, conjugated or strained double bonds are needed to ensure reactivity. After a few attempts at the reaction of **1a** with styrene **7a**, we found that the Pd catalyst was unreactive. In contrast, styrene **7a** successfully reacted with disilane **1a** in the Ni^0^-catalyzed reaction to afford the corresponding bis-silylated product **8a** in 85% yield and with a 86:14 regioisomeric ratio (Fig. 5). Next, we carried out the nickel-catalyzed bis-silylation of diverse alkenes with **1a**. First, all of the reactions with substrates bearing a substituent in the *para*-, *meta*-, or *ortho*-position proceeded well with good yields and regioselectivities (**8b–d**). Likewise, the reaction also proceeded well with 1-hexene, affording bis-silylated product **8e** in 86% yield and with 84:16 regioselectivity. Moreover, the scope of the reaction could be further extended to *cis*-bisaryl alkene (**8f**). However, *cis*-bisalkyl alkenes and *trans*-alkenes are currently not suitable under the optimized conditions.

### Synthetic utility

**Fig. 6:**
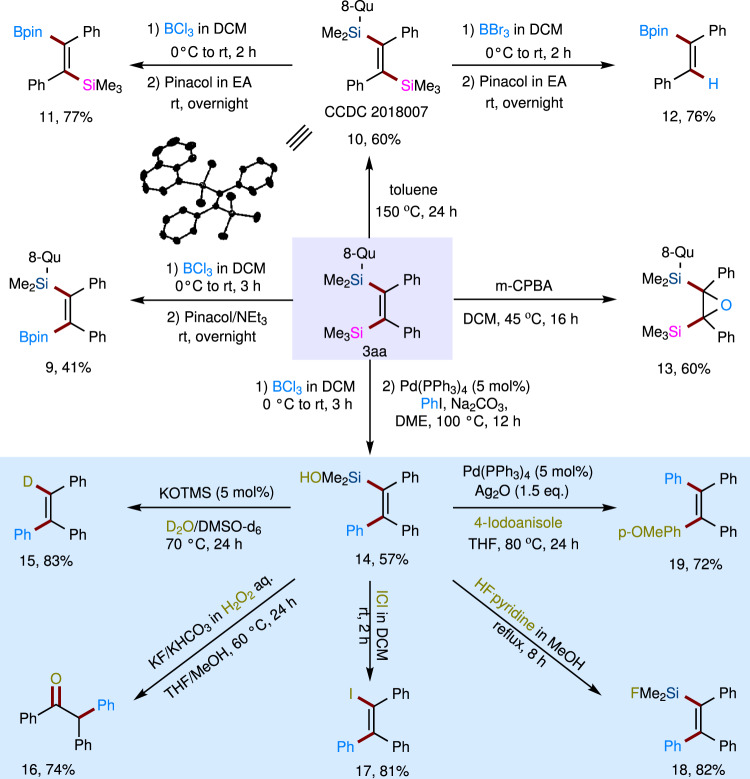
Synthetic utilities of product 3aa. The product **3aa** resulting from bis-silylation can be conveniently transformed to a variety of skeletons by programmable sequential synthesis. Yields are for isolated and purified products. The detailed procedures for these downstream transformations have been presented in Supplementary Information (SI).

Notably, all of these bis-silylated products are air-stable, can react on a gram scale and are easily purified on a silica column (1.18 g of **3aa**, 90%). To investigate the synthetic utility of the olefinic products obtained by our method, we took **3aa** as an example to prove that the two vicinal silyl substituents could be sufficiently differentiated in downstream transformations to achieve structurally diverse skeletons (Fig. 6). The TMS group on product **3aa** could be chemoselectively transformed to a Bpin group by treatment with BCl_3_ in DCM, followed by quenching with Pinacol at room temperature, yielding compound **9**. Treatment of **3aa** in toluene at 150 °C for 24 h led to *cis*-to-*trans* isomerization of the two vicinal silyl groups to yield product **10** in 60% yield, which was confirmed by X-ray crystal analysis. In contrast, the 8-QuMe_2_Si moiety on *trans*-product **10** preferentially reacted with BCl_3_ in DCM to yield compound **11**, in which the TMS group remained intact. Interestingly, by employing BBr_3_ in DCM, both desilylation and borylation of product **10** took place to afford tri-substituted alkenylborate **12** in 76% yield. In addition, *cis*-epoxidation of the C=C bond of **3aa** afforded product **13** in 60% yield by treatment with *m*-CPBA in DCM. Of note, because of the distinguishable reactivities of the two nonequivalent silyl groups on **3aa**, this compound could be utilized as a platform to regio- and stereoselectively transform into diverse tetrasubstituted olefins in a stepwise manner by a sequential assembly strategy. Triaryl-substituted alkenylsilanol **14** was first synthesized from **3aa** by sequential borodesilylation and Suzuki coupling. Then, the remaining Me_2_SiOH group on **14** originating from hydrolysis of the 8-Qu group during Suzuki coupling could further undergo diverse transformations, such as deuteration, Tamao’s oxidation, iodination, and fluorination, to achieve structurally diverse synthetic building blocks **15**, **16**, **17**, and **18**, respectively. Furthermore, the Hiyama−Denmark cross-coupling of **14** and 4-iodoanisole proceeded smoothly to give tetraaryl-substituted olefin **19** in 72% yield, which has been widely used as an AIE luminogen.

**Fig. 7:**
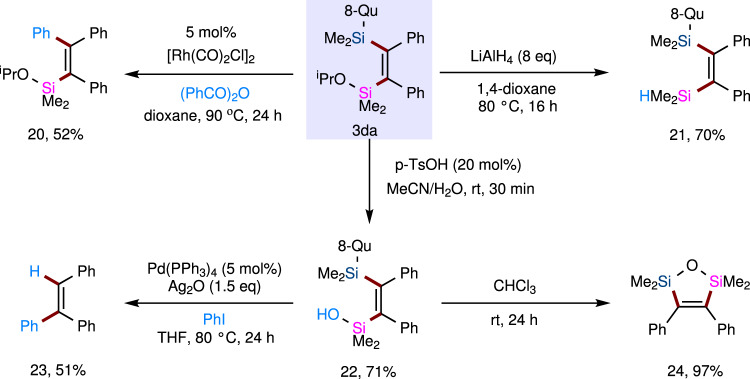
Synthetic utility of product 3da. These results indicate that the two vicinal silyl substituents on product **3da** can be sufficiently differentiated in downstream transformations to achieve structurally diverse skeletons. Yields are for isolated and purified products. The detailed procedures for these downstream transformations have been presented in Supplementary Information (SI).

The introduction of an alkoxy group attached to silicon generally modifies the reactivity of the corresponding silyl group. Thus, we wondered whether our product **3da** bearing an isopropoxy group on the silicon atom possesses richer and more colorful reactivities than compound **3aa** (Fig. 7). Indeed, there is no need for preborodesilylation for the cross-coupling of **3da** with aryl electrophiles. We show the cross-coupling of **3da** with benzoic anhydride to afford compound **20** under Rh^I^-catalytic conditions. The Si–O bond of product **3da** can be reduced by LiAlH_4_ to give the corresponding hydrosilane **21** (70% yield), which can further serve as an indispensable reagent in hydrosilylation. In addition, **3da** can also be easily hydrolyzed to yield the corresponding silanol **22** in 71% yield, which is also capable of cross-coupling with iodobenzene to produce compound **23** and cyclization to obtain compound **24** in 97% yield, which was synthesized by Au-catalyzed dehydrogenative cycloaddition of specific dihydrodisilanes to alkynes.

### Proposed mechanism

**Fig. 8:**
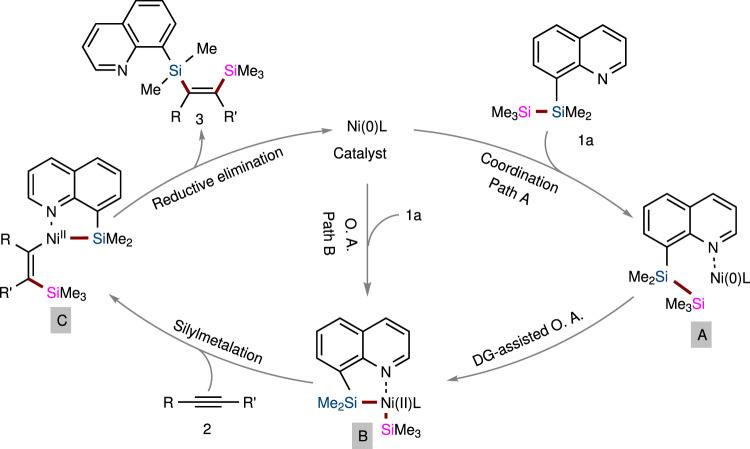
Proposed catalytic cycle. Specifically, **A**: transition state by coordination of Ni^0^ to the nitrogen atom of disilane TMDQ reagent; **B**: transition state after oxidative addition of Si−Si bond; **C**: transition state for silylmetalation of an internal alkyne.

Following the literature, we proposed a catalytic cycle for our study that begins with coordination of Ni^0^ to the nitrogen atom of disilane reagent **1a** to form intermediate **A**, which is followed by coordination-assisted oxidative addition of Si−Si bonds^50–52^, thus producing metallacycle **B** (Fig. 8, path A). Complex **B** further undergoes an intermolecular silylmetalation of an internal alkyne to deliver intermediate **C**, followed by reductive elimination to afford bis-silylated product **3** and regenerate the Ni^0^ catalyst. However, other proposals, such as Si−Si oxidative addition of the Ni(0) catalyst directly, followed by coordination of the N atom on the 8-Qu group to form intermediate **B** (Fig. 8, path B), cannot be ruled out at this stage.

In summary, we have developed an air-stable, strongly coordinating unsymmetrical disilane reagent, TMDQ, which is capable of bis-silylation of internal alkynes in a Ni^0^-catalytic system, leading to the synthesis of bis-silylated olefinic products, in which the two nonequivalent silyl groups can be sufficiently differentiated and transformed in a stepwise manner for further downstream transformations, making this synthetic protocol of great potential utility. The introduction of the 8-Qu group on the silicon atom at the unsymmetrical disilane and employment of a Ni(0)-catalytic system are crucial for adjusting the coordinating ability of the metal center and achieving higher reactivity.

## Method

### General procedure for Ni-catalyzed bis-silylation of internal alkynes with TMDQ reagent

In the nitrogen-filled glovebox, to an oven-dried 8-mL sealed tube equipped with a Teflon-coated magnetic stir bar were added Ni(COD)_2_ (5–10.0 mol%), ligand (10–12.0 mol%), toluene (2 mL), and the reaction mixture was stirred for 30 min, then TMDQ reagent **1** (0.2 mmol, 1.0 equiv.), internal alkynes **2** (0.4–2 mmol, 2.0–10 equiv.) were added. The vial was sealed with a screw-top septum cap, removed from the glovebox and placed in a heating block that was preheated to 100 °C with vigorous stirring for 36–48 h under N_2_ atmosphere. After been cooled to room temperature, the reaction mixture was filtered through a pad of celite and concentrated in vacuo. The resulting residue was purified by silica gel flash chromatography to give the desired product **3**.

## Supplementary information

Supplementary Information

## Data Availability

The authors declare that the data supporting the findings of this study are available within the article and its Supplementary Information files, as well as from the corresponding authors on request. The X-ray crystallographic coordinates for structures reported in this study have been deposited at the Cambridge Crystallographic Data Centre (CCDC), under deposition numbers CCDC 2009970 (**3aa**), CCDC 2039522 (**3dz**), and CCDC 2018007 (**10**). These data can be obtained free of charge from The Cambridge Crystallographic Data Centre via www.ccdc.cam.ac.uk/data_request/cif.
