# Supplementary material for: Bis-silylation of internal alkynes enabled by Ni(0) catalysis
Source: Nat Commun. 2021 Jan 4;12:68. doi: 10.1038/s41467-020-20392-w (PMC7782505; doi:10.1038/s41467-020-20392-w)
Supplement: Supplementary file 1 — Supplementary Information [file 41467_2020_20392_MOESM1_ESM.pdf]

Supplementary Information

Bis-silylation of internal alkynes enabled by Ni(0) catalysis

Yun Zhang, Xi-Chao Wang, Cheng-Wei Ju, and Dongbing Zhao*
State Key Laboratory and Institute of Elemento-Organic Chemistry, College of Chemistry, Nankai University, Tianjin 300071, China
E-mail: dongbing.chem@nankai.edu.cn

Table of Contents

General Information 1

Synthesis of Disilane Reagents TMDQ 1

Nickel(0)-Catalyzed Bissilylation of Internal Alkynes 6

Bissilylation of Symmetric Internal Alkynes 6

Bissilylation of Unsymmetric Internal Alkynes 26

Pd-Catalyzed Bissilylation of Terminal Alkynes 39

Pd-Catalyzed Bissilylation of Aryne Precursors 45

Nickel(0)-Catalyzed Bissilylation of Alkenes 50

Synthetic Transformations 56

Gram-scale experiments 56

Synthetic utilities 57

Supplementary References 66

NMR Spectra 69

Supplementary Methods

General Information

Unless otherwise noted, all reactions were set up using standard Schlenk techniques and carried out under a N_2 atmosphere with dry solvents. Commercially available reagents were received from commercial sources without further purification and Dry solvents (<50 ppm H_2O) were purchased and stored over molecular sieves under N_2 atmosphere and were transferred under N_2.

NMR spectra were recorded on Bruker AV 400 spectrometer at 400 MHz (^{1}H NMR), 100 MHz (^{13}C NMR), 376 MHz (^{19}F NMR) using $CDCl_3$ or DMSO-d_6 as solvent. The residual solvent signals were used as references for ^{1}H and ^{13}C NMR spectra and the chemical shifts converted to the TMS scale ($CDCl_3$: δ_H = 7.26 ppm, δ_C = 77.16 ppm; $(CD_3)_2SO$: δ_H = 2.50 ppm, δ_C = 39.52 ppm).

GC-MS spectra was obtained using electron ionization (Thermo Scientific Trace 300/GC-System and ISQ/QD). High resolution mass spectra (HRMS) of product were recorded on Varian 7.0T FTMS with ESI resource or Q Exactive GC-Orbitrap MS. Thin-layer chromatography was performed on pre-coated silica gel 60 F_{254} plates. TLC plates were visualized by exposure to short wave ultraviolet light (254 nm). Silica gel 60H (200-300 mesh) manufactured by Qingdao Haiyang Chemical Group Co. (China) was used for general chromatography. Compounds **3au–dą, 8aa–ae & 18** presented in this paper were purified on C18(ODS) column (5µm, 21.2×250 mm) with H_2O/MeCN by preparative RP-HPLC with a Bonna-Agela CHEETAH HP series.

Synthesis of Disilane Reagents TMDQ

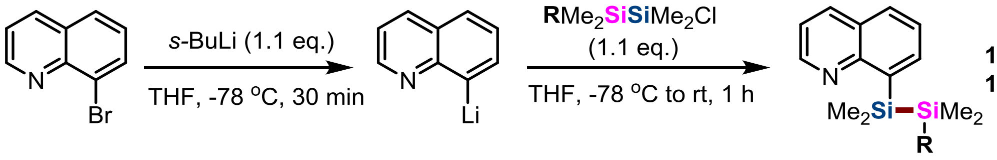

1a, **R** = Me
1b, **R** = Ph

Following a modified literature procedure,[1] *sec*-butyllithium (1.3 M in cyclohexane, 17 mL, 22 mmol) was added dropwise *via* syringe to a magnetically stirred solution of 8-bromoquinoline (4.2 g, 20 mmol) in 50.0 mL of tetrahydrofuran at -78 ℃ over 5 min, followed by stirring for an additional 30 min at this temperature, $Me_2RSiSiMe_2Cl$ (22 mmol) was added subsequently at -78 ℃, then the reaction was allowed to warm to room temperature for additional 1 h. The reaction was quenched with sat. NH_4Cl aq. (20 mL) and extracted with Et_2O (3×20 mL). The combined organic layers were then dried over Na_2SO_4, filtered and concentrated under reduced pressure. The residues were purified by silica gel flash column chromatography (PE) to afford disilane reagent TMDQ **1a** & **1b**.

8-(1,1,2,2,2-Pentamethyldisilanyl)quinoline (1a)

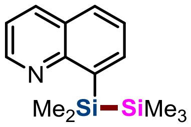

Following the general procedure to give the disilane reagent **1a** (3.21 g, 62% yield) as a colorless oil. **R*f*** (PE): 0.6. **^{1}H NMR (400 MHz, CDCl$_3$)** δ 8.90 (dd, *J* = 4.3, 1.8 Hz, 1H), 8.13 (dd, *J* = 8.2, 1.8 Hz, 1H), 7.84 (dd, *J* = 6.8, 1.5 Hz, 1H), 7.79 (dd, *J* = 8.3, 1.5 Hz, 1H), 7.52 (dd, *J* = 8.1, 6.7 Hz, 1H), 7.37 (dd, *J* = 8.2, 4.2 Hz, 1H), 0.45 (s, 6H). 0.02 (s, 9H). **^{13}C NMR (101 MHz, CDCl$_3$)** δ 152.5, 148.7, 142.7, 136.0, 135.6, 128.5, 127.5, 126.3, 120.8, -1.18, -2.8. **HRMS (ESI)** m/z Calcd. For $C_{14}H_{22}NSi_2$ $(M+H)^+$: 260.1285, found 260.1285.

8-(1,1,2,2-Tetramethyl-2-phenyldisilanyl)quinoline (1b)

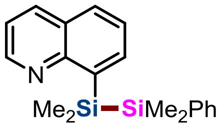

Following the general procedure to give the disilane reagent **1b** (3.53 g, 55% yield) as a colorless viscous liquid. **R*f*** (PE/DCM = 20/1): 0.4. **^{1}H NMR (400 MHz, CDCl$_3$)** δ 8.85 (td, *J* = 4.4, 1.9 Hz, 1H), 8.12 (dt, *J* = 8.2, 2.0 Hz, 1H), 7.88 – 7.83 (m, 1H), 7.81 (ddd, *J* = 8.1, 3.1, 1.5 Hz, 1H), 7.55 – 7.45 (m, 3H), 7.37 (ddd, *J* = 8.2, 4.2, 2.1 Hz, 1H), 7.33 – 7.27 (m, 3H), 0.47 (d, *J* = 6.9 Hz, 6H), 0.36 (d, *J* = 7.1 Hz, 6H). **^{13}C NMR (101 MHz, CDCl$_3$)** δ 152.4, 148.7, 142.1, 141.0, 136.1, 135.7, 134.0, 128.7, 128.0, 127.5, 126.3, 120.9, -2.5, -2.7. **HRMS (ESI)** m/z Calcd. For $C_{19}H_{24}NSi_2$ $(M+H)^+$: 322.1442, found 322.1444.

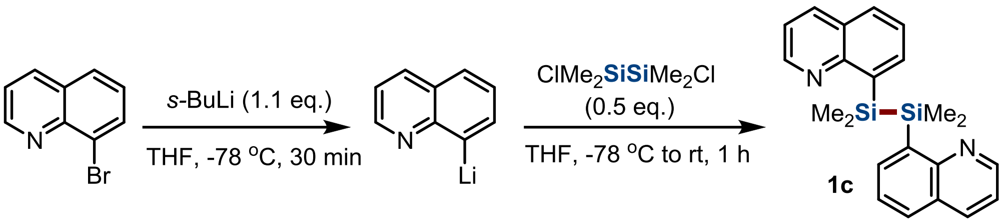

Following a modified literature procedure,[1] *sec*-butyllithium (1.3 M in cyclohexane, 17 mL, 22 mmol, 1.1 equiv) was added dropwise *via* syringe to a magnetically stirred solution of 8-bromoquinoline (4.2 g, 20 mmol, 1 equiv) in 50.0 mL of tetrahydrofuran at -78 ℃ over 5 min, followed by stirring for an additional 30 min at this temperature. To a solution of $ClMe_2SiSiMe_2Cl$ (10 mmol) in THF (20 mL) was added dropwise the above cold organolithium reagent at -78 ℃ over 5 min, then the mixture was allowed to warm to room temperature and stirred for additional 1 h. The reaction was quenched with sat. NH_4Cl aq. (20 mL) and extracted with Et_2O (3 × 20 mL). The combined organic layers were then dried over Na_2SO_4, filtered and concentrated under reduced pressure. The residues were purified by silica gel flash column chromatography (PE/DCM = 2/1) to afford disilane reagent **1c**.

1,1,2,2-Tetramethyl-1,2-di(quinolin-8-yl)disilane (1c)

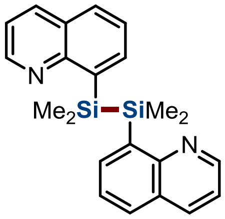

Following the procedure to give the disilane reagent **1c** (2.15 g, 58% yield) as a yellow solid. **R*f*** (PE/EA = 20/1): 0.4. **^{1}H NMR (400 MHz, $CDCl_3$)** δ 8.80 (dd, *J* = 4.2, 1.8 Hz, 2H), 8.03 (dd, *J* = 8.3, 1.8 Hz, 2H), 7.76 (dd, *J* = 6.9, 1.5 Hz, 2H), 7.68 (dd, *J* = 8.0, 1.4 Hz, 2H), 7.41 (dd, *J* = 8.1, 6.7 Hz, 2H), 7.30 (dd, *J* = 8.2, 4.2 Hz, 2H), 0.42 (s, 12H). **^{13}C NMR (101 MHz, $CDCl_3$)** δ 152.3, 148.5, 143.9, 135.8, 135.3, 128.0, 127.2, 126.1, 120.6, -2.3. **HRMS (ESI)** m/z Calcd. For $C_{22}H_{25}N_2Si_2$ $(M+H)^+$: 373.1551, found 373.1549.

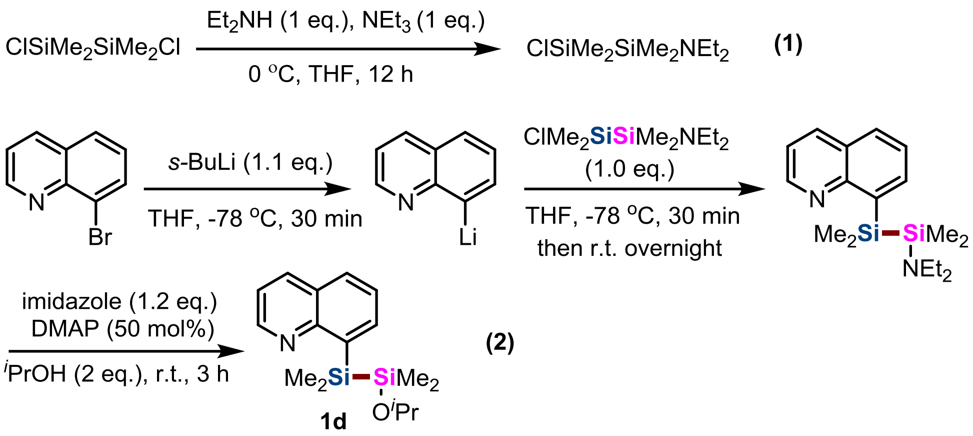

Step 1: Following a modified literature procedure,[2] to a solution of $ClSiMe_2SiMe_2Cl$ (5.6 g, 30 mmol) and Et_3N (3.1 g, 4.3 mL, 30 mmol, 1.0 eq.) in THF (60 mL) was added a solution of Et_2NH (2.2 g, 3.1 mL, 30 mmol) in THF (25 mL) at 0 ℃ over 20 min. The reaction mixture was stirred at 0 ℃ for 12 h. and then 1 h with warming the mixture up to room temperature. To the mixture was added hexane, filtered, washed with hexane, concentration of the filtrate under reduced pressure yielded the crude chlorosilane reagent (4.9 g, 22 mmol, 73%), which was taken directly to the next step without further purification.

Step 2: *sec*-Butyllithium (1.3 M in cyclohexane, 19 mL, 24.2 mmol) was added dropwise *via* syringe to a magnetically stirred solution of 8-bromoquinoline (4.6 g, 22 mmol) in 50.00 mL of tetrahydrofuran at -78 ℃ over 5 min, followed by stirring for an additional 30 min at this temperature, and this cold suspension was added dropwise to a solution of $ClMe_2SiSiMe_2NEt_2$ (22 mmol) in THF (20 mL) at -78 ℃ over 30 min, then the mixture was allowed to warm to room temperature and stirred overnight. Without work-up, imidazole (1.7 g, 24.2 mmol), DMAP (1.3 g, 11 mmol, 50 mol%) and ^{i}PrOH (2.6 g, 3.4 mL, 44 mmol, 2 eq.) was added sequentially to this solution and stirred at room temperature for further 3 h. The reaction was quenched with sat. NH_4Cl aq. (20 mL) and extracted with Et_2O (3×20 mL). The combined organic layers were then dried over Na_2SO_4, filtered and concentrated under reduced pressure. The residue was distilled under reduced pressure (125 ℃/0.5 mmHg) to give disilane reagent **1d**.

8-(2-Isopropoxy-1,1,2,2-tetramethyldisilanyl)quinoline (1d)

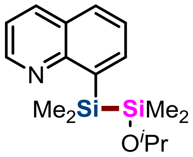

Following the general procedure B to give the disilane reagent **1d** (3.4 g, 50%) as a pale-yellow viscous liquid. **R*f*** (PE/DCM = 5/1): 0.4. **^{1}H NMR (400 MHz, $CDCl_3$)** δ 8.87 (dd, *J* = 4.2, 1.8 Hz, 1H), 8.11 (dd, *J* = 8.2, 1.9 Hz, 1H), 7.88 (dd, *J* = 6.8, 1.5 Hz, 1H), 7.80 (dd, *J* = 8.1, 1.5 Hz, 1H), 7.53 (dd, *J* = 8.1, 6.7 Hz, 1H), 7.36 (dd, *J* = 8.2, 4.2 Hz, 1H), 3.89 (hept, *J* = 6.0 Hz, 1H), 1.02 (d, *J* = 6.1 Hz, 6H), 0.52 (s, 6H), 0.24 (s, 6H). **^{13}C NMR (101 MHz, $CDCl_3$)** δ 152.3, 148.6, 142.3, 136.1, 135.7, 128.6, 127.5, 126.4, 120.9, 65.7, 25.9, 0.9, -2.4. **HRMS (ESI)** m/z Calcd. for $C_{16}H_{26}NOSi_2$ $[M+H]^+$ 304.1547, Found 304.1546.

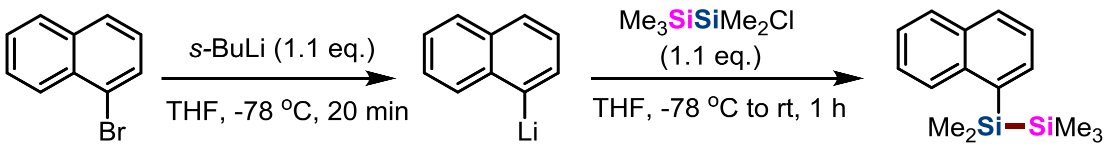

Following a modified literature procedure,[1] *n*-BuLi (1.5 M in hexane, 7.4 mL, 11 mmol) was added dropwise *via* syringe to a magnetically stirred solution of 1-bromonaphthalene (2.1 g, 10 mmol) in 30.0 mL of Tetrahydrofuran at -78 ℃ over 5 min, followed by stirring for an additional 20 min at this temperature, $Me_3SiSiMe_2Cl$ (11 mmol) was added subsequently at -78 ℃, then the reaction was allowed to warm to room temperature for additional 1 h. The reaction was quenched with sat. NH_4Cl aq. (20 mL) and extracted with Et_2O (3×20 mL). The combined organic layers were then dried over Na_2SO_4, filtered and concentrated under reduced pressure. The residue was purified by silica gel flash column chromatography (PE) to afford disilane reagent **1f**.

1,1,1,2,2-Pentamethyl-2-(naphthalen-1-yl)disilane (1f)

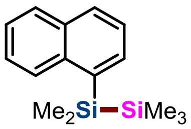

Following the general procedure C to give the product **1c** (2.2 g, 85% yield) as a colorless oil. **R*f*** (PE): 0.6. **^{1}H NMR (400 MHz, $CDCl_3$)** δ 8.06 – 7.98 (m, 1H), 7.92 – 7.82 (m, 2H), 7.72 – 7.63 (m, 1H), 7.56 – 7.42 (m, 3H), 0.55 (d, *J* = 4.8 Hz, 6H), 0.11 (d, *J* = 4.9 Hz, 9H). **^{13}C NMR (101 MHz, $CDCl_3$)** δ 138.1, 137.3, 133.4, 129.4, 129.1, 128.7, 128.0, 125.9, 125.5, 125.4, 125.4, -1.4, -2.2. **HRMS (EI)** calcd. For $C_{15}H_{22}Si_2$ $(M)^+$: 258.1260, found 258.1254.

Nickel(0)-Catalyzed Bissilylation of Internal Alkynes

Bissilylation of Symmetric Internal Alkynes

Supplementary Table 1. Optimization of reaction conditions[a]

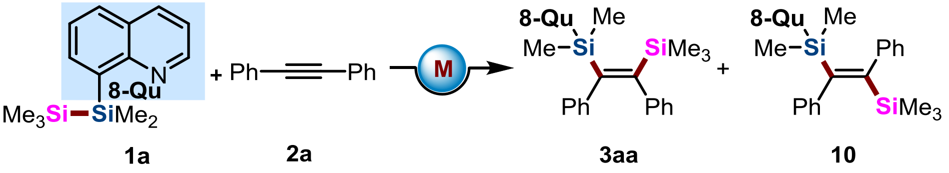

Entry	Catalyst	Ligand	Additive	Yield[%][b]	3aa:10[c]
1[d]	Pd(acac)$_2$ (4 mol%)	^{t}BuNC (60 mol%)	^{t}BuOK (10 mol%)	N.R.	N.D.
2	Ni(COD)$_2$ (10 mol%)	**L1** (12 mol%)	^{t}BuOK (12 mol%)	70	75:25
3	Ni(COD)$_2$ (10 mol%)	SIPr·HCl (12 mol%)	^{t}BuOK (12 mol%)	92	88:12
4	Ni(COD)$_2$ (10 mol%)	SIPr·HBF$_4$ (12 mol%)	^{t}BuOK (12 mol%)	92	84:16
5	Ni(COD)$_2$ (10 mol%)	SIMes·HBF$_4$ (12 mol%)	^{t}BuOK (12 mol%)	trace	N.D.
6[e]	Ni(COD)$_2$ (10 mol%)	SIPr·HCl (12 mol%)	^{t}BuOK (12 mol%)	88	100:0
7[e, f]	Ni(COD)$_2$ (10 mol%)	SIPr·HCl (12 mol%)	^{t}BuOK (12 mol%)	88	100:0
8[e, g]	Ni(COD)$_2$ (10 mol%)	SIPr·HCl (12 mol%)	^{t}BuOK (12 mol%)	82	100:0
9[e, f]	Ni(COD)$_2$ (10 mol%)	IPr·HCl (12 mol%)	^{t}BuOK (12 mol%)	34	100:0
10[e, f]	Ni(COD)$_2$ (10 mol%)	ICy·HCl (12 mol%)	^{t}BuOK (12 mol%)	20	100:0
11[e, f]	Ni(COD)$_2$ (10 mol%)	SIPr (12 mol%)	none	98	100:0
12[e, f]	Ni(COD)$_2$ (10 mol%)	PPh$_3$ (20 mol%)	none	92	100:0
13[e, f]	Ni(COD)$_2$ (5 mol%)	SIPr (6 mol%)	none	92	100:0

[a] Reactions were carried out with [M] precatalyst, ligand, additive, disilane **1a** (0.20 mmol) and diphenylacetylene **2a** (0.60 mmol, 3 eq.) in toluene for 48 h at 130 ℃ under an N_2 atmosphere. [b] Yields of isolated products. [c] Ratios were determined by ^{1}H NMR spectroscopy of the crude mixture. [d] The reaction was conducted at 120 ℃. [e] The reaction was conducted at 100 ℃. [f] **2a** (0.4 mmol, 2 eq.) was used. [g] **2a** (0.3 mmol, 1.5 eq.) was used. N.R.: No reaction (N.R.). N.D.: not determined. **L1**: (4R,5R)-4,5-diphenyl-1,3-di-o-tolyl-4,5-dihydro-1H-imidazol-3-ium tetrafluoroborate.

General procedure: In the nitrogen-filled glovebox, to an oven-dried 8-mL sealed tube equipped with a Teflon-coated magnetic stir bar were added Ni(COD)$_2$ (5–10.0 mol%), ligand (10–12.0 mol%), toluene (2 mL), and the reaction mixture was stirred for 30 min, then disilane reagent **1** (0.2 mmol, 1.0 equiv), symmetric internal alkynes **2** (0.4–2

mmol, 2.0–10 equiv) were added. The vial was sealed with a screw-top septum cap, removed from the glovebox and placed in a heating block that was pre-heated to 100 ℃ with vigorous stirring for 36–48 h under N_2 atmosphere. After been cooled to room temperature, the reaction mixture was filtered through a pad of celite and concentrated in vacuo. The resulting residue was purified by silica gel flash chromatography to give the desired product **3aa**–**db**.

(*Z*)-8-((1,2-Diphenyl-2-(trimethylsilyl)vinyl)dimethylsilyl)quinoline (3aa)

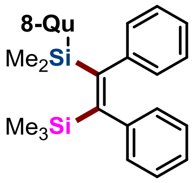

Following the general procedure, in the nitrogen-filled glovebox, to an oven-dried 8-mL sealed tube equipped with a Teflon-coated magnetic stir bar were added Ni(COD)$_2$ (5.6 mg, 0.02 mmol, 10.0 mol%), SIPr (9.5 mg, 0.024 mmol, 12.0 mol%), toluene (2 mL), and the reaction mixture was stirred for 30 min, then disilane reagent **1a** (52 mg, 0.2 mmol, 1.0 equiv), diphenylethyne **2a** (71.2 mg, 0.4 mmol, 2.0 equiv) were added. The vial was sealed with a screw-top septum cap, removed from the glovebox and placed in a heating block that was pre-heated to 100 ℃ with vigorous stirring for 48 h under N_2 atmosphere. After been cooled to room temperature, the reaction mixture was filtered through a pad of celite and concentrated in vacuo. The resulting residue was purified by silica gel flash chromatography (PE/DCM = 20/1) to give the desired product **3aa** (85.6 mg, 98% yield, white solid). **R_f** (PE/DCM = 20/1): 0.4. **^{1}H NMR (400 MHz, CDCl$_3$)** δ 9.28 (dd, *J* = 4.2, 1.9 Hz, 1H), 8.47 (dd, *J* = 21.4, 6.9 Hz, 2H), 8.16 (d, *J* = 8.1 Hz, 1H), 7.92 – 7.82 (m, 1H), 7.70 (dd, *J* = 8.2, 4.2 Hz, 1H), 7.33 – 7.24 (m, 4H), 7.23 – 7.12 (m, 4H), 7.00 (d, *J* = 7.2 Hz, 2H), 0.73 (s, 6H), 0.01 (s, 9H). **^{13}C NMR (101 MHz, CDCl$_3$)** δ 157.9, 157.8, 152.6, 148.8, 147.1, 146.9, 141.3, 137.4, 136.1, 129.5, 128.3, 128.2, 128.0, 127.0, 126.9, 126.0, 124.2, 124.19, 120.9, 1.5, 1.2. **HRMS (ESI)** m/z Calcd. for $C_{28}H_{32}NSi_2$ $[M+H]^+$ 438.2068, Found 438.2068.

(*Z*)-2-((1,2-Diphenyl-2-(trimethylsilyl)vinyl)dimethylsilyl)-*N*,*N*-diisopropyl-benzamide

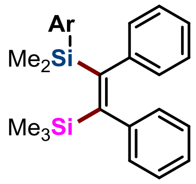

Ar = *o*-CON(*i*Pr)$_2$C$_6$H$_4$

Following the general procedure, in the nitrogen-filled glovebox, to an oven-dried 8-mL sealed tube equipped with a Teflon-coated magnetic stir bar were added Ni(COD)$_2$ (5.6 mg, 0.02 mmol, 10.0 mol%), SIPr (9.5 mg, 0.024 mmol, 12.0 mol%), toluene (2 mL), and the reaction mixture was stirred for 30 min, then disilane reagent **1f** (67 mg, 0.2 mmol, 1.0 equiv), diphenylethyne **2a** (71.2 mg, 0.4 mmol, 2.0 equiv) were added. The vial was sealed with a screw-top septum cap, removed from the glovebox and placed in a heating block that was pre-heated to 100 ℃ with vigorous stirring for 48 h under N_2 atmosphere. After been cooled to room temperature, the reaction mixture was filtered through a pad of celite and concentrated in vacuo. The resulting residue was purified by silica gel flash chromatography (PE/EA = 45/1) to give the desired product (35 mg, 34% yield, pale yellow viscous liquid). **R*f*** (PE/EA = 45/1): 0.4. **^{1}H NMR (400 MHz, CDCl$_3$)** δ 8.14 (dd, *J* = 7.3, 1.1 Hz, 1H), 7.43 (td, *J* = 7.4, 1.2 Hz, 1H), 7.37 (td, *J* = 7.5, 1.4 Hz, 1H), 7.21 (d, *J* = 6.8 Hz, 1H), 7.02 (t, *J* = 7.6 Hz, 2H), 6.95 (t, *J* = 7.6 Hz, 2H), 6.88 (t, *J* = 7.4 Hz, 1H), 6.82 (t, *J* = 7.4 Hz, 1H), 6.74 (dd, *J* = 12.1, 7.1 Hz, 4H), 3.85 (dt, *J* = 12.8, 6.4 Hz, 1H), 3.49 (dt, *J* = 13.3, 6.6 Hz, 1H), 1.56 (d, *J* = 6.7 Hz, 6H), 1.16 (d, *J* = 6.3 Hz, 6H), 0.31 (s, 6H), -0.08 (s, 9H). **^{13}C NMR (101 MHz, CDCl$_3$)** δ 172.1, 160.4, 157.2, 146.8, 146.3, 143.9, 138.2, 137.4, 128.5, 128.4, 128.2, 127.9, 127.1, 127.1, 125.8, 124.5, 124.3, 50.82, 45.9, 21.0, 20.6, 2.4, 1.4. **HRMS (ESI)** m/z Calcd. for $C_{32}H_{44}NOSi_2$ $[M+H]^+$ 514.2956, Found 514.2960.

(*Z*)-8-((1,2-Di-*p*-tolyl-2-(trimethylsilyl)vinyl)dimethylsilyl)quinoline (3ab)

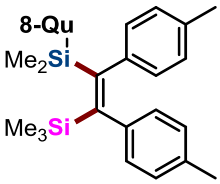

Following the general procedure, in the nitrogen-filled glovebox, to an oven-dried 8-mL sealed tube equipped with a Teflon-coated magnetic stir bar were added Ni(COD)$_2$ (5.6 mg, 0.02 mmol, 10.0 mol%), SIPr (9.5 mg, 0.024 mmol, 12.0 mol%), toluene (2 mL), and the reaction mixture was stirred for 30 min, then disilane reagent **1a** (52 mg, 0.2 mmol, 1.0 equiv), 1,2-di-*p*-tolylethyne **2b** (82.4 mg, 0.4 mmol, 2.0 equiv) were added. The

vial was sealed with a screw-top septum cap, removed from the glovebox and placed in a heating block that was pre-heated to 100 ℃ with vigorous stirring for 48 h under N_2 atmosphere. After been cooled to room temperature, the reaction mixture was filtered through a pad of celite and concentrated in vacuo. The resulting residue was purified by silica gel flash chromatography (PE/DCM = 20/1) to give the desired product **3ab** (89 mg, 96% yield, white solid). **R*f*** (PE/DCM = 20/1): 0.4. **^{1}H NMR (400 MHz, $CDCl_3$)** δ 8.99 (dd, *J* = 4.1, 1.8 Hz, 1H), 8.24 (dd, *J* = 6.8, 1.5 Hz, 1H), 8.15 (dd, *J* = 8.2, 1.9 Hz, 1H), 7.87 (dd, *J* = 8.1, 1.5 Hz, 1H), 7.61 (dd, *J* = 8.1, 6.7 Hz, 1H), 7.40 (dd, *J* = 8.2, 4.2 Hz, 1H), 6.89 – 6.79 (m, 6H), 6.64 (d, *J* = 8.0 Hz, 2H), 2.18 (s, 3H), 2.16 (s, 3H), 0.45 (s, 6H), -0.27 (s, 9H). **^{13}C NMR (101 MHz, $CDCl_3$)** δ 158.1, 157.5, 152.6, 148.8, 144.1, 143.9, 141.5, 137.5, 136.1, 133.3, 133.2, 129.4, 128.1, 128.1, 127.9, 127.8, 127.7, 126.0, 120.9, 21.2, 1.7, 1.3. **HRMS (ESI)** m/z Calcd. for $C_{30}H_{36}NSi_2$ $[M+H]^+$ 466.2381, Found 466.2382.

(*Z*)-8-((1,2-Bis(4-methoxyphenyl)-2-(trimethylsilyl)vinyl)dimethylsilyl)quinoline (3ac)

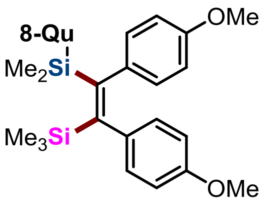

Following the general procedure, in the nitrogen-filled glovebox, to an oven-dried 8-mL sealed tube equipped with a Teflon-coated magnetic stir bar were added $Ni(COD)_2$ (5.6 mg, 0.02 mmol, 10.0 mol%), SIPr (9.5 mg, 0.024 mmol, 12.0 mol%), toluene (2 mL), and the reaction mixture was stirred for 30 min, then disilane reagent **1a** (52 mg, 0.2 mmol, 1.0 equiv), 1,2-bis(4-methoxyphenyl)ethyne **2c** (95.2 mg, 0.4 mmol, 2.0 equiv) were added. The vial was sealed with a screw-top septum cap, removed from the glovebox and placed in a heating block that was pre-heated to 100 ℃ with vigorous stirring for 48 h under N_2 atmosphere. After been cooled to room temperature, the reaction mixture was filtered through a pad of celite and concentrated in vacuo. The resulting residue was purified by silica gel flash chromatography (PE/DCM = 5/1) to give the desired product **3ac** (94 mg, 95% yield, white solid). **R*f*** (PE/DCM = 5/1): 0.3. **^{1}H NMR (400 MHz, $CDCl_3$)** δ 8.98 (dd, *J* = 4.1, 1.8 Hz, 1H), 8.20 (dd, *J* = 6.7, 1.5 Hz, 1H), 8.14 (dd, *J* = 8.3, 1.8 Hz, 1H), 7.86 (dd, *J* = 8.2, 1.5 Hz,

1H), 7.59 (dd, J = 8.1, 6.7 Hz, 1H), 7.39 (dd, J = 8.2, 4.1 Hz, 1H), 6.85 – 6.79 (m, 2H), 6.66 – 6.53 (m, 6H), 3.68 (s, 3H), 3.66 (s, 3H), 0.45 (s, 6H), -0.28 (s, 9H). **^{13}C NMR (101 MHz, CDCl$_3$)** δ 158.1, 157.7, 156.3, 156.3, 152.6, 148.8, 141.4, 139.7, 139.5, 137.4, 136.1, 129.4, 129.2, 129.1, 127.9, 126.0, 120.9, 112.5, 112.4, 55.0, 55.0, 1.6, 1.3. **HRMS (ESI)** m/z Calcd. for $C_{30}H_{36}NO_2Si_2$ $[M+H]^+$ 498.2279, Found 498.2278.

(*Z*)-8-((1,2-Bis(4-(*tert*-butyl)phenyl)-2-(trimethylsilyl)vinyl)dimethylsilyl)quinoline (**3ad**)

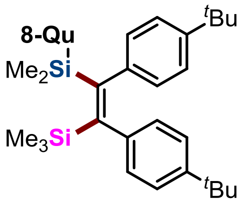

Following the general procedure, in the nitrogen-filled glovebox, to an oven-dried 8-mL sealed tube equipped with a Teflon-coated magnetic stir bar were added Ni(COD)$_2$ (5.6 mg, 0.02 mmol, 10.0 mol%), SIPr (9.5 mg, 0.024 mmol, 12.0 mol%), toluene (2 mL), and the reaction mixture was stirred for 30 min, then disilane reagent **1a** (52 mg, 0.2 mmol, 1.0 equiv), 1,2-bis(4-(*tert*-butyl)phenyl)ethyne **2d** (116 mg, 0.4 mmol, 2.0 equiv) were added. The vial was sealed with a screw-top septum cap, removed from the glovebox and placed in a heating block that was pre-heated to 100 ℃ with vigorous stirring for 48 h under N$_2$ atmosphere. After been cooled to room temperature, the reaction mixture was filtered through a pad of celite and concentrated in vacuo. The resulting residue was purified by silica gel flash chromatography (PE/DCM = 20/1) to give the desired product **3ad** (100 mg, 91% yield, white solid). **R*$_f$*** (PE/DCM = 20/1): 0.4. **^{1}H NMR (400 MHz, CDCl$_3$)** δ 8.98 (dd, J = 4.1, 1.9 Hz, 1H), 8.24 (dd, J = 6.8, 1.5 Hz, 1H), 8.14 (dd, J = 8.3, 1.9 Hz, 1H), 7.86 (dd, J = 8.1, 1.5 Hz, 1H), 7.60 (dd, J = 8.1, 6.7 Hz, 1H), 7.40 (dd, J = 8.3, 4.1 Hz, 1H), 7.00 – 6.91 (m, 4H), 6.76 (d, J = 8.4 Hz, 2H), 6.59 (d, J = 8.3 Hz, 2H), 1.17 (s, 9H), 1.15 (s, 9H), 0.47 (s, 6H), -0.26 (s, 9H). **^{13}C NMR (101 MHz, CDCl$_3$)** δ 158.5, 158.0, 152.7, 148.8, 146.6, 146.5, 144.1, 143.9, 141.6, 137.5, 136.1, 129.4, 128.0, 127.9, 127.9, 126.0, 123.5, 123.4, 120.9, 34.2, 34.2, 31.5, 31.4, 1.7, 1.3. **HRMS (ESI)** m/z Calcd. for $C_{36}H_{48}NSi_2$ $[M+H]^+$ 550.3320, Found 550.3318.

(*Z*)-8-((1,2-Bis(4-fluorophenyl)-2-(trimethylsilyl)vinyl)dimethylsilyl)quinoline (3ae)

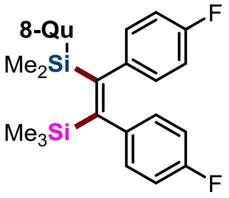

Following the general procedure, in the nitrogen-filled glovebox, to an oven-dried 8-mL sealed tube equipped with a Teflon-coated magnetic stir bar were added Ni(COD)$_2$ (5.6 mg, 0.02 mmol, 10.0 mol%), SIPr (9.5 mg, 0.024 mmol, 12.0 mol%), toluene (2 mL), and the reaction mixture was stirred for 30 min, then disilane reagent **1a** (52 mg, 0.2 mmol, 1.0 equiv), 1,2-bis(4-fluorophenyl)ethyne **2e** (85.6 mg, 0.4 mmol, 2.0 equiv) were added. The vial was sealed with a screw-top septum cap, removed from the glovebox and placed in a heating block that was pre-heated to 100 ℃ with vigorous stirring for 48 h under N_2 atmosphere. After been cooled to room temperature, the reaction mixture was filtered through a pad of celite and concentrated in vacuo. The resulting residue was purified by silica gel flash chromatography (PE/DCM = 20/1) to give the desired product **3ae** (81 mg, 86% yield, white solid). **R*f*** (PE/DCM = 20/1): 0.4. **^{1}H NMR (400 MHz, CDCl$_3$)** δ 9.14 (dd, *J* = 4.2, 1.8 Hz, 1H), 8.30 (ddd, *J* = 19.9, 7.5, 1.7 Hz, 2H), 8.03 (dd, *J* = 8.1, 1.5 Hz, 1H), 7.79 – 7.69 (m, 1H), 7.58 (dd, *J* = 8.2, 4.1 Hz, 1H), 7.04 – 6.95 (m, 2H), 6.94 – 6.81 (m, 4H), 6.80 – 6.72 (m, 2H), 0.59 (s, 6H), -0.15 (s, 9H). **^{13}C NMR (101 MHz, CDCl$_3$)** δ 160.2 (d, *J* = 240 Hz), 158.1, 157.7, 152.5, 148.8, 142.8 (d, *J* = 3.0 Hz), 142.7 (d, *J* = 3.0 Hz), 140.9, 137.2, 136.2, 129.6 (d, *J* = 8.0 Hz), 129.4 (d, *J* = 8.0 Hz), 129.3, 128.0, 126.1, 121.0, 114.0 (d, *J* = 21.0 Hz), 113.9 (d, *J* = 20.0 Hz), 1.3, 1.0. **^{19}F NMR (377 MHz, CDCl$_3$)** δ -119.26, -119.28, -119.29, -119.30, -119.32, -119.33, -119.34, -119.35, -119.37, -119.38. **HRMS (ESI)** m/z Calcd. for $C_{28}H_{30}F_2NSi_2$ [M+H]$^+$ 474.1879, Found 474.1877.

(*Z*)-8-((1,2-Bis(4-(trifluoromethyl)phenyl)-2-(trimethylsilyl)vinyl)dimethylsilyl)q-uinoline (3af)

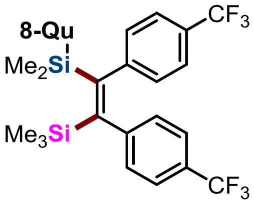

Following the general procedure, in the nitrogen-filled glovebox, to an oven-dried 8-mL sealed tube equipped with a Teflon-coated magnetic stir bar were added Ni(COD)$_2$ (5.6 mg, 0.02 mmol, 10.0 mol%), SIPr (9.5 mg, 0.024 mmol, 12.0 mol%), toluene (2 mL),

and the reaction mixture was stirred for 30 min, then disilane reagent **1a** (52 mg, 0.2 mmol, 1.0 equiv), 1,2-bis(4-(trifluoromethyl)phenyl)ethyne **2f** (125.6 mg, 0.4 mmol, 2.0 equiv) were added. The vial was sealed with a screw-top septum cap, removed from the glovebox and placed in a heating block that was pre-heated to 100 ℃ with vigorous stirring for 48 h under N_2 atmosphere. After been cooled to room temperature, the reaction mixture was filtered through a pad of celite and concentrated in vacuo. The resulting residue was purified by silica gel flash chromatography (PE/DCM = 20/1) to give the desired product **3af** (86 mg, 75% yield, white solid). **R_f** (PE/DCM = 20/1): 0.4. **^{1}H NMR (400 MHz, $CDCl_3$)** δ 8.98 (dd, *J* = 4.2, 1.8 Hz, 1H), 8.16 (dd, *J* = 8.3, 1.8 Hz, 1H), 8.07 (dd, *J* = 6.8, 1.5 Hz, 1H), 7.87 (dd, *J* = 8.1, 1.5 Hz, 1H), 7.57 (dd, *J* = 8.1, 6.8 Hz, 1H), 7.44 (dd, *J* = 8.2, 4.1 Hz, 1H), 7.28 – 7.19 (m, 4H), 7.01 (d, *J* = 7.8 Hz, 2H), 6.74 (d, *J* = 7.8 Hz, 2H), 0.41 (s, 6H), -0.35 (s, 9H). **^{13}C NMR (101 MHz, $CDCl_3$)** δ 158.0, 157.1, 152.4, 150.6, 148.8, 140.3, 137.1, 136.4, 130.8, 129.8, 129.7, 128.3, 128.1, 126.8 (q, *J* = 32 Hz), 126.8 (q, *J* = 32 Hz), 126.1, 124.5 (q, *J* = 270 Hz), 124.4 (q, *J* = 270 Hz), 124.3 (q, *J* = 4.0 Hz), 124.1 (q, *J* = 4.0 Hz), 121.2, 1.0, 0.9. **^{19}F NMR (377 MHz, $CDCl_3$)** δ -62.23, -62.24. **HRMS (ESI)** m/z Calcd. for $C_{30}H_{30}F_6NSi_2$ $[M+H]^+$ 574.1815, Found 574.1818.

Diethyl 4,4'-(1-(dimethyl(quinolin-8-yl)silyl)-2-(trimethylsilyl)ethene-1,2-diyl)(*Z*)-dibenzoate (3ag)

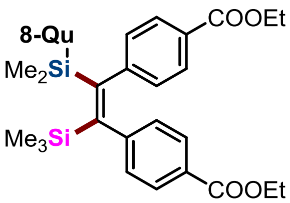

Following the general procedure, in the nitrogen-filled glovebox, to an oven-dried 8-mL sealed tube equipped with a Teflon-coated magnetic stir bar were added Ni(COD)$_2$ (5.6 mg, 0.02 mmol, 10.0 mol%), SIPr (9.5 mg, 0.024 mmol, 12.0 mol%), toluene (2 mL), and the reaction mixture was stirred for 30 min, then disilane reagent **1a** (52 mg, 0.2 mmol, 1.0 equiv), diethyl 4,4'-(ethyne-1,2-diyl)dibenzoate **2g** (128.8 mg, 0.4 mmol, 2.0 equiv) were added. The vial was sealed with a screw-top septum cap, removed from the glovebox and placed in a heating block that was pre-heated to 100 ℃ with vigorous stirring for 48 h under N_2 atmosphere. After been cooled to room temperature, the reaction mixture was filtered through a pad of celite and

concentrated in vacuo. The resulting residue was purified by silica gel flash chromatography (PE/DCM = 4/1) to give the desired product **3ag** (77 mg, 66% yield, white solid). **R*f*** (PE/DCM = 2/1): 0.4. **^{1}H NMR (400 MHz, $CDCl_3$)** δ 8.99 (dd, *J* = 4.1, 1.9 Hz, 1H), 8.15 (dd, *J* = 8.3, 1.9 Hz, 1H), 8.10 (dd, *J* = 6.8, 1.5 Hz, 1H), 7.87 (dd, *J* = 8.1, 1.5 Hz, 1H), 7.74 – 7.65 (m, 4H), 7.57 (dd, *J* = 8.1, 6.8 Hz, 1H), 7.42 (dd, *J* = 8.1, 4.2 Hz, 1H), 7.01 (d, *J* = 8.0 Hz, 2H), 6.74 (d, *J* = 8.1 Hz, 2H), 4.27 (qd, *J* = 7.2, 1.6 Hz, 4H), 1.33 (t, *J* = 7.1 Hz, 6H), 0.41 (s, 6H), -0.35 (s, 9H). **^{13}C NMR (101 MHz, $CDCl_3$)** δ 166.9, 166.8, 157.9, 157.2, 152.4, 152.1, 152.0, 148.8, 140.4, 137.1, 136.2, 129.7, 128.7, 128.5, 128.1, 128.0, 127.9, 126.6, 126.6, 126.1, 121.1, 60.7, 60.7, 14.4, 1.1, 0.9. **HRMS (ESI)** m/z Calcd. for $C_{34}H_{40}NO_4Si_2$ $[M+H]^+$ 582.2490, Found 582.2492.

(*Z*)-4,4'-(1-(Dimethyl(quinolin-8-yl)silyl)-2-(trimethylsilyl)ethene-1,2-diyl)bis(*N*, *N*-dimethylaniline) (3ah)

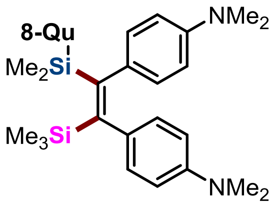

Following the general procedure, in the nitrogen-filled glovebox, to an oven-dried 8-mL sealed tube equipped with a Teflon-coated magnetic stir bar were added $Ni(COD)_2$ (5.6 mg, 0.02 mmol, 10.0 mol%), SIPr (9.5 mg, 0.024 mmol, 12.0 mol%), toluene (2 mL), and the reaction mixture was stirred for 30 min, then disilane reagent **1a** (52 mg, 0.2 mmol, 1.0 equiv), 4,4'-(ethyne-1,2-diyl)bis(*N*,*N*-dimethylaniline) **2h** (105.6 mg, 0.4 mmol, 2.0 equiv) were added. The vial was sealed with a screw-top septum cap, removed from the glovebox and placed in a heating block that was pre-heated to 100 ℃ with vigorous stirring for 48 h under N_2 atmosphere. After been cooled to room temperature, the reaction mixture was filtered through a pad of celite and concentrated in vacuo. The resulting residue was purified by silica gel flash chromatography (PE/EA = 4/1) to give the desired product **3ah** (75 mg, 72% yield, white solid). **R*f*** (PE/EA = 2/1): 0.4. **^{1}H NMR (400 MHz, $CDCl_3$)** δ 8.96 (dd, *J* = 4.2, 1.9 Hz, 1H), 8.06 (dd, *J* = 8.2, 1.9 Hz, 1H), 7.59 (dd, *J* = 8.1, 1.6 Hz, 1H), 7.36 (dd, *J* = 8.1, 4.1 Hz, 1H), 7.28 – 7.25 (m, 1H), 7.22 – 7.15 (m, 3H), 6.73 (d, *J* = 8.6 Hz, 2H), 6.20 (d, *J* = 8.5 Hz, 2H), 6.09 (d, *J* = 8.6 Hz, 2H), 2.95 (s, 6H), 2.80 (s, 6H), 0.13 (s,

6H), -0.40 (s, 9H). **^{13}C NMR (101 MHz, $CDCl_3$)** δ 159.2, 158.3, 152.2, 148.9, 148.3, 148.2, 142.1, 135.6, 135.6, 135.2, 134.4, 129.5, 128.5, 127.9, 127.4, 126.1, 120.4, 112.2, 111.8, 41.2, 41.1, 0.6, 0.2. **HRMS (ESI)** m/z Calcd. for $C_{32}H_{42}N_3Si_2$ $[M+H]^+$ 524.2912, Found 524.2912.

(*Z*)-8-((1,2-Di-*m*-tolyl-2-(trimethylsilyl)vinyl)dimethylsilyl)quinoline (3ai)

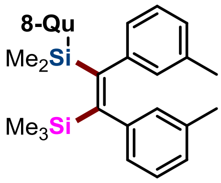

Following the general procedure, in the nitrogen-filled glovebox, to an oven-dried 8-mL sealed tube equipped with a Teflon-coated magnetic stir bar were added Ni(COD)$_2$ (5.6 mg, 0.02 mmol, 10.0 mol%), SIPr (9.5 mg, 0.024 mmol, 12.0 mol%), toluene (2 mL), and the reaction mixture was stirred for 30 min, then disilane reagent **1a** (52 mg, 0.2 mmol, 1.0 equiv), 1,2-di-*o*-tolylethyne **2i** (82.4 mg, 0.4 mmol, 2.0 equiv) were added. The vial was sealed with a screw-top septum cap, removed from the glovebox and placed in a heating block that was pre-heated to 100 ℃ with vigorous stirring for 48 h under N_2 atmosphere. After been cooled to room temperature, the reaction mixture was filtered through a pad of celite and concentrated in vacuo. The resulting residue was purified by silica gel flash chromatography (PE/DCM = 20/1) to give the desired product **3ai** (88 mg, 95% yield, white solid). **R*f*** (PE/DCM = 20/1): 0.4. **^{1}H NMR (400 MHz, $CDCl_3$)** δ 9.01 (dt, *J* = 4.1, 2.0 Hz, 1H), 8.25 (d, *J* = 6.1 Hz, 1H), 8.16 (d, *J* = 7.9 Hz, 1H), 7.88 (d, *J* = 8.0 Hz, 1H), 7.66 – 7.58 (m, 1H), 7.42 (ddd, *J* = 8.2, 4.2, 2.0 Hz, 1H), 6.91 (q, *J* = 8.2 Hz, 2H), 6.74 – 6.64 (m, 4H), 6.61 – 6.50 (m, 2H), 2.19 (s, 3H), 2.16 (s, 3H), 0.48 (s, 6H), -0.23 (s, 9H). **^{13}C NMR (101 MHz, $CDCl_3$)** δ 158.0, 157.5, 152.6, 148.8, 146.9, 146.8, 141.5, 137.5, 136.1, 136.1, 135.9, 129.4, 129.2, 128.0, 126.7, 126.6, 126.0, 125.3, 125.3, 124.8, 124.8, 120.9, 21.5, 1.6, 1.3. **HRMS (ESI)** m/z Calcd. for $C_{30}H_{36}NSi_2$ $[M+H]^+$ 466.2381, Found 466.2383.

(*Z*)-8-((1,2-Bis(3-methoxyphenyl)-2-(trimethylsilyl)vinyl)dimethylsilyl)quinoline (3aj)

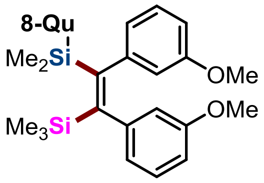

Following the general procedure, in the nitrogen-filled glovebox, to an oven-dried 8-mL sealed tube equipped with a Teflon-coated magnetic stir bar were added Ni(COD)$_2$ (5.6 mg, 0.02

mmol, 10.0 mol%), SIPr (9.5 mg, 0.024 mmol, 12.0 mol%), toluene (2 mL), and the reaction mixture was stirred for 30 min, then disilane reagent **1a** (52 mg, 0.2 mmol, 1.0 equiv), 1,2-bis(2-methoxyphenyl)ethyne **2j** (95.2 mg, 0.4 mmol, 2.0 equiv) were added. The vial was sealed with a screw-top septum cap, removed from the glovebox and placed in a heating block that was pre-heated to 100 ℃ with vigorous stirring for 48 h under N_2 atmosphere. After been cooled to room temperature, the reaction mixture was filtered through a pad of celite and concentrated in vacuo. The resulting residue was purified by silica gel flash chromatography (PE/DCM = 5/1) to give the desired product **3aj** (87 mg, 88% yield, white solid). **R*f*** (PE/DCM = 5/1): 0.4. **^{1}H NMR (400 MHz, $CDCl_3$)** δ 9.00 (dd, *J* = 4.2, 1.8 Hz, 1H), 8.16 (ddd, *J* = 17.7, 7.5, 1.7 Hz, 2H), 7.87 (dd, *J* = 8.1, 1.5 Hz, 1H), 7.59 (dd, *J* = 8.1, 6.7 Hz, 1H), 7.40 (dd, *J* = 8.2, 4.1 Hz, 1H), 6.95 (td, *J* = 7.9, 4.0 Hz, 2H), 6.63 – 6.48 (m, 2H), 6.49 – 6.41 (m, 2H), 6.41 – 6.25 (m, 2H), 3.66 (s, 3H), 3.63 (s, 3H), 0.48 (d, *J* = 17.3 Hz, 6H), -0.26 (s, 9H). **^{13}C NMR (101 MHz, $CDCl_3$)** δ 158.5, 158.4, 157.4, 152.5, 148.7, 148.4, 148.3, 141.1, 137.3, 136.1, 129.5, 128.0, 127.9, 127.8, 126.0, 121.0, 120.9, 114.1, 114.0, 110.0, 109.7, 55.1, 55.1, 1.4, 1.3, 1.2. **HRMS (ESI)** m/z Calcd. for $C_{30}H_{36}NO_2Si_2$ $[M+H]^+$ 498.2279, Found 498.2281.

(*Z*)-8-((1,2-Bis(3-fluorophenyl)-2-(trimethylsilyl)vinyl)dimethylsilyl)quinoline (3ak)

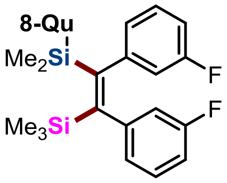

Following the general procedure, in the nitrogen-filled glovebox, to an oven-dried 8-mL sealed tube equipped with a Teflon-coated magnetic stir bar were added $Ni(COD)_2$ (5.6 mg, 0.02 mmol, 10.0 mol%), SIPr (9.5 mg, 0.024 mmol, 12.0 mol%), toluene (2 mL), and the reaction mixture was stirred for 30 min, then disilane reagent **1a** (52 mg, 0.2 mmol, 1.0 equiv), 1,2-bis(2-fluorophenyl)ethyne **2k** (85.6 mg, 0.4 mmol, 2.0 equiv) were added. The vial was sealed with a screw-top septum cap, removed from the glovebox and placed in a heating block that was pre-heated to 100 ℃ with vigorous stirring for 48 h under N_2 atmosphere. After been cooled to room temperature, the reaction mixture was filtered through a pad of celite and concentrated in vacuo. The resulting residue was purified by silica gel flash chromatography (PE/DCM = 20/1) to give the desired product **3ak**

(74 mg, 78% yield, white solid). **R$_f$** (PE/DCM = 20/1): 0.4. **^{1}H NMR (400 MHz, CDCl$_3$)** δ 9.01 (dd, *J* = 4.3, 1.8 Hz, 1H), 8.16 (dd, *J* = 8.3, 1.8 Hz, 1H), 8.10 (dd, *J* = 6.7, 1.4 Hz, 1H), 7.88 (dd, *J* = 8.1, 1.4 Hz, 1H), 7.58 (dd, *J* = 8.1, 6.8 Hz, 1H), 7.44 (dd, *J* = 8.3, 4.1 Hz, 1H), 6.97 (t, *J* = 7.5 Hz, 2H), 6.69 (s, 2H), 6.58 (t, *J* = 8.6 Hz, 2H), 6.45 (d, *J* = 7.6 Hz, 1H), 6.39 (d, *J* = 10.0 Hz, 1H), 0.46 (d, *J* = 7.2 Hz, 3H), 0.38 (d, *J* = 10.6 Hz, 3H), -0.34 (s, 9H). **^{13}C NMR (101 MHz, CDCl$_3$)** δ 162.2 (d, *J* = 243 Hz), 161.0, 157.6, 156.9, 152.4, 149.1 (d, *J* = 11.0 Hz), 149.1 (d, *J* = 11.0 Hz), 148.9, 140.6, 137.2, 136.2, 129.7, 128.6 (d, *J* = 4.0 Hz), 128.4 (d, *J* = 4.0 Hz), 128.0, 126.1, 123.9, 121.1, 115.1 (d, *J* = 20.0 Hz), 114.8 (d, *J* = 20.0 Hz), 111.4, 111.2, 1.2, 0.9. **^{19}F NMR (377 MHz, CDCl$_3$)** δ -114.80 (dd, *J* = 57.2, 7.7 Hz), -115.12 (dd, *J* = 71.3, 7.8 Hz). **HRMS (ESI)** m/z Calcd. for $C_{28}H_{30}F_2NSi_2$ $[M+H]^+$ 474.1879, Found 474.1880.

(*Z*)-8-((1,2-Bis(3-(trifluoromethyl)phenyl)-2-(trimethylsilyl)vinyl)dimethylsilyl)quinoline (3al)

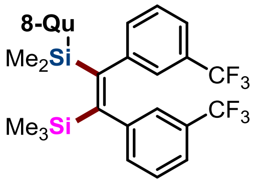

Following the general procedure, in the nitrogen-filled glovebox, to an oven-dried 8-mL sealed tube equipped with a Teflon-coated magnetic stir bar were added Ni(COD)$_2$ (5.6 mg, 0.02 mmol, 10.0 mol%), SIPr (9.5 mg, 0.024 mmol, 12.0 mol%), toluene (2 mL), and the reaction mixture was stirred for 30 min, then disilane reagent **1a** (52 mg, 0.2 mmol, 1.0 equiv), 1,2-bis(2-(trifluoromethyl)phenyl)ethyne **2l** (125.6 mg, 0.4 mmol, 2.0 equiv) were added. The vial was sealed with a screw-top septum cap, removed from the glovebox and placed in a heating block that was pre-heated to 100 ℃ with vigorous stirring for 48 h under N_2 atmosphere. After been cooled to room temperature, the reaction mixture was filtered through a pad of celite and concentrated in vacuo. The resulting residue was purified by silica gel flash chromatography (PE/DCM = 20/1) to give the desired product **3al** (86 mg, 75% yield, white solid). **R$_f$** (PE/DCM = 20/1): 0.4. **^{1}H NMR (400 MHz, CDCl$_3$)** δ 9.02 (dd, *J* = 4.2, 1.8 Hz, 1H), 8.17 (dd, *J* = 8.3, 1.8 Hz, 1H), 8.06 (dd, *J* = 6.8, 1.5 Hz, 1H), 7.88 (dd, *J* = 8.1, 1.5 Hz, 1H), 7.58 (dd, *J* = 8.1, 6.7 Hz, 1H), 7.46 (dd, *J* = 8.3, 4.1 Hz, 1H), 7.22 (d, *J* = 20.1 Hz, 1H), 7.13 – 7.03 (m, 5H), 6.92 – 6.75 (m, 2H), 0.45 (dd, *J* = 46.4, 6.5 Hz, 6H), -0.30 (s, 9H). **^{13}C NMR (101 MHz,**

CDCl3) δ 158.6, 157.4, 152.3, 148.8, 147.4, 140.3, 137.0, 136.3, 131.1, 129.8, 128.1, 127.6, 127.4, 126.2, 125.4, 124.8 (q. *J* = 4.0 Hz), 121.3 (q, *J* = 4.0 Hz), 121.3, 1.2, 0.8. **^{19}F NMR (377 MHz, CDCl3)** δ -62.80 (d, *J* = 14.0 Hz), -63.02 (d, *J* = 14.0 Hz). **HRMS (ESI)** m/z Calcd. for $C_{30}H_{30}F_6NSi_2$ $[M+H]^+$ 574.1815, Found 574.1815.

(*Z*)-8-((1,2-Bis(3,5-dimethylphenyl)-2-(trimethylsilyl)vinyl)dimethylsilyl)quinoline (3am)

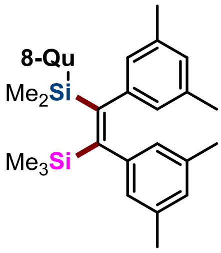

Following the general procedure, in the nitrogen-filled glovebox, to an oven-dried 8-mL sealed tube equipped with a Teflon-coated magnetic stir bar were added Ni(COD)$_2$ (5.6 mg, 0.02 mmol, 10.0 mol%), SIPr (9.5 mg, 0.024 mmol, 12.0 mol%), toluene (2 mL), and the reaction mixture was stirred for 30 min, then disilane reagent **1a** (52 mg, 0.2 mmol, 1.0 equiv), 1,2-bis(3,5-dimethylphenyl)ethyne **2m** (93.6 mg, 0.4 mmol, 2.0 equiv) were added. The vial was sealed with a screw-top septum cap, removed from the glovebox and placed in a heating block that was pre-heated to 100 ℃ with vigorous stirring for 48 h under N_2 atmosphere. After been cooled to room temperature, the reaction mixture was filtered through a pad of celite and concentrated in vacuo. The resulting residue was purified by silica gel flash chromatography (PE/DCM = 20/1) to give the desired product **3am** (87 mg, 88% yield, white solid). **R*f*** (PE/DCM = 20/1): 0.4. **^{1}H NMR (400 MHz, CDCl3)** δ 8.99 (dd, *J* = 4.1, 1.8 Hz, 1H), 8.26 (dd, *J* = 6.6, 1.5 Hz, 1H), 8.15 (dd, *J* = 8.2, 1.9 Hz, 1H), 7.88 (d, *J* = 1.5 Hz, 1H), 7.63 (dd, *J* = 8.1, 6.8 Hz, 1H), 7.41 (dd, *J* = 8.2, 4.1 Hz, 1H), 6.50 (d, *J* = 4.2 Hz, 3H), 6.47 (s, 1H), 6.37 (d, *J* = 1.5 Hz, 2H), 2.16 (s, 6H), 2.11 (s, 6H), 0.48 (s, 6H), -0.21 (s, 9H). **^{13}C NMR (101 MHz, CDCl3)** δ 158.2, 157.3, 152.7, 148.8, 146.8, 146.6, 141.6, 137.6, 136.1, 135.7, 135.6, 129.3, 127.9, 126.2, 126.2, 126.0, 125.6, 125.5, 120.9, 21.4, 1.8, 1.4. **HRMS (ESI)** m/z Calcd. for $C_{32}H_{40}NSi_2$ $[M+H]^+$ 494.2694, Found 494.2695.

(*Z*)-8-((1,2-Bis(3,4-dimethoxyphenyl)-2-(trimethylsilyl)vinyl)dimethylsilyl)quinoline (3an)

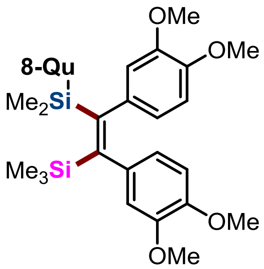

Following the general procedure, in the nitrogen-filled glovebox, to an oven-dried 8-mL sealed tube equipped with a Teflon-coated magnetic stir bar were added Ni(COD)$_2$ (5.6 mg, 0.02 mmol, 10.0 mol%), SIPr (9.5 mg, 0.024 mmol, 12.0 mol%), toluene (2 mL), and the reaction mixture was stirred for 30 min, then disilane reagent **1a** (52 mg, 0.2 mmol, 1.0 equiv), 1,2-bis(3,4-dimethoxyphenyl)ethyne **2n** (119.2 mg, 0.4 mmol, 2.0 equiv) were added. The vial was sealed with a screw-top septum cap, removed from the glovebox and placed in a heating block that was pre-heated to 100 ℃ with vigorous stirring for 48 h under N_2 atmosphere. After been cooled to room temperature, the reaction mixture was filtered through a pad of celite and concentrated in vacuo. The resulting residue was purified by silica gel flash chromatography (PE/EA = 3/1) to give the desired product **3an** (65 mg, 58% yield, white solid). **R*f*** (PE/EA = 1/1): 0.4. **^{1}H NMR (400 MHz, CDCl$_3$)** δ 8.95 (dd, *J* = 4.1, 1.8 Hz, 1H), 8.14 (dd, *J* = 8.3, 1.8 Hz, 1H), 8.10 (dd, *J* = 6.8, 1.5 Hz, 1H), 7.84 (dd, *J* = 8.2, 1.5 Hz, 1H), 7.56 (dd, *J* = 8.1, 6.7 Hz, 1H), 7.39 (dd, *J* = 8.2, 4.1 Hz, 1H), 6.52 (dd, *J* = 9.5, 8.2 Hz, 2H), 6.43 (d, *J* = 8.7 Hz, 2H), 6.29 – 6.13 (m, 2H), 3.74 (s, 3H), 3.72 (s, 3H), 3.68 (s, 3H), 3.61 (s, 3H), 0.44 (s, 6H), -0.28 (s, 9H). **^{13}C NMR (101 MHz, CDCl$_3$)** δ 157.8, 157.7, 152.5, 148.6, 147.7, 147.6, 145.9, 145.8, 141.4, 139.9, 139.8, 137.2, 136.2, 129.4, 128.0, 126.0, 120.9, 120.11, 112.6, 109.8, 55.9, 55.7, 55.7, 1.6, 1.3. **HRMS (ESI)** m/z Calcd. for $C_{32}H_{40}NO_4Si_2$ $[M+H]^+$ 558.2490, Found 558.2490.

(Z)-8-((1,2-Di(naphthalenyl)-2-(trimethylsilyl)vinyl)dimethylsilyl)quinoline (3ao)

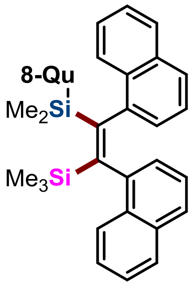

Following the general procedure, in the nitrogen-filled glovebox, to an oven-dried 8-mL sealed tube equipped with a Teflon-coated magnetic stir bar were added Ni(COD)$_2$ (5.6 mg, 0.02 mmol, 10.0 mol%), SIPr (9.5 mg, 0.024 mmol, 12.0 mol%), toluene (2 mL), and the reaction mixture was stirred for 30 min, then disilane reagent **1a** (52 mg, 0.2 mmol, 1.0 equiv), 1,2-di(naphthalen-1-yl)ethyne **2o** (111.2 mg, 0.4 mmol, 2.0 equiv)

were added. The vial was sealed with a screw-top septum cap, removed from the glovebox and placed in a heating block that was pre-heated to 100 ℃ with vigorous stirring for 48 h under N_2 atmosphere. After been cooled to room temperature, the reaction mixture was filtered through a pad of celite and concentrated in vacuo. The resulting residue was purified by silica gel flash chromatography (PE/DCM = 4/1) to give the desired product **3ao** (95 mg, 88% yield, white solid). **R*f*** (PE/DCM = 1/1): 0.4. **^{1}H NMR (400 MHz, CDCl$_3$)** δ 9.23 (dd, *J* = 4.2, 1.8 Hz, 1H), 8.51 (dd, *J* = 8.4, 1.1 Hz, 1H), 8.24 (dd, *J* = 8.2, 1.8 Hz, 1H), 8.21 (dd, *J* = 6.8, 1.5 Hz, 1H), 8.03 (dd, *J* = 8.1, 1.5 Hz, 1H), 7.92 (dd, *J* = 8.1, 1.4 Hz, 1H), 7.65 – 7.53 (m, 5H), 7.41 – 7.31 (m, 3H), 7.28 – 7.25 (m, 1H), 7.21 (d, *J* = 8.2 Hz, 1H), 7.15 (dd, *J* = 7.2, 1.2 Hz, 1H), 6.84 (dd, *J* = 8.1, 7.1 Hz, 1H), 6.77 (dd, *J* = 8.1, 7.1 Hz, 1H), 6.66 (dd, *J* = 7.1, 1.2 Hz, 1H), 0.63 (s, 3H), 0.06 (s, 3H), -0.42 (s, 9H). **^{13}C NMR (101 MHz, CDCl$_3$)** δ 157.8, 157.5, 152.8, 149.0, 144.7, 144.4, 141.4, 137.4, 136.5, 133.0, 132.9, 131.8, 131.6, 129.7, 128.2, 127.9, 127.8, 127.6, 126.2, 125.0, 125.0, 124.9, 124.7, 124.6, 124.4, 124.3, 123.6, 122.9, 121.1, 1.1, 1.1, 0.9. **HRMS (ESI)** m/z Calcd. for $C_{36}H_{36}NSi_2$ $[M+H]^+$ 538.2381, Found 538.2382.

(Z)-8-((1,2-di(naphthalen-1-yl)-2-(trimethylsilyl)vinyl)dimethylsilyl)quinoline (3ap)

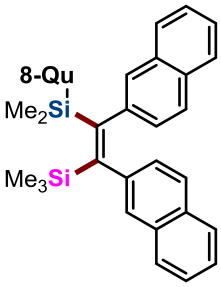

Following the general procedure, in the nitrogen-filled glovebox, to an oven-dried 8-mL sealed tube equipped with a Teflon-coated magnetic stir bar were added Ni(COD)$_2$ (5.6 mg, 0.02 mmol, 10.0 mol%), SIPr (9.5 mg, 0.024 mmol, 12.0 mol%), toluene (2 mL), and the reaction mixture was stirred for 30 min, then disilane reagent **1a** (52 mg, 0.2 mmol, 1.0 equiv), 1,2-di(naphthalen-2-yl)ethyne **2p** (111.2 mg, 0.4 mmol, 2.0 equiv) were added. The vial was sealed with a screw-top septum cap, removed from the glovebox and placed in a heating block that was pre-heated to 100 ℃ with vigorous stirring for 48 h under N_2 atmosphere. After been cooled to room temperature, the reaction mixture was filtered through a pad of celite and concentrated in vacuo. The resulting residue was purified by silica gel flash (PE/DCM = 1/1) chromatography to

give the desired product **3ao** (77 mg, 72% yield, white solid). **R$_f$** (PE/DCM = 1/1): 0.4. **^{1}H NMR (400 MHz, CDCl$_3$)** δ 8.90 (dd, *J* = 4.1, 1.8 Hz, 1H), 8.11 (dd, *J* = 6.7, 1.5 Hz, 1H), 8.00 (dd, *J* = 8.2, 1.8 Hz, 1H), 7.72 (dd, *J* = 8.1, 1.4 Hz, 1H), 7.49 – 7.44 (m, 2H), 7.37 (t, *J* = 7.7 Hz, 3H), 7.32 – 7.20 (m, 4H), 7.14 – 7.00 (m, 6H), 6.80 (d, *J* = 8.3 Hz, 1H), 0.34 (s, 3H), 0.26 (s, 3H), -0.40 (s, 9H). **^{13}C NMR (101 MHz, CDCl$_3$)** δ 152.6, 148.9, 141.2, 137.4, 136.2, 133.0, 131.03, 130.99, 129.5, 128.0, 127.63, 127.59, 127.53, 126.5, 126.4, 126.1, 125.4, 125.2, 124.6, 124.5, 121.0, 1.3. **HRMS (ESI)** m/z Calcd. for $C_{36}H_{36}NSi_2$ $[M+H]^+$ 538.2381, Found 538.2380.

(*Z*)-8-(Dimethyl(3-(trimethylsilyl)but-2-en-2-yl)silyl)quinoline (3aq)

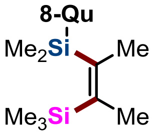

Following the general procedure, in the nitrogen-filled glovebox, to an oven-dried 8-mL sealed tube equipped with a Teflon-coated magnetic stir bar were added Ni(COD)$_2$ (4.2 mg, 0.015 mmol, 5.0 mol%), SPhos (12.3 mg, 0.03 mmol, 10.0 mol%), toluene (3 mL), and the reaction mixture was stirred for 30 min, then disilane reagent **1a** (78 mg, 0.3 mmol, 1.0 equiv), but-2-yne **2q** (32.4 mg, 0.6 mmol, 2.0 equiv) were added. The vial was sealed with a screw-top septum cap, removed from the glovebox and placed in a heating block that was pre-heated to 100 ℃ with vigorous stirring for 36 h under N_2 atmosphere. After been cooled to room temperature, the reaction mixture was filtered through a pad of celite and concentrated in vacuo. The resulting residue was purified by silica gel flash chromatography (PE) to give the desired product **3aq** (83 mg, 88% yield, colorless oil). **R$_f$** (PE): 0.6. **^{1}H NMR (400 MHz, CDCl$_3$)** δ 8.89 (dd, *J* = 4.2, 1.9 Hz, 1H), 8.11 (dd, *J* = 8.2, 1.9 Hz, 1H), 7.89 (dd, *J* = 6.8, 1.6 Hz, 1H), 7.80 (dd, *J* = 8.1, 1.5 Hz, 1H), 7.49 (dd, *J* = 8.1, 6.7 Hz, 1H), 7.34 (dd, *J* = 8.2, 4.1 Hz, 1H), 1.96 (d, *J* = 1.2 Hz, 3H), 1.93 (d, *J* = 1.2 Hz, 3H), 0.65 (s, 6H), 0.02 (s, 9H). **^{13}C NMR (101 MHz, CDCl$_3$)** δ 152.6, 149.2, 149.0, 147.8, 141.8, 137.2, 136.1, 129.1, 127.9, 126.0, 120.7, 21.2, 20.7, 1.9, 1.4. **HRMS (ESI)** m/z Calcd. for $C_{18}H_{28}NSi_2$ $[M+H]^+$ 314.1755, Found 314.1751.

(*Z*)-8-(Dimethyl(4-(trimethylsilyl)hex-3-en-3-yl)silyl)quinoline (3ar)

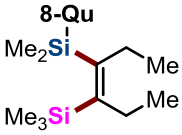

Following the general procedure, in the nitrogen-filled glovebox, to an oven-dried 8-mL sealed tube equipped with a Teflon-coated magnetic

stir bar were added Ni(COD)$_2$ (2.8 mg, 0.01 mmol, 5.0 mol%), SPhos (8.2 mg, 0.02 mmol, 10.0 mol%), toluene (2 mL), and the reaction mixture was stirred for 30 min, then disilane reagent **1a** (52 mg, 0.2 mmol, 1.0 equiv), hex-3-yne **2r** (33 mg, 0.4 mmol, 2.0 equiv) were added. The vial was sealed with a screw-top septum cap, removed from the glovebox and placed in a heating block that was pre-heated to 100 ℃ with vigorous stirring for 36 h under N$_2$ atmosphere. After been cooled to room temperature, the reaction mixture was filtered through a pad of celite and concentrated in vacuo. The resulting residue was purified by silica gel flash chromatography (PE) to give the desired product **3ar** (64 mg, 94% yield, white solid). **R*f*** (PE): 0.6. **^{1}H NMR (400 MHz, CDCl$_3$)** δ 8.89 (dd, *J* = 4.2, 1.8 Hz, 1H), 8.10 (dd, *J* = 8.2, 1.9 Hz, 1H), 7.95 (dd, *J* = 6.8, 1.5 Hz, 1H), 7.80 (dd, *J* = 8.1, 1.5 Hz, 1H), 7.49 (dd, *J* = 8.1, 6.8 Hz, 1H), 7.34 (dd, *J* = 8.2, 4.1 Hz, 1H), 2.44 (dq, *J* = 22.1, 7.5 Hz, 4H), 1.08 (t, *J* = 7.4 Hz, 3H), 1.01 (t, *J* = 7.5 Hz, 3H), 0.69 (s, 6H), -0.03 (s, 9H). **^{13}C NMR (101 MHz, CDCl$_3$)** δ 154.6, 153.2, 152.6, 148.8, 142.4, 137.6, 136.1, 129.0, 127.8, 125.9, 120.6, 26.7, 26.2, 15.7, 15.6, 2.2, 2.1. **HRMS (ESI)** m/z Calcd. for $C_{20}H_{32}NSi_2$ $[M+H]^+$ 342.2068, Found 342.2069.

(*Z*)-8-(dimethyl(5-(trimethylsilyl)oct-4-en-4-yl)silyl)quinoline (3as)

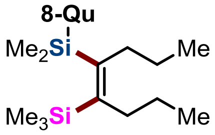

Following the general procedure, in the nitrogen-filled glovebox, to an oven-dried 8-mL sealed tube equipped with a Teflon-coated magnetic stir bar were added Ni(COD)$_2$ (2.8 mg, 0.01 mmol, 5.0 mol%), SPhos (8.2 mg, 0.02 mmol, 10.0 mol%), toluene (2 mL), and the reaction mixture was stirred for 30 min, then disilane reagent **1a** (52 mg, 0.2 mmol, 1.0 equiv), oct-4-yne **2s** (44 mg, 0.4 mmol, 2.0 equiv) were added. The vial was sealed with a screw-top septum cap, removed from the glovebox and placed in a heating block that was pre-heated to 100 ℃ with vigorous stirring for 36 h under N$_2$ atmosphere. After been cooled to room temperature, the reaction mixture was filtered through a pad of celite and concentrated in vacuo. The resulting residue was purified by silica gel flash chromatography (PE) to give the desired product **3as** (72 mg, 97% yield, white solid). **R*f*** (PE): 0.6. **^{1}H NMR (400 MHz, CDCl$_3$)** δ 8.91 (dd, *J* = 4.2, 1.9 Hz, 1H), 8.11 (dd, *J* = 8.2, 1.9 Hz, 1H), 7.98 (dd, *J* = 6.8, 1.5 Hz, 1H), 7.81 (dd, *J* = 8.0, 1.5 Hz, 1H), 7.51

(dd, J = 8.1, 6.8 Hz, 1H), 7.35 (dd, J = 8.2, 4.1 Hz, 1H), 2.47 – 2.31 (m, 4H), 1.54 – 1.36 (m, 4H), 1.04 (t, J = 7.3 Hz, 3H), 0.92 (t, J = 7.3 Hz, 3H), 0.71 (s, 6H), -0.00 (s, 9H). **^{13}C NMR (101 MHz, $CDCl_3$)** δ 153.4, 152.7, 152.1, 148.8, 142.4, 137.5, 136.0, 129.0, 127.8, 125.9, 120.6, 36.6, 36.3, 24.5, 24.3, 14.8, 14.8, 2.2, 2.1. **HRMS (ESI)** m/z Calcd. for $C_{22}H_{36}NSi_2$ $[M+H]^+$ 370.2381, Found 370.2380.

(*Z*)-8-(Dimethyl(6-(trimethylsilyl)dec-5-en-5-yl)silyl)quinoline (3at)

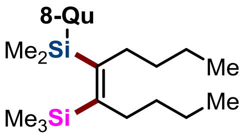

Following the general procedure, in the nitrogen-filled glovebox, to an oven-dried 8-mL sealed tube equipped with a Teflon-coated magnetic stir bar were added Ni(COD)$_2$ (2.8 mg, 0.01 mmol, 5.0 mol%), SPhos (8.2 mg, 0.02 mmol, 10.0 mol%), toluene (2 mL), and the reaction mixture was stirred for 30 min, then disilane reagent **1a** (52 mg, 0.2 mmol, 1.0 equiv), dec-5-yne **2t** (55.2 mg, 0.4 mmol, 2.0 equiv) were added. The vial was sealed with a screw-top septum cap, removed from the glovebox and placed in a heating block that was pre-heated to 100 ℃ with vigorous stirring for 36 h under N_2 atmosphere. After been cooled to room temperature, the reaction mixture was filtered through a pad of celite and concentrated in vacuo. The resulting residue was purified by silica gel flash chromatography (PE) to give the desired product **3at** (76 mg, 96% yield, white solid). **R_f** (PE): 0.6. **^{1}H NMR (400 MHz, $CDCl_3$)** δ 8.89 (dd, J = 4.2, 1.9 Hz, 1H), 8.10 (dd, J = 8.2, 1.9 Hz, 1H), 7.94 (dd, J = 6.8, 1.5 Hz, 1H), 7.80 (dd, J = 8.1, 1.5 Hz, 1H), 7.49 (dd, J = 8.1, 6.8 Hz, 1H), 7.34 (dd, J = 8.2, 4.2 Hz, 1H), 2.48 – 2.21 (m, 4H), 1.41 (d, J = 4.5 Hz, 4H), 1.30 (dt, J = 9.9, 4.9 Hz, 4H), 1.05 – 0.94 (m, 3H), 0.86 (t, J = 6.9 Hz, 3H), 0.67 (s, 6H), -0.04 (s, 9H). **^{13}C NMR (101 MHz, $CDCl_3$)** δ 153.3, 152.6, 152.0, 148.8, 142.4, 137.6, 136.1, 129.0, 127.8, 125.9, 120.6, 34.0, 33.6, 33.3, 33.1, 23.5, 23.4, 14.2, 14.1, 2.3, 2.2. **HRMS (ESI)** m/z Calcd. for $C_{24}H_{40}NSi_2$ $[M+H]^+$ 398.2694, Found 398.2694.

(*Z*)-8-((2-(Dimethyl(phenyl)silyl)-1,2-diphenylvinyl)dimethylsilyl)quinoline (3ba)

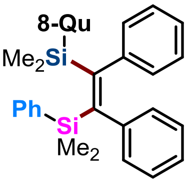

Following the general procedure, in the nitrogen-filled glovebox, to an oven-dried 8-mL sealed tube equipped with a Teflon-coated magnetic stir bar were added Ni(COD)$_2$ (5.6 mg, 0.02 mmol, 10.0 mol%), SIPr

(9.5 mg, 0.024 mmol, 12.0 mol%), toluene (2 mL), and the reaction mixture was stirred for 30 min, then disilane reagent **1b** (64.2 mg, 0.2 mmol, 1.0 equiv), diphenylethyne **2a** (71.2 mg, 0.4 mmol, 2.0 equiv) were added. The vial was sealed with a screw-top septum cap, removed from the glovebox and placed in a heating block that was pre-heated to 100 ℃ with vigorous stirring for 48 h under N_2 atmosphere. After been cooled to room temperature, the reaction mixture was filtered through a pad of celite and concentrated in vacuo. The resulting residue was purified by silica gel flash chromatography (PE/DCM = 10/1) to give the desired product **3ba** (80 mg, 80% yield, white solid). **R_f** (PE/DCM = 10/1): 0.4. **^{1}H NMR (400 MHz, CDCl$_3$)** δ 8.95 (dd, *J* = 4.0, 1.9 Hz, 1H), 8.06 (dd, *J* = 8.3, 1.9 Hz, 1H), 7.85 – 7.71 (m, 2H), 7.43 (t, *J* = 7.4 Hz, 1H), 7.35 (d, *J* = 6.8 Hz, 3H), 7.26 – 7.17 (m, 3H), 6.86 (dt, *J* = 15.3, 7.4 Hz, 4H), 6.78 – 6.71 (m, 4H), 6.64 (d, *J* = 7.5 Hz, 2H), 0.23 (s, 6H), -0.08 (s, 6H). **^{13}C NMR (101 MHz, CDCl$_3$)** δ 160.3, 155.1, 152.4, 148.7, 146.8, 146.6, 141.2, 139.8, 137.0, 136.1, 134.5, 129.3, 128.7, 128.6, 128.3, 127.8, 127.4, 126.9, 126.7, 126.0, 124.2, 124.2, 120.9, 1.1, 0.5. **HRMS (ESI)** m/z Calcd. for $C_{33}H_{34}NSi_2$ $[M+H]^+$ 500.2224, Found 500.2227.

(*Z*)-1,2-Bis(dimethyl(quinolin-8-yl)silyl)-1,2-diphenylethene (3ca)

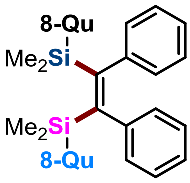

Following the general procedure, in the nitrogen-filled glovebox, to an oven-dried 8-mL sealed tube equipped with a Teflon-coated magnetic stir bar were added Ni(COD)$_2$ (5.6 mg, 0.02 mmol, 10.0 mol%), SIPr (9.5 mg, 0.024 mmol, 12.0 mol%), toluene (2 mL), and the reaction mixture was stirred for 30 min, then disilane reagent **1c** (76.4 mg, 0.2 mmol, 1.0 equiv), diphenylethyne **2a** (71.2 mg, 0.4 mmol, 2.0 equiv) were added. The vial was sealed with a screw-top septum cap, removed from the glovebox and placed in a heating block that was pre-heated to 100 ℃ with vigorous stirring for 48 h under N_2 atmosphere. After been cooled to room temperature, the reaction mixture was filtered through a pad of celite and concentrated in vacuo. The resulting residue was purified by silica gel flash chromatography (PE/EA = 4/1) to give the desired product **3ca** (61 mg, 55% yield, yellow solid). **R_f** (PE/EA = 2/1): 0.4. **^{1}H NMR (400 MHz, CDCl$_3$)** δ 9.01 (dd, *J* = 4.1, 1.8 Hz, 2H), 8.10 (dd, *J* = 8.2, 1.9 Hz, 2H), 7.84 (dd, *J* = 6.7, 1.5 Hz, 2H), 7.78 (dd, *J*

= 8.1, 1.5 Hz, 2H), 7.48 (dd, *J* = 8.1, 6.7 Hz, 2H), 7.39 (dd, *J* = 8.2, 4.1 Hz, 2H), 6.92 – 6.85 (m, 8H), 6.81 – 6.76 (m, 2H), 0.10 (s, 12H). **^{13}C NMR (101 MHz, $CDCl_3$)** δ 157.0, 152.4, 148.6, 147.3, 141.3, 137.3, 136.0, 129.1, 128.5, 127.6, 126.5, 125.9, 123.9, 120.7, 0.7. **HRMS (ESI)** m/z Calcd. for $C_{36}H_{35}N_2Si_2$ $[M+H]^+$ 551.2333, Found 551.2332.

(*Z*)-8-((2-(Isopropoxydimethylsilyl)-1,2-diphenylvinyl)dimethylsilyl)quinoline (3da)

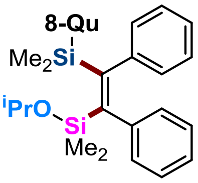

Following the general procedure, in the nitrogen-filled glovebox, to an oven-dried 8-mL sealed tube equipped with a Teflon-coated magnetic stir bar were added $Ni(COD)_2$ (5.6 mg, 0.02 mmol, 10.0 mol%), SIPr (9.5 mg, 0.024 mmol, 12.0 mol%), toluene (2 mL), and the reaction mixture was stirred for 30 min, then disilane reagent **1d** (61 mg, 0.2 mmol, 1.0 equiv), diphenylethyne **2a** (71.2 mg, 0.4 mmol, 2.0 equiv) were added. The vial was sealed with a screw-top septum cap, removed from the glovebox and placed in a heating block that was pre-heated to 100 ℃ with vigorous stirring for 48 h under N_2 atmosphere. After been cooled to room temperature, the reaction mixture was filtered through a pad of celite and concentrated in vacuo. The resulting residue was purified by silica gel flash chromatography (PE/DCM = 5/1) to give the desired product **3da** (74 mg, 77% yield, white solid). **R_f** (PE/DCM = 2/1): 0.4. **^{1}H NMR (400 MHz, $CDCl_3$)** δ 8.95 (dd, *J* = 4.0, 1.6 Hz, 1H), 8.22 (dd, *J* = 6.8, 1.5 Hz, 1H), 8.12 (dd, *J* = 8.2, 1.9 Hz, 1H), 7.81 (dd, *J* = 8.1, 1.6 Hz, 1H), 7.55 (dd, *J* = 8.1, 6.8 Hz, 1H), 7.37 (dd, *J* = 8.2, 4.1 Hz, 1H), 7.02 (t, *J* = 7.6 Hz, 2H), 6.91 (q, *J* = 7.9 Hz, 3H), 6.84 – 6.76 (m, 5H), 3.77 (hept, *J* = 6.0 Hz, 1H), 0.74 (d, *J* = 6.1 Hz, 6H), 0.51 (s, 6H), -0.13 (s, 6H). **^{13}C NMR (101 MHz, $CDCl_3$)** δ 159.0, 156.8, 152.6, 148.6, 146.8, 145.6, 142.1, 137.6, 136.0, 131.6, 128.7, 128.2, 127.8, 127.1, 126.7, 126.0, 124.5, 124.1, 120.6, 65.5, 25.3, 1.7, 0.1. **HRMS (ESI)** m/z Calcd. for $C_{30}H_{36}NOSi_2$ $[M+H]^+$ 482.2330, Found. 482.2333.

(*Z*)-8-((5-(Isopropoxydimethylsilyl)oct-4-en-4-yl)dimethylsilyl)quinoline (3db)

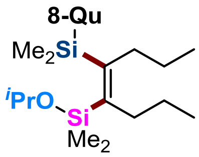

Following the general procedure, in the nitrogen-filled glovebox, to an oven-dried 8-mL sealed tube equipped with a Teflon-coated magnetic stir bar were added Ni(COD)$_2$ (2.8 mg, 0.01 mmol, 5.0 mol%), SPhos (8.2 mg, 0.02 mmol, 10.0 mol%), toluene (2 mL), and the reaction mixture was stirred for 30 min, then disilane reagent **1d** (61 mg, 0.2 mmol, 1.0 equiv), oct-4-yne **2r** (44 mg, 0.4 mmol, 2.0 equiv) were added. The vial was sealed with a screw-top septum cap, removed from the glovebox and placed in a heating block that was pre-heated to 100 ℃ with vigorous stirring for 36 h under N_2 atmosphere. After been cooled to room temperature, the reaction mixture was filtered through a pad of celite and concentrated in vacuo. The resulting residue was purified by silica gel flash chromatography (PE) to give the desired product **3db** (68 mg, 82% yield, colorless oil). **R*f*** (PE): 0.6. **^{1}H NMR (400 MHz, CDCl$_3$)** δ 8.87 (dd, *J* = 4.1, 1.9 Hz, 1H), 8.08 (dd, *J* = 8.2, 1.9 Hz, 1H), 7.91 (dd, *J* = 6.8, 1.6 Hz, 1H), 7.76 (dd, *J* = 8.1, 1.5 Hz, 1H), 7.45 (dd, *J* = 8.1, 6.8 Hz, 1H), 7.32 (dd, *J* = 8.2, 4.2 Hz, 1H), 3.75 (hept, *J* = 6.1 Hz, 1H), 2.35 – 2.22 (m, 4H), 1.49 – 1.26 (m, 4H), 0.98 (t, *J* = 7.3 Hz, 3H), 0.83 (t, *J* = 7.3 Hz, 3H), 0.70 (s, 3H), 0.69 (s, 3H), 0.66 (s, 6H), 0.08 (s, 6H). **^{13}C NMR (101 MHz, CDCl$_3$)** δ 153.8, 152.6, 151.6, 148.6, 143.3, 137.5, 136.0, 128.4, 127.7, 125.9, 120.4, 64.8, 36.4, 34.8, 25.2, 24.4, 24.2, 15.0, 14.7, 2.2, 0.7. **HRMS (ESI)** m/z Calcd. for $C_{24}H_{40}NOSi_2$ [M+H]$^+$ 414.2643, Found 414.2645.

Bissilylation of Unsymmetric Internal Alkynes

Supplementary Table 2. Optimization of reaction conditions[a]

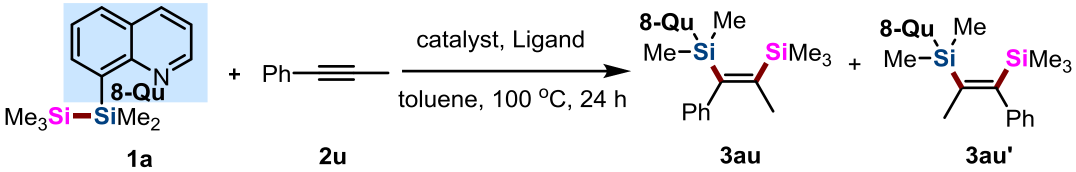

Entry	[M] Catalyst	Ligand	Yield[%][b]	3au:3au'[c]
1	Ni(COD)$_2$ (10 mol%)	SIPr (12 mol%)	NR	ND
2	Ni(COD)$_2$ (10 mol%)	PPh$_3$ (20 mol%)	33	77:23
3	Ni(COD)$_2$ (10 mol%)	PAd$_2$^{n}Bu (20 mol%)	trace	N.D.
4	Ni(COD)$_2$ (10 mol%)	PMe$_3$ (20 mol%)	trace	N.D.
5	Ni(COD)$_2$ (10 mol%)	P(2-OMeC$_6$H$_4$)$_3$ (20 mol%)	< 5	76:24
6	Ni(COD)$_2$ (10 mol%)	P(3-OMeC$_6$H$_4$)$_3$ (20 mol%)	24	78:22
7	Ni(COD)$_2$ (10 mol%)	**L1** (20 mol%)	18	74:26
8	Ni(COD)$_2$ (10 mol%)	**L2** (20 mol%)	N.R.	N.D.
9	Ni(COD)$_2$ (10 mol%)	XPhos (20 mol%)	69	79:21
10	Ni(COD)$_2$ (10 mol%)	SPhos (20 mol%)	59	77:23
11	Ni(COD)$_2$ (10 mol%)	RockPhos (20 mol%)	77	79:21
12	Ni(COD)$_2$ (10 mol%)	RuPhos (20 mol%)	41	78:22
13	Ni(COD)$_2$ (10 mol%)	tBuXPhos (20 mol%)	75	80:20
**14**[d]	**Ni(COD)$_2$ (10 mol%)**	**BrettPhos (20 mol%)**	**83**	**80:20**
15	Ni(COD)$_2$ (10 mol%)	tBuBrettPhos (20 mol%)	80[c]	80:20
16	Ni(COD)$_2$ (10 mol%)	AdBrettPhos (20 mol%)	79[c]	79:21
17	Ni(COD)$_2$ (10 mol%)	JohnPhos (20 mol%)	64	79:21
18	Ni(COD)$_2$ (10 mol%)	MePhos (20 mol%)	17	76:24
19	Ni(COD)$_2$ (10 mol%)	VPhos (20 mol%)	59	79:21
20	Ni(COD)$_2$ (10 mol%)	CPhos (20 mol%)	52	77:23
21	Ni(COD)$_2$ (10 mol%)	QPhos (20 mol%)	42	76:24
22	Ni(COD)$_2$ (10 mol%)	**L3** (20 mol%)	62	80:20
23	Ni(COD)$_2$ (10 mol%)	**L4** (20 mol%)	63	79:21
24	Ni(COD)$_2$ (10 mol%)	**L5** (20 mol%)	54	74:26
25	Ni(COD)$_2$ (10 mol%)	**L6** (20 mol%)	18	75:25
26	Ni(COD)$_2$ (10 mol%)	**L7** (20 mol%)	13	73:27

27	$Ni(COD)_2$ (10 mol%)	**L8** (20 mol%)	42	76:24
28[d]	**$Ni(COD)_2$ (5 mol%)**	**BrettPhos (10 mol%)**	**80**	**80:20**
29	$Ni(PPh_3)_4$ (5 mol%)	BrettPhos (10 mol%)	9	77:23
30	$NiBr_2$·DME (5 mol%)	BrettPhos (10 mol%)	N.R.	N.D.
31	$Ni(PPh_3)_2Cl_2$ (5 mol%)	BrettPhos (10 mol%)	N.R.	N.D.
32	$NiCl_2$ (5 mol%)	BrettPhos (10 mol%)	N.R.	N.D.

[a] Reactions were carried out with catalyst, ligand, disilane **1a** (0.20 mmol) and 1-phenylpropyne **2u** (0.40 mmol, 2 eq.) in toluene for 24 h at 100 ℃ under an N_2 atmosphere. [b] Yields were determined by ^1H NMR spectroscopy of the crude mixture with an internal standard (CH_2Br_2). [c] The **3au:3au′** ratio was determined by GC analysis. [d] Yields of isolated products. N.R.: No reaction (N.R.). N.D.: not determined.

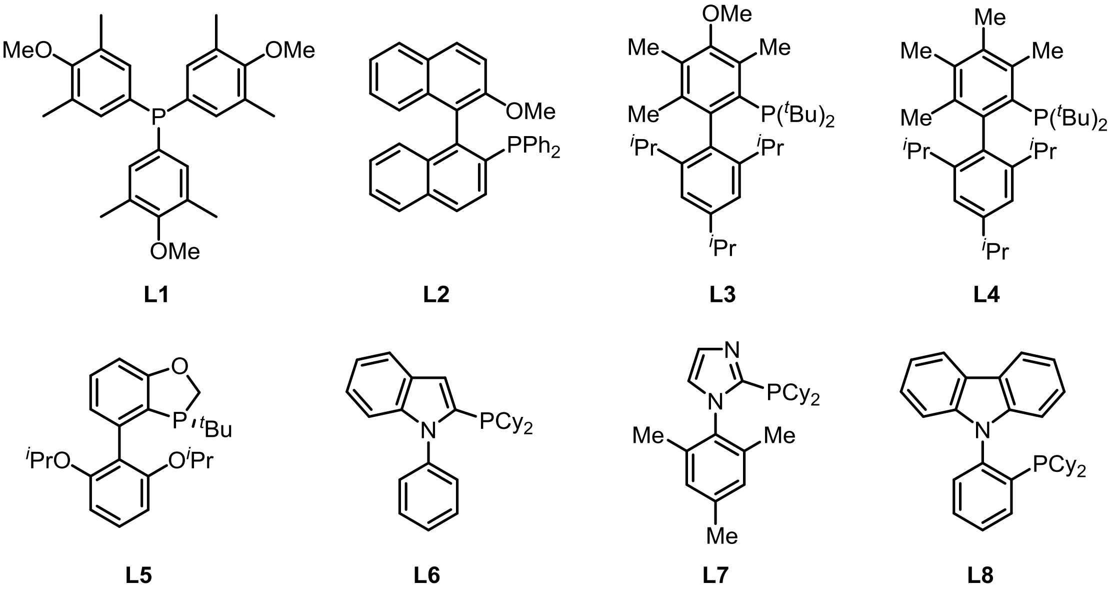

Supplementary Table 3. Optimization of reaction conditions[a]

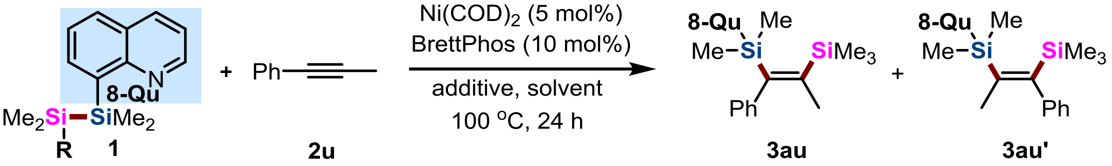

Entry	Disilane	Additive	Solvent	Yield[%][b]	3au:3au′[c]
1	**1a**	none	1,4-dioxane	77[c]	80:20
2	**1a**	none	THF	70	79:21
3	**1a**	none	DME	N.R.	N.D.
**4**[d]	**1a**	**none**	**DMF**	**82**	**83:17**
5	**1a**	none	DMA	80[c]	80:20.

6	**1a**	none	DMSO	45	81:19
7	**1a**	none	MeCN	76[c]	81:19
8	**1a**	none	hexane	70	72:28
9	**1a**	MAD (20 mol%)	DMF	67	81:19
10	**1a**	$B(C_6F_5)_3$ (20 mol%)	DMF	26	76:24
11	**1a**	TBAI (20 mol%)	DMF	51	80:20
12[d, e]	**1a**	none	DMF	74	82:18
13[d, f]	**1a**	none	DMF	70	82:18
14	**1b**	none	DMF	trace	N.D.
**15**	**1d**	**none**	**DMF**	**81**	**86:14**

[a] Reactions were carried out with $Ni(COD)_2$ (5 mol%), BrettPhos (10 mol%), additive (20 mol%), disilane **1** (0.20 mmol) and 1-phenylpropyne **2u** (0.40 mmol, 2 eq.) in solvent for 24 h at 100 ℃ under an N_2 atmosphere. [b] Yields were determined by ^{1}H NMR spectroscopy of the crude mixture with an internal standard (CH_2Br_2). [c] The **3au:3au′** ratio was determined by GC analysis. [d] Yields of isolated products. [e] The reaction was conducted at 90 ℃. [f] The reaction was conducted at 80 ℃. N.R.: No reaction (N.R.). N.D.: not determined.

General procedure: In the nitrogen-filled glovebox, to an oven-dried 8-mL sealed tube equipped with a Teflon-coated magnetic stir bar were added $Ni(COD)_2$ (2.8 mg, 0.01 mmol, 5.0 mol%), BrettPhos (10.8 mg, 0.02 mmol, 10.0 mol%), DMF (2 mL), and the reaction mixture was stirred for 15 min, then disilane reagent **1** (0.2 mmol, 1.0 equiv), unsymmetric internal alkynes (0.4 mmol, 2.0 equiv) were added. The vial was sealed with a screw-top septum cap, removed from the glovebox and placed in a heating block that was pre-heated to 100 ℃ with vigorous stirring for 24 h under N_2 atmosphere. The reaction mixture is cooled to room temperature and the regioselectivity was determined by GC analysis or ^{1}H NMR of crude materials. After removal of the solvent, the residue was purified by reversed phase C18(ODS) column (5μm, 21.2×250 mm) with MeCN to afford **3au–dä** The mobile phase flow rate was 10 mL/min, and the detection was at 254 nm.

(*Z*)-8-(dimethyl(1-phenyl-2-(trimethylsilyl)prop-1-en-1-yl)silyl)quinoline (3au) & **(*Z*)-8-(dimethyl(1-phenyl-1-(trimethylsilyl)prop-1-en-2-yl)silyl)quinoline (3au′)**

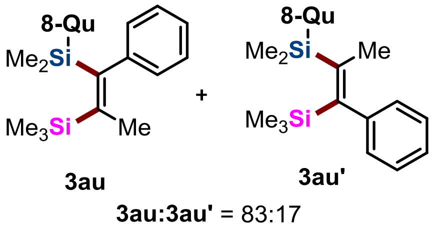

Following the general procedure, in the nitrogen-filled glovebox, to an oven-dried 8-mL sealed tube equipped with a Teflon-coated magnetic stir bar were added Ni(COD)$_2$ (2.8 mg, 0.01 mmol, 5.0 mol%), BrettPhos (10.8 mg, 0.02 mmol, 10.0 mol%), DMF (2 mL), and the reaction mixture was stirred for 15 min, then disilane reagent **1a** (52 mg, 0.2 mmol, 1.0 equiv), prop-1-yn-1-ylbenzene **2u** (46.4 mg, 0.4 mmol, 2.0 equiv) were added. The vial was sealed with a screw-top septum cap, removed from the glovebox and placed in a heating block that was pre-heated to 100 ℃ with vigorous stirring for 24 h under N$_2$ atmosphere. The reaction mixture is cooled to room temperature and the regioselectivity was determined by GC analysis (r.r. = 83:17). After removal of the solvent, the residue was purified by reversed phase C18(ODS) column (5μm, 21.2×250 mm) with MeCN to afford **3au** and **3au′** (62 mg, 82%) as colorless viscous liquid. **R*f*** (PE/DCM = 20/1): 0.4. **Major product (3au)**: **^{1}H NMR (400 MHz, CDCl$_3$)** δ 8.89 (dd, *J* = 4.1, 1.8 Hz, 1H), 8.10 (dd, *J* = 3.6, 1.7 Hz, 1H), 8.08 (t, *J* = 1.9 Hz, 1H), 7.80 (dd, *J* = 8.1, 1.5 Hz, 1H), 7.50 (dd, *J* = 8.1, 6.8 Hz, 1H), 7.34 (dd, *J* = 8.2, 4.2 Hz, 1H), 7.29 – 7.22 (m, 2H), 7.14 – 7.08 (m, 1H), 7.05 – 6.98 (m, 2H), 1.63 (s, 3H), 0.33 (s, 6H), -0.15 (s, 9H). **^{13}C NMR (101 MHz, CDCl$_3$)** δ 155.7, 152.5, 150.3, 148.9, 148.1, 141.6, 137.5, 136.1, 129.3, 128.0, 127.9, 127.6, 125.9, 124.7, 120.8, 22.8, 1.9, 0.9. **HRMS (ESI)** m/z Calcd. for $C_{23}H_{30}NSi_2$ $[M+H]^+$ 376.1911, Found 376.1911. **Minor product (3au′)**: **^{1}H NMR (400 MHz, CDCl$_3$)** δ 8.91 (dd, *J* = 4.2, 1.9 Hz, 1H), 8.13 (dd, *J* = 8.2, 1.9 Hz, 1H), 7.98 (dd, *J* = 6.8, 1.5 Hz, 1H), 7.83 (dd, *J* = 8.1, 1.5 Hz, 1H), 7.53 (dd, *J* = 8.1, 6.7 Hz, 1H), 7.38 (dd, *J* = 8.2, 4.1 Hz, 1H), 7.32 – 7.26 (m, 2H), 7.14 (ddt, *J* = 7.9, 7.0, 1.3 Hz, 1H), 6.91 – 6.82 (m, 2H), 1.66 (s, 3H), 0.68 (s, 6H), -0.19 (s, 9H). **^{13}C NMR (101 MHz, CDCl$_3$)** δ 156.7, 152.6, 149.8, 148.9, 148.0, 141.5, 136.8, 136.1, 129.2, 128.1, 127.9, 127.5, 126.1, 124.7, 120.8, 23.6, 1.6, 1.4. **HRMS (ESI)** m/z Calcd. for $C_{23}H_{30}NSi_2$ $[M+H]^+$ 376.1911, Found 376.1911.

(*Z*)-8-((2-(isopropoxydimethylsilyl)-1-phenylprop-1-en-1-yl)dimethylsilyl)quinoline (3du) and (*Z*)-8-((1-(isopropoxydimethylsilyl)-1-phenylprop-1-en-2-yl)dimethylsilyl)quinoline (**3du′**)

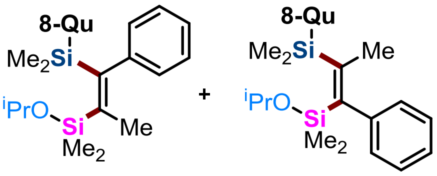

3du **3du'**

3du:3du' = 86:14

Following the general procedure, in the nitrogen-filled glovebox, to an oven-dried 8-mL sealed tube equipped with a Teflon-coated magnetic stir bar were added $Ni(COD)_2$ (2.8 mg, 0.01 mmol, 5.0 mol%), BrettPhos (10.8 mg, 0.02 mmol, 10.0 mol%), DMF (2 mL), and the reaction mixture was stirred for 15 min, then disilane reagent **1d** (61 mg, 0.2 mmol, 1.0 equiv), prop-1-yn-1-ylbenzene **2u** (46.4 mg, 0.4 mmol, 2.0 equiv) were added. The vial was sealed with a screw-top septum cap, removed from the glovebox and placed in a heating block that was pre-heated to 100 ℃ with vigorous stirring for 24 h under N_2 atmosphere. The reaction mixture is cooled to room temperature and the regioselectivity was determined by GC analysis (r.r. = 86:14). After removal of the solvent, the residue was purified by reversed phase C18(ODS) column (5μm, 21.2×250 mm) with MeCN to afford **3du** and **3du′** (68 mg, 81%) as colorless viscous liquid. **R*f*** (PE/DCM = 10/1): 0.4. **Major product** (**3du**): **^{1}H NMR (400 MHz, $CDCl_3$)** δ 8.83 (dd, *J* = 4.1, 1.8 Hz, 1H), 8.03 (ddd, *J* = 10.1, 7.5, 1.7 Hz, 2H), 7.71 (dd, *J* = 8.1, 1.5 Hz, 1H), 7.43 (dd, *J* = 8.1, 6.8 Hz, 1H), 7.26 (dd, *J* = 8.2, 4.1 Hz, 1H), 7.18 (t, *J* = 7.6 Hz, 2H), 7.06 – 6.99 (m, 1H), 6.97 – 6.90 (m, 2H), 3.65 (hept, *J* = 6.1 Hz, 1H), 1.56 (s, 3H), 0.61 (d, *J* = 6.1 Hz, 6H), 0.34 (s, 6H), -0.00 (s, 6H). **^{13}C NMR (101 MHz, $CDCl_3$)** δ 157.1, 152.5, 148.8, 148.7, 147.9, 142.4, 137.5, 136.0, 128.6, 127.9, 127.8, 127.3, 125.9, 124.6, 120.5, 65.1, 25.2, 21.3, 2.1, -0.1. **HRMS (ESI)** m/z Calcd. for $C_{25}H_{34}NOSi_2$ $[M+H]^+$ 420.2173, Found 420.2170. **Minor product** (**3du′**): **^{1}H NMR (400 MHz, $CDCl_3$)** δ 8.89 (dd, *J* = 4.2, 1.8 Hz, 1H), 8.11 (dd, *J* = 8.2, 1.9 Hz, 1H), 7.98 (dd, *J* = 6.7, 1.5 Hz, 1H), 7.80 (dd, *J* = 8.1, 1.5 Hz, 1H), 7.51 (dd, *J* = 8.1, 6.8 Hz, 1H), 7.35 (dd, *J* = 8.3, 4.2 Hz, 1H), 7.29 (dd, *J* = 8.2, 6.9 Hz, 2H), 7.18 – 7.11 (m, 1H), 6.96 – 6.90 (m, 2H), 3.88 (hept, *J* = 6.1 Hz, 1H), 1.55 (s, 3H), 0.87 (d, *J* = 6.1 Hz, 6H), 0.72 (s, 6H), -0.06 (s, 6H). **^{13}C NMR (101 MHz, $CDCl_3$)** δ 155.0,

152.6, 151.9, 148.8, 147.0, 142.4, 136.8, 136.0, 128.7, 128.1, 128.1, 127.8, 126.1, 124.9, 120.6, 65.4, 25.5, 23.4, 1.2, 0.7. **HRMS (ESI)** m/z Calcd. for $C_{25}H_{34}NOSi_2$ $[M+H]^+$ 420.2173, Found 420.2169.

(*Z*)-8-((2-(isopropoxydimethylsilyl)-1-(4-methoxyphenyl)prop-1-en-1-yl)dimethylsilyl)quinoline (3dv) and **(*Z*)-8-((1-(isopropoxydimethylsilyl)-1-(4-methoxyphenyl)prop-1-en-2-yl)dimethylsilyl)quinoline** (**3dv′**)

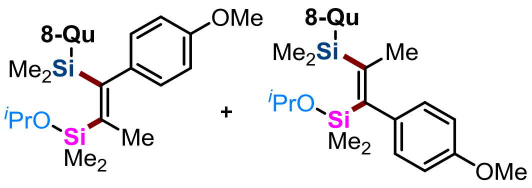

Following the general procedure, in the nitrogen-filled glovebox, to an oven-dried 8-mL sealed tube equipped with a Teflon-coated magnetic stir bar were added $Ni(COD)_2$ (2.8 mg, 0.01 mmol, 5.0 mol%), BrettPhos (10.8 mg, 0.02 mmol, 10.0 mol%), DMF (2 mL), and the reaction mixture was stirred for 15 min, then disilane reagent **1d** (61 mg, 0.2 mmol, 1.0 equiv), 1-methoxy-4-(prop-1-yn-1-yl)benzene **2v** (58.4 mg, 0.4 mmol, 2.0 equiv) were added. The vial was sealed with a screw-top septum cap, removed from the glovebox and placed in a heating block that was pre-heated to 100 ℃ with vigorous stirring for 24 h under N_2 atmosphere. The reaction mixture is cooled to room temperature and the regioselectivity was determined by GC analysis (r.r. = 89:11). After removal of the solvent, the residue was purified by reversed phase C18(ODS) column (5μm, 21.2×250 mm) with MeCN to afford **3dv** and **3dv′** (56 mg, 62%) as colorless viscous liquid. **R*f*** (PE/DCM = 5/1): 0.4. **Major product** (**3dv**): **^{1}H NMR (400 MHz, $CDCl_3$)** δ 8.90 (dd, *J* = 4.2, 1.8 Hz, 1H), 8.10 (t, *J* = 2.0 Hz, 1H), 8.08 (dd, *J* = 3.6, 1.7 Hz, 1H), 7.78 (dd, *J* = 8.1, 1.5 Hz, 1H), 7.50 (dd, *J* = 8.1, 6.7 Hz, 1H), 7.33 (dd, *J* = 8.2, 4.1 Hz, 1H), 6.95 – 6.88 (m, 2H), 6.85 – 6.78 (m, 2H), 3.78 (s, 3H), 3.72 (hept, *J* = 6.1 Hz, 1H), 1.65 (s, 3H), 0.69 (d, *J* = 6.1 Hz, 6H), 0.42 (s, 6H), 0.06 (s, 6H). **^{13}C NMR (101 MHz, $CDCl_3$)** δ 156.9, 156.7, 152.5, 149.4, 148.7, 142.5, 140.1, 137.5, 135.9, 128.6, 128.3, 127.8, 125.9, 120.5, 113.3, 65.1, 55.2, 25.2, 21.3, 2.1, -0.1. **HRMS (ESI)** m/z Calcd. for $C_{26}H_{36}NO_2Si_2$ $[M+H]^+$ 450.2279, Found 450.2277. **Minor product** (**3dv′**): **^{1}H NMR (400 MHz, $CDCl_3$)** δ 8.89 (dd, *J* = 4.3, 1.9 Hz, 1H), 8.11 (dd, *J* = 8.2, 1.9 Hz, 1H), 7.97

(dd, J = 6.8, 1.5 Hz, 1H), 7.79 (dd, J = 8.2, 1.5 Hz, 1H), 7.51 (dd, J = 8.1, 6.7 Hz, 1H), 7.35 (dd, J = 8.2, 4.2 Hz, 1H), 6.85 (s, 4H), 3.86 (hept, J = 6.1 Hz, 1H), 3.80 (s, 3H), 1.57 (s, 3H), 0.84 (d, J = 6.1 Hz, 6H), 0.71 (s, 6H), -0.06 (s, 6H). **^{13}C NMR (101 MHz, $CDCl_3$)** δ 157.3, 154.5, 152.5, 148.7, 142.5, 139.0, 136.8, 136.1, 129.1, 128.7, 127.8, 126.1, 120.6, 113.6, 65.4, 55.3, 25.4, 23.3, 1.2, 0.6. **HRMS (ESI)** m/z Calcd. for $C_{26}H_{36}NO_2Si_2$ $[M+H]^+$ 450.2279, Found 450.2278.

(*Z*)-8-((2-(isopropoxydimethylsilyl)-1-(3-methoxyphenyl)prop-1-en-1-yl)dimethylsilyl)quinoline (3dw) and **(*Z*)-8-((1-(isopropoxydimethylsilyl)-1-(3-methoxyphenyl)prop-1-en-2-yl)dimethylsilyl)quinoline** (**3dw′**)

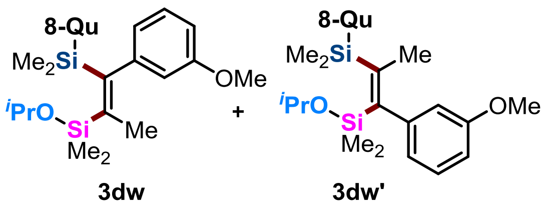

3dw:3dw' = 89:11

Following the general procedure, in the nitrogen-filled glovebox, to an oven-dried 8-mL sealed tube equipped with a Teflon-coated magnetic stir bar were added $Ni(COD)_2$ (2.8 mg, 0.01 mmol, 5.0 mol%), BrettPhos (10.8 mg, 0.02 mmol, 10.0 mol%), DMF (2 mL), and the reaction mixture was stirred for 15 min, then disilane reagent **1d** (61 mg, 0.2 mmol, 1.0 equiv), 1-methoxy-3-(prop-1-yn-1-yl)benzene **2w** (58.4 mg, 0.4 mmol, 2.0 equiv) were added. The vial was sealed with a screw-top septum cap, removed from the glovebox and placed in a heating block that was pre-heated to 100 ℃ with vigorous stirring for 24 h under N_2 atmosphere. The reaction mixture is cooled to room temperature and the regioselectivity was determined by GC analysis (r.r. = 89:11). After removal of the solvent, the residue was purified by reversed phase C18(ODS) column (5μm, 21.2×250 mm) with MeCN to afford **3dw** and **3dw′** (61 mg, 68%,) as colorless viscous liquid. **R*f*** (PE/DCM = 5/1): 0.4. **Major product** (**3dw**): **^{1}H NMR (400 MHz, $CDCl_3$)** δ 8.90 (dd, J = 4.1, 1.9 Hz, 1H), 8.12 – 8.04 (m, 2H), 7.77 (dd, J = 8.1, 1.5 Hz, 1H), 7.49 (dd, J = 8.1, 6.8 Hz, 1H), 7.33 (dd, J = 8.3, 4.1 Hz, 1H), 7.17 (t, J = 7.8 Hz, 1H), 6.65 (ddd, J = 8.2, 2.7, 1.0 Hz, 1H), 6.63 – 6.55 (m, 2H), 3.76 – 3.65 (m, 4H), 1.65 (s, 3H), 0.70 (d, J = 6.1 Hz, 6H), 0.43 (d, J = 5.0 Hz, 6H), 0.06 (s, 6H). **^{13}C NMR (101 MHz, $CDCl_3$)** δ 159.4, 156.9, 152.5, 149.4, 148.7, 142.3, 137.4, 136.0, 128.9, 128.6, 127.8, 125.9, 120.5,

119.9, 113.0, 109.9, 65.1, 55.2, 25.2, 21.3, 1.9, -0.1. **HRMS (ESI)** m/z Calcd. for $C_{26}H_{36}NO_2Si_2$ [M+H]$^+$ 450.2279, Found 450.2274. **Minor product** (**3dw′**): **^{1}H NMR (400 MHz, CDCl$_3$)** δ 8.89 (dd, *J* = 4.2, 1.9 Hz, 1H), 8.11 (dd, *J* = 8.2, 1.9 Hz, 1H), 7.97 (dd, *J* = 6.8, 1.5 Hz, 1H), 7.80 (dd, *J* = 8.1, 1.5 Hz, 1H), 7.51 (dd, *J* = 8.1, 6.8 Hz, 1H), 7.35 (dd, *J* = 8.2, 4.1 Hz, 1H), 7.21 (t, *J* = 7.8 Hz, 1H), 6.70 (ddd, *J* = 8.2, 2.6, 0.9 Hz, 1H), 6.54 (dt, *J* = 7.5, 1.2 Hz, 1H), 6.49 (dd, *J* = 2.6, 1.4 Hz, 1H), 3.89 (hept, *J* = 6.1 Hz, 1H), 3.80 (s, 3H), 1.57 (s, 3H), 0.88 (d, *J* = 6.1 Hz, 6H), 0.71 (s, 6H), -0.05 (s, 6H). **^{13}C NMR (101 MHz, CDCl$_3$)** δ 157.3, 154.5, 152.5, 148.7, 142.5, 139.0, 136.8, 136.1, 129.1, 128.7, 127.8, 126.1, 120.6, 113.6, 65.4, 55.3, 25.4, 23.3, 1.2, 0.6. **HRMS (ESI)** m/z Calcd. for $C_{26}H_{36}NO_2Si_2$ [M+H]$^+$ 450.2279, Found 450.2274.

(*Z*)-8-((2-(isopropoxydimethylsilyl)-1-(*o*-tolyl)prop-1-en-1-yl)dimethylsilyl)quinoline (3dx) and **(*Z*)-8-((1-(isopropoxydimethylsilyl)-1-(o-tolyl)prop-1-en-2-yl)dimethylsilyl)quinoline** (**3dx′**)

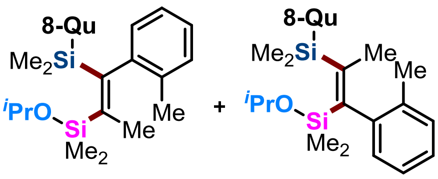

3dx **3dx'**

3dx:3dx' = 89:11

Following the general procedure, in the nitrogen-filled glovebox, to an oven-dried 8-mL sealed tube equipped with a Teflon-coated magnetic stir bar were added Ni(COD)$_2$ (2.8 mg, 0.01 mmol, 5.0 mol%), BrettPhos (10.8 mg, 0.02 mmol, 10.0 mol%), DMF (2 mL), and the reaction mixture was stirred for 15 min, then disilane reagent **1d** (61 mg, 0.2 mmol, 1.0 equiv), 1-methyl-2-(prop-1-yn-1-yl)benzene **2x** (52 mg, 0.4 mmol, 2.0 equiv) were added. The vial was sealed with a screw-top septum cap, removed from the glovebox and placed in a heating block that was pre-heated to 100 ℃ with vigorous stirring for 24 h under N_2 atmosphere. The reaction mixture is cooled to room temperature and the regioselectivity was determined by GC analysis (r.r. = 89:11). After removal of the solvent, the residue was purified by reversed phase C18(ODS) column (5μm, 21.2×250 mm) with MeCN to afford **3dx** and **3dx′** (62 mg, 72%) as colorless viscous liquid. **R*f*** (PE/DCM = 10/1): 0.4. **Major product** (**3dx**): **^{1}H NMR (400 MHz, CDCl$_3$)** δ 8.89 (dd, *J* = 4.2, 1.8 Hz, 1H), 8.24 (dd, *J* = 6.8, 1.5 Hz, 1H), 8.10 (dd, *J* = 8.2, 1.9 Hz, 1H), 7.80 (dd, *J* = 8.1, 1.5 Hz, 1H), 7.53 (dd, *J* = 8.1, 6.8 Hz, 1H),

7.33 (dd, J = 8.2, 4.1 Hz, 1H), 7.20 – 7.15 (m, 1H), 7.14 – 7.03 (m, 2H), 6.97 (dd, J = 7.3, 1.6 Hz, 1H), 3.69 (hept, J = 6.1 Hz, 1H), 2.26 (s, 3H), 1.61 (s, 3H), 0.69 (d, J = 6.1 Hz, 3H), 0.66 (s, 3H), 0.49 (d, J = 6.1 Hz, 3H), 0.16 (s, 3H), 0.12 (d, J = 6.1 Hz, 6H). **^{13}C NMR (101 MHz, $CDCl_3$)** δ 155.9, 152.6, 149.5, 148.7, 147.1, 142.3, 137.8, 136.0, 133.9, 129.7, 128.6, 127.8, 126.9, 125.9, 125.7, 125.0, 120.4, 65.0, 25.1, 24.9, 20.8, 20.2, 2.7, 1.6, -0.1, -0.4. **HRMS (ESI)** m/z Calcd. for $C_{26}H_{36}NOSi_2$ $[M+H]^+$ 434.2330, Found 434.2328. **Minor product** (**3dx′**): **^{1}H NMR (400 MHz, $CDCl_3$)** δ 8.88 (dd, J = 4.4, 1.7 Hz, 1H), 8.11 (dd, J = 8.2, 1.8 Hz, 1H), 7.98 (dd, J = 6.8, 1.5 Hz, 1H), 7.80 (dd, J = 8.1, 1.5 Hz, 1H), 7.51 (dd, J = 8.1, 6.7 Hz, 1H), 7.38 – 7.32 (m, 1H), 7.12 (tt, J = 7.2, 3.7 Hz, 2H), 7.05 (td, J = 7.3, 1.6 Hz, 1H), 6.82 (dd, J = 7.3, 1.6 Hz, 1H), 3.94 (hept, J = 6.0 Hz, 1H), 2.16 (s, 3H), 1.44 (s, 3H), 0.98 (d, J = 6.1 Hz, 3H), 0.93 (d, J = 6.1 Hz, 3H), 0.74 (s, 3H), 0.69 (s, 3H), -0.04 (d, J = 12.9 Hz, 6H). **^{13}C NMR (101 MHz, $CDCl_3$)** δ 151.2, 147.8, 145.1, 141.4, 135.7, 133.7, 128.8, 127.8, 126.8, 126.6, 125.1, 124.6, 124.3, 119.7, 64.4, 24.7, 24.6, 21.8, 19.0, 0.00, -0.03, -0.2, -0.3. **HRMS (ESI)** m/z Calcd. for $C_{26}H_{36}NOSi_2$ $[M+H]^+$ 434.2330, Found 434.2329.

(*Z*)-8-((1-(2-fluorophenyl)-2-(isopropoxydimethylsilyl)prop-1-en-1-yl)dimethylsilyl)quinoline (**3dy**) and **(*Z*)-8-((1-(2-fluorophenyl)-1-(isopropoxydimethylsilyl)prop-1-en-2-yl)dimethylsilyl)quinoline** (**3dy′**)

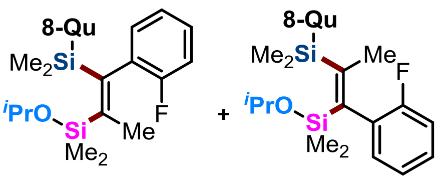

3dy **3dy'**

3dy:3dy' = 90:10

Following the general procedure, in the nitrogen-filled glovebox, to an oven-dried 8-mL sealed tube equipped with a Teflon-coated magnetic stir bar were added Ni(COD)$_2$ (2.8 mg, 0.01 mmol, 5.0 mol%), BrettPhos (10.8 mg, 0.02 mmol, 10.0 mol%), DMF (2 mL), and the reaction mixture was stirred for 15 min, then disilane reagent **1d** (61 mg, 0.2 mmol, 1.0 equiv), 1-fluoro-2-(prop-1-yn-1-yl)benzene **2y** (54 mg, 0.4 mmol, 2.0 equiv) were added. The vial was sealed with a screw-top septum cap, removed from the glovebox and placed in a heating block that was pre-heated to 100 ℃ with vigorous stirring for 24 h under N_2 atmosphere. The reaction mixture is cooled to room temperature and the regioselectivity was determined by GC analysis (r.r. = 90:10).

After removal of the solvent, the residue was purified by reversed phase C18(ODS) column (5μm, 21.2×250 mm) with MeCN to afford **3dy** and **3dy′** (59 mg, 68%) as colorless viscous liquid. **R*f*** (PE/DCM = 10/1): 0.4. **Major product** (**3dy**): **^{1}H NMR (400 MHz, $CDCl_3$)** δ 8.89 (dd, *J* = 4.2, 1.9 Hz, 1H), 8.17 (dd, *J* = 6.8, 1.5 Hz, 1H), 8.08 (dd, *J* = 8.3, 1.9 Hz, 1H), 7.78 (dd, *J* = 8.1, 1.5 Hz, 1H), 7.50 (dd, *J* = 8.1, 6.8 Hz, 1H), 7.32 (dd, *J* = 8.2, 4.1 Hz, 1H), 7.14 – 7.06 (m, 1H), 7.06 – 7.01 (m, 2H), 7.01 – 6.95 (m, 1H), 3.70 (hept, *J* = 6.1 Hz, 1H), 1.68 (s, 3H), 0.63 (dd, *J* = 6.1, 3.4 Hz, 6H), 0.54 (s, 3H), 0.37 (s, 3H), 0.11 (d, *J* = 13.3 Hz, 6H). **^{13}C NMR (101 MHz, $CDCl_3$)** δ 158.5 (d, *J* = 241.1 Hz), 152.5, 152.0, 150.1, 148.7, 142.2, 137.6, 136.0, 134.6 (d, *J* = 19.2 Hz), 129.8 (d, *J* = 5.1 Hz), 128.7, 127.8, 126.8 (d, *J* = 8.1 Hz), 126.0, 123.7 (d, *J* = 4.0 Hz), 120.5, 115.1 (d, *J* = 23.2 Hz), 65.1, 25.1, 25.0, 21.4, 1.9, 1.8, -0.2. **^{19}F NMR (377 MHz, $CDCl_3$)** δ -115.19, -115.21, -115.23. **HRMS (ESI)** m/z Calcd. for $C_{25}H_{33}FNSi_2$ $[M+H]^+$ 438.2079, Found 438.2079. **Minor product** (**3dy′**):**^{1}H NMR (400 MHz, $CDCl_3$)** δ 8.89 (dd, *J* = 4.2, 1.9 Hz, 1H), 8.11 (dd, *J* = 8.2, 1.9 Hz, 1H), 8.00 (dd, *J* = 6.8, 1.5 Hz, 1H), 7.80 (dd, *J* = 8.1, 1.5 Hz, 1H), 7.52 (dd, *J* = 8.1, 6.8 Hz, 1H), 7.35 (dd, *J* = 8.3, 4.2 Hz, 1H), 7.19 – 7.13 (m, 1H), 7.11 – 6.99 (m, 2H), 6.92 (td, *J* = 7.5, 1.9 Hz, 1H), 3.91 (hept, *J* = 6.1 Hz, 1H), 1.58 (s, 3H), 0.92 (d, *J* = 6.2 Hz, 3H), 0.84 (d, *J* = 6.1 Hz, 3H), 0.77 (s, 3H), 0.70 (s, 3H), -0.00 (d, *J* = 10.8 Hz, 6H). **^{13}C NMR (101 MHz, $CDCl_3$)** δ 158.9 (d, *J* = 242.4 Hz), 154.9, 152.6, 148.8, 147.9, 141.8, 137.0, 136.0, 133.9 (d, *J* = 16.2 Hz), 130.1 (d, *J* = 5.1 Hz), 128.8, 127.8, 127.2 (d, *J* = 8.1 Hz), 126.2, 123.9 (d, *J* = 3.0 Hz), 120.6, 115.3 (d, *J* = 23.2 Hz), 65.5, 25.5, 25.4, 23.6, 1.1, 1.1, 0.6, 0.4, 0.3. **^{19}F NMR (377 MHz, $CDCl_3$)** δ -114.51, -114.53, -114.55, -114.57. **HRMS (ESI)** m/z Calcd. for $C_{25}H_{33}FNSi_2$ $[M+H]^+$ 438.2079, Found 438.2077.

(*Z*)-8-((2-(isopropoxydimethylsilyl)-1-(naphthalen-1-yl)prop-1-en-1-yl)dimethylsilyl)quinoline (3dz) and **(Z)-8-((1-(isopropoxydimethylsilyl)-1-(naphthalen-1-yl)prop-1-en-2-yl)dimethylsilyl)quinoline** (**3dz′**)

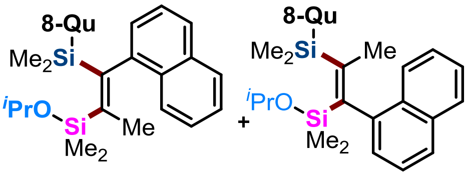

3dz **3dz'**

3dz:3dz' = 90:10

Following the general procedure, in the nitrogen-filled glovebox, to an oven-dried 8-mL sealed tube equipped with a Teflon-coated magnetic stir bar were added Ni(COD)$_2$ (2.8 mg, 0.01 mmol, 5.0 mol%), BrettPhos (10.8 mg, 0.02 mmol, 10.0 mol%), DMF (2 mL), and the reaction mixture was stirred for 15 min, then disilane reagent **1d** (61 mg, 0.2 mmol, 1.0 equiv), 1-methyl-1-(prop-1-yn-1-yl)naphthalene **2z** (66.4 mg, 0.4 mmol, 2.0 equiv) were added. The vial was sealed with a screw-top septum cap, removed from the glovebox and placed in a heating block that was pre-heated to 100 ℃ with vigorous stirring for 24 h under N_2 atmosphere. The reaction mixture is cooled to room temperature and the regioselectivity was determined by GC analysis (r.r. = 90:10). After removal of the solvent, the residue was purified by reversed phase C18(ODS) column (5μm, 21.2×250 mm) with MeCN to afford **3dz** and **3dz′** (70 mg, 75%) as white solid. **R*f*** (PE/DCM = 10/1): 0.4. **Major product** (**3dz**): **^{1}H NMR (400 MHz, CDCl$_3$)** δ 8.91 (dd, *J* = 4.2, 1.9 Hz, 1H), 8.29 (dd, *J* = 6.8, 1.6 Hz, 1H), 8.13 – 8.06 (m, 2H), 7.89 – 7.84 (m, 1H), 7.81 (dd, *J* = 8.1, 1.5 Hz, 1H), 7.67 (d, *J* = 8.2 Hz, 1H), 7.56 (dd, *J* = 8.1, 6.7 Hz, 1H), 7.52 – 7.45 (m, 2H), 7.43 (dd, *J* = 8.2, 7.0 Hz, 1H), 7.34 (dd, *J* = 8.2, 4.2 Hz, 1H), 7.23 (dd, *J* = 7.0, 1.2 Hz, 1H), 3.77 (hept, *J* = 6.1 Hz, 1H), 1.59 (s, 3H), 0.82 (d, *J* = 6.1 Hz, 3H), 0.70 (s, 3H), 0.57 (d, *J* = 6.1 Hz, 3H), 0.15 (d, *J* = 15.1 Hz, 6H), -0.00 (s, 3H). **^{13}C NMR (101 MHz, CDCl$_3$)** δ 154.7, 152.6, 151.4, 148.6, 145.7, 142.3, 137.7, 136.0, 133.7, 1313, 128.7, 128.4, 127.8, 126.4, 125.9, 125.7, 125.5, 125.5, 125.2, 123.6, 120.5, 65.2, 65.2, 25.3, 25.0, 21.4, 2.4, 1.2, 0.1, -0.3. **HRMS (ESI)** m/z Calcd. for $C_{29}H_{36}NOSi_2$ $[M+H]^+$ 470.2330, Found 470.2327. **Minor product** (**3dz′**): **^{1}H NMR (400 MHz, CDCl$_3$)** δ 8.99 (dd, *J* = 4.3, 1.9 Hz, 1H), 8.14 (dd, *J* = 8.3, 1.9 Hz, 1H), 8.01 (ddd, *J* = 17.2, 7.5, 1.5 Hz, 2H), 7.85 – 7.77 (m, 2H), 7.65 (d, *J* = 8.2 Hz, 1H), 7.55 (dd, *J* = 8.1, 6.7 Hz, 1H), 7.44 – 7.36 (m,

4H), 7.05 (dd, J = 7.0, 1.3 Hz, 1H), 3.97 (hept, J = 6.1 Hz, 1H), 1.40 (s, 3H), 1.01 (dd, J = 8.9, 6.1 Hz, 6H), 0.82 (s, 3H), 0.74 (s, 3H), -0.14 (d, J = 4.7 Hz, 6H). **^{13}C NMR (101 MHz, $CDCl_3$)** δ 154.4, 148.9, 144.7, 142.5, 136.6, 136.2, 133.8, 131.5, 128.9, 128.3, 127.8, 126.7, 126.2, 125.6, 125.5, 125.4, 125.3, 124.4, 120.7, 65.5, 29.8, 25.7, 25.7, 23.3, 1.0, 0.83, 0.80, 0.5. **HRMS (ESI)** m/z Calcd. for $C_{29}H_{36}NOSi_2$ $[M+H]^+$ 470.2330, Found 470.2328.

(Z)-8-((2-(isopropoxydimethylsilyl)-1-phenylpent-1-en-1-yl)dimethylsilyl)quinoline (3dä) and **(Z)-8-((1-(isopropoxydimethylsilyl)-1-phenylpent-1-en-2-yl)dimethylsilyl)quinoline** (3dä′)

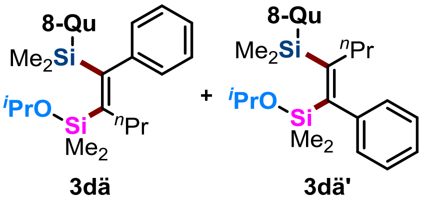

3dä:3dä', r.r. = 73:27

Following the general procedure, in the nitrogen-filled glovebox, to an oven-dried 8-mL sealed tube equipped with a Teflon-coated magnetic stir bar were added $Ni(COD)_2$ (2.8 mg, 0.01 mmol, 5.0 mol%), BrettPhos (10.8 mg, 0.02 mmol, 10.0 mol%), DMF (2 mL), and the reaction mixture was stirred for 15 min, then disilane reagent **1d** (61 mg, 0.2 mmol, 1.0 equiv), pent-1-yn-1-ylbenzene **2ä** (58 mg, 0.4 mmol, 2.0 equiv) were added. The vial was sealed with a screw-top septum cap, removed from the glovebox and placed in a heating block that was pre-heated to 100 ℃ with vigorous stirring for 24 h under N_2 atmosphere. After been cooled to room temperature, the reaction mixture was filtered through a pad of celite and concentrated in vacuo. The regioselectivity was determined by ^{1}H NMR of crude materials (r.r. = 73:27). The residue was purified by reversed phase C18(ODS) column (5μm, 21.2×250 mm) with MeCN to afford **3dä** and **3dä′** (58 mg, 65%) as white solid. **R_f** (PE/DCM = 20/1): 0.4. **Major product** (**3dä**): **^{1}H NMR (400 MHz, $CDCl_3$)** δ 8.90 (dd, J = 4.1, 1.9 Hz, 1H), 8.09 (dd, J = 8.2, 1.9 Hz, 1H), 8.04 (dd, J = 6.8, 1.5 Hz, 1H), 7.77 (dd, J = 8.2, 1.5 Hz, 1H), 7.48 (dd, J = 8.1, 6.8 Hz, 1H), 7.33 (dd, J = 8.2, 4.1 Hz, 1H), 7.18 (t, J = 7.6 Hz, 2H), 7.10 – 7.03 (m, 1H), 7.02 – 6.94 (m, 2H), 3.83 (hept, J = 6.1 Hz, 1H), 2.06 – 1.93 (m, 2H), 1.29 – 1.21 (m, 2H), 0.81 (d, J = 6.1 Hz, 6H), 0.67 (t, J = 7.3 Hz, 3H), 0.40 (s, 6H), 0.04 (s, 6H). **^{13}C NMR (101 MHz, $CDCl_3$)** δ 156.9, 153.7, 152.6, 148.6, 147.2,

142.1, 137.5, 136.0, 128.6, 127.7, 127.7, 127.4, 125.9, 124.6, 120.5, 65.1, 37.2, 25.4, 23.9, 14.7, 1.7, 0.8. **HRMS (ESI)** m/z Calcd. for $C_{27}H_{38}NOSi_2$ $[M+H]^+$ 448.2486, Found 448.2485. **Minor product** (**3dä′**): **^{1}H NMR (400 MHz, CDCl$_3$)** δ 8.97 – 8.86 (m, 1H), 8.11 (dd, *J* = 8.2, 1.8 Hz, 1H), 8.07 (dd, *J* = 6.8, 1.5 Hz, 1H), 7.79 (dd, *J* = 8.2, 1.5 Hz, 1H), 7.51 (dd, *J* = 8.1, 6.8 Hz, 1H), 7.35 (dd, *J* = 8.2, 4.2 Hz, 1H), 7.28 (t, *J* = 7.5 Hz, 2H), 7.20 – 7.12 (m, 1H), 7.02 – 6.96 (m, 2H), 3.69 (hept, *J* = 6.1 Hz, 1H), 2.04 – 1.93 (m, 2H), 1.29 – 1.15 (m, 3H), 0.73 (s, 6H), 0.64 (d, *J* = 6.1 Hz, 6H), 0.54 (t, *J* = 7.3 Hz, 3H), -0.16 (s, 6H). **^{13}C NMR (101 MHz, CDCl$_3$)** δ 156.2, 155.1, 148.7, 146.0, 142.9, 137.6, 136.1, 128.6, 128.5, 127.8, 126.0, 125.1, 120.5, 65.2, 38.7, 25.1, 24.1, 14.56, 1.9, 0.1. **HRMS (ESI)** m/z Calcd. for $C_{27}H_{38}NOSi_2$ $[M+H]^+$ 448.2486, Found 448.2486.

Pd-Catalyzed Bissilylation of Terminal Alkynes

Supplementary Table 4. Optimization of reaction conditions[a]

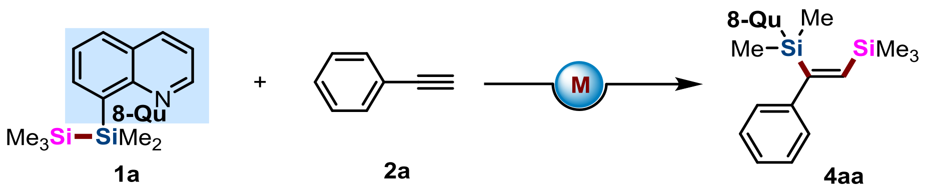

Entry	Catalyst	Ligand	Additive	Solvent	Yield[%][b]
1	Ni(COD)$_2$ (10 mol%)	SIPr (12 mol%)	none	toluene	N.R.
2	Ni(COD)$_2$ (5 mol%)	SPhos (10 mol%)	none	toluene	N.R.
3	Ni(COD)$_2$ (5 mol%)	BrettPhos (10 mol%)	none	toluene	N.R.
4	Pd(acac)$_2$ (5 mol%)	*t*-BuNC (60 mol%)	*t*-BuOK	toluene	65
**4**	**Pd(acac)$_2$ (5 mol%)**	***t*-OcNC (60 mol%)**	*t*-BuOK	toluene	**74**
5	Pd(TFA)$_2$ (5 mol%)	*t*-OcNC (60 mol%)	*t*-BuOK	toluene	64

[a] Reactions were carried out with [M] precatalyst, ligand, additive (10 mol%), disilane **1a** (0.20 mmol) and phenylacetylene **2a** (0.40 mmol, 2 eq.) in toluene for 24 h at 120 ℃ under an N_2 atmosphere. [b] Yields of isolated products. N.R.: No reaction (N.R.). N.D.: not determined.

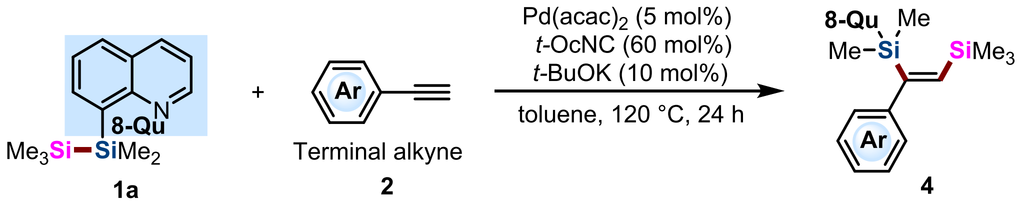

General procedure: Following a modified literature procedure,[3] in the nitrogen-filled glovebox, to an oven-dried 8-mL sealed tube equipped with a Teflon-coated magnetic stir bar were added Pd(acac)$_2$ (3 mg, 0.01 mmol, 5.0 mol%), *t*-OcNC (17 mg, 0.12 mmol, 60 mol%), toluene (2 mL), and then **1a** (52 mg, 0.2 mmol, 1.0 equiv), terminal alkynes **2** (0.4 mmol, 2.0 equiv) and *t*-BuOK (2.3 mg, 0.02 mmol, 10 mol%) were added successively. The vial was sealed with a screw-top septum cap, removed from the glovebox and placed in a heating block that was pre-heated to 120 ℃ with vigorous stirring for 24 h under N_2 atmosphere. After been cooled to room temperature, the reaction mixture was filtered through a pad of celite and concentrated in vacuo. The

resulting residue was purified by silica gel flash chromatography to give the desired product **4**.

(*Z*)-8-(Dimethyl(1-phenyl-2-(trimethylsilyl)vinyl)silyl)quinoline (4aa)

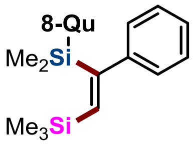

Following the general procedure, in the nitrogen-filled glovebox, to an oven-dried 8-mL sealed tube equipped with a Teflon-coated magnetic stir bar were added Pd(acac)$_2$ (3 mg, 0.01 mmol, 5.0 mol%), *t*-OcNC (17 mg, 0.12 mmol, 60 mol%), toluene (2 mL), and then **1a** (52 mg, 0.2 mmol, 1.0 equiv), phenylacetylene **2a** (41 mg, 44 μL, 0.4 mmol, 2.0 equiv) and *t*-BuOK (2.3 mg, 0.02 mmol, 10 mol%) were added successively. The vial was sealed with a screw-top septum cap, removed from the glovebox and placed in a heating block that was pre-heated to 120 ℃ with vigorous stirring for 24 h under N_2 atmosphere. After been cooled to room temperature, the reaction mixture was filtered through a pad of celite and concentrated in vacuo. The resulting residue was purified by silica gel flash chromatography (PE/DCM = 20/1) to give the desired product **4aa** (53 mg, 74% yield, r.r. = 100 : 0, colorless oil). **R*f*** (PE/DCM = 20/1): 0.4. **^{1}H NMR (400 MHz, CDCl$_3$)** δ 8.94 (dd, *J* = 4.1, 1.8 Hz, 1H), 8.16 – 8.09 (m, 2H), 7.87 (dd, *J* = 8.2, 1.5 Hz, 1H), 7.57 (dd, *J* = 8.1, 6.7 Hz, 1H), 7.38 (dd, *J* = 8.2, 4.2 Hz, 1H), 7.28 (d, *J* = 4.3 Hz, 4H), 7.23 – 7.15 (m, 1H), 6.58 (s, 1H), 0.57 (s, 6H), -0.10 (s, 9H). **^{13}C NMR (101 MHz, CDCl$_3$)** δ 163.4, 152.6, 151.5, 149.0, 148.9, 140.7, 137.6, 136.1, 129.6, 127.9, 127.7, 127.0, 126.0, 125.6, 120.8, 1.2, 0.7. **HRMS (ESI)** m/z Calcd. for $C_{22}H_{28}NSi_2$ $[M+H]^+$ 362.1755, Found 362.1754.

(*Z*)-8-(Dimethyl(1-(*p*-tolyl)-2-(trimethylsilyl)vinyl)silyl)quinoline (4ab)

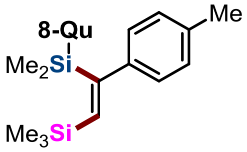

Following the general procedure, in the nitrogen-filled glovebox, to an oven-dried 8-mL sealed tube equipped with a Teflon-coated magnetic stir bar were added Pd(acac)$_2$ (3 mg, 0.01 mmol, 5.0 mol%), *t*-OcNC (17 mg, 0.12 mmol, 60 mol%), toluene (2 mL), and then **1a** (52 mg, 0.2 mmol, 1.0 equiv), 1-ethynyl-4-methylbenzene (46.4 mg, 0.4 mmol, 2.0 equiv) and *t*-BuOK (2.3 mg, 0.02 mmol, 10 mol%) were added successively. The vial was sealed

with a screw-top septum cap, removed from the glovebox and placed in a heating block that was pre-heated to 120 ℃ with vigorous stirring for 24 h under N_2 atmosphere. After been cooled to room temperature, the reaction mixture was filtered through a pad of celite and concentrated in vacuo. The resulting residue was purified by silica gel flash chromatography (PE/DCM = 20/1) to give the desired product **4ab** (50 mg, 66% yield, r.r. ≥ 95 : 5, colorless oil). **R_f** (PE/DCM = 20/1): 0.4. **^{1}H NMR (400 MHz, $CDCl_3$)** δ 8.92 (dd, J = 4.2, 1.9 Hz, 1H), 8.16 – 8.08 (m, 2H), 7.86 (dd, J = 8.1, 1.5 Hz, 1H), 7.56 (dd, J = 8.1, 6.8 Hz, 1H), 7.38 (dd, J = 8.3, 4.3 Hz, 1H), 7.21 – 7.14 (m, 2H), 7.09 (d, J = 7.8 Hz, 2H), 6.56 (s, 1H), 2.35 (s, 3H), 0.55 (s, 6H), -0.12 (s, 9H). **^{13}C NMR (101 MHz, $CDCl_3$)** δ 163.2, 152.6, 149.0, 148.9, 148.6, 140.8, 137.7, 136.1, 135.1, 131.5, 129.6, 129.5, 129.3, 128.4, 127.9, 126.9, 126.3, 126.0, 120.8, 21.2, 1.3, 0.7. **HRMS (ESI)** m/z Calcd. for $C_{23}H_{30}NSi_2$ $[M+H]^+$ 376.1911, Found 376.1911.

(*Z*)-8-((1-(4-Methoxyphenyl)-2-(trimethylsilyl)vinyl)dimethylsilyl)quinoline (4ac)

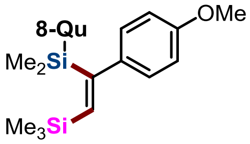

Following the general procedure, in the nitrogen-filled glovebox, to an oven-dried 8-mL sealed tube equipped with a Teflon-coated magnetic stir bar were added Pd(acac)$_2$ (3 mg, 0.01 mmol, 5.0 mol%), *t*-OcNC (17 mg, 0.12 mmol, 60 mol%), toluene (2 mL), and then **1a** (52 mg, 0.2 mmol, 1.0 equiv), 1-ethynyl-4-methoxybenzene (53 mg, 0.4 mmol, 2.0 equiv) and *t*-BuOK (2.3 mg, 0.02 mmol, 10 mol%) were added successively. The vial was sealed with a screw-top septum cap, removed from the glovebox and placed in a heating block that was pre-heated to 120 ℃ with vigorous stirring for 24 h under N_2 atmosphere. After been cooled to room temperature, the reaction mixture was filtered through a pad of celite and concentrated in vacuo. The resulting residue was purified by silica gel flash chromatography (PE/DCM = 10/1) to give the desired product **4ac** (50 mg, 64% yield, r.r. ≥ 95 : 5, colorless oil). **R_f** (PE/DCM = 10/1): 0.4. **^{1}H NMR (400 MHz, $CDCl_3$)** δ 8.91 (dd, J = 4.2, 1.8 Hz, 1H), 8.13 (dd, J = 8.2, 1.8 Hz, 1H), 8.08 (dd, J = 6.8, 1.5 Hz, 1H), 7.85 (dd, J = 8.2, 1.5 Hz, 1H), 7.55 (dd, J = 8.1, 6.7 Hz, 1H), 7.37 (dd, J = 8.2, 4.1 Hz, 1H), 7.21 – 7.16 (m, 2H), 6.84 – 6.79 (m, 2H), 6.55 (s, 1H), 3.80 (s, 3H), 0.54 (s, 6H), -0.12 (s, 9H). **^{13}C NMR (101 MHz, $CDCl_3$)** δ 162.7, 157.8, 152.6, 149.0, 148.7,

144.1, 140.8, 137.6, 136.1, 129.54, 128.0, 127.9, 126.0, 120.8, 113.1, 55.3, 1.3, 0.7. **HRMS (ESI)** m/z Calcd. for $C_{23}H_{30}NOSi_2$ $[M+H]^+$ 392.1860, Found 392.1860.

(*Z*)-8-((1-(4-Fluorophenyl)-2-(trimethylsilyl)vinyl)dimethylsilyl)quinoline (4ad)

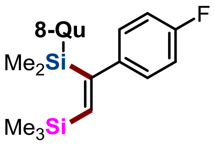

Following the general procedure, in the nitrogen-filled glovebox, to an oven-dried 8-mL sealed tube equipped with a Teflon-coated magnetic stir bar were added $Pd(acac)_2$ (3 mg, 0.01 mmol, 5.0 mol%), *t*-OcNC (17 mg, 0.12 mmol, 60 mol%), toluene (2 mL), and then **1a** (52 mg, 0.2 mmol, 1.0 equiv), 1-ethynyl-4-fluorobenzene (48 mg, 0.4 mmol, 2.0 equiv) and *t*-BuOK (2.3 mg, 0.02 mmol, 10 mol%) were added successively. The vial was sealed with a screw-top septum cap, removed from the glovebox and placed in a heating block that was pre-heated to 120 ℃ with vigorous stirring for 24 h under N_2 atmosphere. After been cooled to room temperature, the reaction mixture was filtered through a pad of celite and concentrated in vacuo. The resulting residue was purified by silica gel flash chromatography (PE/DCM = 20/1) to give the desired product **4ad** (53 mg, 70% yield, r.r. ≥ 95 : 5, colorless oil). **R_f** (PE/DCM = 20/1): 0.4. **^{1}H NMR (400 MHz, $CDCl_3$)** δ 8.90 (dd, *J* = 4.2, 2.2 Hz, 1H), 8.13 (dd, *J* = 8.3, 1.9 Hz, 1H), 8.05 (dd, *J* = 6.7, 2.4 Hz, 1H), 7.86 (dt, *J* = 8.2, 1.2 Hz, 1H), 7.59 – 7.51 (m, 1H), 7.41 – 7.34 (m, 1H), 7.20 (ddd, *J* = 8.2, 4.1, 1.8 Hz, 2H), 6.98 – 6.88 (m, 2H), 6.50 (d, *J* = 0.9 Hz, 1H), 0.52 (s, 6H), -0.14 (s, 9H). **^{13}C NMR (101 MHz, $CDCl_3$)** δ 162.5, 160.1, 152.5, 149.3, 149.0, 147.4 (d, *J* = 3.0 Hz), 140.5, 137.4, 136.1, 129.7, 128.4 (d, *J* = 8.1 Hz), 127.9, 126.1, 120.9, 114.3 (d, *J* = 21.2 Hz), 1.1, 0.6. **^{19}F NMR (377 MHz, $CDCl_3$)** δ -118.31, -118.33, -118.34, -118.37. **HRMS (ESI)** m/z Calcd. for $C_{22}H_{27}FNSi_2$ $[M+H]^+$ 380.1661, Found 380.1663.

(Z)-8-(Dimethyl(1-(4-(trifluoromethyl)phenyl)-2-(trimethylsilyl)vinyl)silyl)quinoline (4ae)

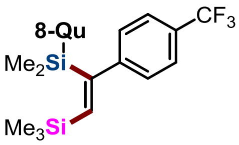

Following the general procedure, in the nitrogen-filled glovebox, to an oven-dried 8-mL sealed tube equipped with a Teflon-coated magnetic stir bar were added $Pd(acac)_2$ (3 mg, 0.01 mmol, 5.0 mol%), *t*-OcNC (17 mg, 0.12 mmol, 60 mol%), toluene (2 mL), and then **1a** (52 mg,

0.2 mmol, 1.0 equiv), 1-ethynyl-4-(trifluoromethyl)benzene (68 mg, 0.4 mmol, 2.0 equiv) and *t*-BuOK (2.3 mg, 0.02 mmol, 10 mol%) were added successively. The vial was sealed with a screw-top septum cap, removed from the glovebox and placed in a heating block that was pre-heated to 120 ℃ with vigorous stirring for 24 h under N_2 atmosphere. After been cooled to room temperature, the reaction mixture was filtered through a pad of celite and concentrated in vacuo. The resulting residue was purified by silica gel flash chromatography (PE/DCM = 20/1) to give the desired product **4ae** (59 mg, 69% yield, r.r. = 88.5 : 11.5, colorless oil). **R*f*** (PE/DCM = 20/1): 0.4. **^{1}H NMR (400 MHz, $CDCl_3$)** δ 8.80 (dt, *J* = 4.1, 1.4 Hz, 1H), 8.03 (dt, *J* = 8.4, 1.3 Hz, 1H), 7.95 (dt, *J* = 6.8, 1.3 Hz, 1H), 7.77 (dt, *J* = 8.1, 1.3 Hz, 1H), 7.46 (ddd, *J* = 8.0, 6.7, 0.9 Hz, 1H), 7.40 (d, *J* = 8.0 Hz, 2H), 7.30 – 7.23 (m, 3H), 6.39 (d, *J* = 1.0 Hz, 1H), 0.42 (s, 7H), -0.26 (s, 9H), -0.31 (s, 1H). **^{13}C NMR (101 MHz, $CDCl_3$)** δ 162.6, 155.22, 155.20, 152.5, 150.0, 148.97, 148.91, 140.1, 137.4, 136.9, 136.2, 129.8, 129.6, 127.92, 127.86, 127.77, 127.5, 127.3, 126.08, 126.05, 124.6 (d, *J* = 4.1 Hz), 123.4, 121.0, 120.9, 1.0, 0.5, 0.2, -1.4. **^{19}F NMR (377 MHz, $CDCl_3$)** δ -62.04. **HRMS (ESI)** m/z Calcd. for $C_{23}H_{27}F_3NSi_2$ $[M+H]^+$ 430.1629, Found 430.1627.

(*Z*)-8-((1-(3-Methoxyphenyl)-2-(trimethylsilyl)vinyl)dimethylsilyl)quinoline (4af)

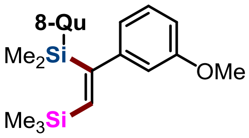

Following the general procedure, in the nitrogen-filled glovebox, to an oven-dried 8-mL sealed tube equipped with a Teflon-coated magnetic stir bar were added $Pd(acac)_2$ (3 mg, 0.01 mmol, 5.0 mol%), *t*-OcNC (17 mg, 0.12 mmol, 60 mol%), toluene (2 mL), and then **1a** (52 mg, 0.2 mmol, 1.0 equiv), 1-ethynyl-3-methoxybenzene (53 mg, 0.4 mmol, 2.0 equiv) and *t*-BuOK (2.3 mg, 0.02 mmol, 10 mol%) were added successively. The vial was sealed with a screw-top septum cap, removed from the glovebox and placed in a heating block that was pre-heated to 120 ℃ with vigorous stirring for 24 h under N_2 atmosphere. After been cooled to room temperature, the reaction mixture was filtered through a pad of celite and concentrated in vacuo. The resulting residue was purified by silica gel flash chromatography (PE/DCM = 10/1) to give the desired product **4af** (56 mg, 72% yield, r.r. = 91 : 9, colorless oil). **R*f*** (PE/DCM = 10/1): 0.4. **^{1}H NMR (400 MHz, $CDCl_3$)**

δ 8.92 (dd, *J* = 4.2, 1.8 Hz, 1H), 8.12 (dd, *J* = 8.3, 1.9 Hz, 1H), 8.08 (dd, *J* = 6.8, 1.5 Hz, 1H), 7.85 (dd, *J* = 8.1, 1.5 Hz, 1H), 7.55 (dd, *J* = 8.1, 6.8 Hz, 1H), 7.37 (dd, *J* = 8.3, 4.1 Hz, 1H), 7.17 (t, *J* = 8.0 Hz, 1H), 6.87 – 6.80 (m, 2H), 6.74 – 6.71 (m, 1H), 6.54 (s, 1H), 3.77 (s, 3H), 0.67 (s, 1H), 0.54 (s, 6H), -0.00 (s, 1H), -0.14 (s, 9H). **^{13}C NMR (101 MHz, $CDCl_3$)** δ 163.3, 159.0, 153.0, 152.6, 148.9, 148.7, 140.6, 137.5, 136.1, 129.6, 128.6, 127.9, 126.0, 120.9, 1196, 112.7, 111.0, 55.2, 11, 0.6. **HRMS (ESI)** m/z Calcd. for $C_{23}H_{30}NOSi_2$ $[M+H]^+$ 392.1860, Found 392.1860.

Pd-Catalyzed Bissilylation of Aryne Precursors

Supplementary Table 5. Optimization of reaction conditions[a]

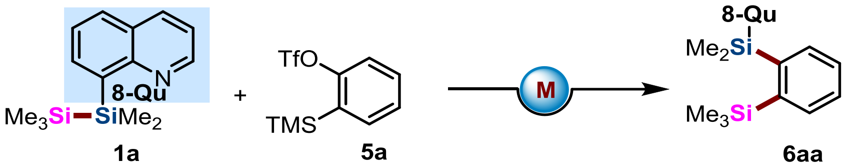

Entry	[M] Catalyst	Ligand	Base/Additive	Solvent	Yield[%][b]
1[c]	Ni(COD)$_2$ (10 mol%)	SIPr (12 mol%)	KF/18-crown-6	toluene	N.R.
2	Ni(COD)$_2$ (10 mol%)	SIPr (12 mol%)	CsF	Toluene/THF	12
3	Pd(acac)$_2$ (5 mol%)	*t*-OcNC (60 mol%)	CsF	THF	49
**4**	**Pd(acac)$_2$ (5 mol%)**	***t*-OcNC (60 mol%)**	**KF/18-crown-6**	**THF**	**83**
5	Pd(acac)$_2$ (5 mol%)	*t*-OcNC (60 mol%)	CsF	MeCN	80
6	Pd(acac)$_2$ (5 mol%)	*t*-OcNC (60 mol%)	KF/18-crown-6	MeCN	33
7[d]	Pd(acac)$_2$ (5 mol%)	*t*-OcNC (60 mol%)	KF/18-crown-6	THF	52

[a] Reactions were carried out with [M] precatalyst, ligand, base (3 equiv), additive (3 equiv), disilane **1a** (0.20 mmol) and 2-(trimethylsilyl)phenyl trifluoromethanesulfonate **5a** (0.40 mmol, 2 eq.) in solvent for 24 h at 50 ℃ under an N_2 atmosphere. [b] Yields of isolated products. [c] The reaction was conducted at 100 ℃. [d] The reaction was conducted at 80 ℃.

General procedure: In the nitrogen-filled glovebox, to an oven-dried 8-mL sealed tube equipped with a Teflon-coated magnetic stir bar were added Pd(acac)$_2$ (3 mg, 0.01 mmol, 5.0 mol%), *t*-OcNC (17 mg, 0.12 mmol, 60 mol%), THF (2 mL), and then TMDQ reagent **1** (0.2 mmol, 1.0 equiv), aryne precursor **5** (0.4 mmol, 2.0 equiv) and KF (35 mg, 0.6 mmol, 3.0 equiv), 18-crown-6 (159 mg, 0.6 mmol, 3.0 equiv) were added successively. The vial was sealed with a screw-top septum cap, removed from the glovebox and placed in a heating block that was pre-heated to 50 ℃ with vigorous stirring for 24 h under N_2 atmosphere. After been cooled to room temperature, the reaction mixture was filtered through a pad of celite and concentrated in vacuo. The resulting residue was purified by silica gel flash chromatography to give the desired product **6**.

8-(Dimethyl(2-(trimethylsilyl)phenyl)silyl)quinoline (6aa)

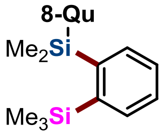

In the nitrogen-filled glovebox, to an oven-dried 8-mL sealed tube equipped with a Teflon-coated magnetic stir bar were added $Pd(acac)_2$ (3 mg, 0.01 mmol, 5.0 mol%), *t*-OcNC (17 mg, 0.12 mmol, 60 mol%), THF (2 mL), and then **1a** (52 mg, 0.2 mmol, 1.0 equiv), 2-(trimethylsilyl)phenyl trifluoromethanesulfonate **5a** (120 mg, 0.4 mmol, 2.0 equiv) and KF (35 mg, 0.6 mmol, 3.0 equiv), 18-crown-6 (159 mg, 0.6 mmol, 3.0 equiv) were added successively. The vial was sealed with a screw-top septum cap, removed from the glovebox and placed in a heating block that was pre-heated to 50 ℃ with vigorous stirring for 24 h under N_2 atmosphere. After been cooled to room temperature, the reaction mixture was filtered through a pad of celite and concentrated in vacuo. The resulting residue was purified by silica gel flash chromatography (PE) to give the desired product **6aa** (56 mg, 83% yield, colorless viscous liquid). **R*f*** (PE): 0.5. **^{1}H NMR (400 MHz, $CDCl_3$)** δ 8.87 (dd, *J* = 4.2, 1.8 Hz, 1H), 8.11 (dd, *J* = 8.2, 1.9 Hz, 1H), 7.83 – 7.74 (m, 3H), 7.59 (dd, *J* = 6.8, 1.5 Hz, 1H), 7.44 – 7.33 (m, 4H), 0.86 (s, 6H), 0.16 (s, 9H). **^{13}C NMR (101 MHz, $CDCl_3$)** δ 152.6, 149.1, 146.9, 145.5, 141.6, 137.9, 136.3, 136.1, 135.4, 135.4, 129.3, 127.8, 127.8, 125.9, 120.7, 2.1, 1.9. **HRMS (ESI)** m/z Calcd. for $C_{20}H_{26}NSi_2$ $[M+H]^+$ 336.1598, Found 336.1602.

8-((4,5-Dimethyl-2-(trimethylsilyl)phenyl)dimethylsilyl)quinoline (6ab)

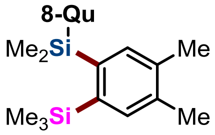

In the nitrogen-filled glovebox, to an oven-dried 8-mL sealed tube equipped with a Teflon-coated magnetic stir bar were added $Pd(acac)_2$ (3 mg, 0.01 mmol, 5.0 mol%), *t*-OcNC (17 mg, 0.12 mmol, 60 mol%), THF (2 mL), and then **1a** (52 mg, 0.2 mmol, 1.0 equiv), 4,5-dimethyl-2-(trimethylsilyl)phenyl trifluoromethanesulfonate **5b** (130.4 mg, 0.4 mmol, 2.0 equiv) and KF (35 mg, 0.6 mmol, 3.0 equiv), 18-crown-6 (159 mg, 0.6 mmol, 3.0 equiv) were added successively. The vial was sealed with a screw-top septum cap, removed from the glovebox and placed in a heating block that was pre-heated to 50 ℃ with vigorous stirring for 24 h under N_2 atmosphere. After been cooled to room temperature, the reaction mixture was filtered through a pad of celite and concentrated in vacuo. The

resulting residue was purified by silica gel flash chromatography (PE) to give the desired product **6ab** (70 mg, 96% yield, colorless viscous liquid). **R*f*** (PE): 0.4. **^{1}H NMR (400 MHz, $CDCl_3$)** δ 8.94 (dd, *J* = 4.2, 1.9 Hz, 1H), 8.14 (dd, *J* = 8.2, 1.9 Hz, 1H), 7.83 (dd, *J* = 8.1, 1.6 Hz, 1H), 7.68 – 7.62 (m, 2H), 7.59 (s, 1H), 7.44 (dd, *J* = 8.1, 6.8 Hz, 1H), 7.38 (dd, *J* = 8.3, 4.1 Hz, 1H), 2.39 (s, 3H), 2.35 (s, 3H), 0.92 (s, 6H), 0.20 (s, 9H). **^{13}C NMR (101 MHz, $CDCl_3$)** δ 152.6, 149.1, 143.9, 142.3, 141.7, 138.1, 137.3, 136.1, 136.1, 129.2, 127.8, 125.9, 120.7, 19.9, 19.8, 2.2, 2.0. **HRMS (ESI)** m/z Calcd. for $C_{22}H_{30}NSi_2$ $[M+H]^+$ 364.1911, Found 364.1909.

8-(Dimethyl(6-(trimethylsilyl)benzo[*d*][1,3]dioxol-5-yl)silyl)quinoline (6ac)

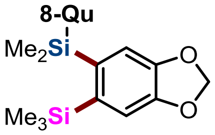

In the nitrogen-filled glovebox, to an oven-dried 8-mL sealed tube equipped with a Teflon-coated magnetic stir bar were added $Pd(acac)_2$ (3 mg, 0.01 mmol, 5.0 mol%), *t*-OcNC (17 mg, 0.12 mmol, 60 mol%), THF (2 mL), and then **1a** (52 mg, 0.2 mmol, 1.0 equiv), 6-(trimethylsilyl)benzo[*d*][1,3]dioxol-5-yl trifluoromethanesulfonate **5c** (136.8 mg, 0.4 mmol, 2.0 equiv) and KF (35 mg, 0.6 mmol, 3.0 equiv), 18-crown-6 (159 mg, 0.6 mmol, 3.0 equiv) were added successively. The vial was sealed with a screw-top septum cap, removed from the glovebox and placed in a heating block that was pre-heated to 50 ℃ with vigorous stirring for 24 h under N_2 atmosphere. After been cooled to room temperature, the reaction mixture was filtered through a pad of celite and concentrated in vacuo. The resulting residue was purified by silica gel flash chromatography (PE) to give the desired product **6ac** (72 mg, 95% yield, colorless viscous liquid). **R*f*** (PE): 0.4. **^{1}H NMR (400 MHz, $CDCl_3$)** δ 8.89 (dd, *J* = 4.2, 1.9 Hz, 1H), 8.11 (dd, *J* = 8.3, 1.9 Hz, 1H), 7.81 (dd, *J* = 8.1, 1.5 Hz, 1H), 7.64 (dd, *J* = 6.8, 1.5 Hz, 1H), 7.44 (dd, *J* = 8.1, 6.8 Hz, 1H), 7.35 (q, *J* = 3.9 Hz, 2H), 7.31 (s, 1H), 5.96 (s, 2H), 0.86 (s, 6H), 0.18 (s, 9H). **^{13}C NMR (101 MHz, $CDCl_3$)** δ 152.5, 149.1, 147.4, 141.5, 140.6, 139.4, 137.8, 136.1, 129.4, 127.8, 125.9, 120.7, 116.6, 115.7, 100.44, 2.4, 2.3. **HRMS (ESI)** m/z Calcd. for $C_{21}H_{26}NO_2Si_2$ $[M+H]^+$ 380.1497, Found 380.1493.

8-(Dimethyl(6-(trimethylsilyl)-2,3-dihydro-1*H*-inden-5-yl)silyl)quinoline (6ad)

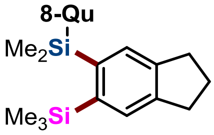

In the nitrogen-filled glovebox, to an oven-dried 8-mL sealed tube equipped with a Teflon-coated magnetic stir bar were added $Pd(acac)_2$ (3 mg, 0.01 mmol, 5.0 mol%), *t*-OcNC (17 mg, 0.12 mmol, 60 mol%), THF (2 mL), and then **1a** (52 mg, 0.2 mmol, 1.0 equiv), 6-(trimethylsilyl)-2,3-dihydro-1*H*-inden-5-yl trifluoromethanesulfonate **5d** (135.2 mg, 0.4 mmol, 2.0 equiv) and KF (35 mg, 0.6 mmol, 3.0 equiv), 18-crown-6 (159 mg, 0.6 mmol, 3.0 equiv) were added successively. The vial was sealed with a screw-top septum cap, removed from the glovebox and placed in a heating block that was pre-heated to 50 ℃ with vigorous stirring for 24 h under N_2 atmosphere. After been cooled to room temperature, the reaction mixture was filtered through a pad of celite and concentrated in vacuo. The resulting residue was purified by silica gel flash chromatography (PE) to give the desired product **6ad** (69 mg, 92% yield, colorless viscous liquid). $\mathbf{R_f}$ (PE): 0.4. **^{1}H NMR (400 MHz, $CDCl_3$)** δ 8.94 (dd, *J* = 4.1, 1.8 Hz, 1H), 8.14 (dd, *J* = 8.2, 1.8 Hz, 1H), 7.83 (dd, *J* = 8.1, 1.2 Hz, 1H), 7.78 (s, 1H), 7.74 (s, 1H), 7.68 – 7.62 (m, 1H), 7.47 – 7.42 (m, 1H), 7.38 (dd, *J* = 8.2, 4.2 Hz, 1H), 3.05 – 2.95 (m, 4H), 2.14 (p, *J* = 7.5 Hz, 2H), 0.92 (s, 6H), 0.21 (s, 9H). **^{13}C NMR (101 MHz, $CDCl_3$)** δ 152.6, 149.1, 144.2, 143.9, 143.9, 142.6, 141.9, 138.1, 136.1, 132.7, 131.9, 129.2, 127.8, 125.9, 120.7, 33.11, 33.07, 25.0, 2.4, 2.2. **HRMS (ESI)** m/z Calcd. for $C_{23}H_{30}NSi_2$ $[M+H]^+$ 376.1911, Found 376.1911.

8-((2-(Dimethyl(phenyl)silyl)phenyl)dimethylsilyl)quinoline (6ba)

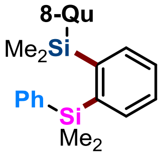

In the nitrogen-filled glovebox, to an oven-dried 8-mL sealed tube equipped with a Teflon-coated magnetic stir bar were added $Pd(acac)_2$ (3 mg, 0.01 mmol, 5.0 mol%), *t*-OcNC (17 mg, 0.12 mmol, 60 mol%), THF (2 mL), and then **1b** (64 mg, 0.2 mmol, 1.0 equiv), 2-(trimethylsilyl)phenyl trifluoromethanesulfonate **5a** (120 mg, 0.4 mmol, 2.0 equiv) and KF (35 mg, 0.6 mmol, 3.0 equiv), 18-crown-6 (159 mg, 0.6 mmol, 3.0 equiv) were added successively. The vial was sealed with a screw-top septum cap, removed from the glovebox and placed in a heating block that was pre-heated to 50 ℃ with vigorous stirring for 24 h under N_2

atmosphere. After been cooled to room temperature, the reaction mixture was filtered through a pad of celite and concentrated in vacuo. The resulting residue was purified by silica gel flash chromatography to give the desired product **6ba** (55 mg, 70% yield, colorless viscous liquid). **R*f*** (PE/DCM = 15/1): 0.4. **^{1}H NMR (400 MHz, CDCl$_3$)** δ 8.73 – 8.63 (m, 1H), 7.94 (d, *J* = 8.2 Hz, 1H), 7.69 – 7.59 (m, 3H), 7.50 (d, *J* = 6.8 Hz, 1H), 7.32 – 7.13 (m, 9H), 0.51 (s, 6H), 0.28 (s, 6H). **^{13}C NMR (101 MHz, CDCl$_3$)** δ 152.4, 149.0, 146.7, 143., 141.70, 140.7, 137.6, 136.8, 136.5, 136.0, 134.3, 129.3, 128.7, 128.1, 127.8, 127.7, 127.6, 125.9, 120.7, 1.7, 1.1. **HRMS (ESI)** m/z Calcd. for $C_{25}H_{28}NSi_2$ $[M+H]^+$ 398.1755, Found 398.1754.

8-(Dimethyl(2-(trimethylsilyl)phenyl)silyl)quinoline (6da)

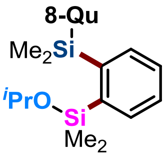

In the nitrogen-filled glovebox, to an oven-dried 8-mL sealed tube equipped with a Teflon-coated magnetic stir bar were added Pd(acac)$_2$ (3 mg, 0.01 mmol, 5.0 mol%), *t*-OcNC (17 mg, 0.12 mmol, 60 mol%), THF (2 mL), and then **1d** (61 mg, 0.2 mmol, 1.0 equiv), 2-(trimethylsilyl)phenyl trifluoromethanesulfonate **5a** (120 mg, 0.4 mmol, 2.0 equiv) and KF (35 mg, 0.6 mmol, 3.0 equiv), 18-crown-6 (159 mg, 0.6 mmol, 3.0 equiv) were added successively. The vial was sealed with a screw-top septum cap, removed from the glovebox and placed in a heating block that was pre-heated to 50 ℃ with vigorous stirring for 24 h under N_2 atmosphere. After been cooled to room temperature, the reaction mixture was filtered through a pad of celite and concentrated in vacuo. The resulting residue was purified by silica gel flash chromatography (PE/DCM = 10/1) to give the desired product **6da** (44 mg, 58% yield, colorless viscous liquid). **R*f*** (PE/DCM = 5/1): 0.4. **^{1}H NMR (400 MHz, CDCl$_3$)** δ 8.86 (dd, *J* = 4.2, 1.9 Hz, 1H), 8.11 (dd, *J* = 8.3, 1.9 Hz, 1H), 7.78 (m, 2H), 7.72 (d, *J* = 6.8 Hz, 1H), 7.63 (dd, *J* = 6.8, 1.5 Hz, 1H), 7.44 – 7.37 (m, 2H), 7.33 (dd, *J* = 8.1, 4.4 Hz, 2H), 3.92 (hept, *J* = 6.0 Hz, 1H), 0.88 (m, 12H), 0.31 (s, 6H). **^{13}C NMR (101 MHz, CDCl$_3$)** δ 152.6, 149.0, 145.7, 145.4, 142.2, 137.8, 136.4, 136.0, 135.0, 128.9, 128.2, 127.8, 127.6, 125.9, 120.6, 65.3, 25.4, 1.7, 1.6. **HRMS (ESI)** m/z Calcd. for $C_{22}H_{29}NNaOSi_2$ $[M+Na]^+$ 402.1680, Found 402.1704.

Nickel(0)-Catalyzed Bissilylation of Alkenes

General procedure: In the nitrogen-filled glovebox, to an oven-dried 8-mL sealed tube equipped with a Teflon-coated magnetic stir bar were added Ni(COD)$_2$ (5.6 mg, 0.02 mmol, 10.0 mol%), SIPr (9.5 mg, 0.024 mmol, 12.0 mol%), toluene (2 mL), and the reaction mixture was stirred for 30 min, then disilane reagent **1** (0.2 mmol, 1.0 equiv) and specific alkene **7** (0.4–2 mmol, 2.0–10 equiv) were added. The vial was sealed with a screw-top septum cap, removed from the glovebox and placed in a heating block that was pre-heated to 100 ℃ with vigorous stirring for 48 h under N$_2$ atmosphere. After been cooled to room temperature, the reaction mixture was filtered through a pad of celite and concentrated in vacuo. The resulting residue was purified by reversed phase C18(ODS) column (5μm, 21.2×250 mm) with MeCN to give the desired product **8**. The mobile phase flow rate was 10 mL/min, and the detection was at 254 nm.

8-(Dimethyl(1-phenyl-2-(trimethylsilyl)ethyl)silyl)quinoline (8aa) and **8-(dimethyl(2-phenyl-2-(trimethylsilyl)ethyl)silyl)quinoline (8aa′)**

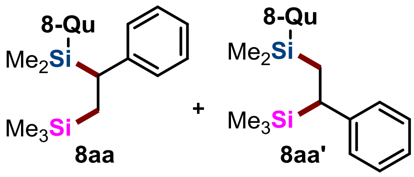

8aa: 8aa' = 86:14

Following the general procedure, in the nitrogen-filled glovebox, to an oven-dried 8-mL sealed tube equipped with a Teflon-coated magnetic stir bar were added Ni(COD)$_2$ (5.6 mg, 0.02 mmol, 10.0 mol%), SIPr (9.5 mg, 0.024 mmol, 12.0 mol%), toluene (2 mL), and the reaction mixture was stirred for 30 min, then disilane reagent **1a** (52 mg, 0.2 mmol, 1.0 equiv), styrene **7a** (41 mg, 0.4 mmol, 2.0 equiv) were added. The vial was sealed with a screw-top septum cap, removed from the glovebox and placed in a heating block that was pre-heated to 100 ℃ with vigorous stirring for 48 h under N$_2$ atmosphere. The reaction mixture is cooled to room temperature and the regioselectivity was determined by GC analysis (r.r. = 86:14). After removal of the solvent, the residue was purified by reversed phase C18(ODS) column (5μm, 21.2×250 mm) with MeCN to afford **8aa** and **8aa′** (72 mg, 85% yield, colorless viscous liquid). **R*f*** (PE): 0.6. **Major product (8aa)**: **^{1}H NMR (400 MHz, CDCl$_3$)** δ 8.98 (dd, *J* = 4.2, 1.8 Hz, 1H), 8.13 (dd, *J* = 8.2, 1.9 Hz, 1H), 7.79

(ddd, J = 15.7, 7.4, 1.5 Hz, 2H), 7.47 (dd, J = 8.1, 6.8 Hz, 1H), 7.40 (dd, J = 8.2, 4.1 Hz, 1H), 7.14 (t, J = 7.5 Hz, 2H), 7.09 – 6.99 (m, 3H), 3.25 (dd, J = 13.2, 2.2 Hz, 1H), 1.11 (dd, J = 15.0, 13.1 Hz, 1H), 0.74 (dd, J = 15.0, 2.2 Hz, 1H), 0.44 (s, 3H), 0.27 (s, 3H), -0.33 (s, 9H). **^{13}C NMR (101 MHz, CDCl$_3$)** δ 152.7, 149.0, 145.5, 140.1, 136.8, 136.2, 129.2, 128.2, 127.8, 127.7, 126.0, 124.0, 120.8, 30.1, 16.1, -1.1, -3.3, -4.8. **HRMS (ESI)** m/z Calcd. for $C_{22}H_{30}NSi_2$ [M+H]$^+$ 364.1911, Found 364.1909. **Minor product** (**8aa′**): **^{1}H NMR (400 MHz, CDCl$_3$)** δ 8.90 (dd, J = 4.1, 1.9 Hz, 1H), 8.10 (dd, J = 8.2, 1.9 Hz, 1H), 7.77 (dd, J = 8.1, 1.5 Hz, 1H), 7.65 (dd, J = 6.7, 1.5 Hz, 1H), 7.43 (dd, J = 8.1, 6.7 Hz, 1H), 7.36 (dd, J = 8.2, 4.2 Hz, 1H), 7.04 (t, J = 7.4 Hz, 2H), 6.97 – 6.91 (m, 1H), 6.88 – 6.83 (m, 2H), 2.02 (dd, J = 12.0, 3.2 Hz, 1H), 1.58 – 1.43 (m, 2H), 0.19 (d, J = 3.3 Hz, 6H), -0.17 (s, 9H). **^{13}C NMR (101 MHz, CDCl$_3$)** δ 152.7, 149.0, 145.3, 141.2, 136.1, 136.1, 129.0, 128.0, 127.7, 127.6, 126.0, 124.0, 120.7, 31.9, 15.4, -1.0, -1.7, -3.2. **HRMS (ESI)** m/z Calcd. for $C_{22}H_{30}NSi_2$ [M+H]$^+$ 364.1911, Found 364.1910.

8-(Dimethyl(1-(*p*-tolyl)-2-(trimethylsilyl)ethyl)silyl)quinoline (8ab) and **8-(dimethyl(2-(*p*-tolyl)-2-(trimethylsilyl)ethyl)silyl)quinoline (8ab′)**

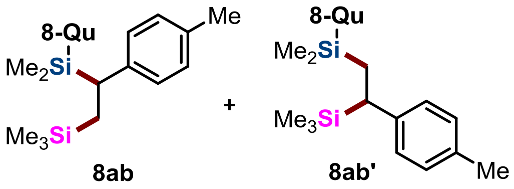

8ab: 8ab' = 85:15

Following the general procedure, in the nitrogen-filled glovebox, to an oven-dried 8-mL sealed tube equipped with a Teflon-coated magnetic stir bar were added Ni(COD)$_2$ (5.6 mg, 0.02 mmol, 10.0 mol%), SIPr (9.5 mg, 0.024 mmol, 12.0 mol%), toluene (2 mL), and the reaction mixture was stirred for 30 min, then disilane reagent **1a** (52 mg, 0.2 mmol, 1.0 equiv), 1-methyl-4-vinylbenzene **7b** (48 mg, 0.4 mmol, 2.0 equiv) were added. The vial was sealed with a screw-top septum cap, removed from the glovebox and placed in a heating block that was pre-heated to 100 ℃ with vigorous stirring for 48 h under N_2 atmosphere. The reaction mixture is cooled to room temperature and the regioselectivity was determined by GC analysis (r.r. = 85:15). After removal of the solvent, the residue was purified by reversed phase C18(ODS) column (5μm, 21.2×250 mm) with MeCN to afford **8ab** and **8ab′** (67 mg, 89% yield) as

colorless viscous liquid. **R$_f$** (PE/DCM = 20/1): 0.4. **Major product** (**8ab**): **^{1}H NMR (400 MHz, CDCl$_3$)** δ 8.98 (dd, *J* = 4.2, 1.8 Hz, 1H), 8.13 (dd, *J* = 8.3, 1.8 Hz, 1H), 7.80 (td, *J* = 7.7, 7.1, 1.5 Hz, 2H), 7.48 (dd, *J* = 8.0, 6.8 Hz, 1H), 7.39 (dd, *J* = 8.3, 4.1 Hz, 1H), 6.97 (s, 4H), 3.22 (dd, *J* = 13.1, 2.2 Hz, 1H), 2.28 (s, 3H), 1.08 (dd, *J* = 15.1, 13.1 Hz, 1H), 0.71 (dd, *J* = 15.0, 2.2 Hz, 1H), 0.43 (s, 3H), 0.27 (s, 3H), -0.33 (s, 9H). **^{13}C NMR (101 MHz, CDCl$_3$)** δ 152.7, 149.0, 142.2, 140.3, 136.8, 136.1, 133.2, 129.1, 128.4, 128.1, 127.7, 126.0, 120.7, 29.5, 21.1, 16.1, -1.0, -3.1, -4.9. **HRMS (ESI)** m/z Calcd. for $C_{23}H_{32}NSi_2$ $[M+H]^+$ 378.2068, Found 378.2066. **Minor product** (**8ab′**): **^{1}H NMR (400 MHz, CDCl$_3$)** δ 8.90 (dd, *J* = 4.2, 1.8 Hz, 1H), 8.09 (dd, *J* = 8.3, 1.9 Hz, 1H), 7.76 (dd, *J* = 8.1, 1.5 Hz, 1H), 7.64 (dd, *J* = 6.8, 1.5 Hz, 1H), 7.41 (dd, *J* = 8.1, 6.8 Hz, 1H), 7.35 (dd, *J* = 8.2, 4.2 Hz, 1H), 6.81 (d, *J* = 7.8 Hz, 2H), 6.72 (d, *J* = 8.1 Hz, 2H), 2.22 (s, 3H), 1.98 (dd, *J* = 11.0, 4.2 Hz, 1H), 1.51 – 1.45 (m, 2H), 0.21 (s, 6H), -0.17 (s, 9H). **^{13}C NMR (101 MHz, CDCl$_3$)** δ 152.7, 148.9, 142.0, 141.3, 136.1, 136.1, 133.1, 128.8, 128.2, 127.9, 127.7, 126.0 120.6, 31.3, 21.0, 15.5, -1.0, -1.7, -3.2. **HRMS (ESI)** m/z Calcd. for $C_{23}H_{32}NSi_2$ $[M+H]^+$ 378.2068, Found 378.2066.

8-(Dimethyl(1-(*m*-tolyl)-2-(trimethylsilyl)ethyl)silyl)quinoline (8ac) and **8-(dimethyl(2-(*m*-tolyl)-2-(trimethylsilyl)ethyl)silyl)quinoline (8ac′)**

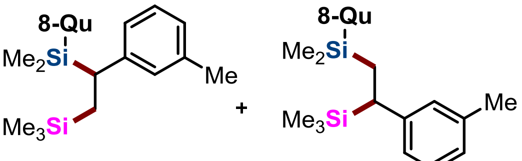

8ac **8ac'**

8ac: 8ac' = 83:17

Following the general procedure, in the nitrogen-filled glovebox, to an oven-dried 8-mL sealed tube equipped with a Teflon-coated magnetic stir bar were added Ni(COD)$_2$ (5.6 mg, 0.02 mmol, 10.0 mol%), SIPr (9.5 mg, 0.024 mmol, 12.0 mol%), toluene (2 mL), and the reaction mixture was stirred for 30 min, then disilane reagent **1a** (52 mg, 0.2 mmol, 1.0 equiv), 1-methyl-3-vinylbenzene **7c** (48 mg, 0.4 mmol, 2.0 equiv) were added. The vial was sealed with a screw-top septum cap, removed from the glovebox and placed in a heating block that was pre-heated to 100 ℃ with vigorous stirring for 48 h under N_2 atmosphere. The reaction mixture is cooled to room temperature and the regioselectivity was determined by GC analysis (r.r. = 83:17). After removal of the solvent, the residue was purified by reversed phase C18(ODS) column

(5μm, 21.2×250 mm) with MeCN to afford **8ac** and **8ac′** (64 mg, 85% yield) as colorless viscous liquid. **R*f*** (PE/DCM = 20/1): 0.4. **Major product** (**8ac**): **^{1}H NMR (400 MHz, $CDCl_3$)** δ 8.98 (dd, *J* = 4.2, 1.8 Hz, 1H), 8.14 (dd, *J* = 8.2, 1.9 Hz, 1H), 7.81 (dd, *J* = 8.1, 1.5 Hz, 1H), 7.78 (dd, *J* = 6.8, 1.5 Hz, 1H), 7.48 (dd, *J* = 8.1, 6.7 Hz, 1H), 7.40 (dd, *J* = 8.2, 4.1 Hz, 1H), 7.04 (t, *J* = 7.8 Hz, 1H), 6.90 – 6.80 (m, 3H), 3.21 (dd, *J* = 13.1, 2.3 Hz, 1H), 2.24 (s, 3H), 1.11 (dd, *J* = 15.0, 13.1 Hz, 1H), 0.73 (dd, *J* = 15.0, 2.3 Hz, 1H), 0.45 (s, 3H), 0.27 (s, 3H), -0.33 (s, 9H). **^{13}C NMR (101 MHz, $CDCl_3$)** δ 152.7, 149.0, 145.3, 140.3, 136.83, 136.81, 136.1, 129.1, 127.8, 127.5, 126.0, 125.3, 124.8, 120.7, 29.9, 21.6, 16.1, -1.0, -3.2, -4.8. **HRMS (ESI)** m/z Calcd. for $C_{23}H_{32}NSi_2$ $[M+H]^+$ 378.2068, Found 378.2066. **Minor product** (**8ac′**): **^{1}H NMR (400 MHz, $CDCl_3$)** δ 8.90 (dd, *J* = 4.2, 1.9 Hz, 1H), 8.10 (dd, *J* = 8.3, 1.9 Hz, 1H), 7.76 (dd, *J* = 8.1, 1.5 Hz, 1H), 7.63 (dd, *J* = 6.7, 1.6 Hz, 1H), 7.42 (dd, *J* = 8.1, 6.7 Hz, 1H), 7.35 (dd, *J* = 8.3, 4.2 Hz, 1H), 6.92 (t, *J* = 7.5 Hz, 1H), 6.73 (d, *J* = 7.5 Hz, 1H), 6.65 (d, *J* = 7.7 Hz, 1H), 6.59 (s, 1H), 2.13 (s, 3H), 1.98 (dd, *J* = 8.8, 6.5 Hz, 1H), 1.54 – 1.47 (m, 2H), 0.21 (d, *J* = 3.9 Hz, 6H), -0.16 (s, 9H). **^{13}C NMR (101 MHz, $CDCl_3$)** δ 152.7, 149.0, 145.1, 141.4, 136.8, 136.10, 136.08, 128.9, 128.8, 127.7, 127.3, 125.9, 125.2, 124.7, 120.6, 31.7, 29.8, 21.6, 15.4, -1.1, -1.6, -3.2. **HRMS (ESI)** m/z Calcd. for $C_{23}H_{32}NSi_2$ $[M+H]^+$ 378.2068, Found 378.2068.

8-(Dimethyl(1-(*o*-tolyl)-2-(trimethylsilyl)ethyl)silyl)quinoline (8ad) and **8-(dimethyl(2-(*o*-tolyl)-2-(trimethylsilyl)ethyl)silyl)quinoline (8ad′)**

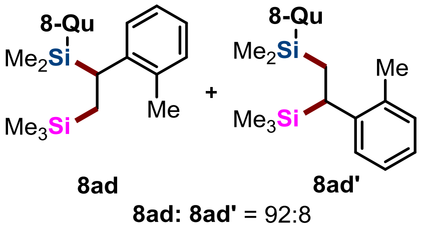

Following the general procedure, in the nitrogen-filled glovebox, to an oven-dried 8-mL sealed tube equipped with a Teflon-coated magnetic stir bar were added $Ni(COD)_2$ (5.6 mg, 0.02 mmol, 10.0 mol%), SIPr (9.5 mg, 0.024 mmol, 12.0 mol%), toluene (2 mL), and the reaction mixture was stirred for 30 min, then disilane reagent **1a** (52 mg, 0.2 mmol, 1.0 equiv), 1-methyl-2-vinylbenzene **7d** (48 mg, 0.4 mmol, 2.0 equiv) were added. The vial was sealed with a screw-top septum cap, removed from the glovebox and placed in a heating block that was pre-heated to 100 ℃ with vigorous stirring for 48 h under N_2 atmosphere.

The reaction mixture is cooled to room temperature and the regioselectivity was determined by GC analysis (r.r. = 92:8). After removal of the solvent, the residue was purified by reversed phase C18(ODS) column (5μm, 21.2×250 mm) with MeCN to afford the mixture of **8ad** and **8ad′** (62 mg, 82% yield) as a colorless viscous liquid. **R*f*** (PE/DCM = 20/1): 0.4. **^{1}H NMR (400 MHz, $CDCl_3$)** δ 8.96 (dd, *J* = 4.2, 1.8 Hz, 1H), 8.15 (dd, *J* = 8.3, 1.9 Hz, 1H), 7.89 (dd, *J* = 6.8, 1.5 Hz, 1H), 7.85 (dd, *J* = 8.2, 1.5 Hz, 1H), 7.54 (dd, *J* = 8.1, 6.7 Hz, 1H), 7.40 (dd, *J* = 8.2, 4.1 Hz, 1H), 7.22 – 7.09 (m, 3H), 7.01 (td, *J* = 7.2, 1.6 Hz, 1H), 3.57 (dd, *J* = 13.0, 2.3 Hz, 1H), 2.49 (s, 3H), 1.12 (dd, *J* = 15.0, 12.9 Hz, 1H), 0.66 (dd, *J* = 15.1, 2.3 Hz, 1H), 0.49 (s, 3H), 0.19 (s, 3H), -0.12 (s, 1H), -0.43 (s, 9H). **^{13}C NMR (101 MHz, $CDCl_3$)** δ 152.7, 148.8, 144.2, 140.3, 136.9, 136.2, 136.2, 135.8, 130.0, 129.2, 127.8, 127.2, 126.07, 126.02, 125.4, 123.8, 120.8, 120.7, 24.2, 20.8, 17.2, -1.2, -2.7, -3.1, -5.8. **HRMS (ESI)** m/z Calcd. for $C_{23}H_{32}NSi_2$ $[M+H]^+$ 378.2068, Found 378.2066.

8-(Dimethyl(1-(trimethylsilyl)hexan-2-yl)silyl)quinoline (8ae) and **8-(dimethyl(2-(trimethylsilyl)hexyl)silyl)quinoline (8ae′)**

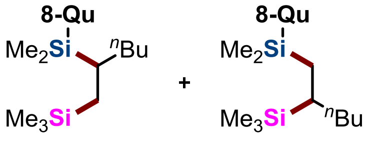

8ae **8ae'**
8ae: 8ae' = 84:16

Following the general procedure, in the nitrogen-filled glovebox, to an oven-dried 8-mL sealed tube equipped with a Teflon-coated magnetic stir bar were added $Ni(COD)_2$ (5.6 mg, 0.02 mmol, 10.0 mol%), SIPr (9.5 mg, 0.024 mmol, 12.0 mol%), toluene (2 mL), and the reaction mixture was stirred for 30 min, then disilane reagent **1a** (52 mg, 0.2 mmol, 1.0 equiv), 1-hexene **7e** (168 mg, 2.0 mmol, 10 equiv) were added. The vial was sealed with a screw-top septum cap, removed from the glovebox and placed in a heating block that was pre-heated to 100 ℃ with vigorous stirring for 48 h under N_2 atmosphere. The reaction mixture is cooled to room temperature and the regioselectivity was determined by GC analysis (r.r. = 84:16). After removal of the solvent, the residue was purified by reversed phase C18(ODS) column (5μm, 21.2×250 mm) with MeCN to afford the mixture of **8ae** and **8ae′** (59 mg, 86% yield) as a colorless viscous liquid. **R*f*** (PE): 0.4. **^{1}H NMR (400 MHz, $CDCl_3$)** δ 8.90 (dd, *J* = 4.2, 1.9 Hz, 1H), 8.10 (dd, *J* = 8.2, 1.9 Hz, 1H), 7.87 (dd, *J* = 6.7, 1.4 Hz,

1H), 7.80 (dd, J = 8.2, 1.3 Hz, 1H), 7.55 – 7.46 (m, 1H), 7.35 (dt, J = 8.2, 3.7 Hz, 1H), 1.59 (dq, J = 9.6, 5.8, 4.2 Hz, 1H), 1.46 (dd, J = 14.2, 7.0 Hz, 1H), 1.35 – 1.23 (m, 2H), 1.14 (dt, J = 6.9, 3.6 Hz, 4H), 1.03 – 0.94 (m, 1H), 0.71 (dd, J = 8.7, 5.8 Hz, 4H), 0.48 (s, 1H), 0.43 (d, J = 9.8 Hz, 6H), -0.10 (d, J = 16.3 Hz, 10H). **^{13}C NMR (101 MHz, $CDCl_3$)** δ 152.8, 149.0, 148.9, 141.7, 136.4, 136.1, 136.0, 129.0, 128.9, 127.7, 126.1, 126.0, 120.7, 33.5, 32.6, 31.6, 31.3, 23.4, 23.3, 20.6, 19.3, 16.9, 15.6, 14.1, -0.7, -1.0, -1.1, -2.3, -3.0, -3.1. **HRMS (ESI)** m/z Calcd. for $C_{20}H_{34}NSi_2$ $[M+H]^+$ 344.2224, Found 344.2223.

8-((1,2-Diphenyl-2-(trimethylsilyl)ethyl)dimethylsilyl)quinoline (8af)

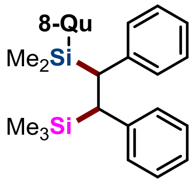

Following the general procedure, in the nitrogen-filled glovebox, to an oven-dried 8-mL sealed tube equipped with a Teflon-coated magnetic stir bar were added $Ni(COD)_2$ (5.6 mg, 0.02 mmol, 10.0 mol%), SIPr (9.5 mg, 0.024 mmol, 12.0 mol%), toluene (2 mL), and the reaction mixture was stirred for 30 min, then disilane reagent **1a** (52 mg, 0.2 mmol, 1.0 equiv), (*Z*)-1,2-diphenylethene **7f** (72 mg, 0.4 mmol, 2.0 equiv) were added. The vial was sealed with a screw-top septum cap, removed from the glovebox and placed in a heating block that was pre-heated to 100 ℃ with vigorous stirring for 48 h under N_2 atmosphere. After been cooled to room temperature, the reaction mixture was filtered through a pad of celite and concentrated in vacuo. The resulting residue was purified by silica gel flash chromatography (PE/DCM = 20/1) to give the desired product **8af** (72 mg, 82% yield, white solid). **R*f*** (PE/DCM = 20/1): 0.4. **^{1}H NMR (400 MHz, $CDCl_3$)** δ 9.05 (dd, J = 4.1, 1.8 Hz, 1H), 8.08 (dd, J = 8.2, 1.8 Hz, 1H), 7.59 (dd, J = 8.0, 1.5 Hz, 1H), 7.40 (dd, J = 8.2, 4.1 Hz, 1H), 7.32 (d, J = 7.3 Hz, 2H), 7.20 (t, J = 7.5 Hz, 2H), 7.15 – 7.01 (m, 3H), 6.99 – 6.38 (m, 5H), 3.90 (d, J = 13.5 Hz, 1H), 2.79 (d, J = 13.5 Hz, 1H), 0.25 (s, 3H), -0.03 (s, 3H), -0.48 (s, 9H). **^{13}C NMR (101 MHz, $CDCl_3$)** δ 152.1, 148.5, 144.6, 143.3, 141.6, 135.9, 135.1, 129.4, 129.0, 128.0, 127.9, 127.5, 127.1, 126.1, 124.8, 124.6, 120.5, 38.7, 36.9, -1.3, -1.4, -3.8. **HRMS (ESI)** m/z Calcd. for $C_{28}H_{34}NSi_2$ $[M+H]^+$ 440.2224, Found 440.2226.

Synthetic Transformations

Gram-scale experiments

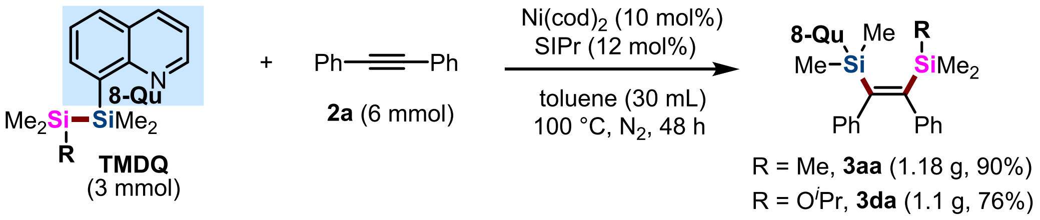

In the nitrogen-filled glovebox, to an oven-dried 100-mL sealed tube equipped with a Teflon-coated magnetic stir bar were added $Ni(COD)_2$ (83 mg, 0.3 mmol, 10.0 mol%), SIPr (141 mg, 0.36 mmol, 12.0 mol%), toluene (30 mL), and the reaction mixture was stirred for 30 min, then the disliane reagent **1a** or **1d** (3 mmol, 1.0 equiv), diphenylacetylene **2a** (6 mmol, 2.0 equiv) were added. The vial was sealed with a screw-top septum cap, removed from the glovebox and placed in an oil bath that was pre-heated to 100 ℃ with vigorous stirring for 48 h under N_2 atmosphere. After been cooled to room temperature, the reaction mixture was filtered through a pad of celite and concentrated in vacuo. The resulting residue was purified by silica gel flash chromatography to give the desired product **3aa** (PE/DCM = 20/1, 1.18 g, 90%) and **3da** (PE/DCM = 5/1, 1.1 g, 76%).

Synthetic utilities

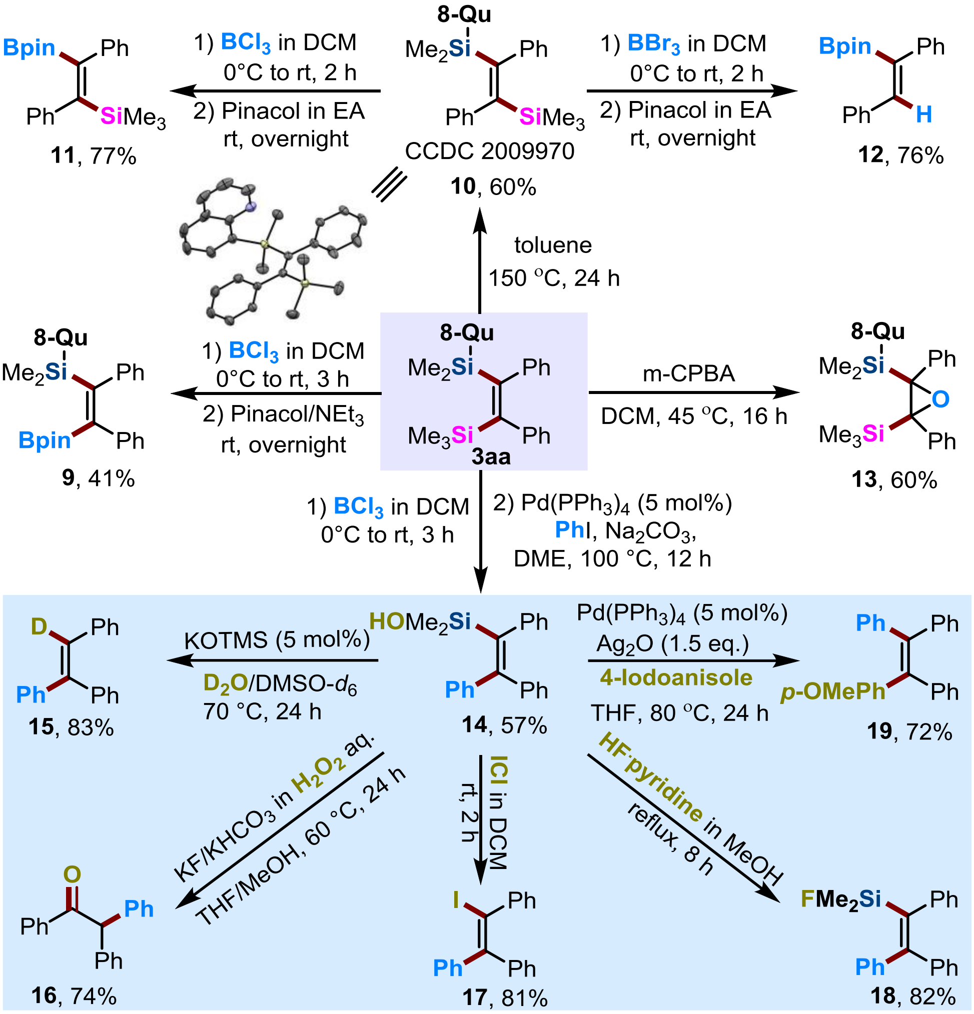

(*E*)-8-((1,2-Diphenyl-2-(4,4,5,5-tetramethyl-1,3,2-dioxaborolan-2-yl)vinyl)dimethylsilyl)quinoline (9)

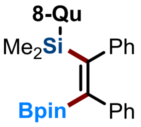

Following a modified literature procedure,[4] in flame-dried Schlenk tube equipped with a magnetic stirring bar, a nitrogen inlet, and a teflon cap was placed (*Z*)-8-((1,2-diphenyl-2-(trimethylsilyl)vinyl)dimethylsilyl)-quinoline **3aa** (87.4 mg, 0.2 mol) and dry dichloromethane (1 mL). This solution was cooled to 0 ℃ and BCl_3 (1.0 M solution in CH_2Cl_2, 0.24 mL, 0.24 mmol, 1.2 equiv) was added dropwise. The resulting mixture was stirred at room temperature for 3 h before pinacol (36 g, 0.3 mmol, 1.5 equiv) and Et_3N (56 μL, 0.4 mmol, 2 equiv) was added. After stirring the mixture at room temperature (25 ℃) overnight, saturated aqueous Na_2CO_3 (ca. 8 mL) was added. The aqueous phase was extracted with Et_2O (3

x 20 mL) and the combined extracts were dried over Na_2SO_4. Evaporation of the solvents and purification by column chromatography (silica gel, pentane/DCM = 10/1) afforded **9** (40 mg, 41% yield) as a white solid. **^{1}H NMR (400 MHz, $CDCl_3$)** δ 8.85 (dd, *J* = 4.2, 1.9 Hz, 1H), 8.07 – 7.98 (m, 2H), 7.72 (dd, *J* = 8.1, 1.5 Hz, 1H), 7.45 (dd, *J* = 8.1, 6.7 Hz, 1H), 7.26 (dd, *J* = 8.2, 4.1 Hz, 1H), 6.94 – 6.81 (m, 10H), 0.81 (s, 12H), 0.43 (s, 6H). **^{13}C NMR (101 MHz, $CDCl_3$)** δ 158.2, 152.4, 148.8, 145.5, 143.6, 141.6, 137.2, 135.9, 129.6, 129.2, 129.0, 128.7, 127.7, 127.1, 127.0, 126.0, 125.1, 124.5, 120.7, 83.5, 24.7, 0.8. **HRMS (ESI)** m/z Calcd. for $C_{31}H_{35}BNO_2Si$ $[M+H]^+$ 492.2525, Found 492.2533.

(*E*)-8-((1,2-Diphenyl-2-(trimethylsilyl)vinyl)dimethylsilyl)quinoline (10)

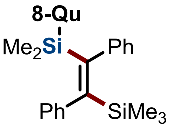

A 10 mL dried Schlenk tube equipped with a magnetic stir bar was charged with (*Z*)-8-((1,2-diphenyl-2-(trimethylsilyl)vinyl)dimethylsilyl)quinoline **3aa** (87.4 mg, 0.2 mol) and toluene (1 mL). Then, the tube was sealed with a Teflon plug under nitrogen atmosphere and stirred at 150 ℃ for 24 h. After the reaction mixture was cooled to room temperature, evaporation of the solvent and purification by column chromatography (silica gel, PE/DCM = 20/1) afforded **10** (52 mg, 60 %) as a white solid. **^{1}H NMR (400 MHz, $CDCl_3$)** δ 9.06 (dd, *J* = 4.1, 1.8 Hz, 1H), 8.12 (dd, *J* = 8.2, 1.8 Hz, 1H), 7.69 (dt, *J* = 6.9, 3.5 Hz, 1H), 7.46 – 7.33 (m, 5H), 7.26 (td, *J* = 6.5, 5.9, 3.6 Hz, 3H), 6.90 (t, *J* = 7.4 Hz, 1H), 6.79 (t, *J* = 7.5 Hz, 2H), 6.46 (d, *J* = 7.1 Hz, 2H), 0.22 (s, 6H), -0.34 (s, 9H). **^{13}C NMR (101 MHz, $CDCl_3$)** δ 158.9, 157.8, 152.0, 148.3, 146.3, 145.1, 141.3, 135.8, 135.3, 128.7, 128.1, 127.8, 127.4, 127.4, 126.7, 126.2, 125.6, 125.1, 120.6, 0.4, 0.0. **HRMS (ESI)** m/z Calcd. for $C_{28}H_{32}NSi_2$ $[M+H]^+$ 438.2068, Found 438.2070.

(*Z*)-(1,2-Diphenyl-2-(4,4,5,5-tetramethyl-1,3,2-dioxaborolan-2-yl)vinyl)trimethylsilane (11)

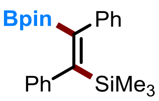

Following a modified literature procedure,[5] in flame-dried Schlenk tube equipped with a magnetic stirring bar, a nitrogen inlet, and a teflon cap was placed (*E*)-8-((1,2-diphenyl-2-(trimethylsilyl)vinyl)dimethylsilyl)quinoline **10** (87.4 mg, 0.2 mol) and dry dichloromethane (1 mL). This solution was cooled to 0 ℃

and BCl_3 (1.0 M solution in CH_2Cl_2, 0.24 mL, 0.24 mmol) was added dropwise. The resulting mixture was stirred at room temperature for 2 h until all starting material was consumed (TLC). After completion, the solvent was evaporated in vacuo, pinacol (48 mg, 2 equiv) and EtOAc (1 mL) were added and the solution stirred at room temperature (25 ℃) overnight. Evaporation of the solvent and purification by column chromatography (silica gel, PE/EA = 10/1) afforded **11** (58 mg, 77% yield) as a white solid. **^{1}H NMR (400 MHz, $CDCl_3$)** δ 7.49 – 7.28 (m, 8H), 7.24 (d, *J* = 1.7 Hz, 2H), 0.98 (s, 12H), -0.17 (s, 9H). **^{13}C NMR (101 MHz, $CDCl_3$)** δ 156.2, 146.1, 142.7, 128.3, 128.2, 128.0, 127.7, 126.6, 125.7, 83.5, 24.4, 0.4. **HRMS (EI)** calcd. For $C_{23}H_{31}BO_2Si$ $[M]^+$ 378.2186, Found 378.2177.

(*E*)-2-(1,2-Diphenylvinyl)-4,4,5,5-tetramethyl-1,3,2-dioxaborolane (12)

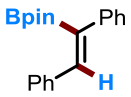

Following a modified literature procedure,[5] a CH_2Cl_2 solution of BBr_3 (23 μL, 60 mg, 0.24 mmol) was added by syringe to a stirred solution of (*E*)-8-((1,2-diphenyl-2-(trimethylsilyl)vinyl)dimethylsilyl)quinoline **10** (87.4 mg, 0.2 mol) in anhydrous CH_2Cl_2 (1 mL) at 0 ℃ under a nitrogen atmosphere. The resulting mixture was stirred at room temperature for 2 h until all starting material was consumed (TLC). After completion, the solvent was evaporated in vacuo, pinacol (48 mg, 2 equiv) and EtOAc (1 mL) were added and the solution stirred at room temperature (25 ℃) overnight. Evaporation of the solvents and purification by column chromatography (silica gel, PE/EA = 10/1) afforded **12** (47 mg, 76% yield) as a white solid. **^{1}H NMR (400 MHz, $CDCl_3$)** δ 7.24 (t, *J* = 6.9 Hz, 4H), 7.11 (q, *J* = 7.5 Hz, 4H), 7.07 – 7.00 (m, 3H), 1.08 (s, 12H). **^{13}C NMR (101 MHz, $CDCl_3$)** δ 142.8, 140.9, 138.9, 128.6, 128.4, 128.4, 128.2, 127.7, 127.1, 127.0, 84.2, 25.0. **HRMS (EI)** calcd. For $C_{20}H_{23}BO_2$ $[M]^+$ 306.1791, Found 306.1780.

8-((2,3-Diphenyl-3-(trimethylsilyl)oxiran-2-yl)dimethylsilyl)quinoline (**13**)

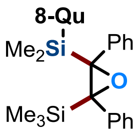

Following a modified literature procedure,[6] to a stirred solution of (*Z*)-8-((1,2-diphenyl-2-(trimethylsilyl)vinyl)dimethylsilyl)quinoline **3aa** (87.4 mg, 0.2 mol) in anhydrous CH_2Cl_2 (1 mL), *m*-CPBA (42 mg, 0.24 mol) was added. The solution was stirred at 45 ℃ for 16 h. After completion the reaction

mixture was filtered to remove the solids and solvent was diluted with water, and extracted with DCM. The separated organic layer was then washed with $NaHCO_3$ solution and dried over anhydrous Na_2SO_4. Subsequently, organic layer was concentrated in vacuo and the crude residue was purified by flash silica gel column chromatography (PE/DCM = 10/1) to afford **13** (54 mg, 60%) as a white solid. **^{1}H NMR (400 MHz, $CDCl_3$)** δ 9.18 (dd, *J* = 4.2, 1.8 Hz, 1H), 8.22 (dd, *J* = 8.3, 1.7 Hz, 1H), 8.00 (dd, *J* = 6.7, 1.4 Hz, 1H), 7.90 (dd, *J* = 8.2, 1.4 Hz, 1H), 7.72 – 7.66 (m, 1H), 7.58 (dd, *J* = 8.1, 6.8 Hz, 1H), 7.54 (dd, *J* = 8.2, 4.1 Hz, 1H), 7.28 (d, *J* = 7.8 Hz, 1H), 7.07 – 6.97 (m, 2H), 6.96 – 6.83 (m, 6H), 0.66 (s, 3H), 0.03 (s, 3H), -0.52 (s, 9H). **^{13}C NMR (101 MHz, $CDCl_3$)** δ 152.3, 148.9, 143.2, 142.3, 140.3, 137.0, 136.5, 129.7, 128.9, 128.0, 127.40 127.1, 126.9, 126.5, 126.3, 126.3, 126.2, 125.1, 125.1, 121.4, 68.7, 68.3, 1.6, -1.2, -3.3. **HRMS (ESI)** m/z Calcd. for $C_{28}H_{32}NOSi_2$ $[M+H]^+$ 454.2017, Found 454.2016.

Dimethyl(1,2,2-triphenylvinyl)silanol (14)

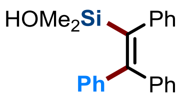

Following a modified literature procedure,[4] in flame-dried Schlenk tube equipped with a magnetic stirring bar, an nitrogen inlet, and a teflon cap was placed (*Z*)-8-((1,2-diphenyl-2-(trimethylsilyl)vinyl)dimethylsilyl)-quinoline **3aa** (87.4 mg, 0.2 mol) and dry dichloromethane (1 mL). This solution was cooled to 0 ℃ and BCl_3 (1.0 M solution in CH_2Cl_2, 0.24 mL, 0.24 mmol, 1.2 equiv) was added dropwise and the resulting mixture was stirred at room temperature for 3 h. The solvent was evaporated at reduced pressure and the residue was dissolved in DME (2 mL), then $Pd(PPh_3)_4$ (12 mg, 0.01 mmol, 5 mol %), iodobenzene (81.6 mg, 0.4 mmol, 2.0 equiv) was added followed by Na_2CO_3 (2.0 M solution in water, 0.5 mL, 1.0 mmol, 5.0 equiv). The tube was sealed with a Teflon plug under nitrogen atmosphere and stirred at 100 ℃ for overnight. After cooling the reaction mixture to room temperature water (ca. 4.0 mL) was added. The aqueous phase was extracted with Et_2O (3 x 10 mL) and the combined organic phases were dried over Na_2SO_4. Evaporation of the solvents and purification by column chromatography (silica gel, PE/EA = 20:1) afforded **14** (38 mg, 57 %) as a white solid. **^{1}H NMR (400 MHz, $CDCl_3$)** δ 7.38 (s, 5H), 7.17 (t, *J* = 6.6

Hz, 2H), 7.12 – 6.90 (m, 8H), 0.01 (s, 6H). **^{13}C NMR (101 MHz, $CDCl_3$)** δ 153.7, 144.1, 144.0, 142.9, 142.5, 129.6, 129.5, 129.4, 128.5, 128.0, 127.8, 127.5, 126.5, 125.6, 1.3. **HRMS (EI)** calcd. For $C_{22}H_{22}OSi$ $[M]^+$: 330.1440, Found 330.1434.

(Ethene-1,1,2-triyl-2-d)tribenzene (15)

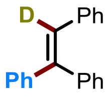

Following a modified literature procedure,[7] a 10 mL dried Schlenk tube equipped with a magnetic stir bar was charged with the KOTMS (1.3 mg, 0.01 mmol, 5 mol %), dimethyl(1,2,2-triphenylvinyl)silanol **14** (66 mg, 0.2 mmol), D_2O (12 μL, 12 mg, 3.0 equiv), and anhydrous DMSO-d_6 (0.5 mL). Then, the tube was sealed with a Teflon plug under nitrogen atmosphere and stirred at 70 ℃ for 6 h. After that, the reaction mixture was cooled to room temperature and determined by GC and GC-MS analysis, which can be successfully converted to **15** in 83% yield. **^{1}H NMR (400 MHz, DMSO-d_6)** δ 7.43 – 7.24 (m, 8H), 7.17 – 7.07 (m, 5H), 7.00 (d, J = 7.0 Hz, 2H). **^{13}C NMR (101 MHz, DMSO-d_6)** δ 142.5, 141.8, 141.7, 139.9, 136.9, 136.8, 129.7, 129.3, 128.9, 128.4, 128.0, 127.6, 127.04 126.9. Data is in accordance with literature.[8]

1,2,2-Triphenylethan-1-one (16)

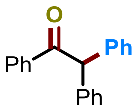

Following a modified literature procedure,[9] to a mixture of KF (0.4 mmol) and $KHCO_3$ (0.4 mmol) in MeOH (0.4 mL) and THF (0.4 mL) were added dimethyl(1,2,2-triphenylvinyl)silanol **14** (66 mg, 0.2 mmol), 30% aqueous H_2O_2 (4 mmol) and the reaction mixture was stirred at 60 ℃ for 24 h. After being cooled to room temperature, the reaction mixture was treated with H_2O (2 mL). The mixture was extracted with Et_2O (10 mL) and the combined organic phase was successively washed with 15% aqueous $Na_2S_2O_3$ (2 mL). Drying over Na_2SO_4 and removal of solvents under reduced pressure. The crude residue was purified by flash silica gel column chromatography (PE/EA = 50/1) to afford a white solid (**16**, 40 mg, 74%). **^{1}H NMR (400 MHz, $CDCl_3$)** δ 8.04 – 7.96 (m, 2H), 7.53 – 7.46 (m, 1H), 7.43 – 7.36 (m, 2H), 7.35 – 7.21 (m, 10H), 6.03 (s, 1H). **^{13}C NMR (101 MHz, $CDCl_3$)** δ 198.3, 139.2, 136.9, 133.2, 129.3, 129.1, 128.9, 128.7, 127.3, 59.6. Data is in accordance with the literature.[10]

(2-Iodoethene-1,1,2-triyl)tribenzene (**17** cas: 22021-09-6)

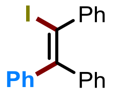

Following a modified literature procedure,[11] to a solution of dimethyl(1,2,2-triphenylvinyl)silanol **14** (66 mg, 0.2 mmol) in CH_2Cl_2 at room temperature (25 ℃) was added 1.0 M solution of ICl in CH_2Cl_2 (0.4 mL). The reaction mixture was stirred over 2 h under N_2 at room temperature (25 ℃). Saturated Na_2SO_3 solution was added and stirred over 1 hour. The layers were separated, and the aqueous layer was washed twice with CH_2Cl_2. The combined organic layer was dried over anhydrous Na_2SO_4, filtered and concentrated under reduced pressure. The crude residue was purified by flash silica gel column chromatography (PE) to afford **17** as a white solid (62 mg, 81%). **^{1}H NMR (400 MHz, $CDCl_3$)** δ 7.40 (d, *J* = 4.8 Hz, 4H), 7.38 – 7.30 (m, 3H), 7.25 – 7.16 (m, 3H), 7.12 – 7.11 (m, 3H), 702 – 6.98 (m, 2H). **^{13}C NMR (101 MHz, $CDCl_3$)** δ 142.0, 141.2, 140.5, 139.5, 130.7, 130.2, 130.1, 129.9, 128.3, 128.2, 128.1, 128.0, 127.7, 127.2. Data is in accordance with the literature.[12]

Fluorodimethyl(1,2,2-triphenylvinyl)silane (18)

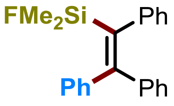

Following a modified literature procedure,[13] HF Pyridine (154 μL, 1.73 mmol, 10 equiv, 65%-70%) was then added to dimethyl(1,2,2-triphenyl-vinyl)silanol **14** (66 mg, 0.2 mmol) in MeOH (2 mL) in a sealed tube. The reaction was the heated at reflux. After 8 hours stirring at reflux, the reaction was cooled and the solvent removed under reduced pressure. The residue was purified by reversed phase C18(ODS) column (5μm, 21.2×250 mm) with H_2O/MeCN to afford **18** (54 mg, 82 %) as a white solid. **^{1}H NMR (400 MHz, $CDCl_3$)** δ 7.38 – 7.30 (m, 5H), 7.18 (t, *J* = 7.3 Hz, 2H), 7.12 – 7.06 (m, 3H), 7.02 (t, *J* = 5.8 Hz, 3H), 6.96 – 6.91 (m, 2H), -0.02 (s, 3H), -0.04 (s, 3H). **^{13}C NMR (101 MHz, $CDCl_3$)** δ156.1 (d, *J* = 3.0 Hz), 143.8, 142.3, 142.0, 141.3 (d, *J* = 14.4 Hz), 130.0, 129.9, 129.7 (d, *J* = 0.8 Hz), 128.29, 128.0, 127.9, 127.5, 126.7, 125.8, 0.3, 0.1. **^{19}F NMR (377 MHz, $CDCl_3$)** δ -153.79 (dt, *J* = 15.2, 7.5 Hz). **HRMS (EI)** calcd. For $C_{22}H_{21}FSi$ $[M]^+$ 332.1397, Found 332.1391.

(2-(4-Methoxyphenyl)ethene-1,1,2-triyl)tribenzene (19)

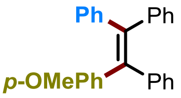

Procedure modified from literature[14]: To a solution of $Pd(PPh_3)_4$ (12 mg, 0.01 mmol, 5 mol %) in THF (1.0 mL) were added 4-Iodoanisole

(94 mg, 0.4 mmol), dimethyl(1,2,2-triphenylvinyl)silanol **14** (66 mg, 0.2 mmol), Ag_2O (69.5 mg, 0.30 mmol), and THF (1.0 mL). After the mixture was stirred at 80 ℃ for 24 h, catalyst and inorganic residue were removed by filtration through a short silica gel pad (EtOAc). The filtrate was washed with 1 N aq HCl (4 × 10 mL) and dried over Na_2SO_4. Removal of solvents under reduced pressure and subsequent silica gel chromatography (PE/EtOAc = 10/1) afforded **19** (52 mg, 72%) as white solid. **^{1}H NMR (400 MHz, $CDCl_3$)** δ 7.16 – 6.99 (m, 15H), 6.97 – 6.90 (m, 2H), 6.69 – 6.61 (m, 2H), 3.74 (s, 3H). **^{13}C NMR (101 MHz, $CDCl_3$)** δ 158.2, 144.2, 144.1, 144.1, 140.6, 140.2, 136.2, 132.7, 131.5, 131.5, 131.5, 127.8, 127.7, 126.5, 126.4, 126.4, 113.2, 55.2. Data is in accordance with the literature.[15]

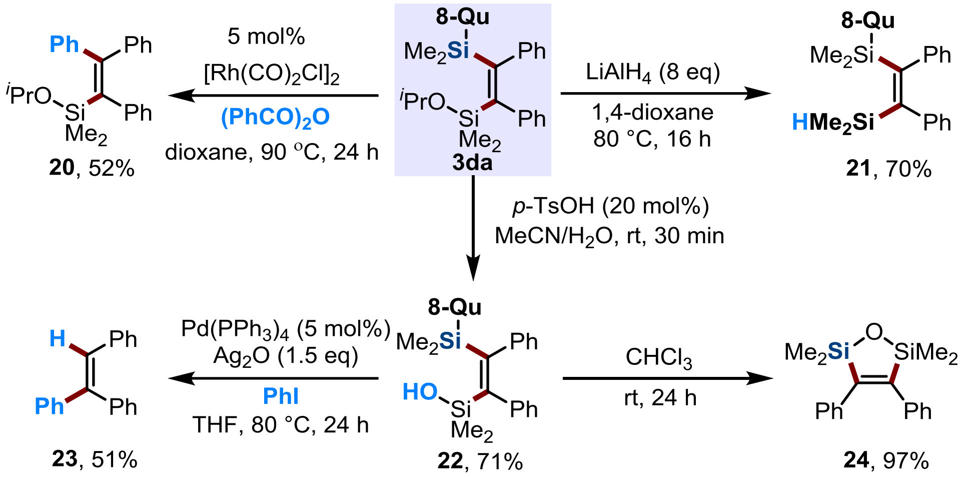

Isopropoxydimethyl(1,2,2-triphenylvinyl)silane (20)

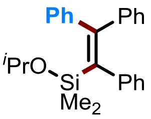

Following the modified literature procedure,[16] $[RhCl(CO)_2]_2$ (7.8 mg, 0.02 mmol, 10 mol%) and acetic anhydride (61.2 mg, 0.6 mmol) were added to a dioxane solution (1 mL) of (*Z*)-8-((2-(isopropoxydimethylsilyl)-1,2-diphenylvinyl)dimethylsilyl)quinoline **3da** (96.2 mg, 0.2 mmol) and the mixture was heated at 90 ℃ for 24 h. After evaporation of the solvent, the crude product was purified by silica gel chromatography (PE/EA = 20/1) to afford **20** (39 mg, 52%) as a white solid. **^{1}H NMR (400 MHz, $CDCl_3$)** δ 7.39 (dd, *J* = 7.9, 1.5 Hz, 2H), 7.37 – 7.27 (m, 3H), 7.12 (t, *J* = 7.4 Hz, 2H), 7.09 – 6.90 (m, 8H), 3.87 (hept, *J* = 6.0 Hz, 1H), 1.04 (d, *J* = 6.1 Hz, 6H), -0.17 (s, 6H). **^{13}C NMR (101 MHz, $CDCl_3$)** δ 154.8, 144.2, 143.8,

143.4, 143.1, 129.9, 129.8, 129.7, 128.0, 127.5 127.4, 126.2, 125.1, 65.3, 25.7, 0.7. **HRMS (EI)** calcd. For $C_{25}H_{28}OSi$ $[M]^+$ 372.1909, Found 372.1901.

(*Z*)-8-((2-(Dimethylsilyl)-1,2-diphenylvinyl)dimethylsilyl)quinoline (21)

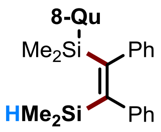

Following the modified literature procedure,[17] to a solution of (*Z*)-8-((2-(isopropoxydimethylsilyl)-1,2-diphenylvinyl)dimethylsilyl)quinoline **3da** (96.2 mg, 0.2 mmol) in 1,4-dioxane (1.0 mL) was added $LiAlH_4$ (61 mg, 1.6 mmol). After the mixture was stirred at 80 ℃ for 16 h, saturated NH_4Cl solution was added carefully at 0 ℃ and the mixture was extracted by Et_2O twice. The combined organic phase was dried over Na_2SO_4, filtered and concentrated. The crude products were purified by column chromatography (PE/EA = 20/1) to afford **21** (59 mg, 70% yield) as white solid. **^{1}H NMR (400 MHz, CDCl$_3$)** δ 8.99 (dd, *J* = 4.0, 1.6 Hz, 1H), 8.09 (dd, *J* = 8.2, 1.5 Hz, 1H), 7.66 (dd, *J* = 7.2, 2.3 Hz, 1H), 7.40 (dd, *J* = 8.2, 4.1 Hz, 1H), 7.35 – 7.29 (m, 4H), 7.25 – 7.19 (m, 3H), 6.87 (t, *J* = 7.3 Hz, 1H), 6.77 (t, *J* = 7.5 Hz, 2H), 6.42 (d, *J* = 7.4 Hz, 2H), 3.62 (p, *J* = 3.7 Hz, 1H), 0.17 (s, 6H), -0.29 (d, *J* = 3.7 Hz, 6H). **^{13}C NMR (101 MHz, CDCl$_3$)** δ 160.1, 156.0, 152.0, 148.4, 146.2, 144.1, 141.1, 135.8, 135.5, 128.5, 128.3, 127.8, 127.5, 127.4, 126.9, 126.2, 125.6, 125.3, 120.6, -0.1, -3.2. **HRMS (ESI)** m/z Calcd. for $C_{27}H_{30}NSi_2$ $[M+H]^+$ 424.1911, Found 424.1909.

(*Z*)-(2-(Dimethyl(quinolin-8-yl)silyl)-1,2-diphenylvinyl)dimethylsilanol (22)

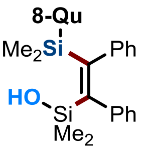

To a solution of (*Z*)-8-((2-(isopropoxydimethylsilyl)-1,2-diphenylvinyl)-dimethylsilyl)quinoline **3da** (96.2 mg, 0.2 mmol) in MeCN/H_2O (2.0 mL/0.2 mL) were added *p*-TsOH (7 mg, 0.04 mmol), and the mixture was stirred at room temperature for 30 min. After completion, the solvent was evaporated in vacuo and the crude product were purified by neutral alumina column chromatography (PE/EA = 10/1) to afford **22** (62 mg, 71%) as a white solid. **^{1}H NMR (400 MHz, CDCl$_3$)** δ 8.92 (dd, *J* = 4.3, 1.8 Hz, 1H), 8.22 (dd, *J* = 8.3, 1.8 Hz, 1H), 7.84 (dd, *J* = 8.1, 1.5 Hz, 1H), 7.66 (dd, *J* = 6.9, 1.5 Hz, 1H), 7.53 (s, 1H), 7.49 – 7.41 (m, 2H), 6.85 (t, *J* = 7.5 Hz, 2H), 6.79 – 6.70 (m, 4H), 6.55 – 6.49 (m, 2H), 6.21 (dd, *J* = 6.5, 3.0 Hz, 2H), 0.45 (s, 6H), -0.00 (s, 6H). **^{13}C NMR (101 MHz, CDCl$_3$)** δ 158.6, 156.2, 150.9, 149.1, 145.7, 145.1, 139.1, 138.1, 138.0, 129.8, 128.7, 128.4, 128.0, 127.2,

126.6, 126.6, 124.5, 124.5, 121.1, 1.9, 1.3. **HRMS (ESI)** m/z Calcd. for $C_{27}H_{30}NOSi_2$ $[M+H]^+$ 440.1860, Found 440.1863.

Ethene-1,1,2-triyltribenzene (23)

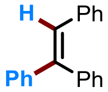

Following the modified literature procedure,[14] in flame-dried Schlenk tube equipped with a magnetic stirring bar, an nitrogen inlet, and a teflon cap was placed (*Z*)-(2-(dimethyl(quinolin-8-yl)silyl)-1,2-diphenylvinyl)dimethylsilanol **22** (87.8 mg, 0.2 mol) and THF (2 mL). Then $Pd(PPh_3)_4$ (12 mg, 0.01 mmol, 5 mol %), iodobenzene (81.6 mg, 0.4 mmol, 2.0 equiv), Ag_2O (69.5 mg, 0.30 mmol) was added. The resulting mixture was stirred at 80 ℃ for 24 h. Catalyst and inorganic residue were removed by filtration through a short silica gel pad (EtOAc). The filtrate was washed with 1 N aq HCl (4 × 10 mL) and dried over Na_2SO_4. Removal of solvents under reduced pressure and subsequent silica gel chromatography (PE/EA = 30:1) afforded **23** (26 mg, 51%) as white solid. **^{1}H NMR (400 MHz, $CDCl_3$)** δ 7.37 – 7.30 (m, 8H), 7.25 – 7.20 (m, 2H), 7.17 – 7.11 (m, 3H), 7.07 – 7.02 (m, 2H), 6.98 (s, 1H). **^{13}C NMR (101 MHz, $CDCl_3$)** δ 143.6, 142.7, 140.5, 137.5, 130.5, 129.7, 128.7, 128.3, 128.3, 128.1, 127.7, 127.6, 127.5, 126.9. Data is in accordance with the literature.[18]

2,2,5,5-Tetramethyl-3,4-diphenyl-2,5-dihydro-1,2,5-oxadisilole (24)

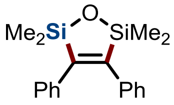

In 25 mL Schlenk tube equipped with a magnetic stirring bar, an nitrogen inlet, and a teflon cap was placed (*Z*)-(2-(dimethyl(quinolin-8-yl)silyl)-1,2-diphenylvinyl)dimethylsilanol **22** (87.8 mg, 0.2 mmol) and $CHCl_3$ (1 mL), then the mixture was stirred at room temperature for 24 h until all starting material was consumed (TLC). Evaporation of the solvents and purification by column chromatography (silica gel, PE/DCM = 20/1) afforded **24** (60 mg, 97%) as a white solid. **^{1}H NMR (400 MHz, $CDCl_3$)** δ 7.22 – 7.13 (m, 4H), 7.13 – 7.07 (m, 2H), 7.01 – 6.90 (m, 4H), 0.36 (s, 12H). **^{13}C NMR (101 MHz, $CDCl_3$)** δ 158.9, 140.8, 128.3, 127.8, 126.0, 0.7. **HRMS (EI)** calcd. For $C_{18}H_{22}OSi_2$ $[M]^+$ 310.1209, Found 310.1202.

Supplementary References

1. Ghaffari, B., Preshlock, S. M., Plattner, D. L., Staples, R. J., Maligres, P. E., Krska, S. W., Maleczka, Jr., R. E. & Smith, III, M. R. Silyl Phosphorus and Nitrogen Donor Chelates for Homogeneous Ortho Borylation Catalysis. *J. Am. Chem. Soc.* **136**, 14345–14348 (2014).
2. Tamao, K., Tsuji, H., Terada, M., Asahara, M., Yamaguchi, S. & Toshimitsu, A. Conformation Control of Oligosilanes Based on Configurationally Constrained Bicyclic Disilane Units. *Angew. Chem. Int. Ed.* **39**, 3287–3290 (2000).
3. Xiao, P., Cao, Y., Gui, Y., Gao, L. & Song, Z. $Me_3Si{-}SiMe_2[o\text{CON}(i\text{Pr})_2{-}C_6H_4]$: An Unsymmetrical Disilane Reagent for Regio- and Stereoselective Bis-Silylation of Alkynes. *Angew. Chem. Int. Ed.* **57**, 4769–4773 (2018).
4. Perrone, S. & Knochel, P. Highly Diastereoselective Preparation of (*E*)-Alkenylsilanes Bearing an α-Chiral Center. *Org. Lett.* **9**, 1041–1044 (2007).
5. Zhao, Z. & Snieckus, V. Directed ortho Metalation–based Methodology. Halo-, Nitroso-, and Boron-induced *ipso*-Desilylation. Link to an in situ Suzuki Reaction. *Org. Lett.* **7**, 2523–2526 (2005).
6. Penjarla, T. R., Kundarapu, M., Baque, S. M. & Bhattacharya, A. Synthesis of 4-Substituted Pyrrolo[2, 3-*c*]quinolines via Microwave-Assisted C-N Bond Formation *ChemistrySelect* **3**, 5386–5839 (2018).
7. Yao, W., Li, R., Jiang, H. & Han, D. An Additive-Free, Base-Catalyzed Protodesilylation of Organosilanes. *J. Org. Chem.* **83**, 2250–2255 (2018).
8. Zhang, W., Liu, M., Wu, H., Ding, J. & Chen, J. Phosphine-free rhodium-catalyzed hydroarylation of diaryl acetylenes with boronic acids. *Tetrahedron Letters* **49**, 5214–5216 (2008).
9. Itami, K., Nokami, T. & Yoshida, J. Unusually Accelerated Silylmethyl Transfer from Tin in Stille Coupling: Implication of Coordination-Driven Transmetalation. *J. Am. Chem. Soc.* **123**, 8773–8779 (2001).
10. Astarloa, I., SanMartin, R., Herrero, M. T. & Domíngueza, E. Aqueous α-Arylation

of Mono- and Diarylethanone Enolates at Low Catalyst Loading. *Adv. Synth. Catal.* **360**, 1711–1718 (2018).

11. Hua, Y., Asgari, P., Avullala, T. & Jeon, J. Catalytic Reductive ortho-C–H Silylation of Phenols with Traceless, Versatile Acetal Directing Groups and Synthetic Applications of Dioxasilines. *J. Am. Chem. Soc.* **138**, 7982–7991 (2016).
12. Iwasaki, M., Araki, Y., Iino, S. & Nishihara, Y. Synthesis of Multisubstituted Triphenylenes and Phenanthrenes by Cascade Reaction of *o*‑Iodobiphenyls or (*Z*)-β-Halostyrenes with *o*‑Bromobenzyl Alcohols through Two Sequential C−C Bond Formations Catalyzed by a Palladium Complex *J. Org. Chem.* **80**, 9247–9263 (2015).
13. Scroggie, K. R., Alcock, L. J., Matos, M. J., Bernardes, G. J. L., Perkins, M. V. & Chalker, J. M. A silicon-labelled amino acid suitable for late-stage fluorination and unexpected oxidative cleavage reactions in the preparation of a key intermediate in the Strecker synthesis. *Peptide Science*. 110:e24069 (2018).
14. Itami, K., Mineno, M., Kamei, T. & Yoshida, J. A General and Straightforward Route toward Diarylmethanes. Integrated Cross-Coupling Reactions Using (2-Pyridyl)silylmethylstannane as an Air-Stable, Storable, and Versatile Coupling Platform *Org. Lett.* **4**, 3635–3638 (2002).
15. Banerjee, M., Emond, S. J., Lindeman, S. V. & Rathore, R. Practical Synthesis of Unsymmetrical Tetraarylethylenes and Their Application for the Preparation of [Triphenylethylene-Spacer-Triphenylethylene] Triads. *J. Org. Chem.* **72**, 8054–8061 (2007).
16. Thiot, C., Mioskowski, C. & Wagner, A. Sequential Hiyama Coupling/Narasaka Acylation Reaction of (*E*)-1,2-Disilylethene: Rapid Assembly of α, β-Unsaturated Carbonyl Motifs. *Eur. J. Org. Chem*. **19**, 3219–3227 (2009).
17. Kuninobu, Y., Yamauchi, K., Tamura, N., Seiki, T. & Takai, K. Rhodium-Catalyzed Asymmetric Synthesis of Spirosilabifluorene Derivatives. *Angew. Chem. Int. Ed*. **52**, 1520–1522 (2013).
18. Li, B., Li, Y., Lu, X., Liu, J., Guan, B. & Shi, Z. Cross-Coupling of Aryl/Alkenyl

Pivalates with Organozinc Reagents via Nickel-Catalyzed C-O Activation under Mild Conditions. *Angew. Chem. Int. Ed.* **47**, 10124–10127 (2008).

NMR Spectra

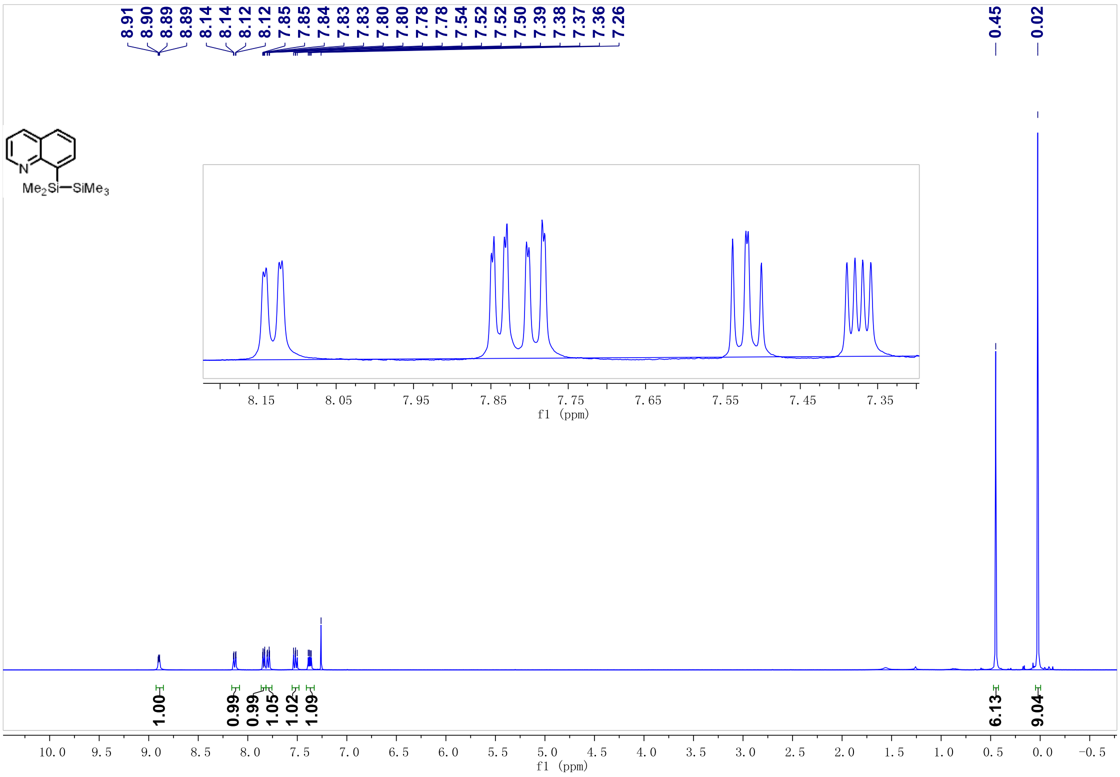

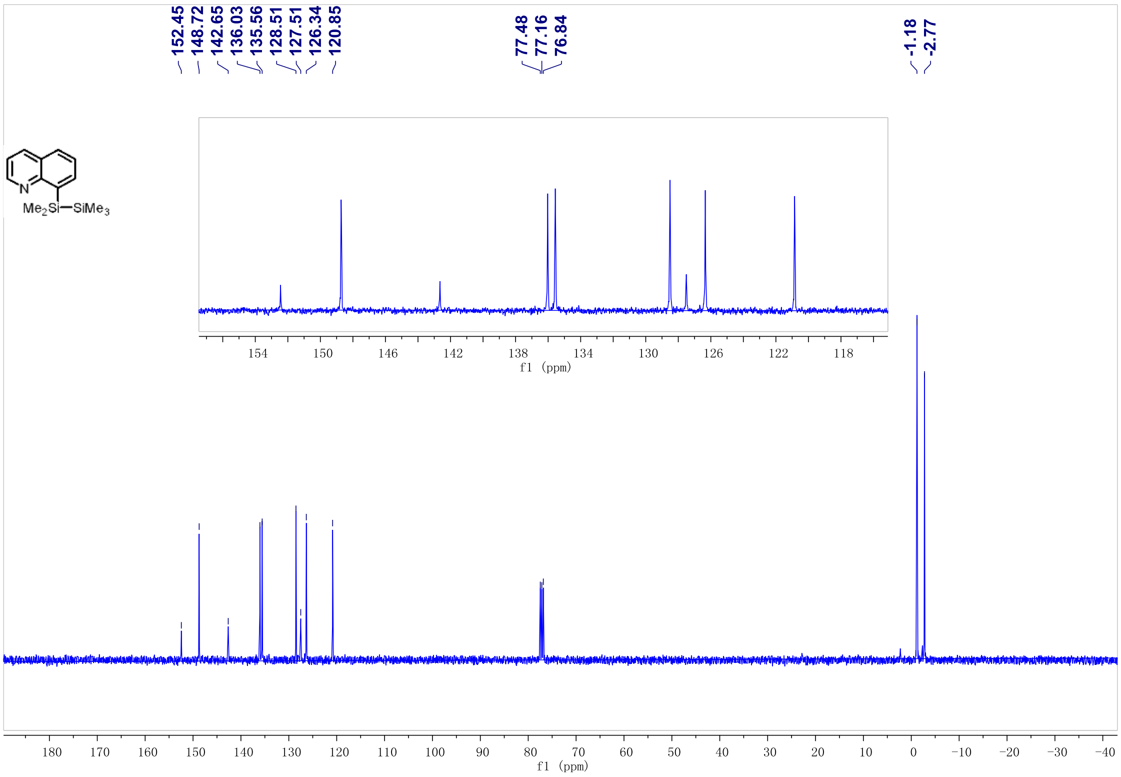

Supplementary Figure 1 ^{1}H and ^{13}C NMR Spectra for compound 1a

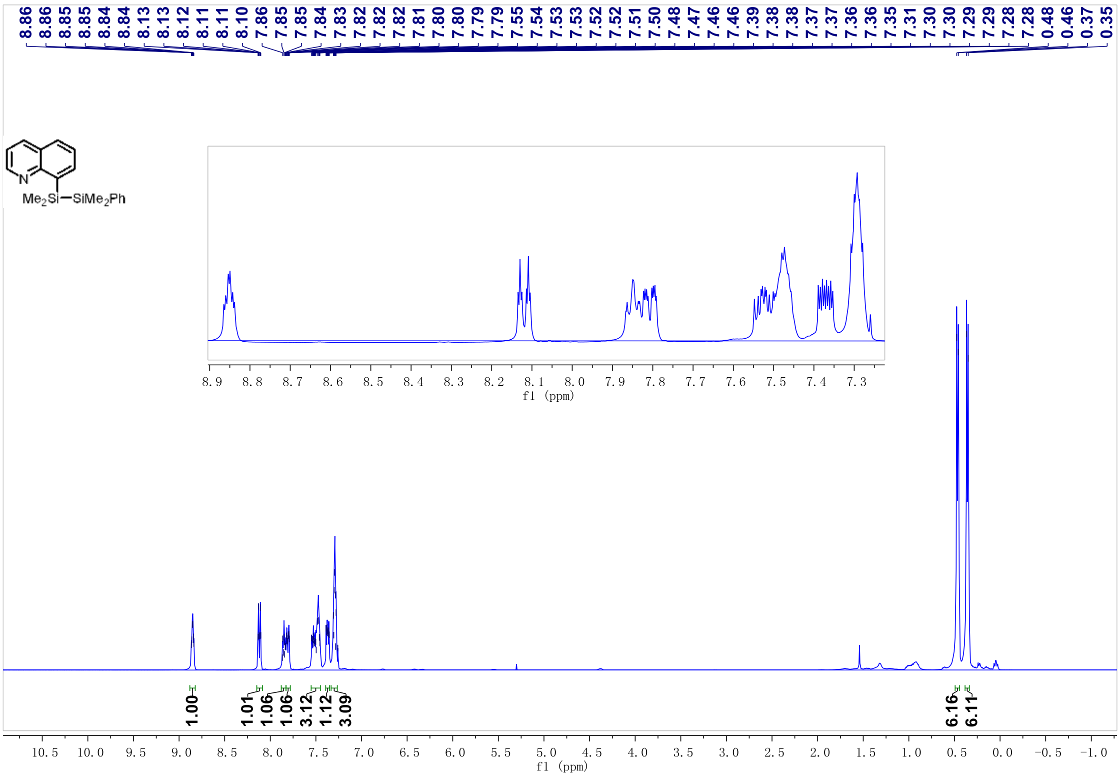

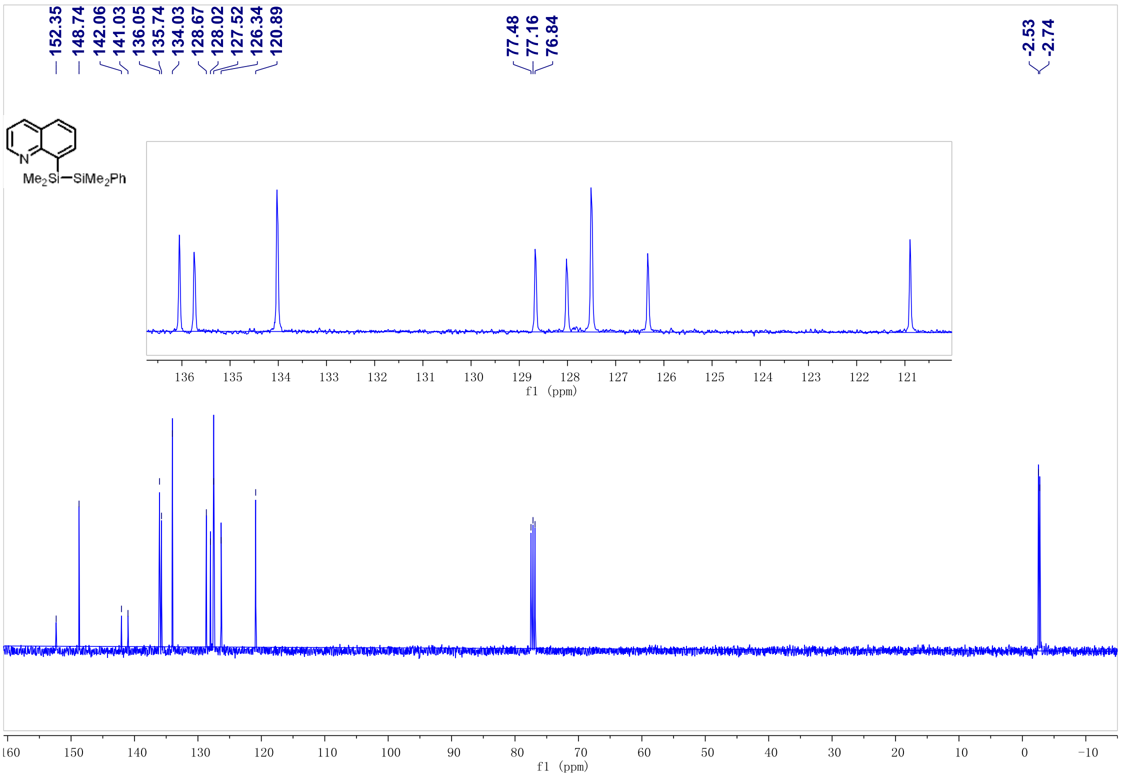

Supplementary Figure 2 ^{1}H and ^{13}C NMR Spectra for compound 1b

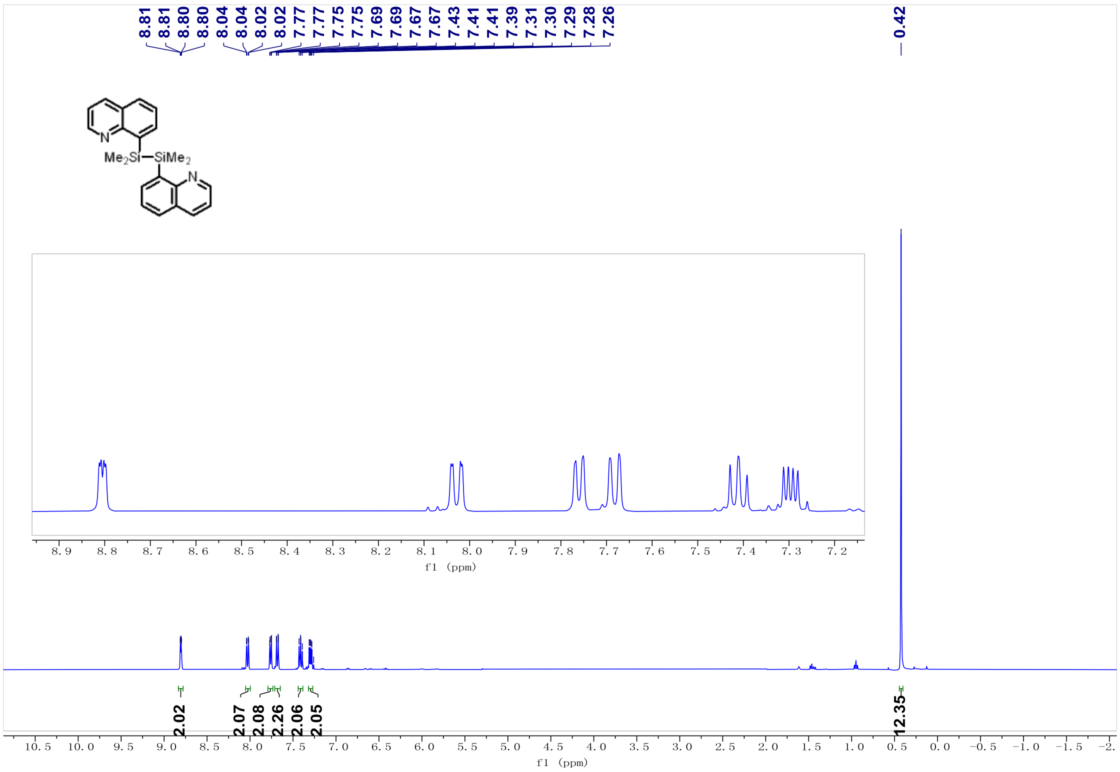

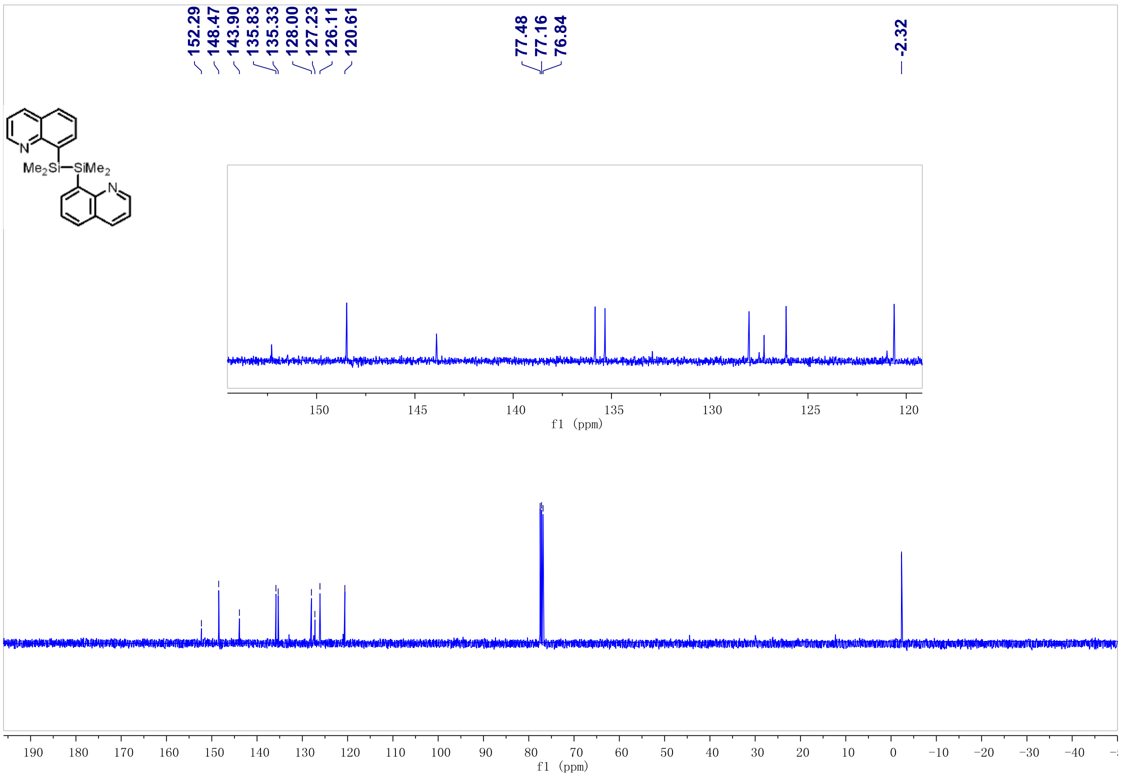

Supplementary Figure 3 ^{1}H and ^{13}C NMR Spectra for compound 1c

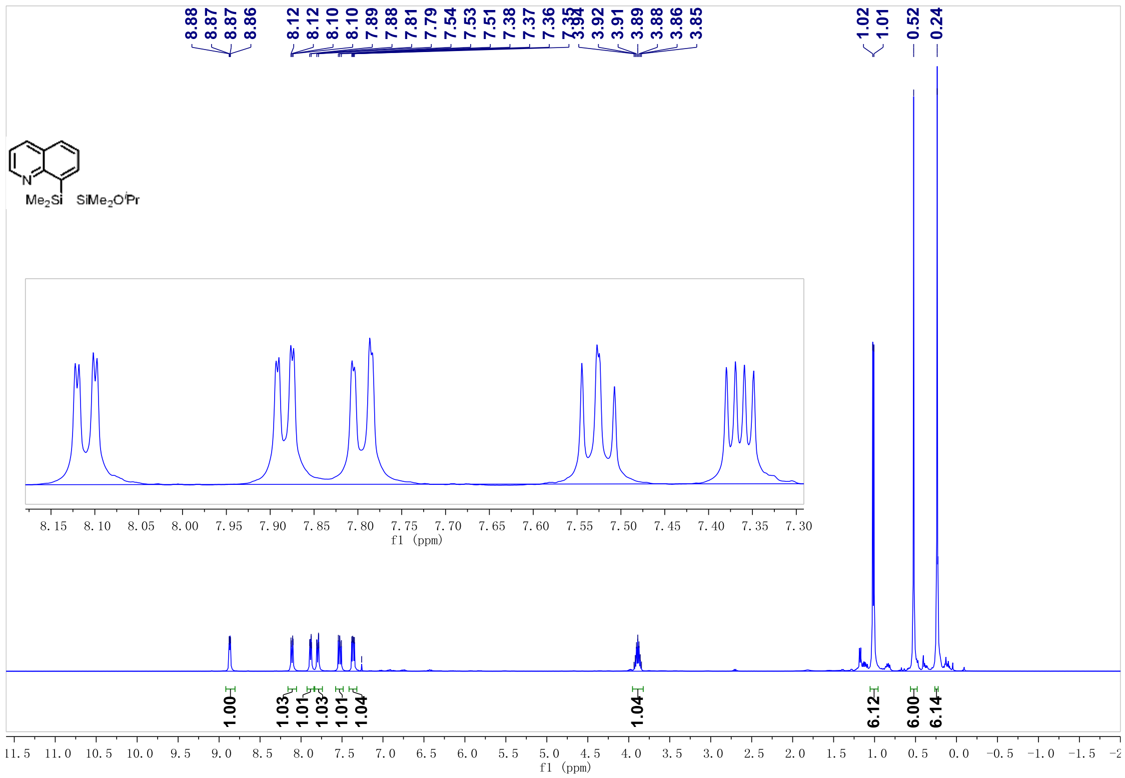

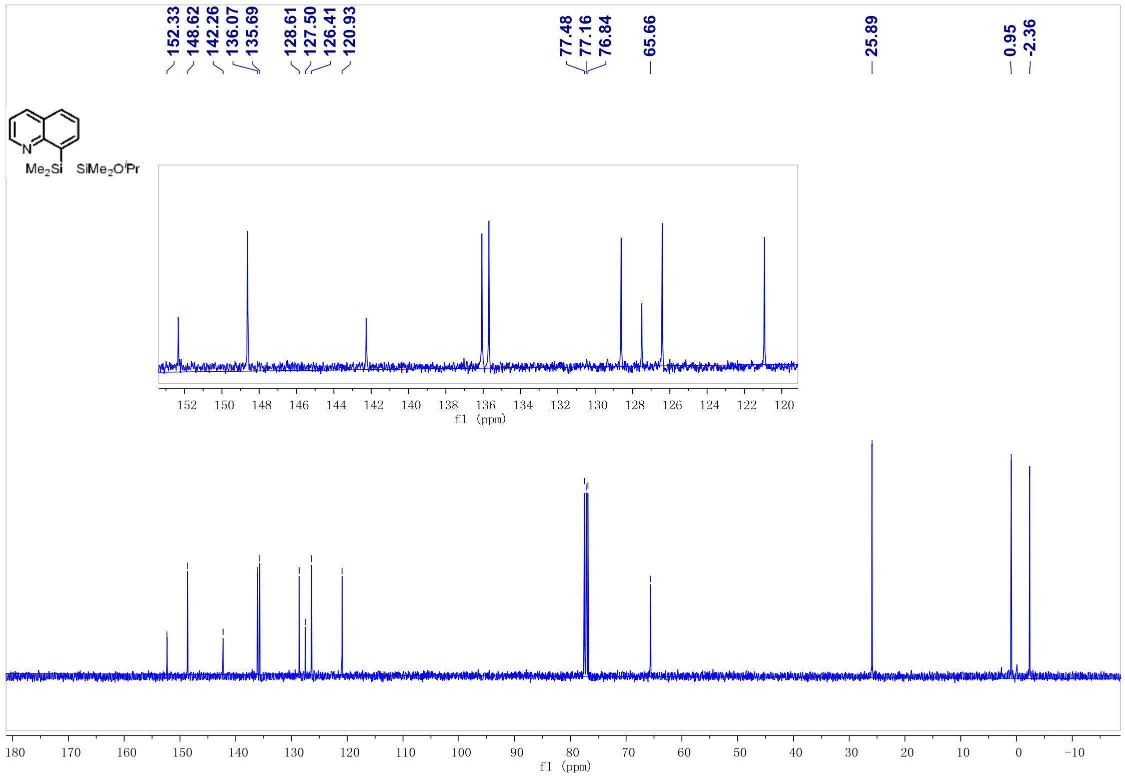

Supplementary Figure 4 ^{1}H and ^{13}C NMR Spectra for compound 1d

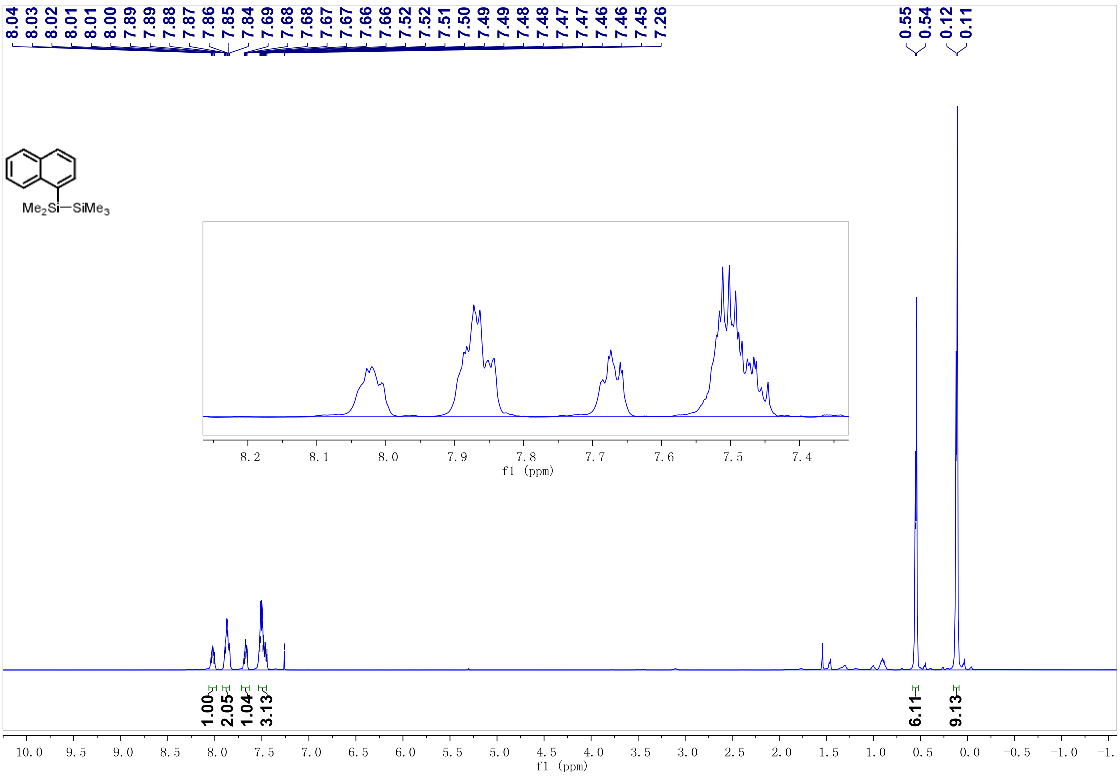

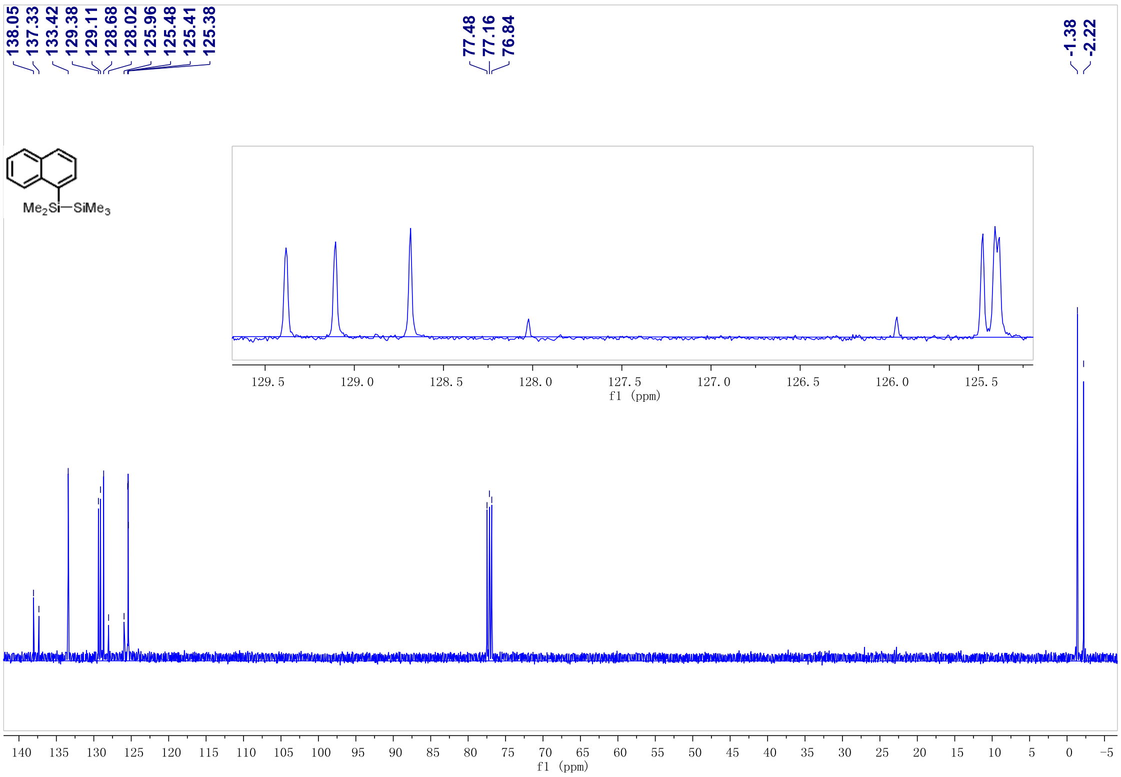

Supplementary Figure 5 ^{1}H and ^{13}C NMR Spectra for compound 1e

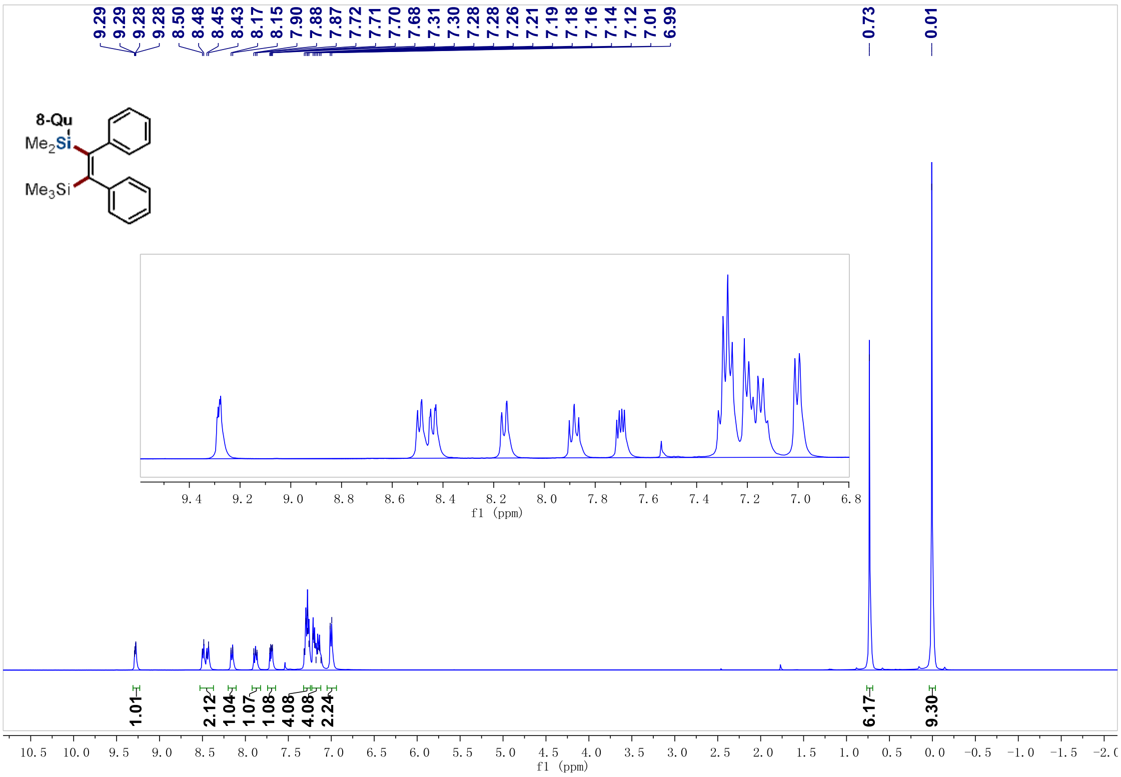

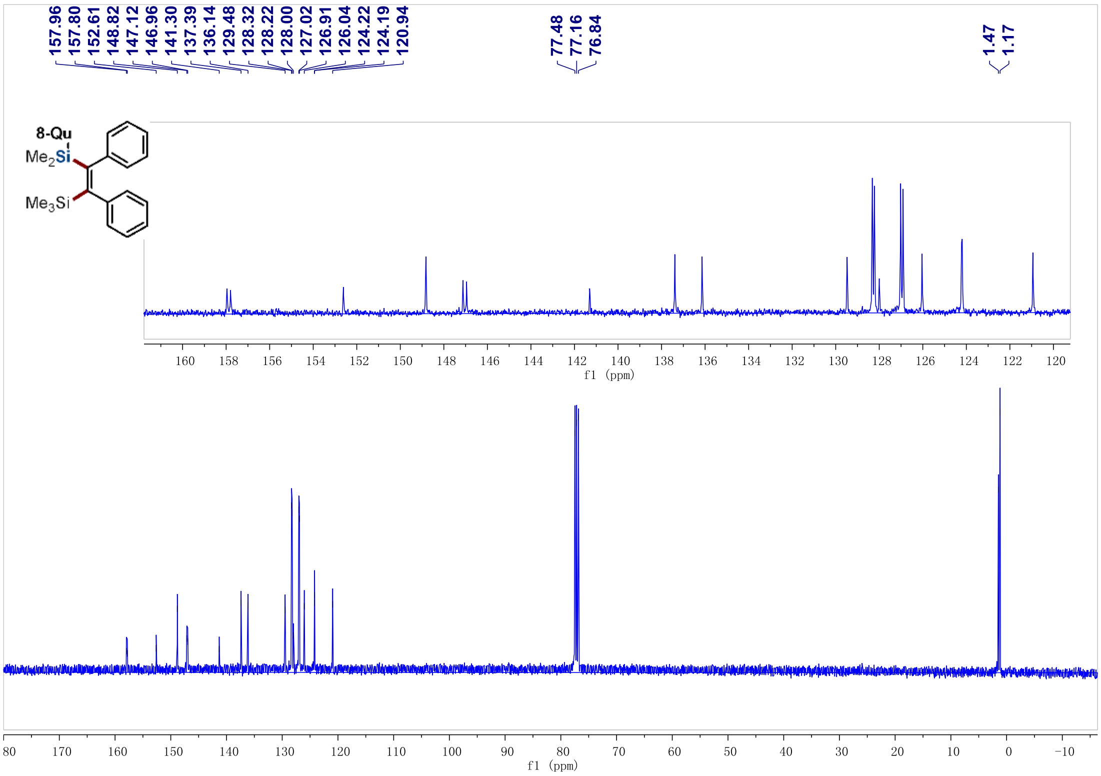

Supplementary Figure 6 ^{1}H and ^{13}C NMR Spectra for compound 3aa

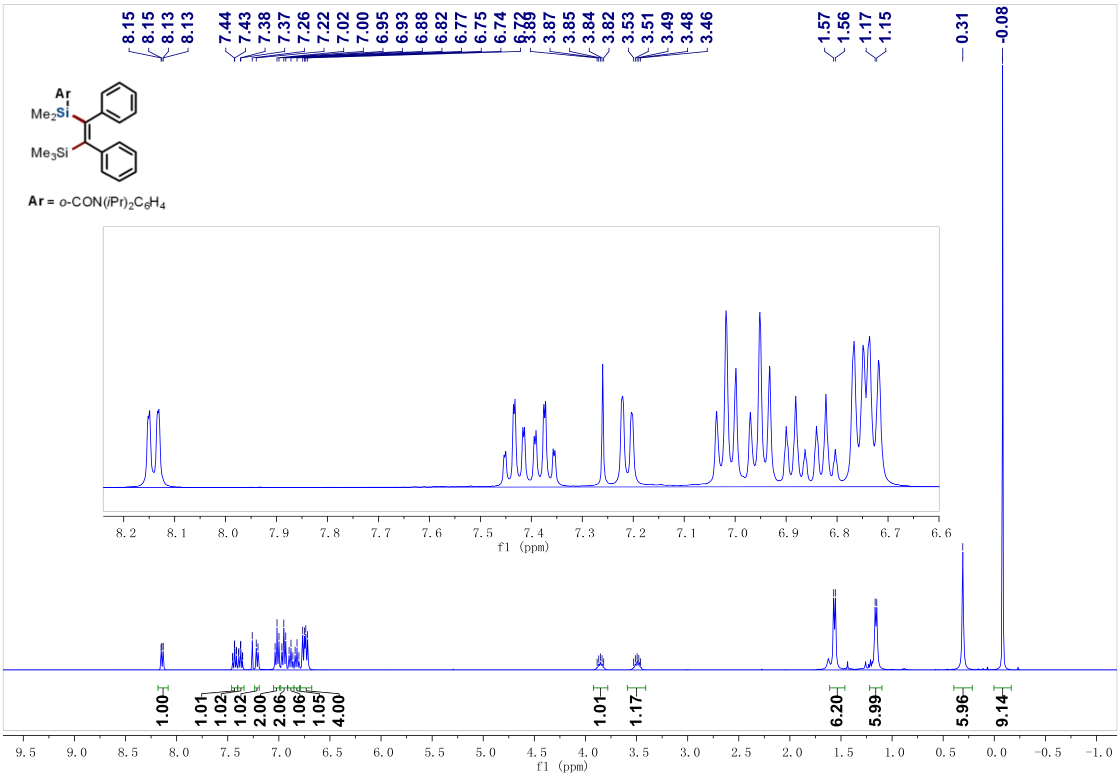

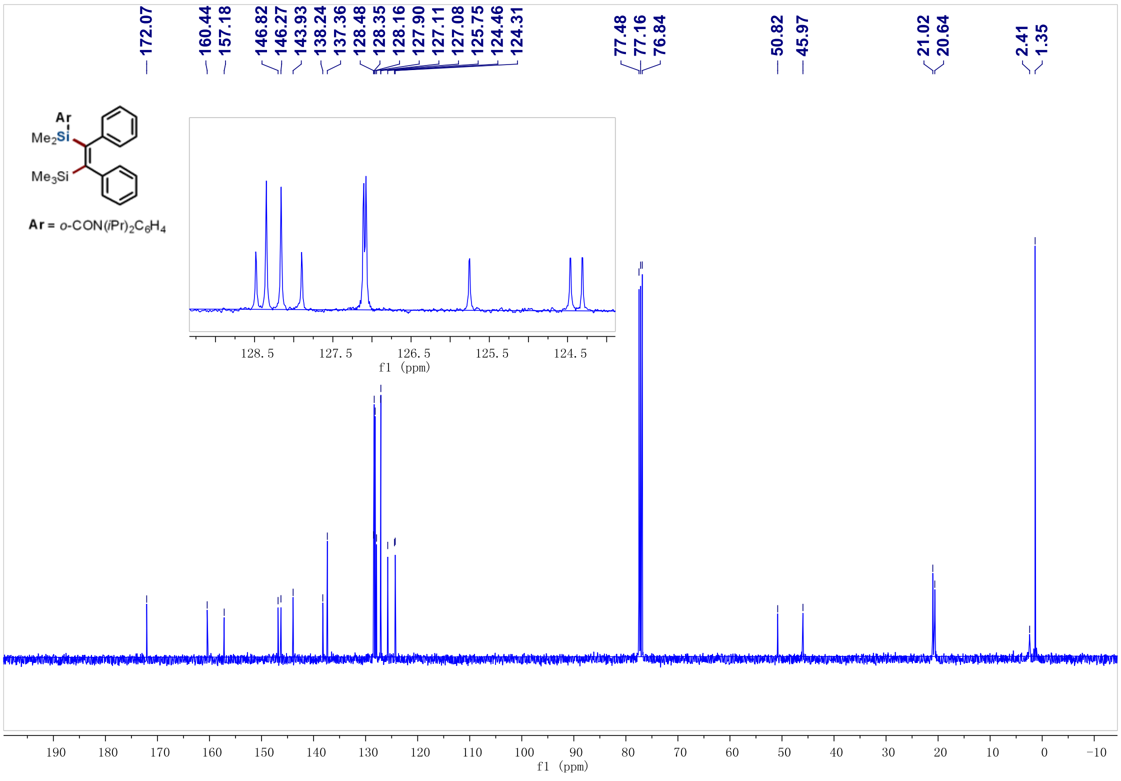

Supplementary Figure 7 ^{1}H and ^{13}C NMR Spectra for compound 3ga

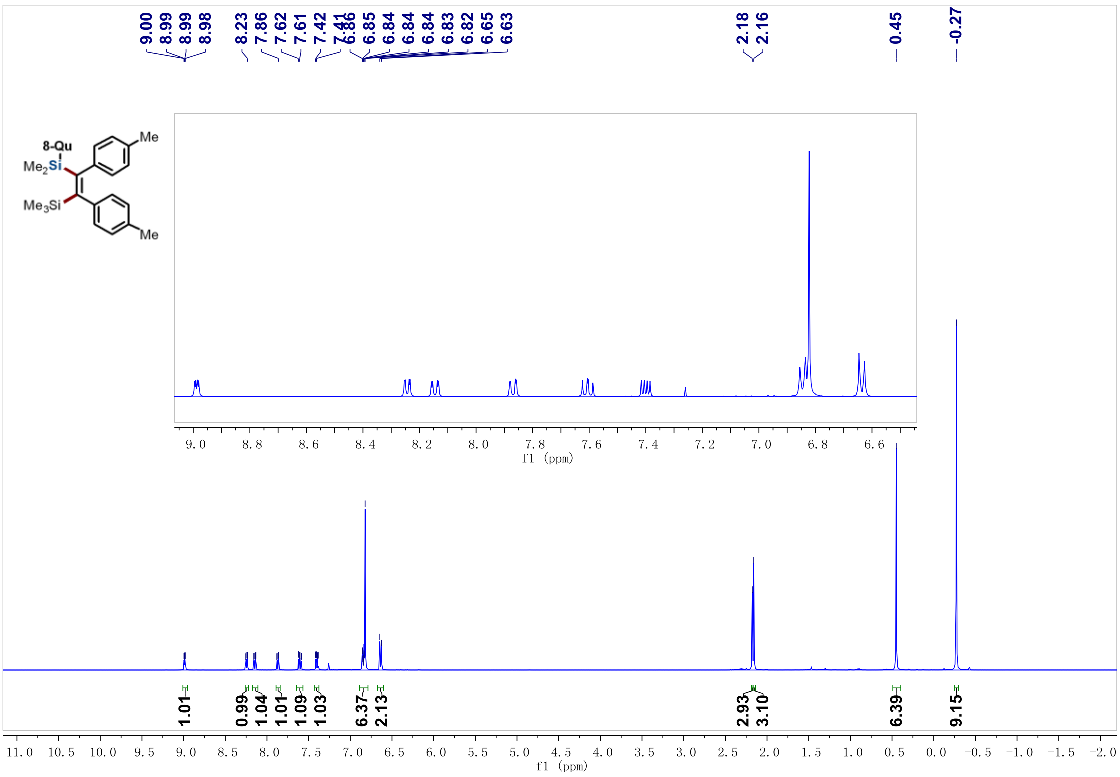

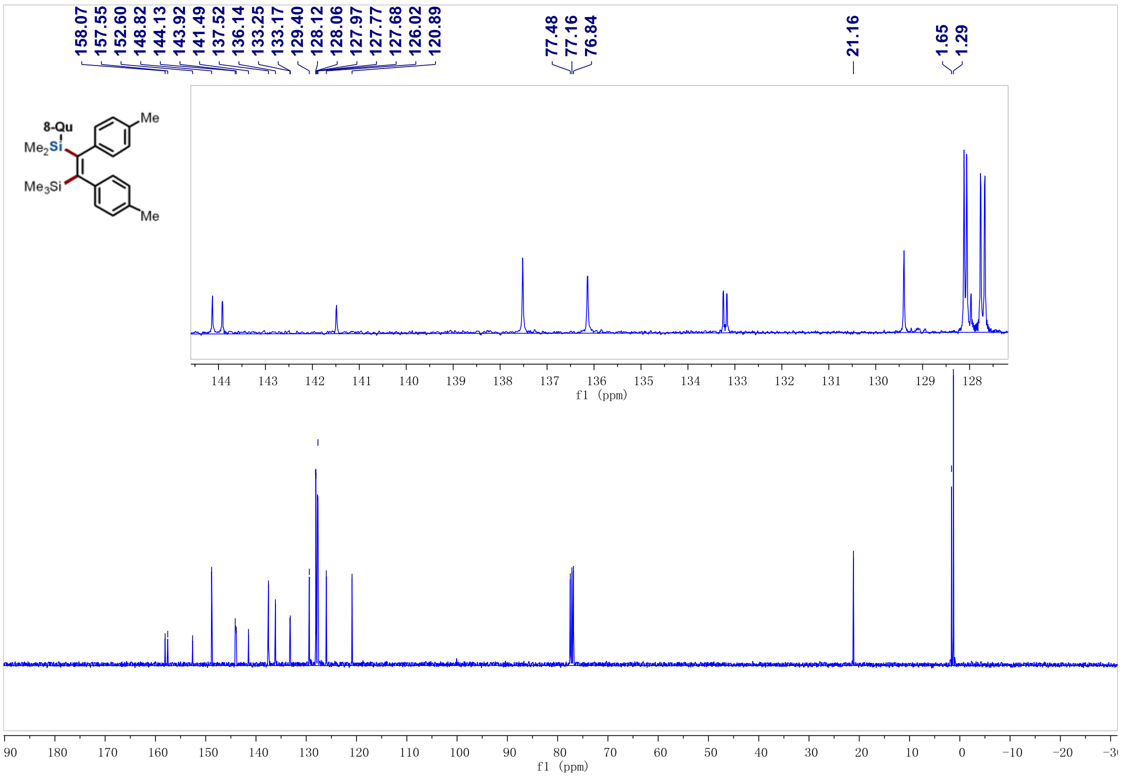

Supplementary Figure 8 ^{1}H and ^{13}C NMR Spectra for compound 3ab

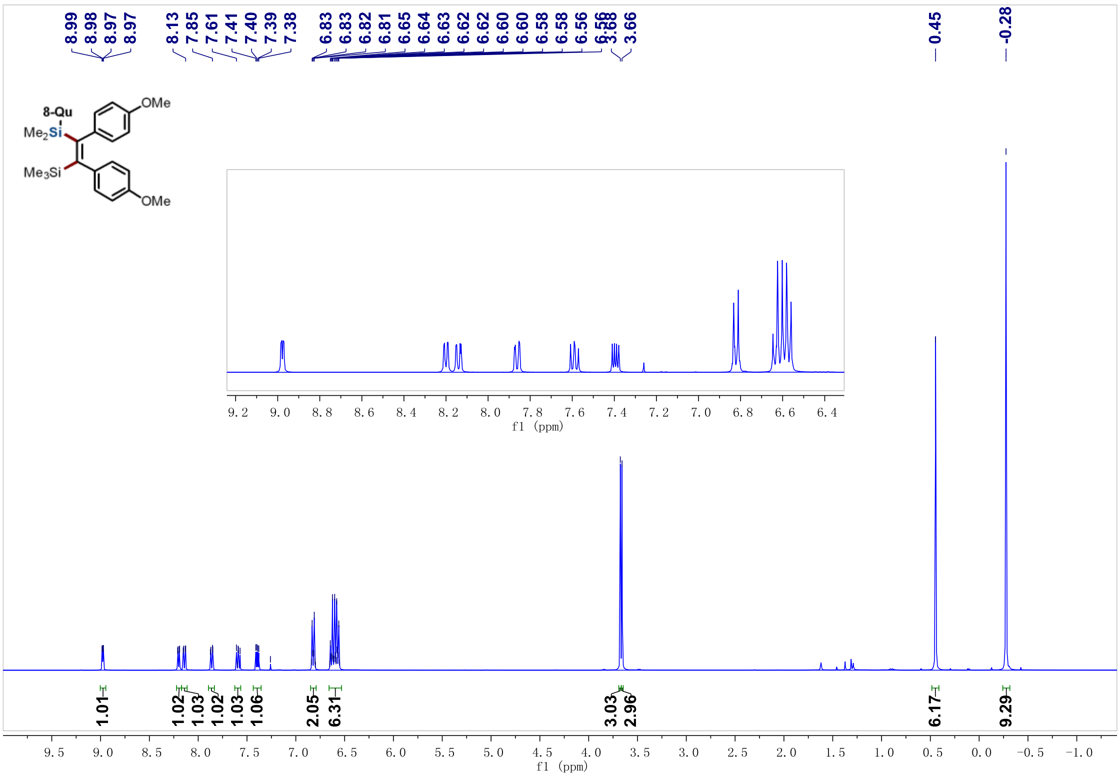

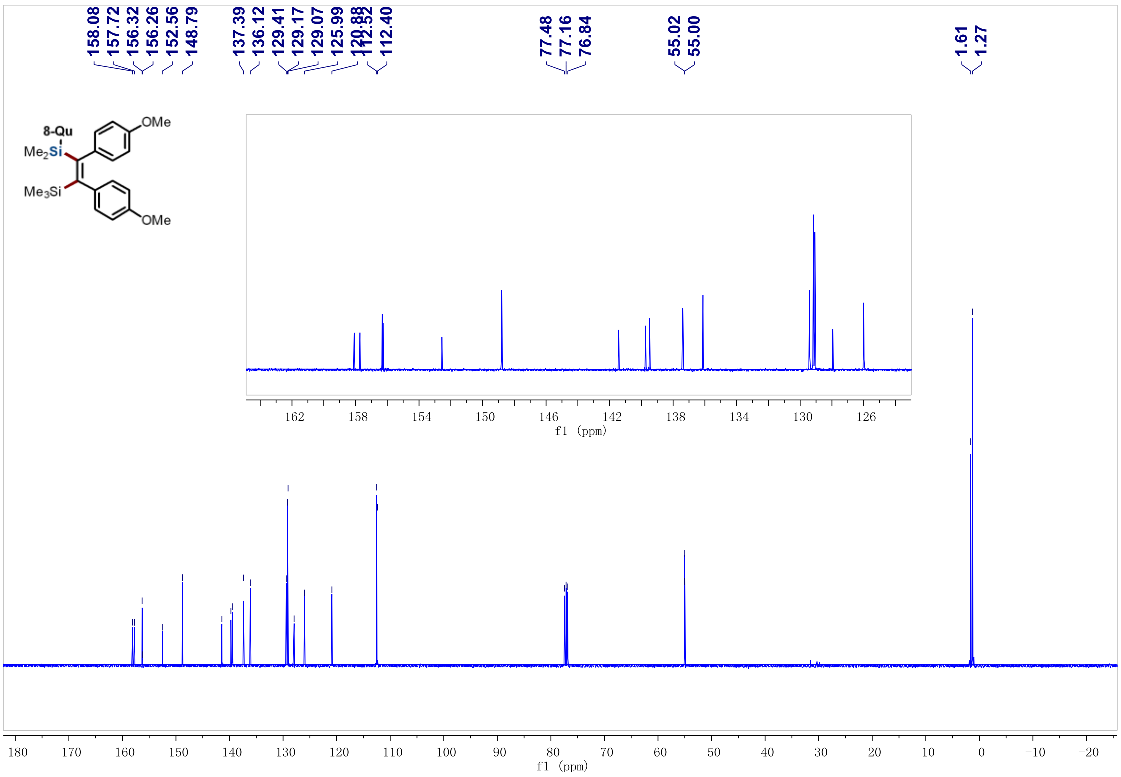

Supplementary Figure 9 ^{1}H and ^{13}C NMR Spectra for compound 3ac

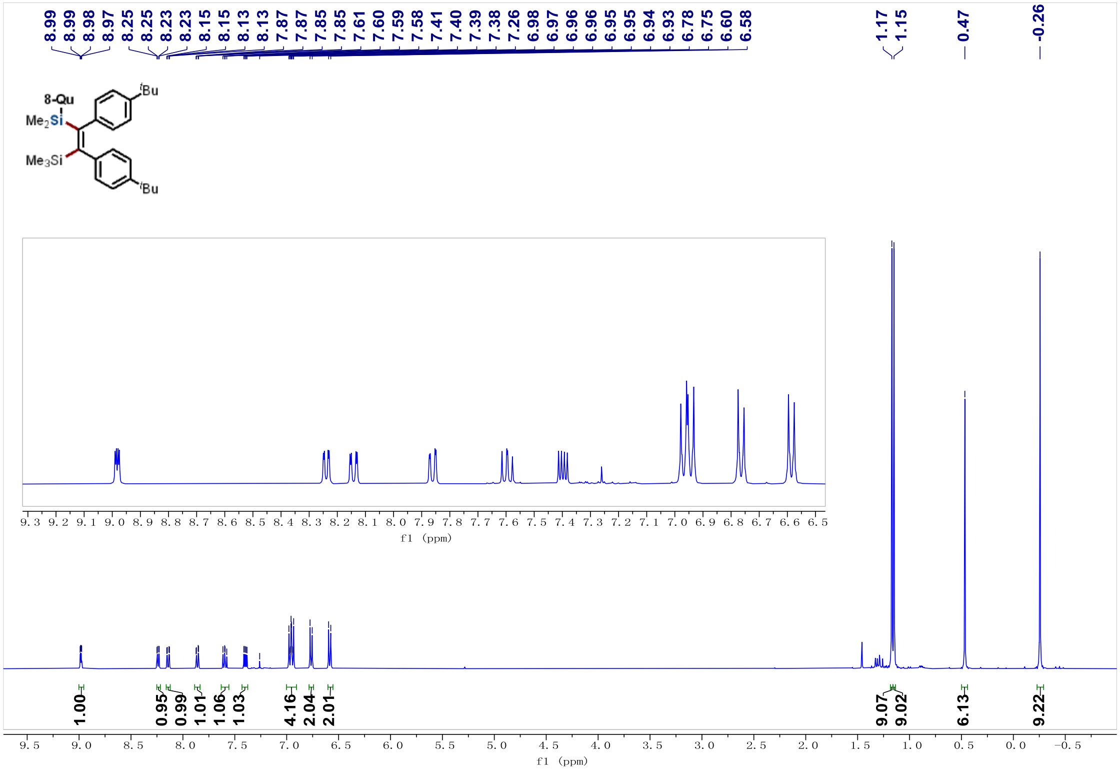

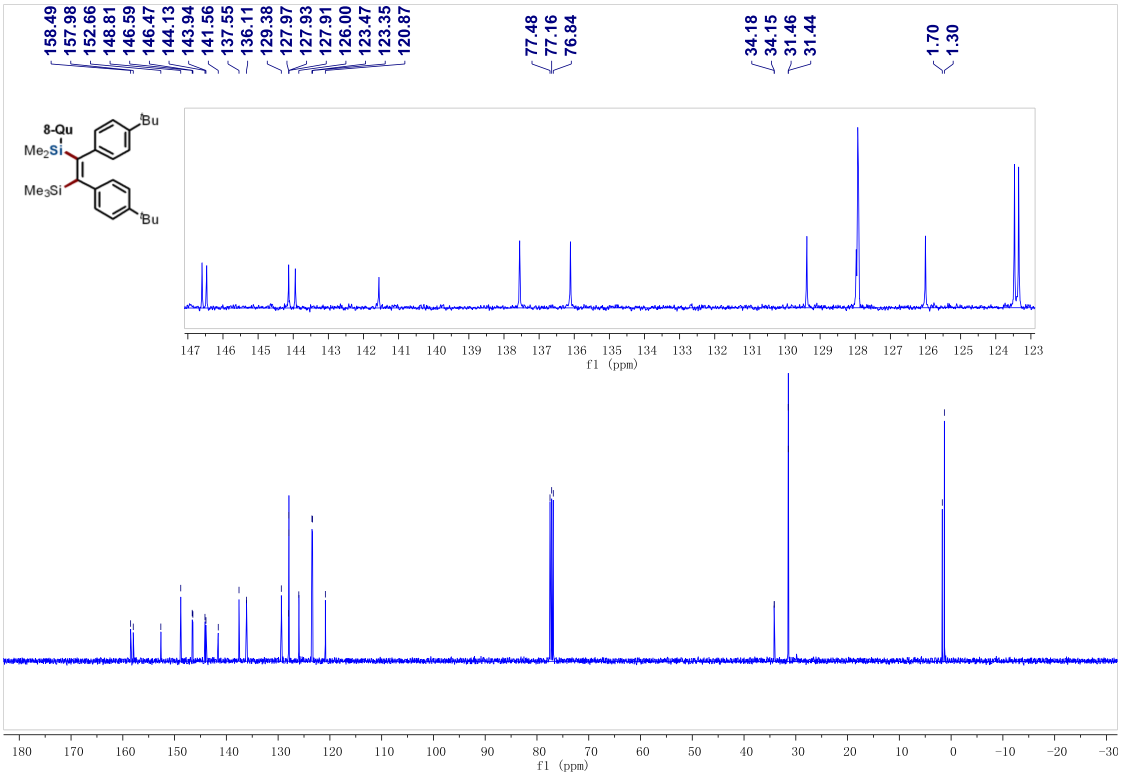

Supplementary Figure 10 ^{1}H and ^{13}C NMR Spectra for compound 3ad

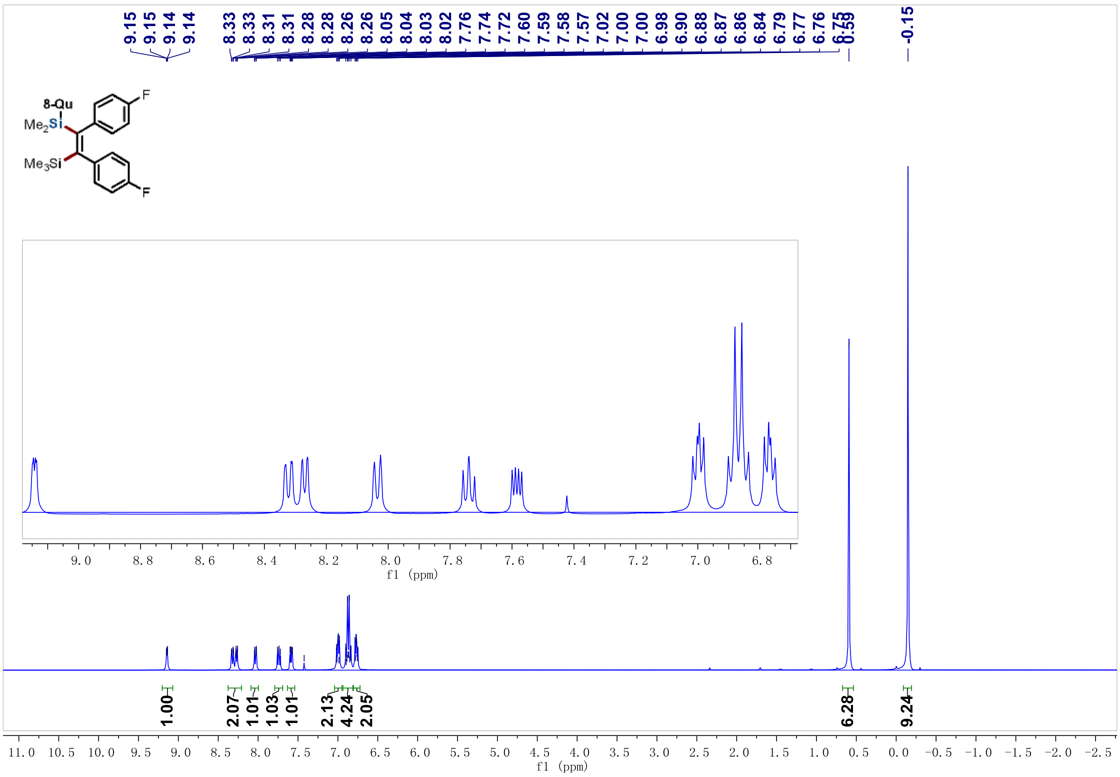
9.15
9.15
9.14
9.14
8.33
8.33
8.31
8.31
8.28
8.26
8.26
8.05
8.04
8.03
8.02
7.76
7.74
7.72
7.60
7.59
7.58
7.57
7.02
7.00
6.98
6.90
6.88
6.87
6.86
6.84
6.79
6.77
6.76
0.59
-0.15
8-Qu
Me2Si
Me3Si
F
F
1.00
2.07
1.01
1.03
1.01
2.13
4.24
2.05
6.28
9.24
f1 (ppm)

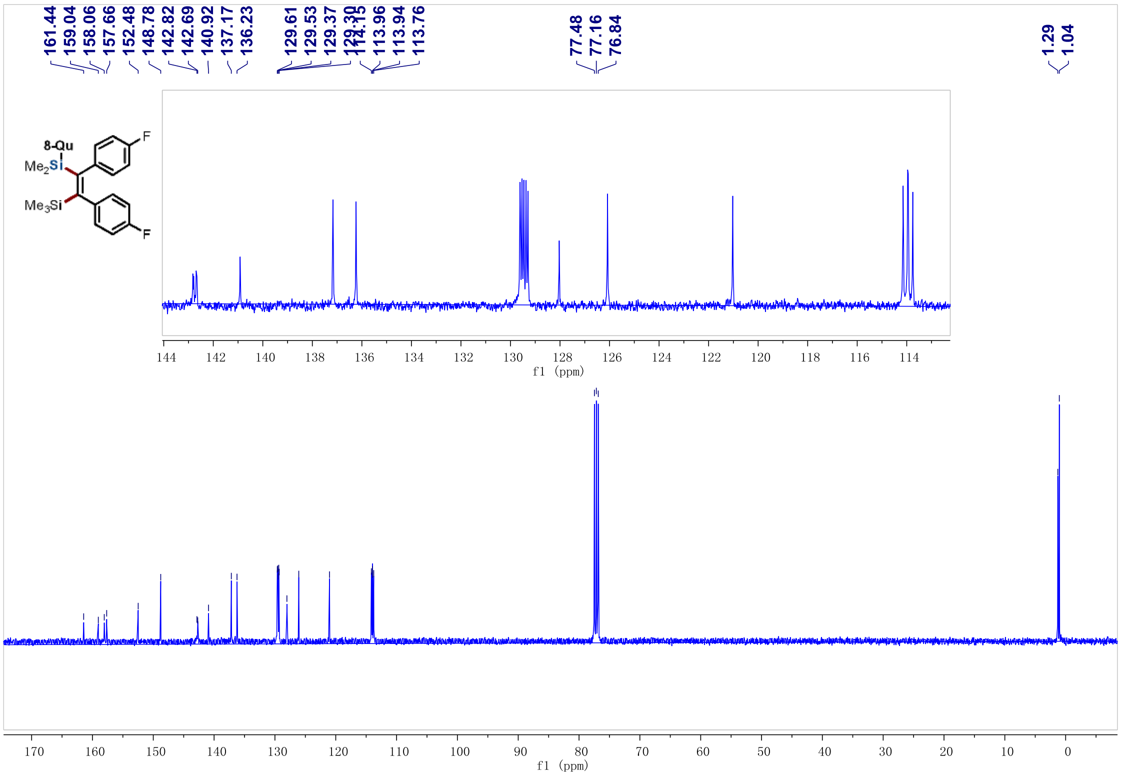
161.44
159.04
158.06
157.66
152.48
148.78
142.82
142.69
140.92
137.17
136.23
129.61
129.53
129.37
129.30
113.96
113.94
113.76
77.48
77.16
76.84
1.29
1.04
8-Qu
Me2Si
Me3Si
F
F
f1 (ppm)

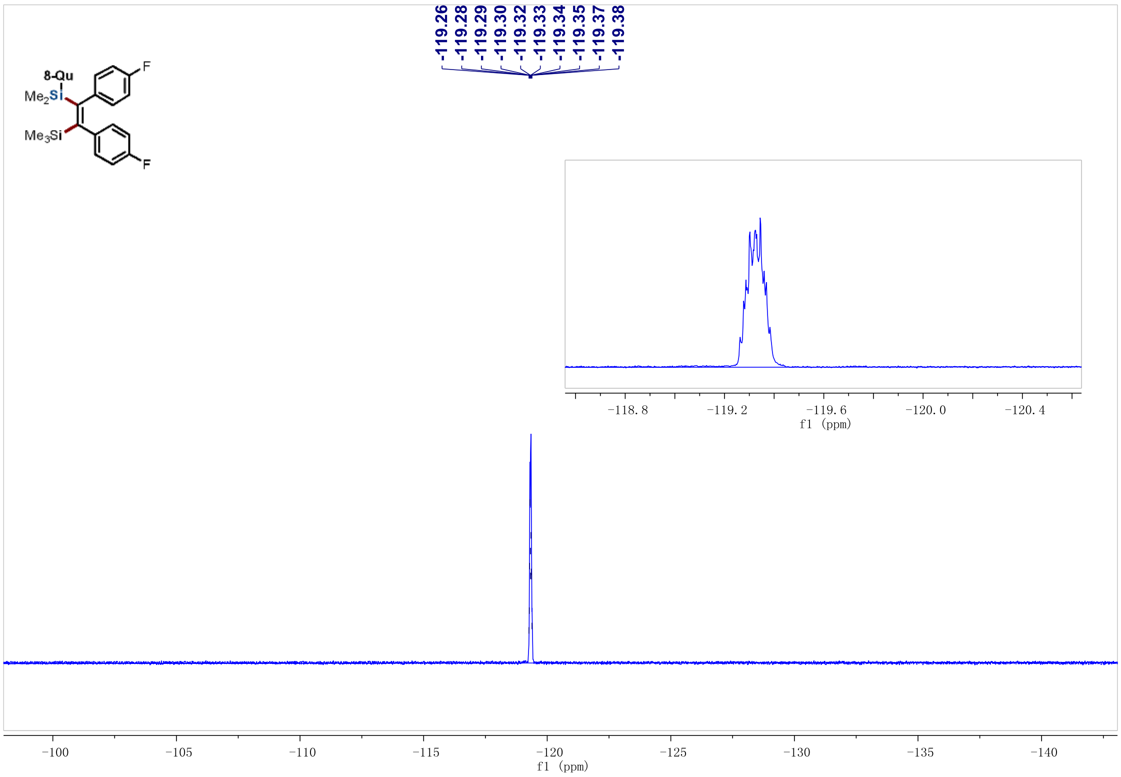

Supplementary Figure 11 ^{1}H, ^{13}C and ^{19}F NMR Spectra for compound 3ae

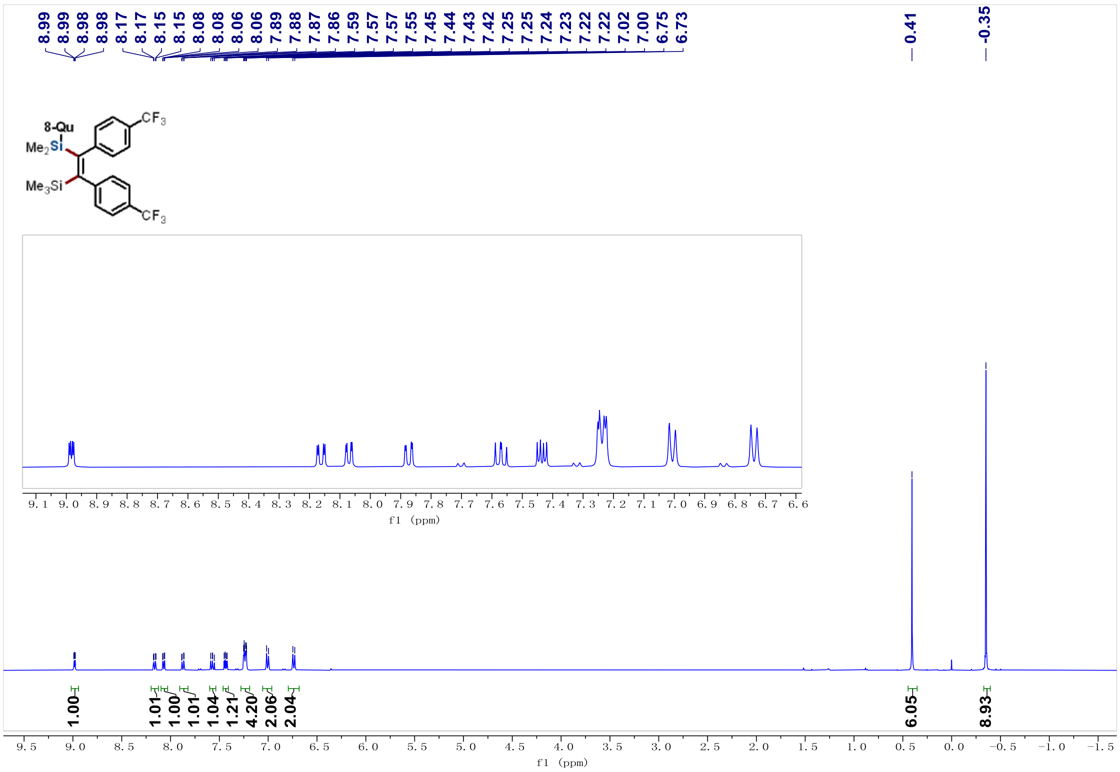
8.99
8.99
8.98
8.98
8.17
8.15
8.15
8.08
8.06
8.06
7.89
7.88
7.86
7.59
7.57
7.57
7.55
7.45
7.44
7.43
7.42
7.25
7.25
7.24
7.23
7.22
7.22
7.02
7.00
6.75
6.73
0.41
-0.35
8-Qu
Me2Si
Me3Si
CF3
CF3
1.00
1.01
1.00
1.01
1.04
1.21
4.20
2.06
2.04
6.05
8.93
f1 (ppm)

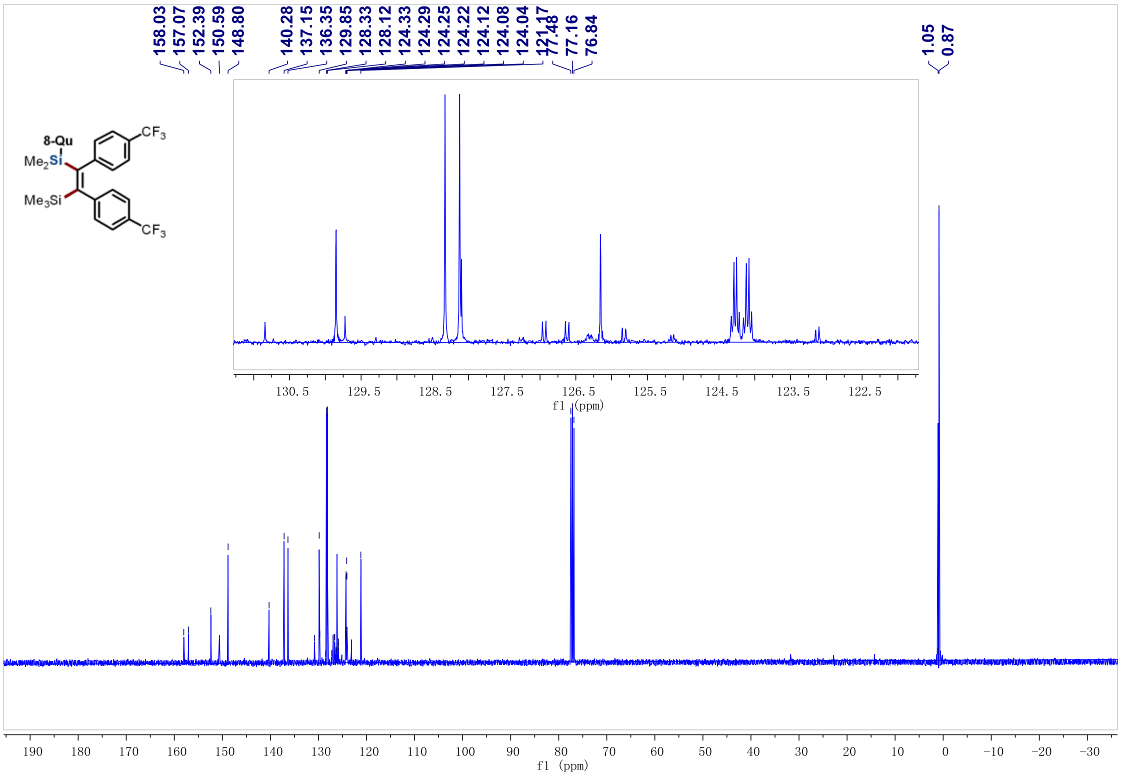
158.03
157.07
152.39
150.59
148.80
140.28
137.15
136.35
129.85
128.33
128.12
124.33
124.29
124.25
124.22
124.12
124.08
124.04
121.17
77.48
77.16
76.84
1.05
0.87
8-Qu
Me2Si
Me3Si
CF3
CF3
f1 (ppm)

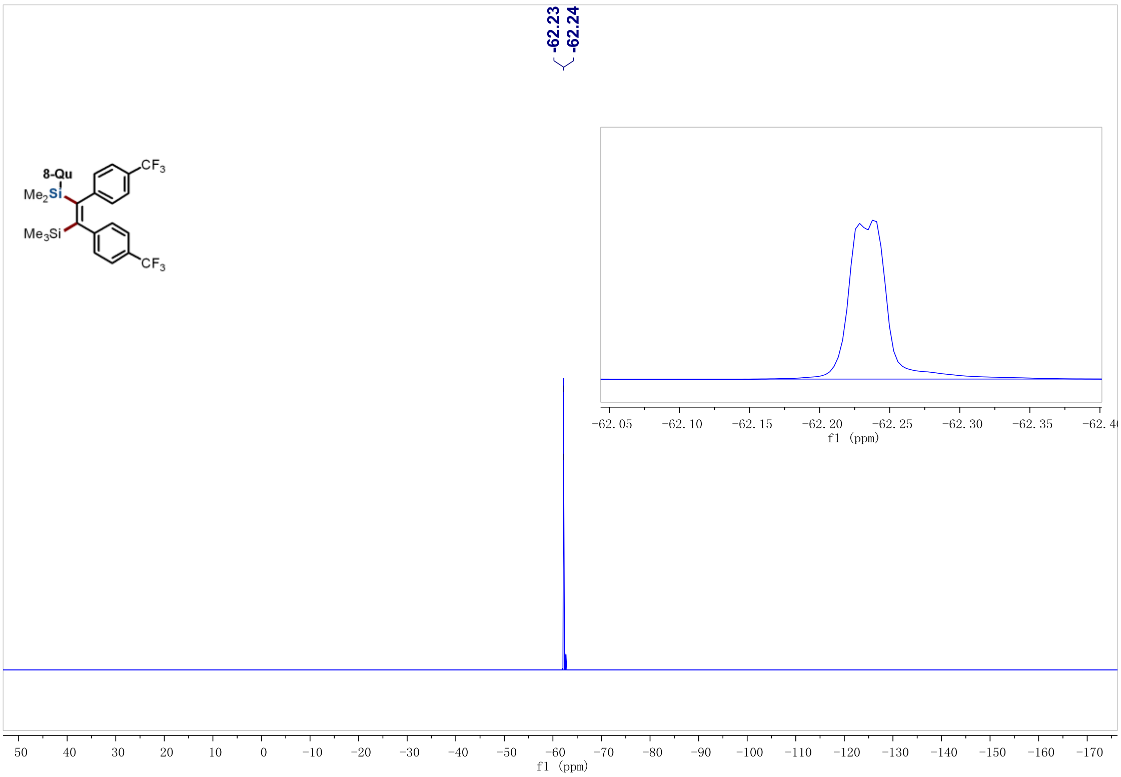

Supplementary Figure 12 ^{1}H, ^{13}C and ^{19}F NMR Spectra for compound 3af

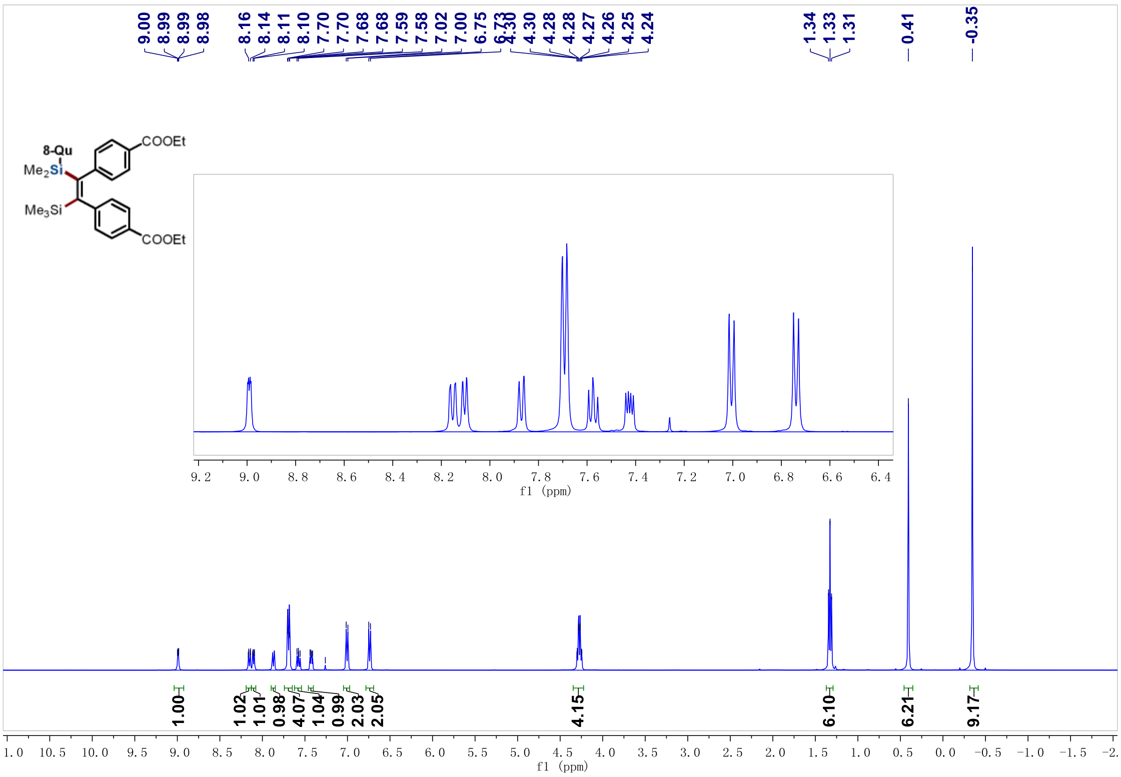

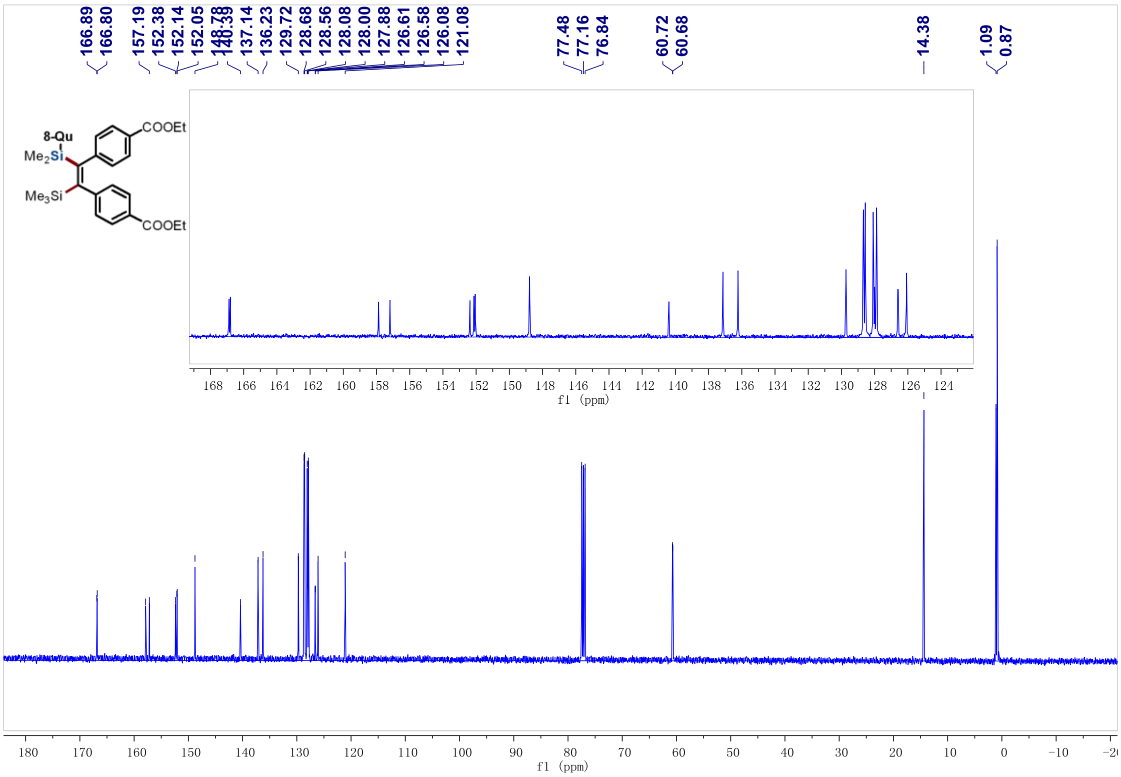

Supplementary Figure 13 ^{1}H and ^{13}C NMR Spectra for compound 3ag

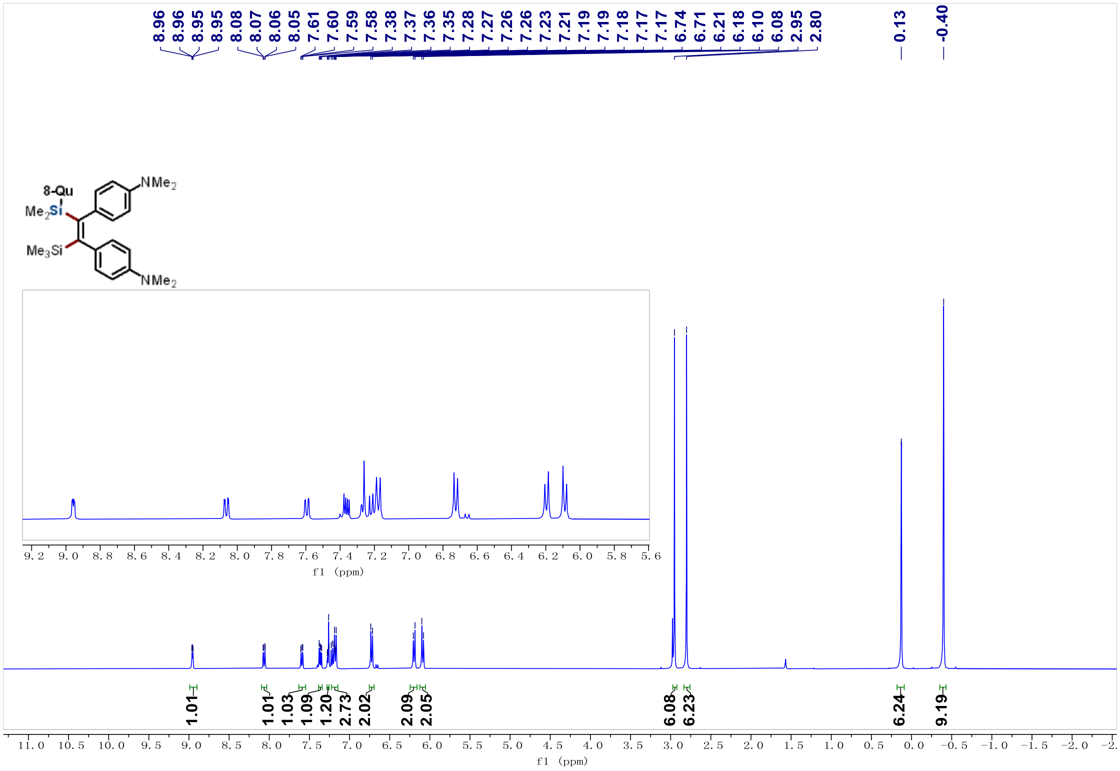

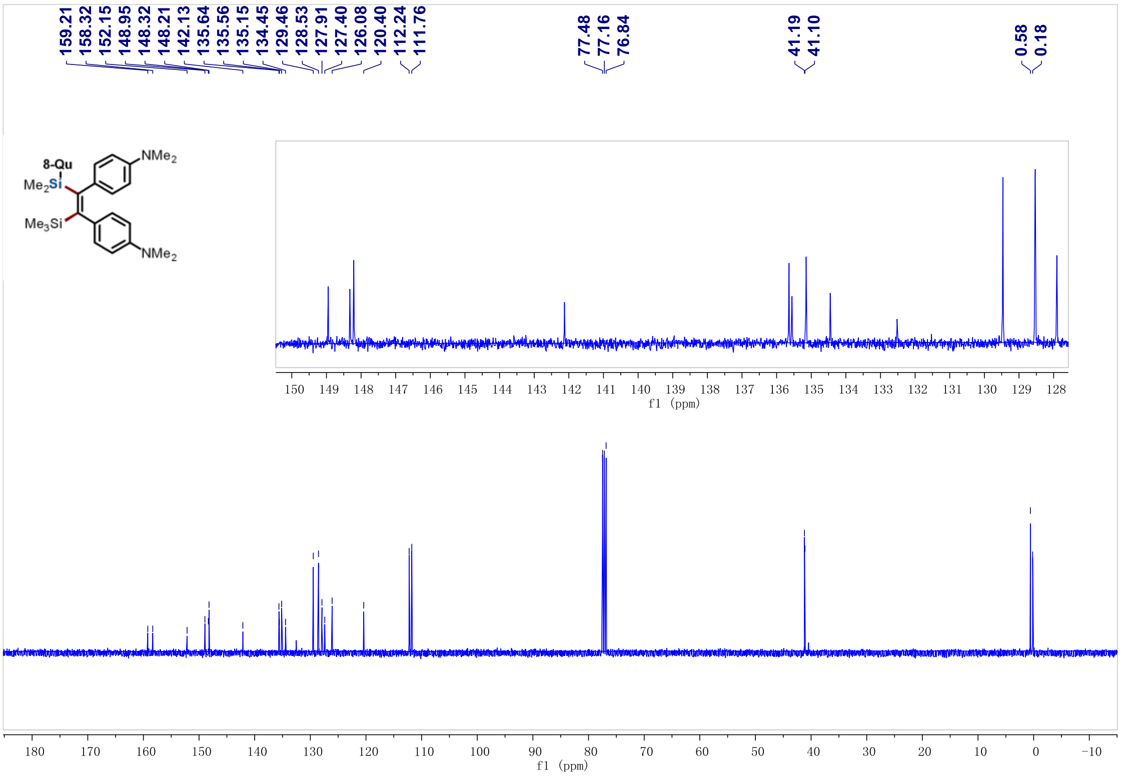

Supplementary Figure 14 ^{1}H and ^{13}C NMR Spectra for compound 3ah

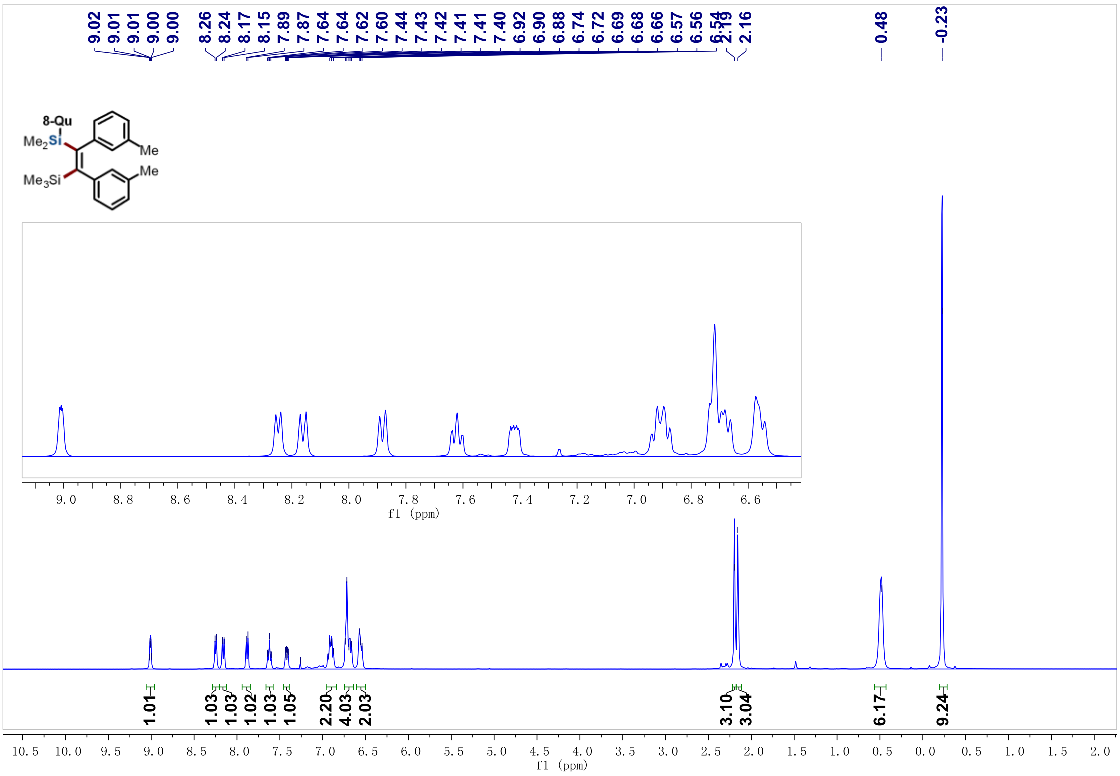

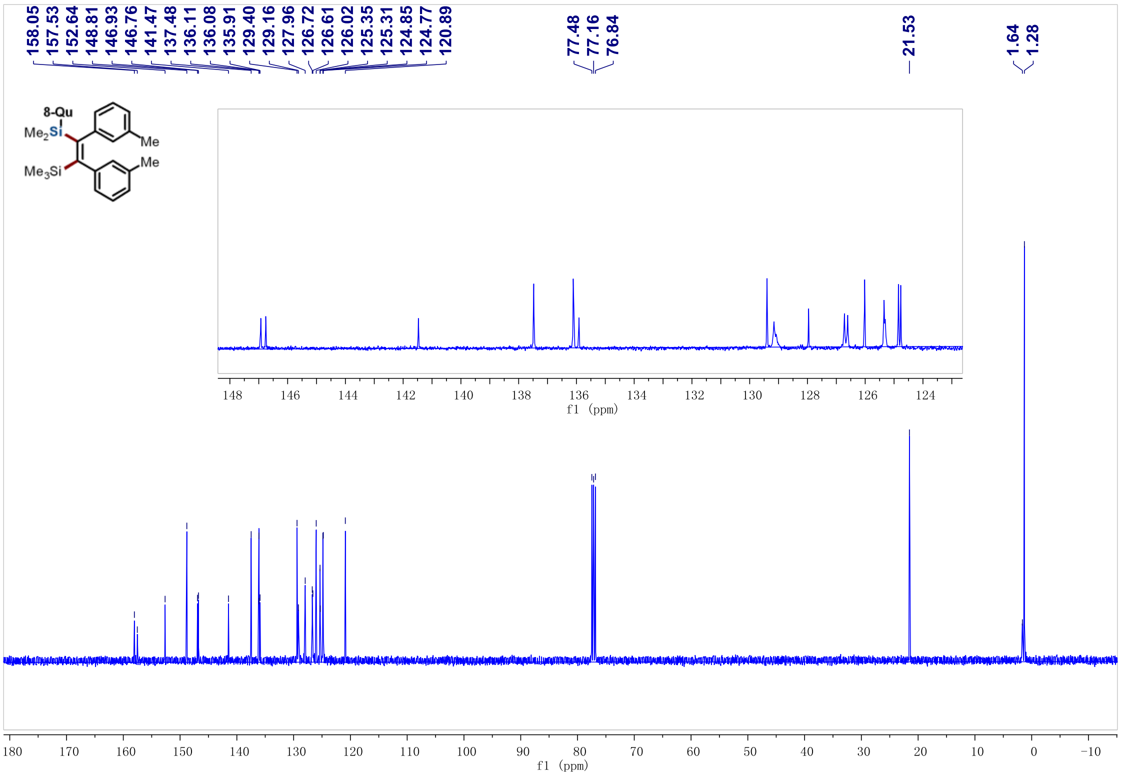

Supplementary Figure 15 ^{1}H and ^{13}C NMR Spectra for compound 3ai

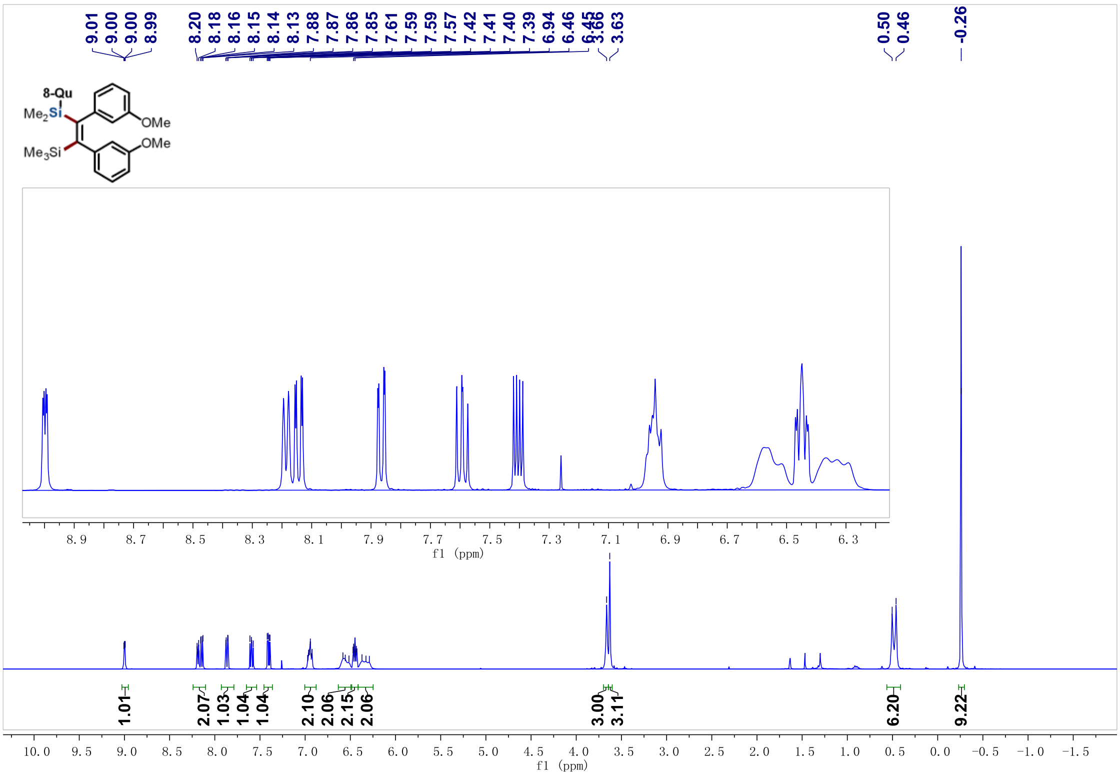

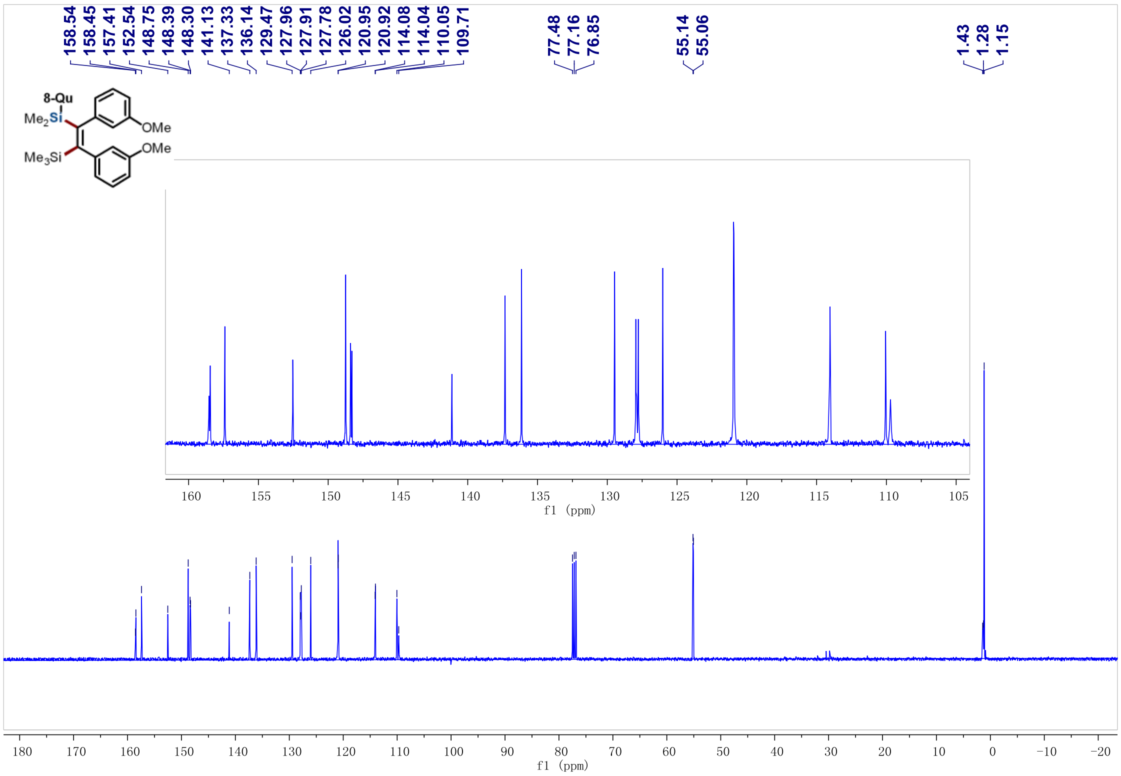

Supplementary Figure 16 ^{1}H and ^{13}C NMR Spectra for compound 3aj

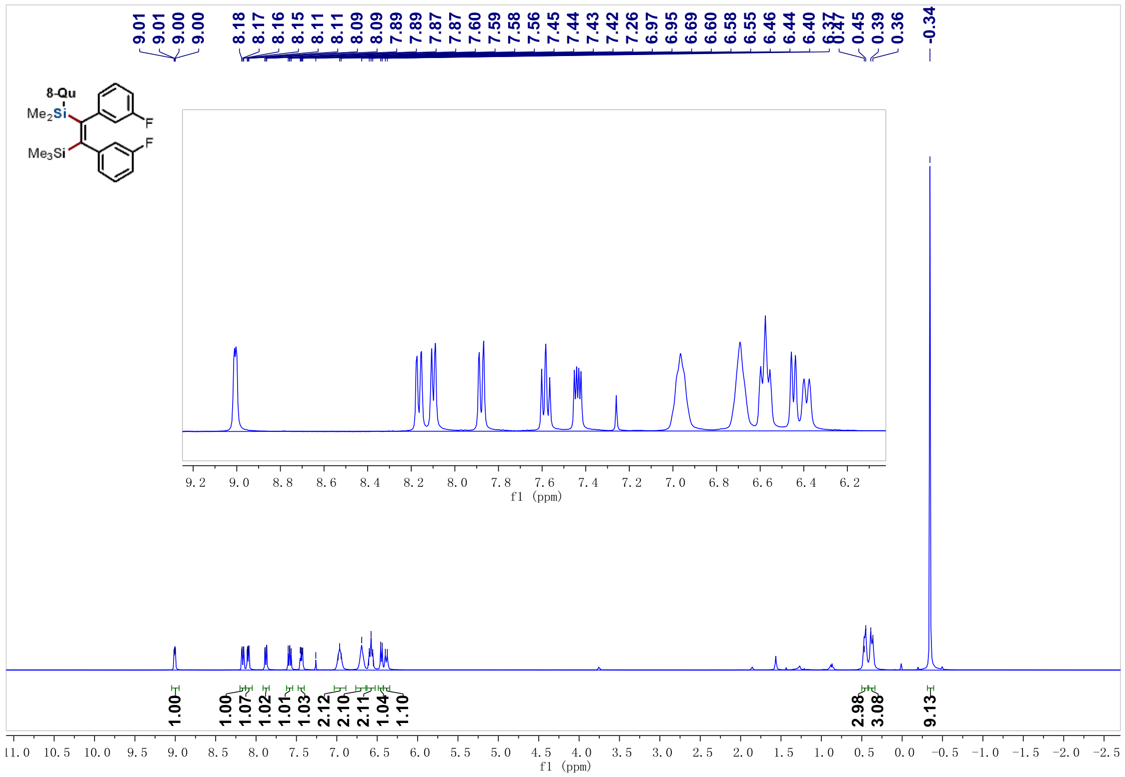

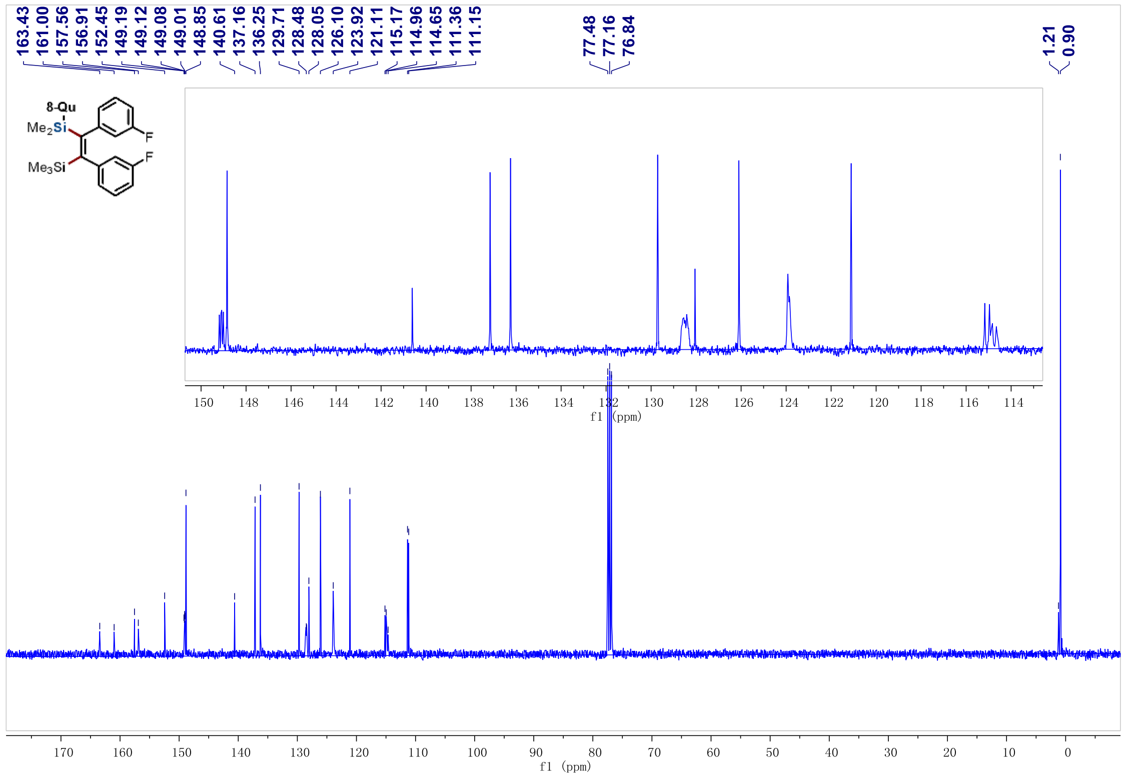

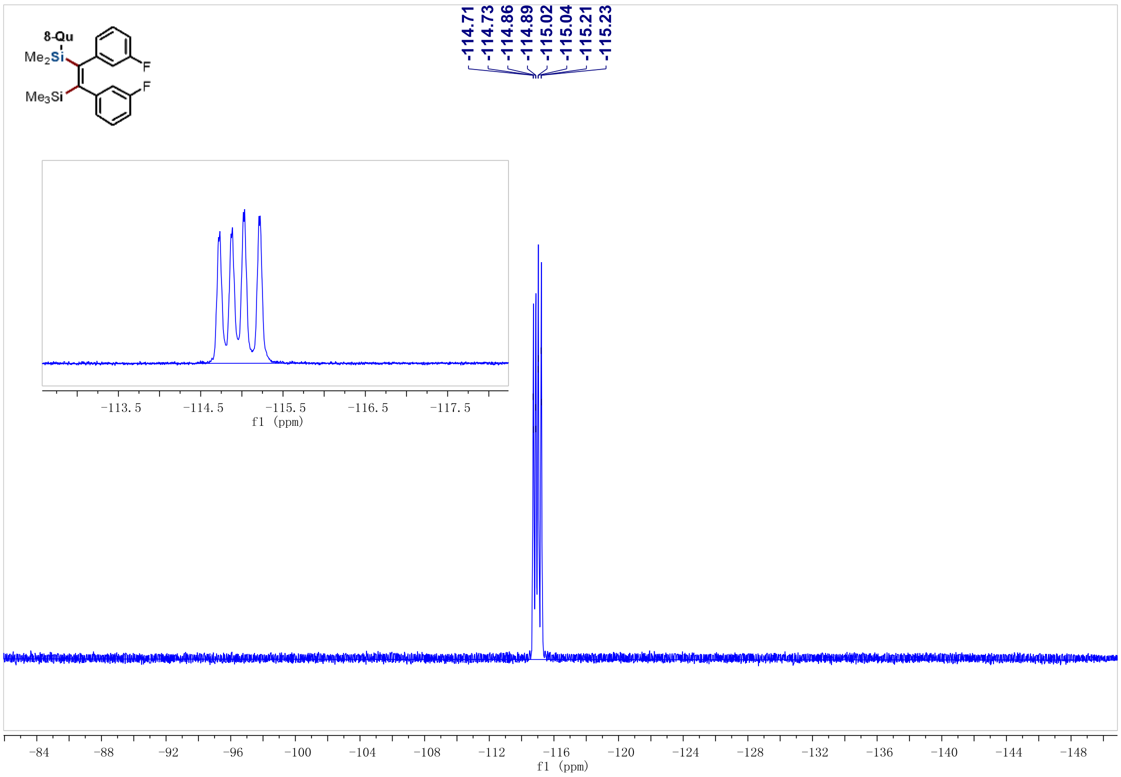

Supplementary Figure 17 ^{1}H, ^{13}C and ^{19}F NMR Spectra for compound 3ak

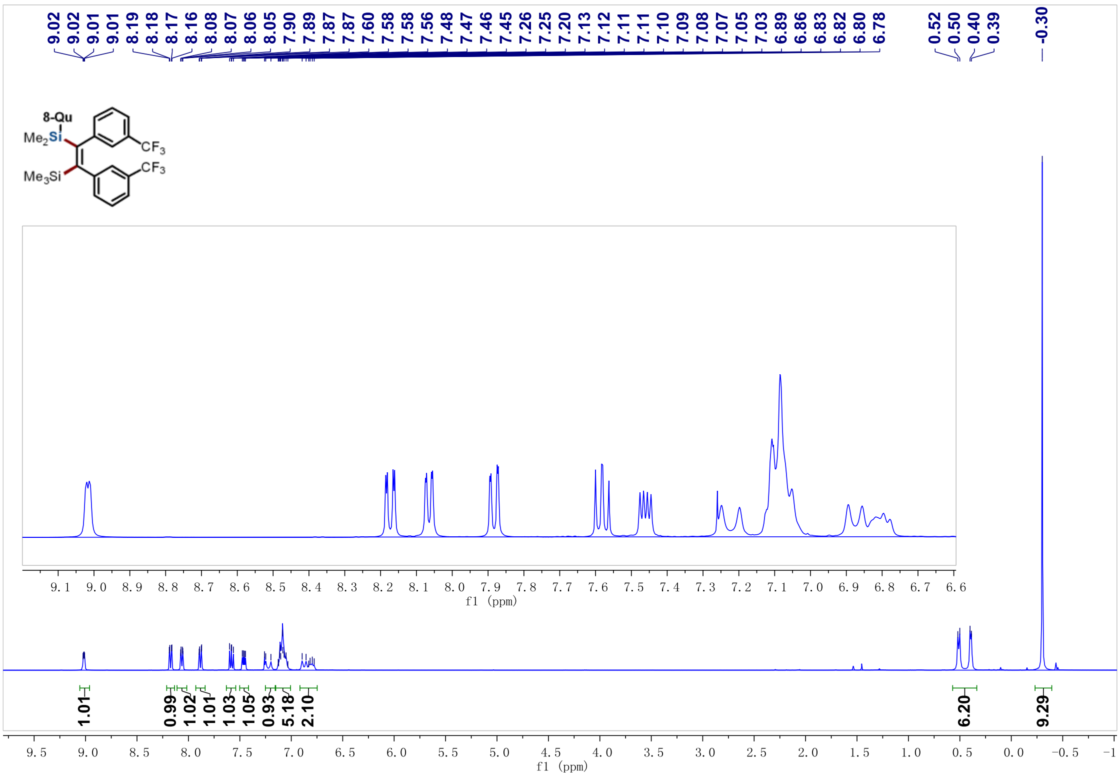

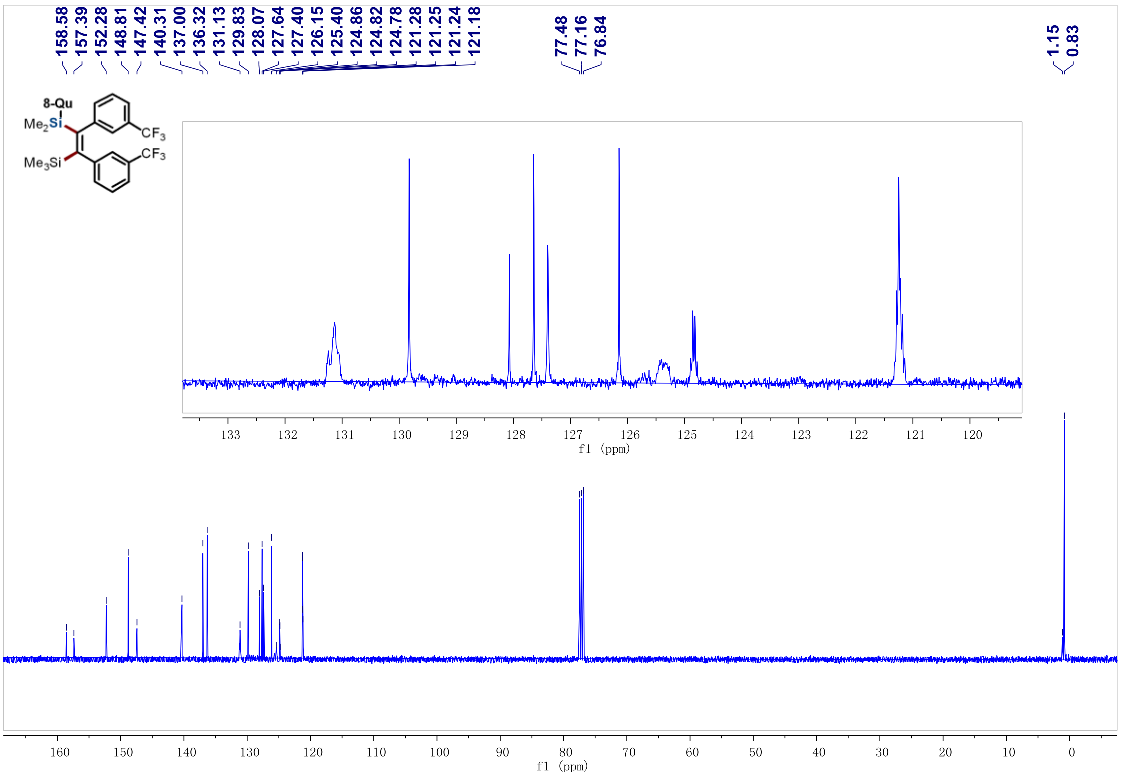

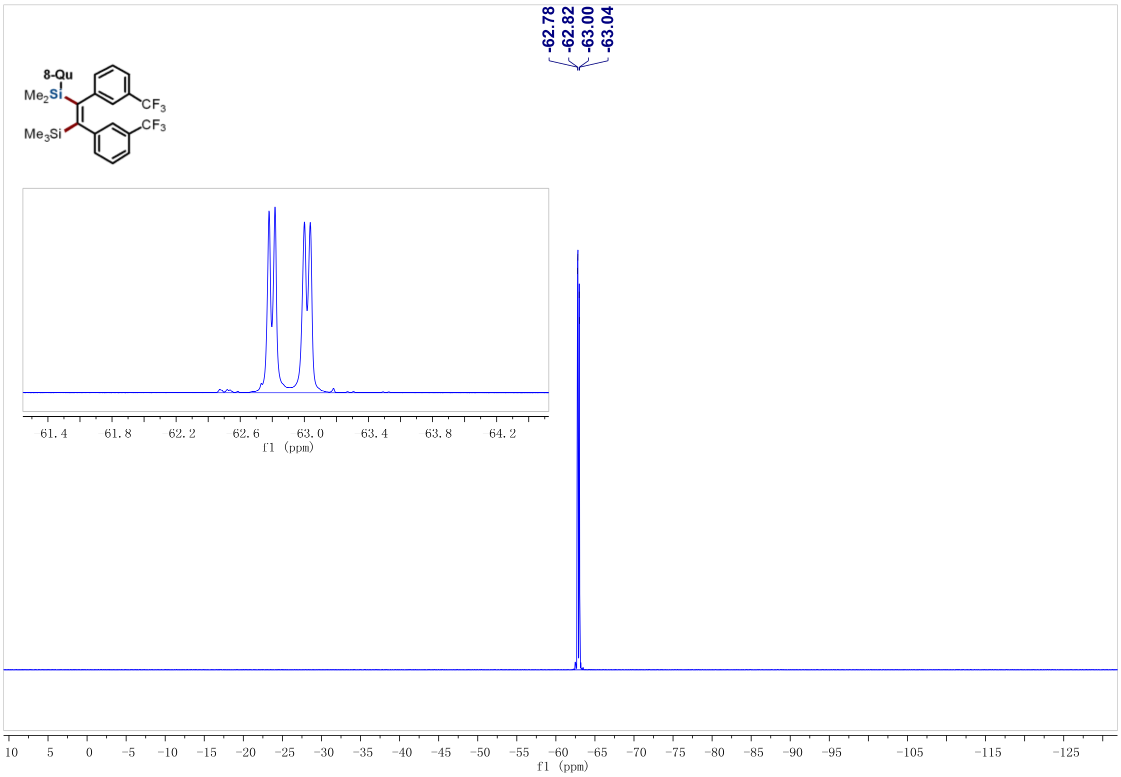

Supplementary Figure 18 ^{1}H, ^{13}C and ^{19}F NMR Spectra for compound 3al

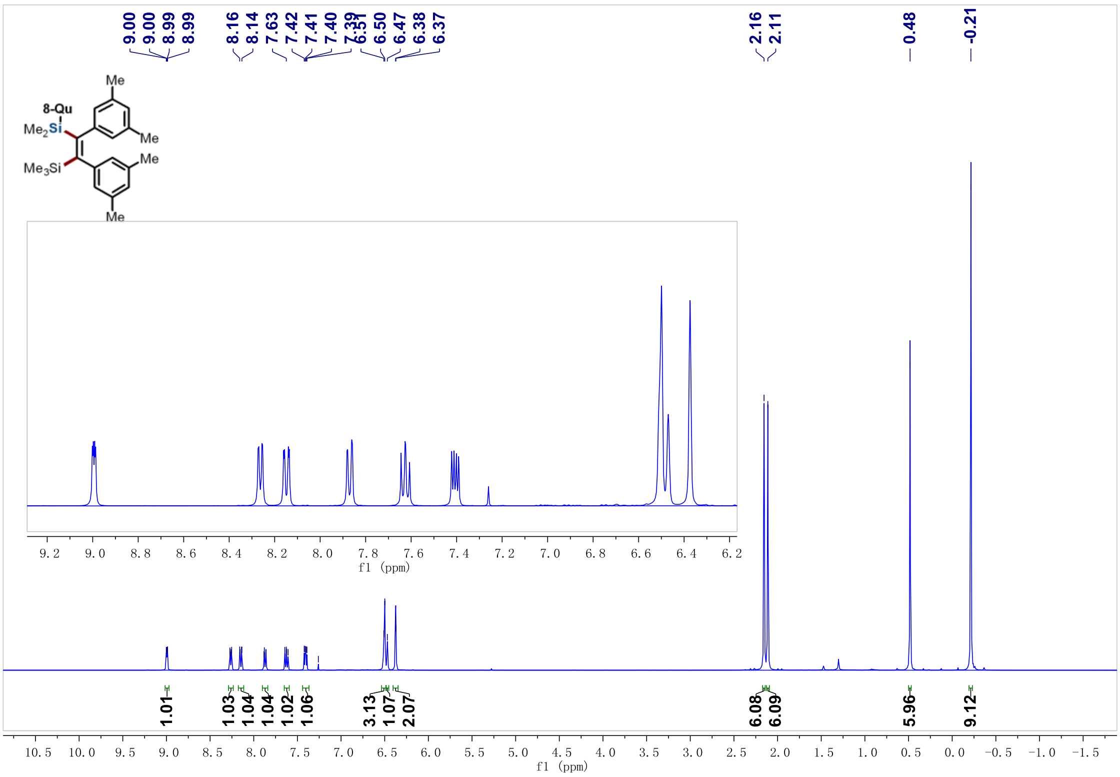

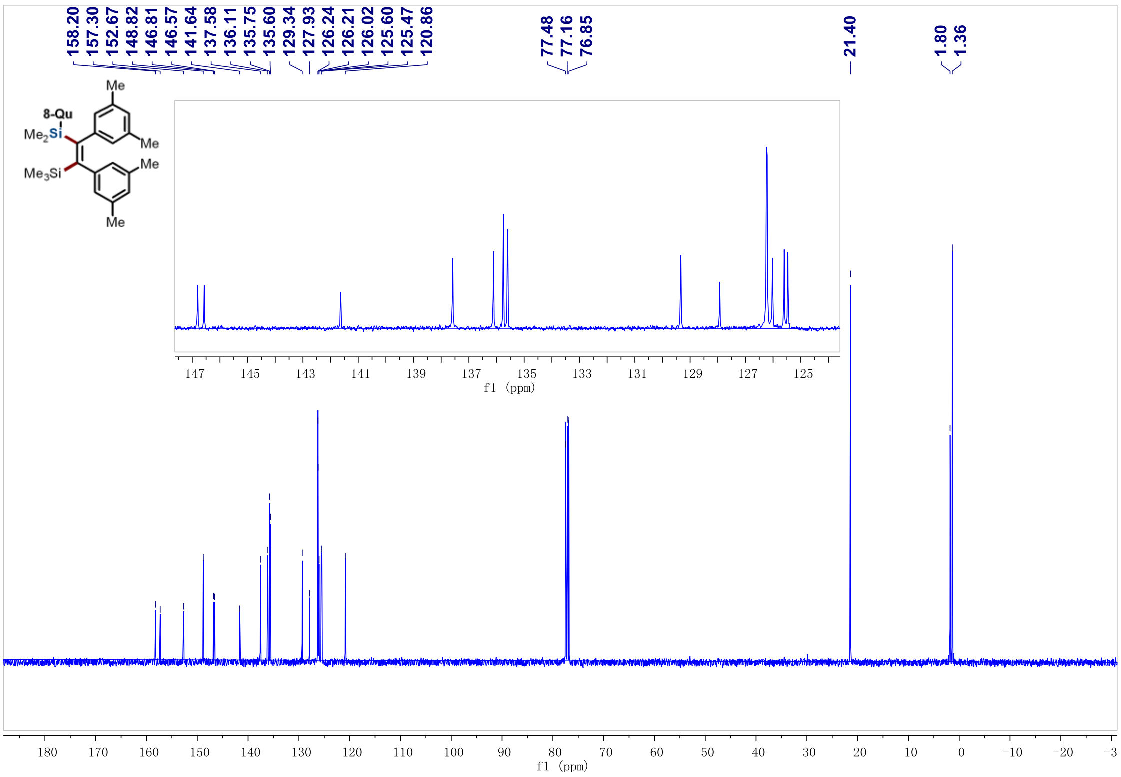

Supplementary Figure 19 ^{1}H and ^{13}C NMR Spectra for compound 3am

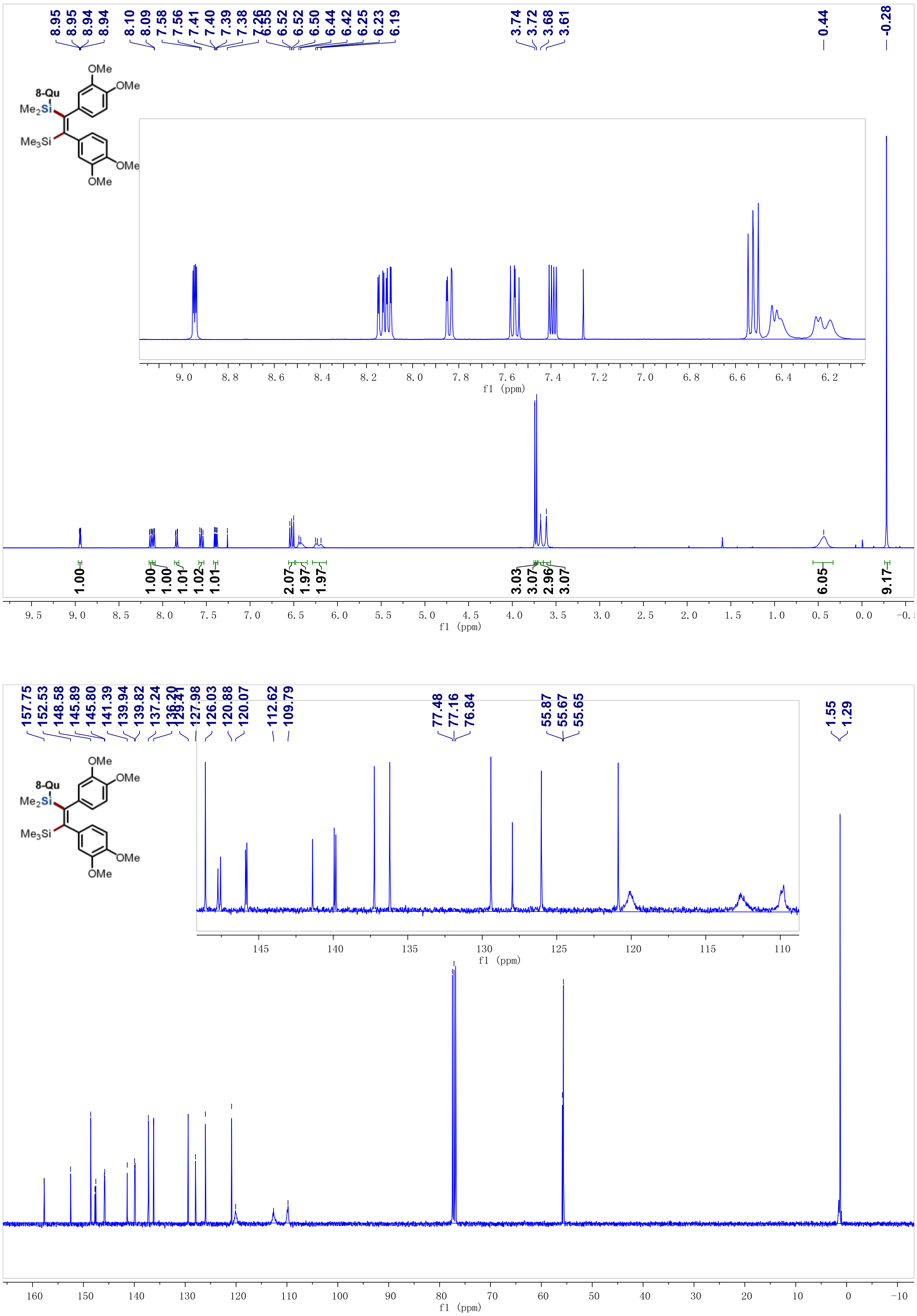

Supplementary Figure 20 ^{1}H and ^{13}C NMR Spectra for compound 3an

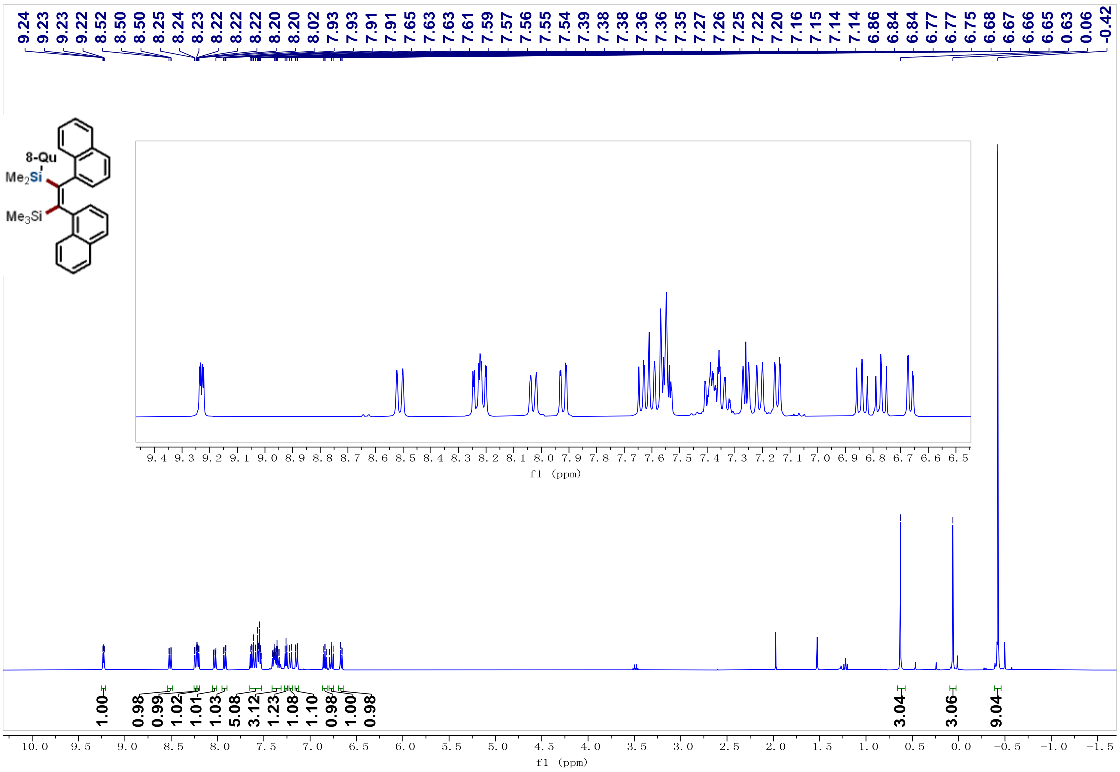

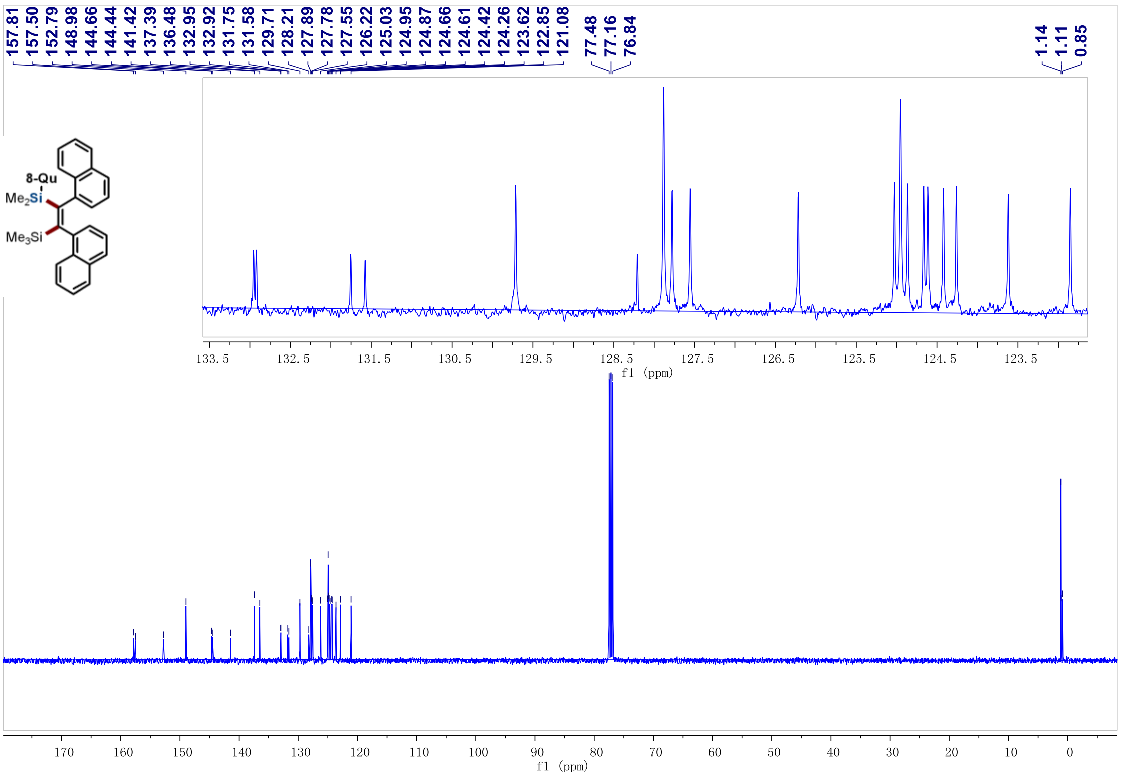

Supplementary Figure 21 ^{1}H and ^{13}C NMR Spectra for compound 3ao

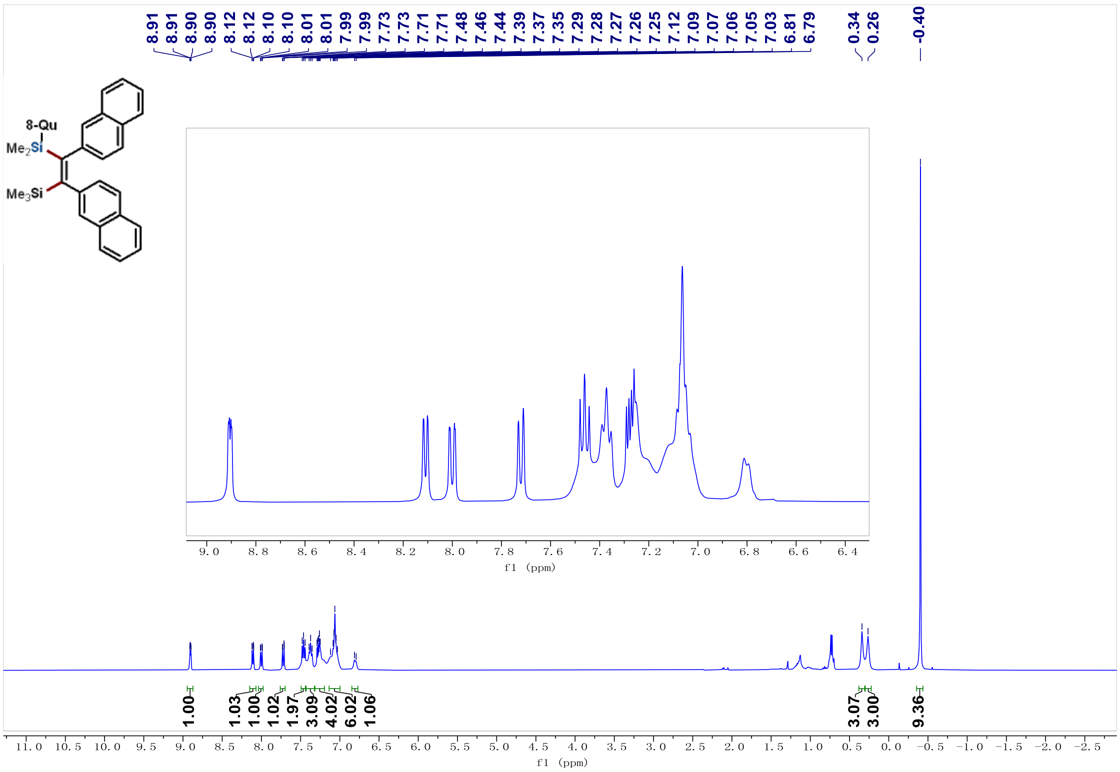

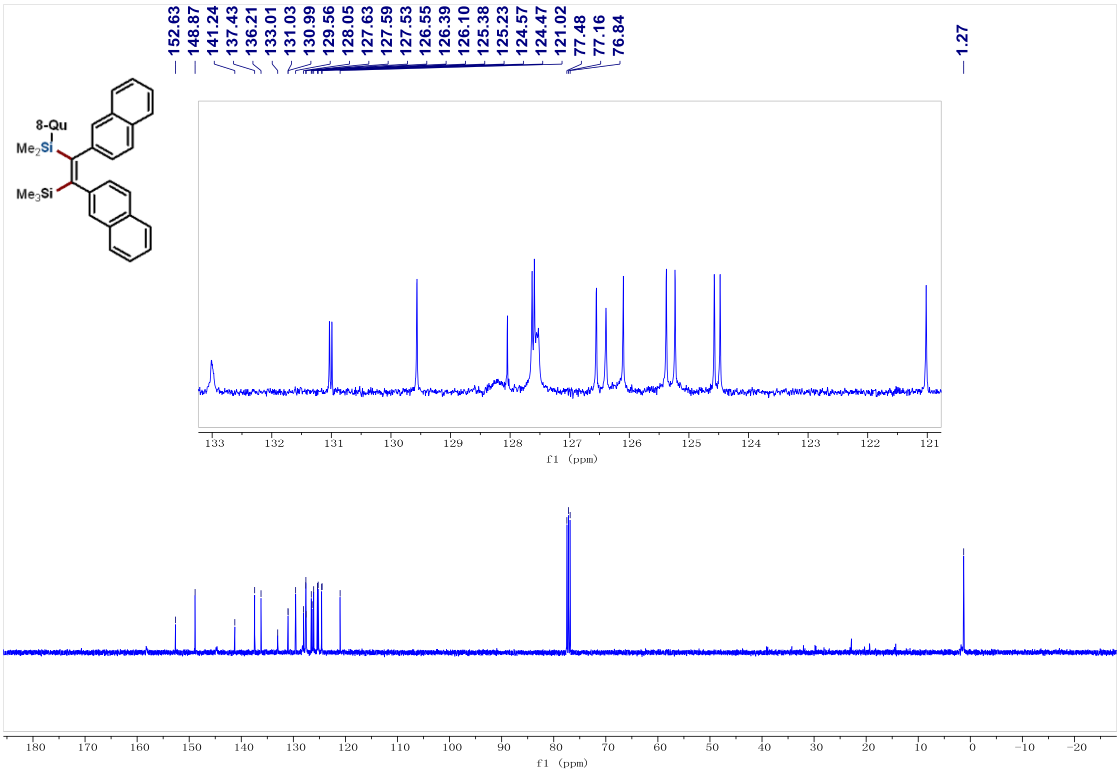

Supplementary Figure 22 ^{1}H and ^{13}C NMR Spectra for compound 3ap

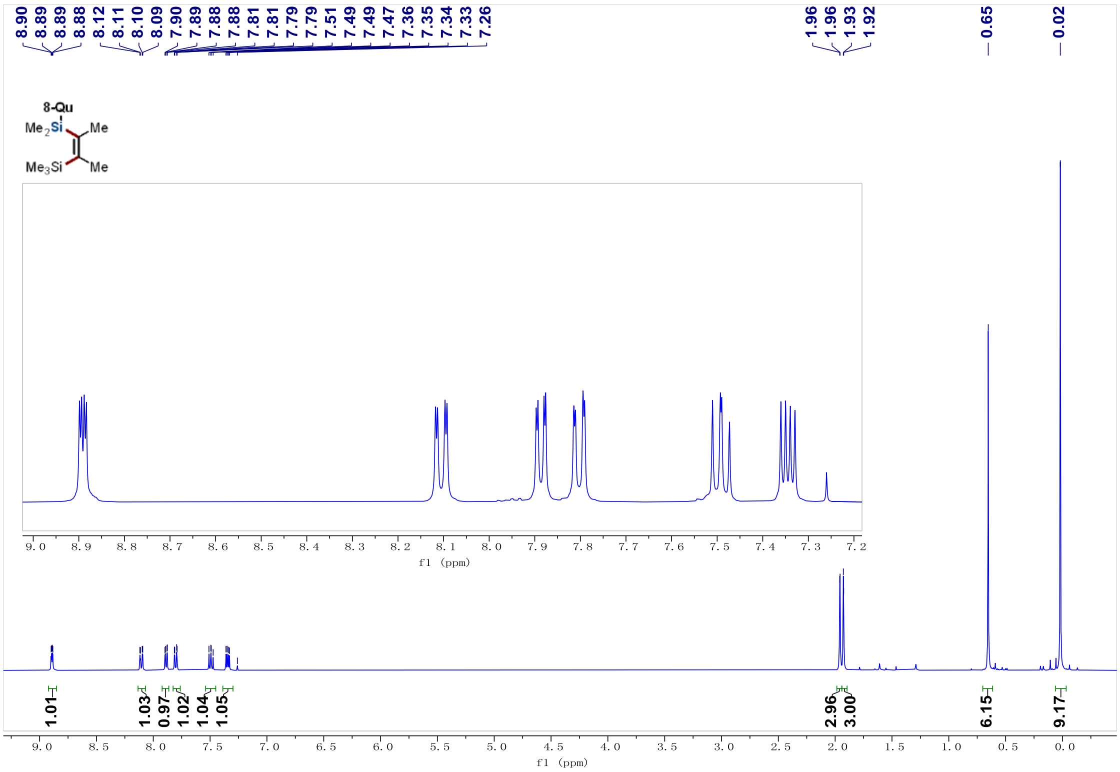

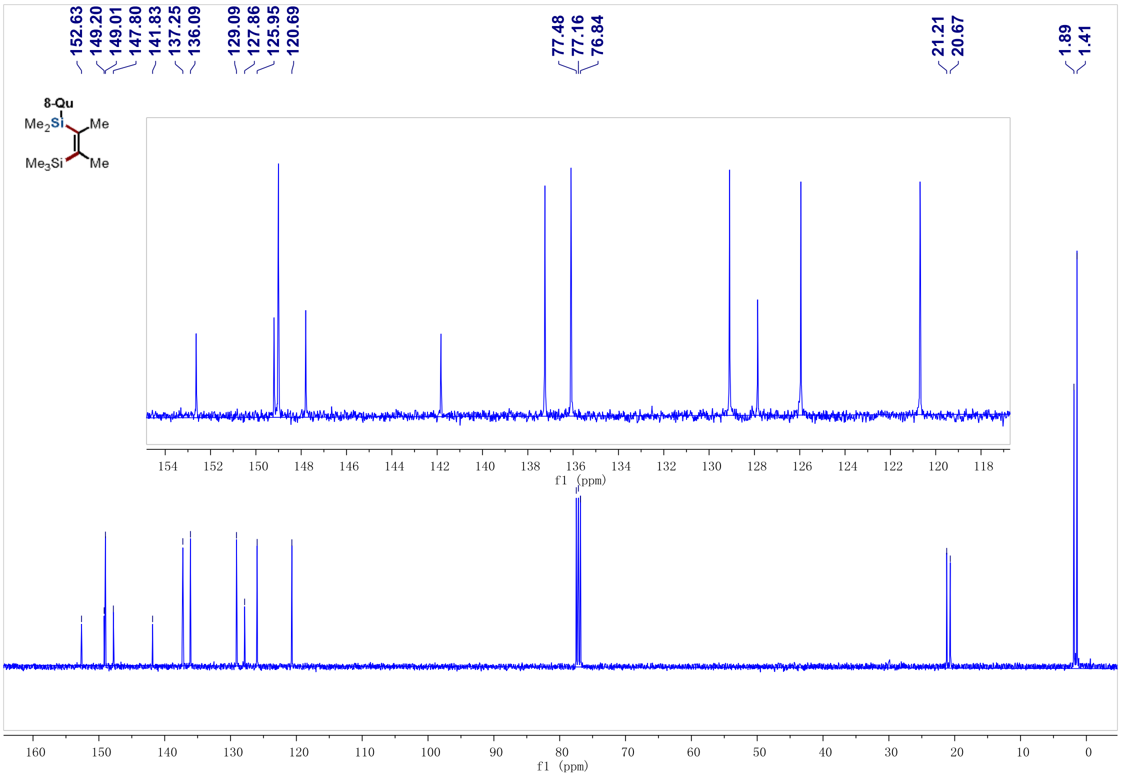

Supplementary Figure 23 ^{1}H and ^{13}C NMR Spectra for compound 3aq

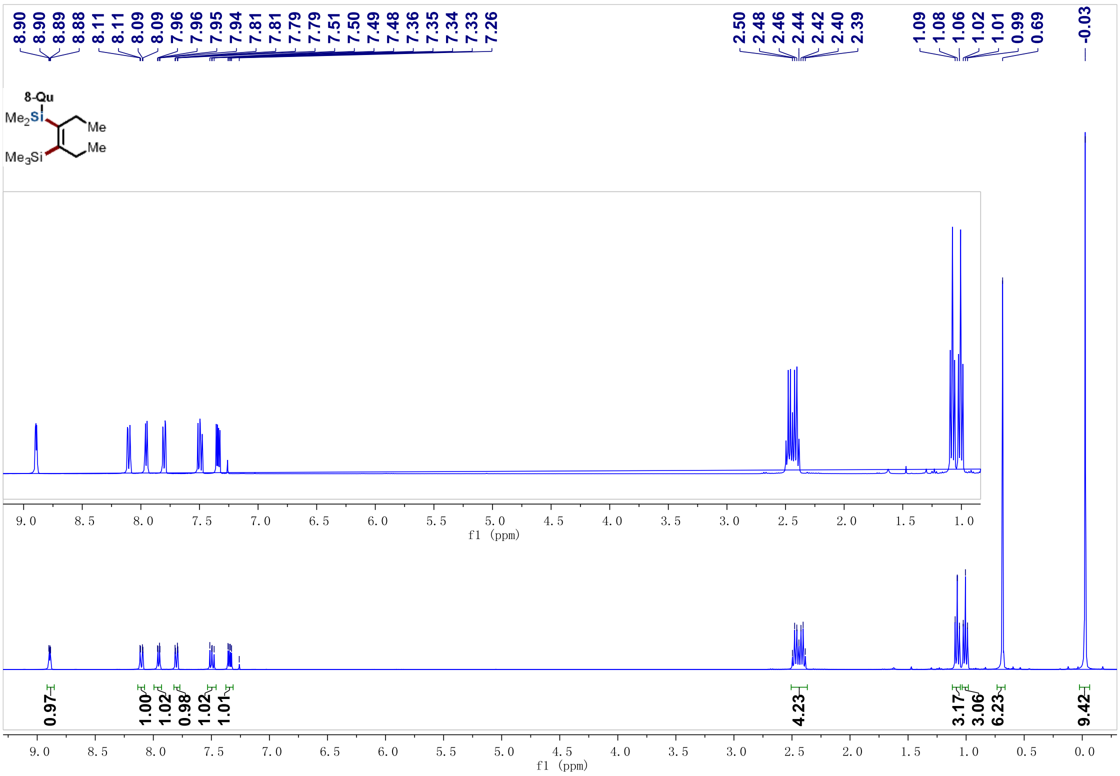

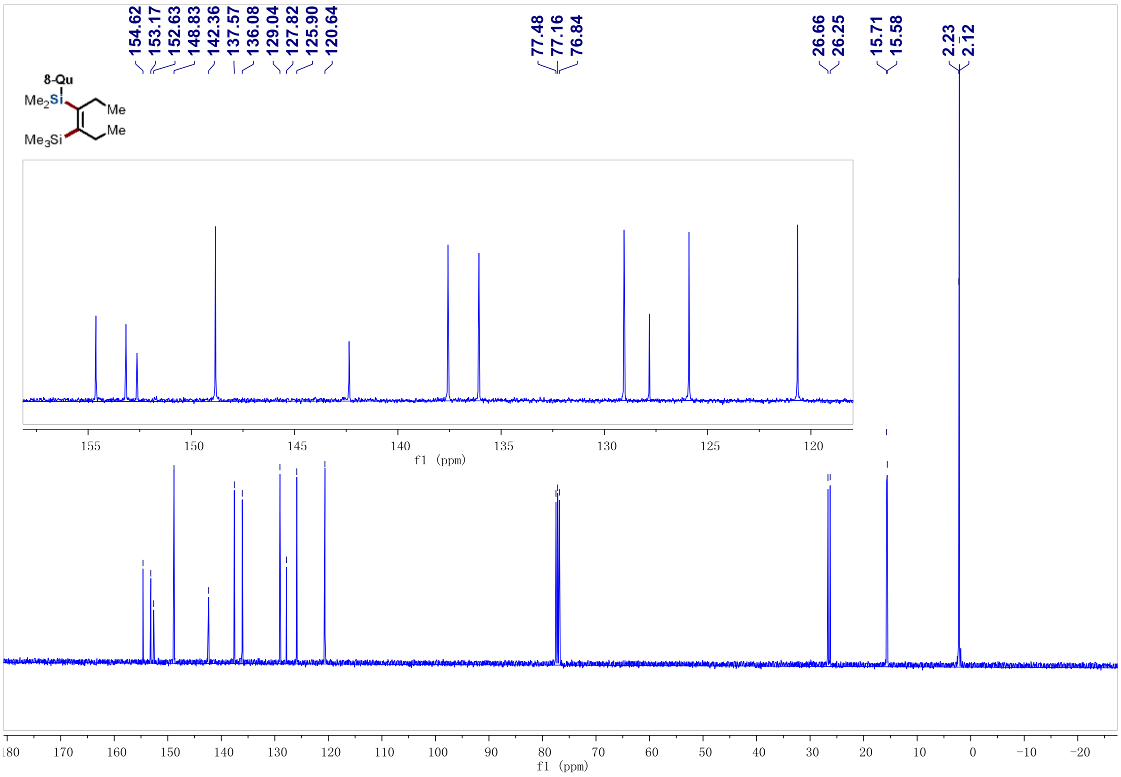

Supplementary Figure 24 ^{1}H and ^{13}C NMR Spectra for compound 3ar

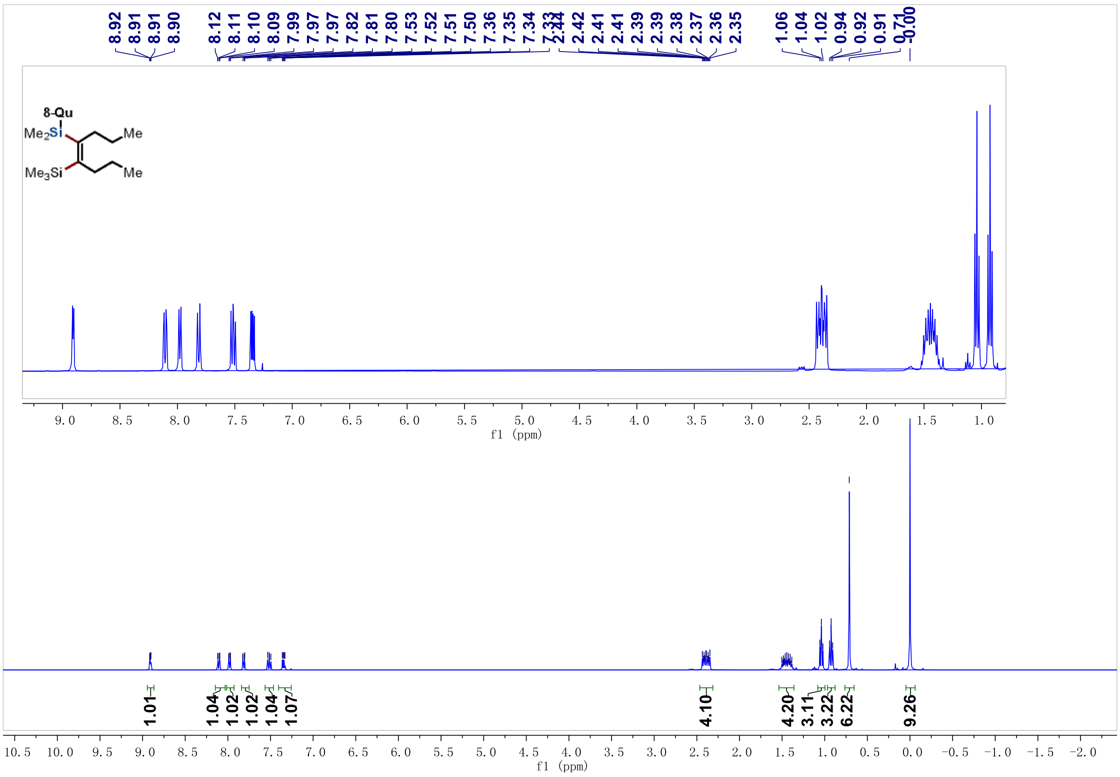

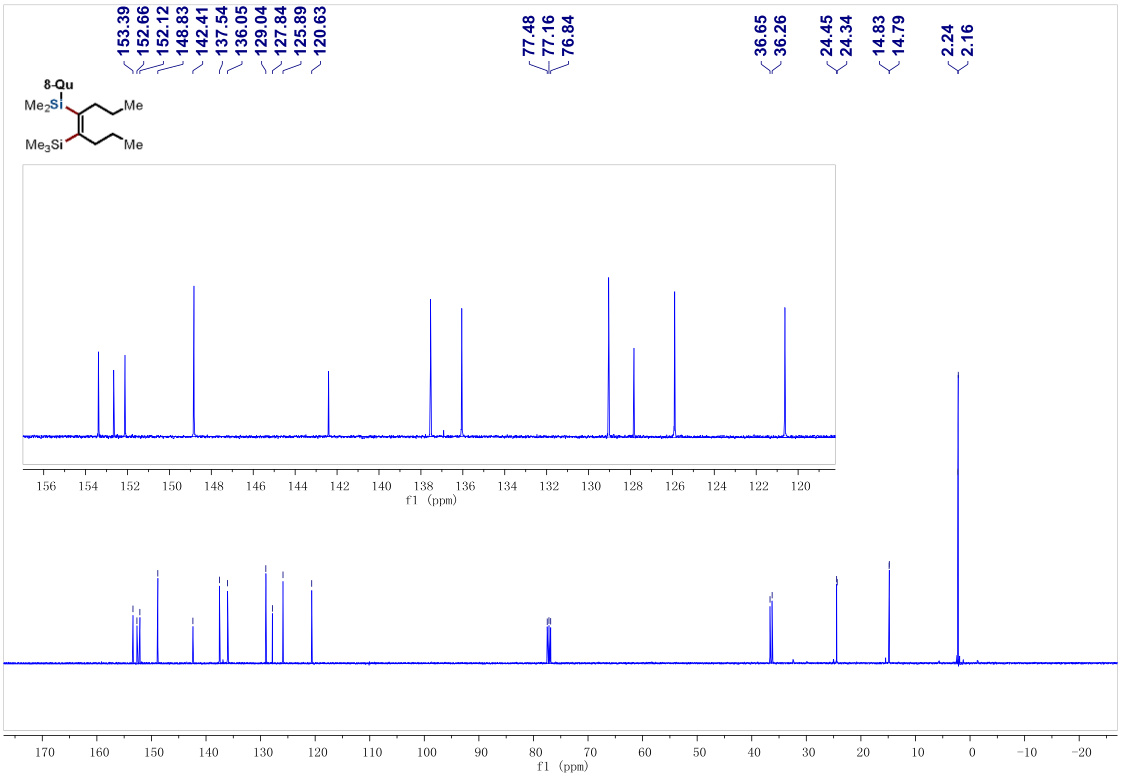

Supplementary Figure 25 ^{1}H and ^{13}C NMR Spectra for compound 3as

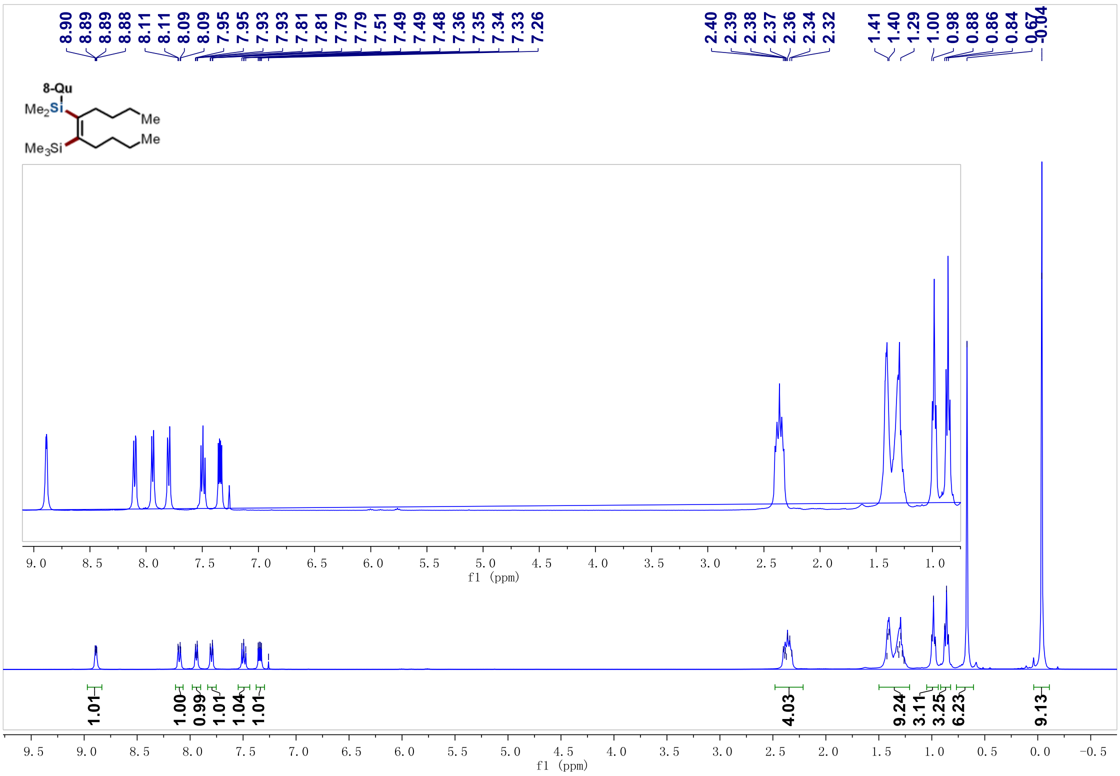

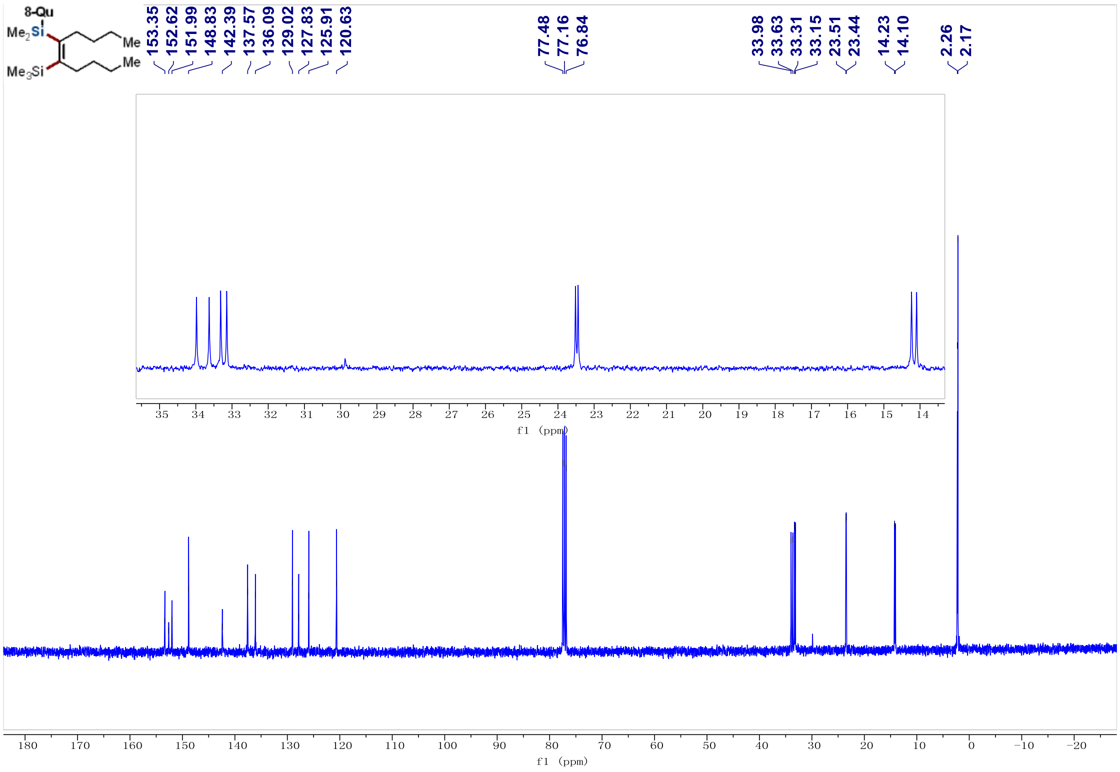

Supplementary Figure 26 ^{1}H and ^{13}C NMR Spectra for compound 3at

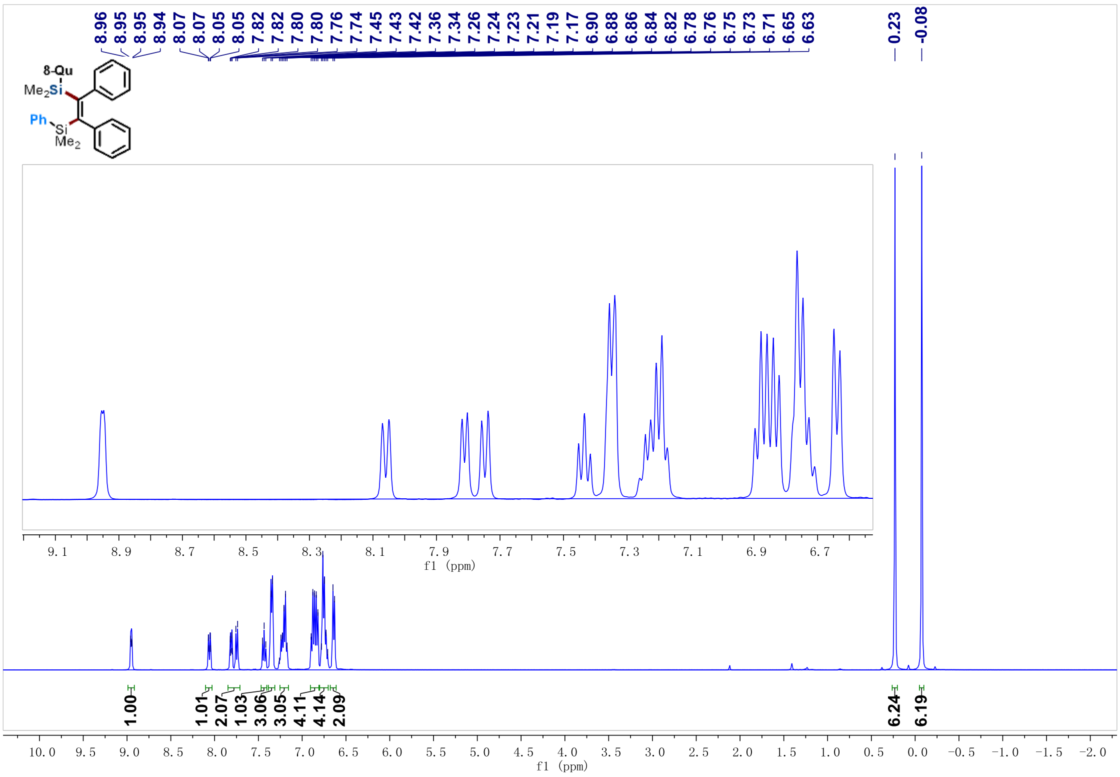

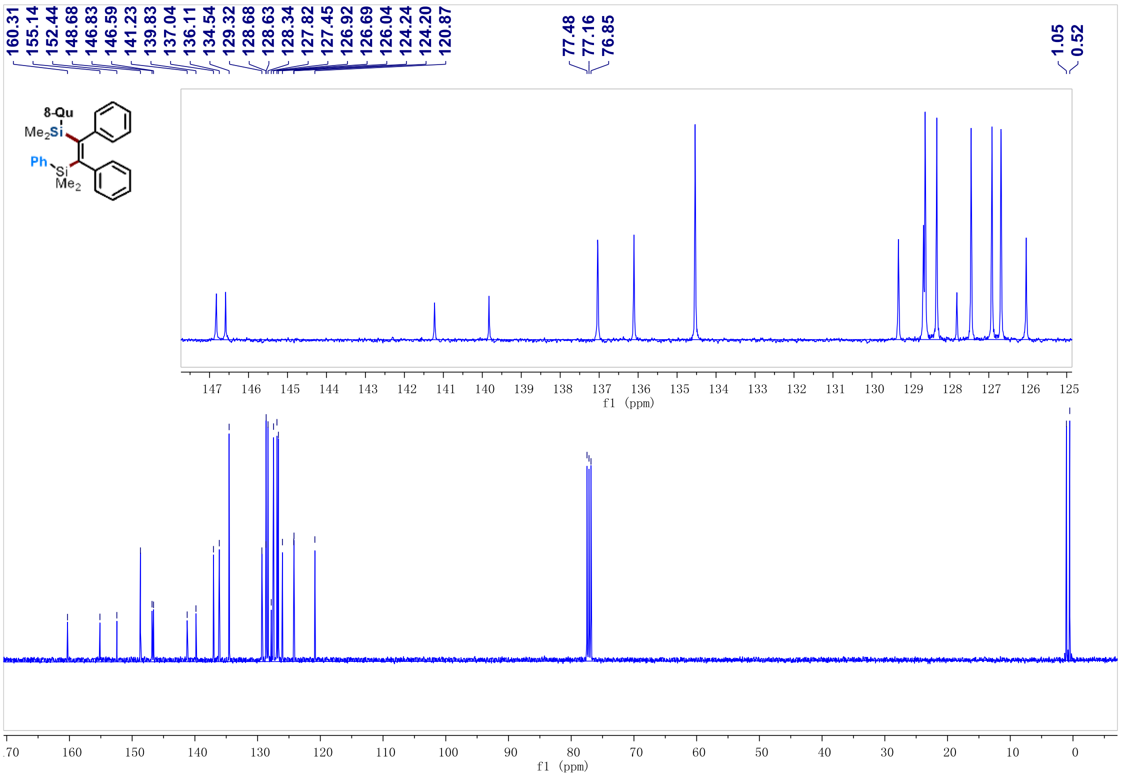

Supplementary Figure 27 ^{1}H and ^{13}C NMR Spectra for compound 3ba

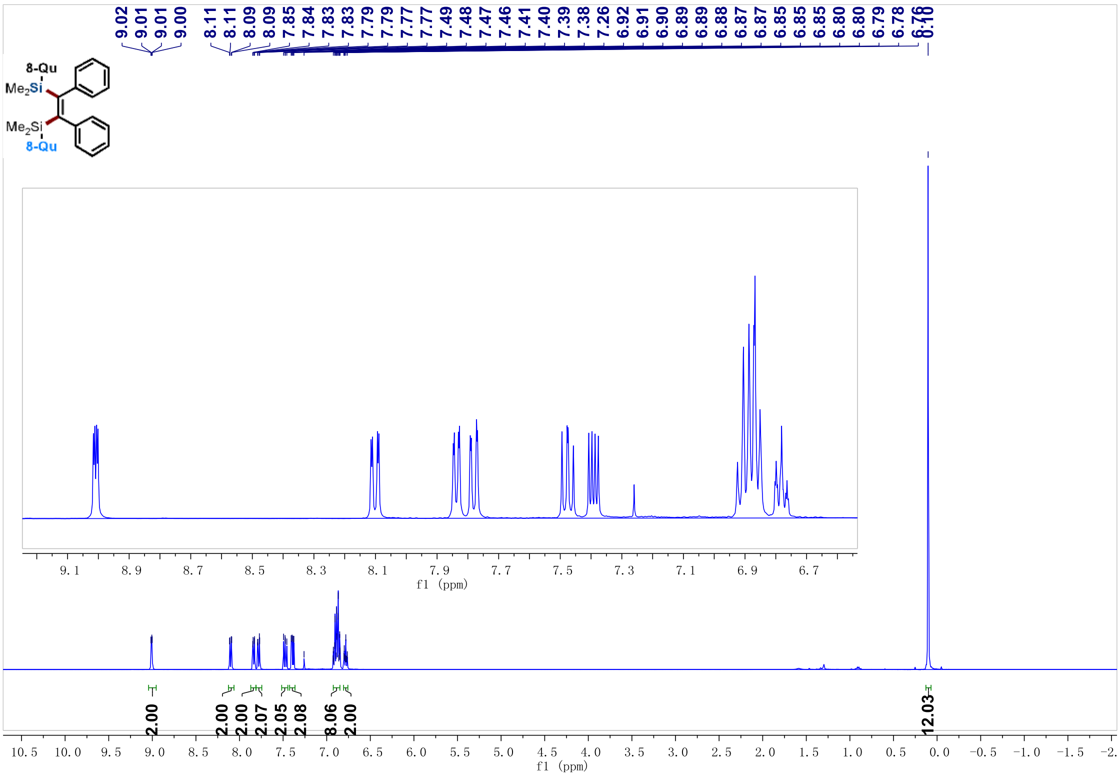

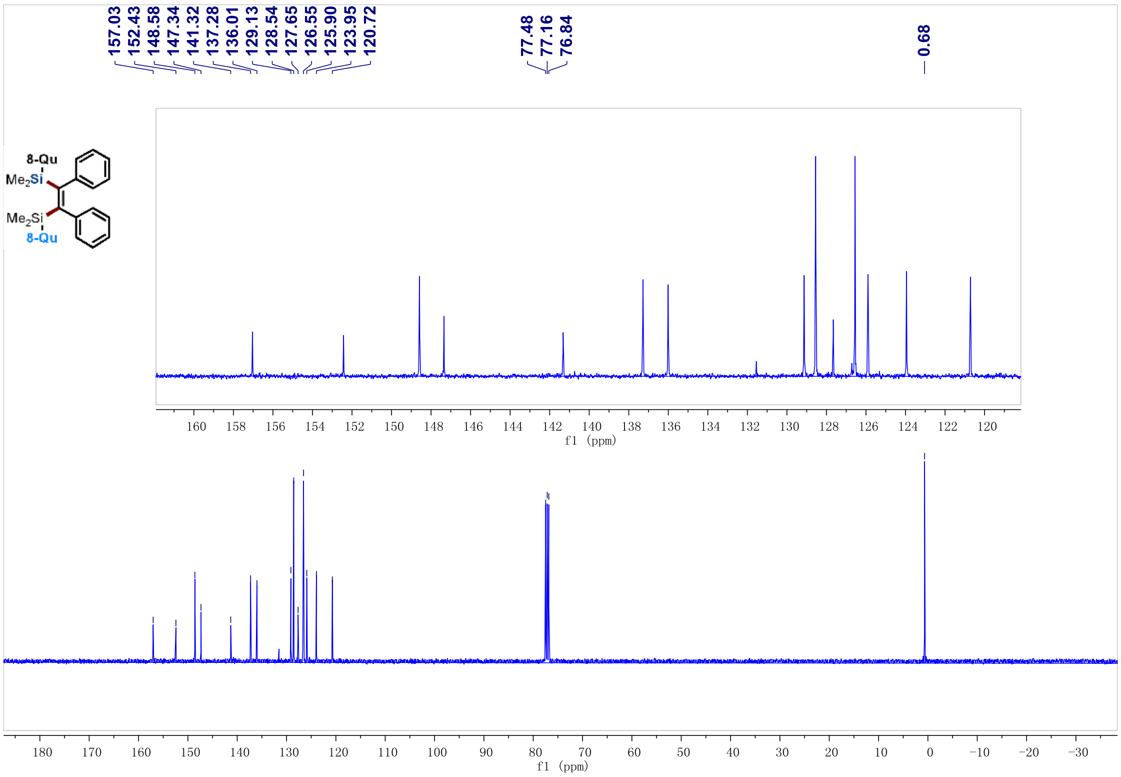

Supplementary Figure 28 ^{1}H and ^{13}C NMR Spectra for compound 3ca

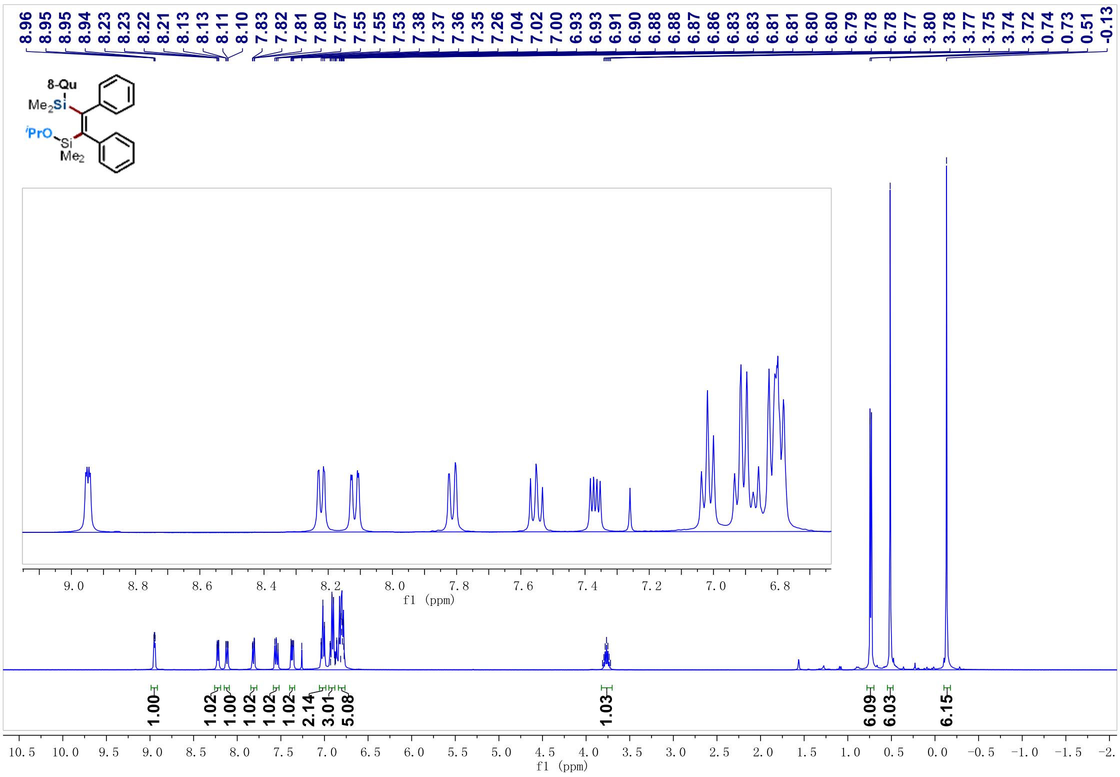

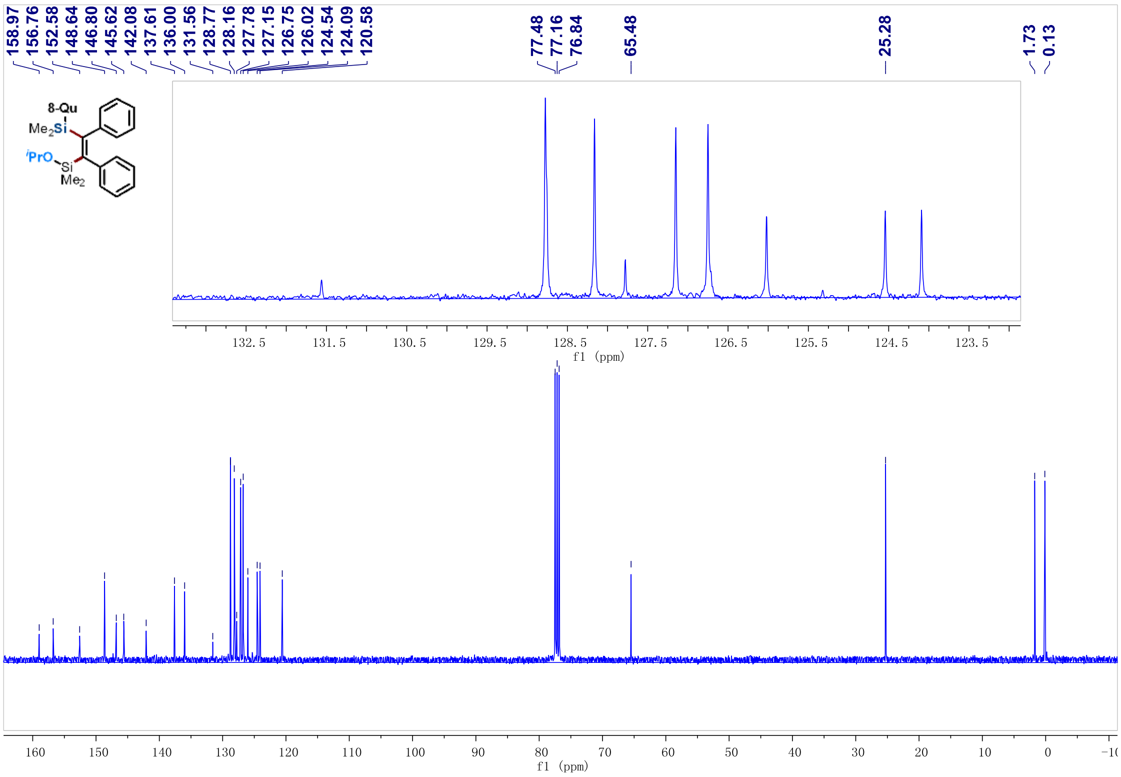

Supplementary Figure 29 ^{1}H and ^{13}C NMR Spectra for compound 3da

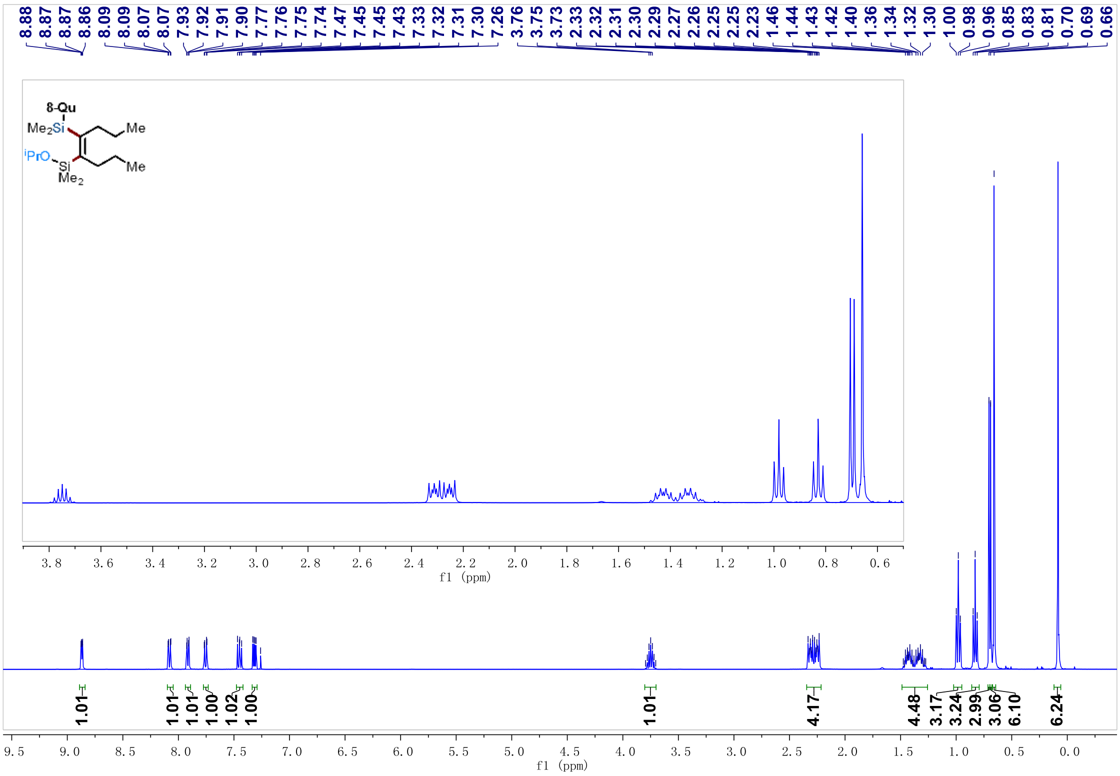

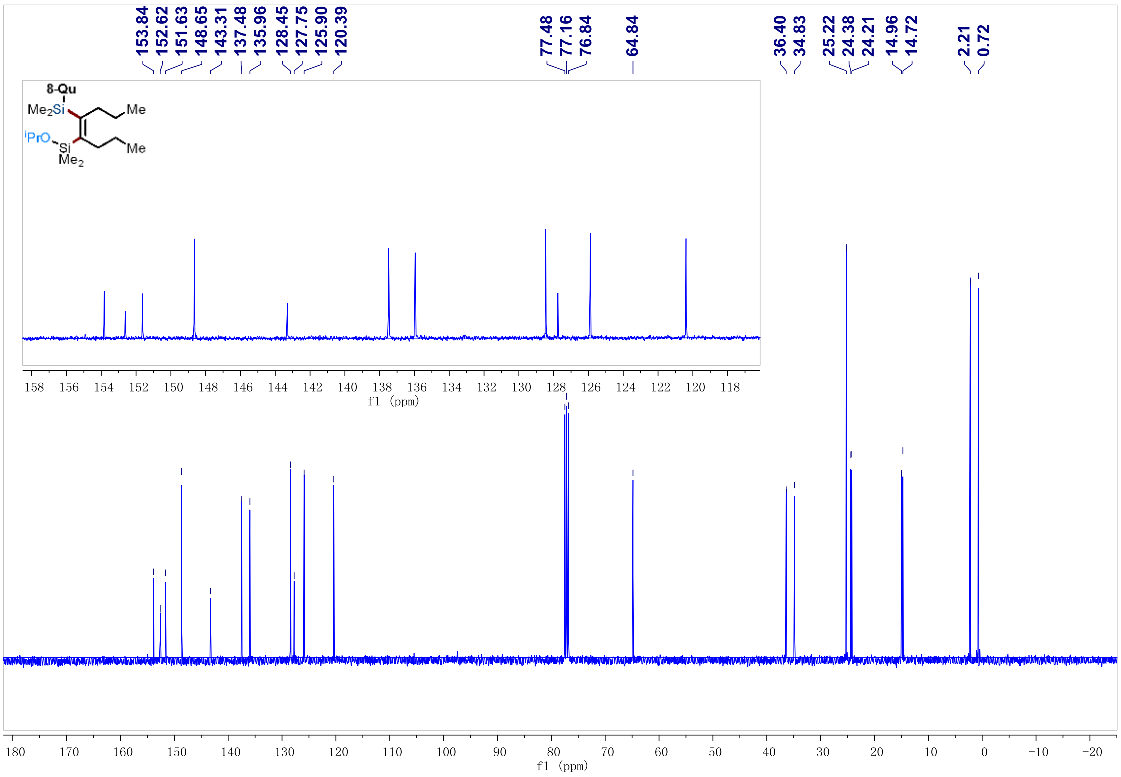

Supplementary Figure 30 ^{1}H and ^{13}C NMR Spectra for compound 3db

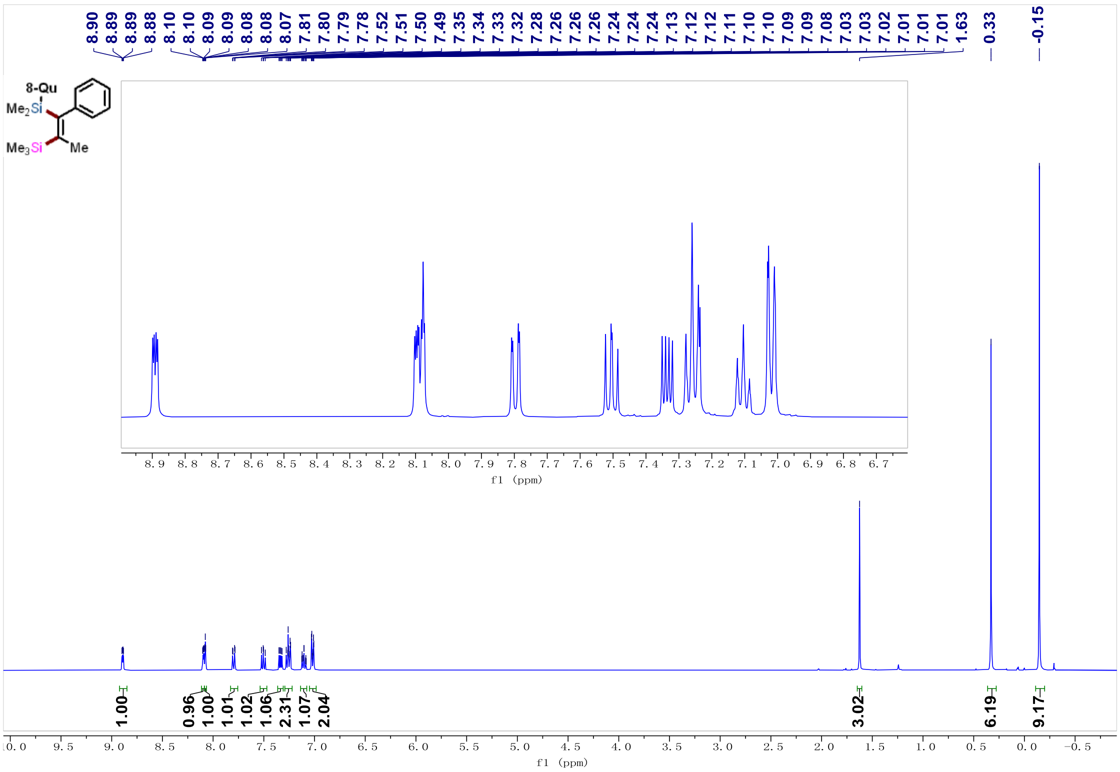

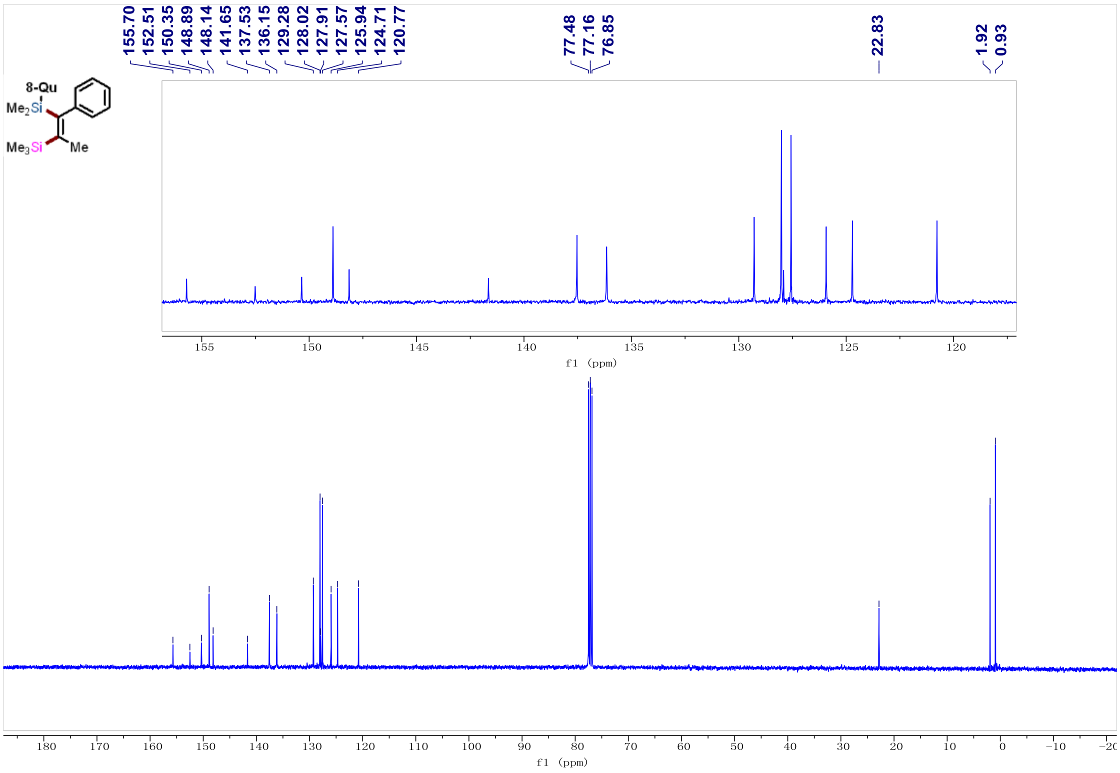

Supplementary Figure 31 ^{1}H and ^{13}C NMR Spectra for compound 3au

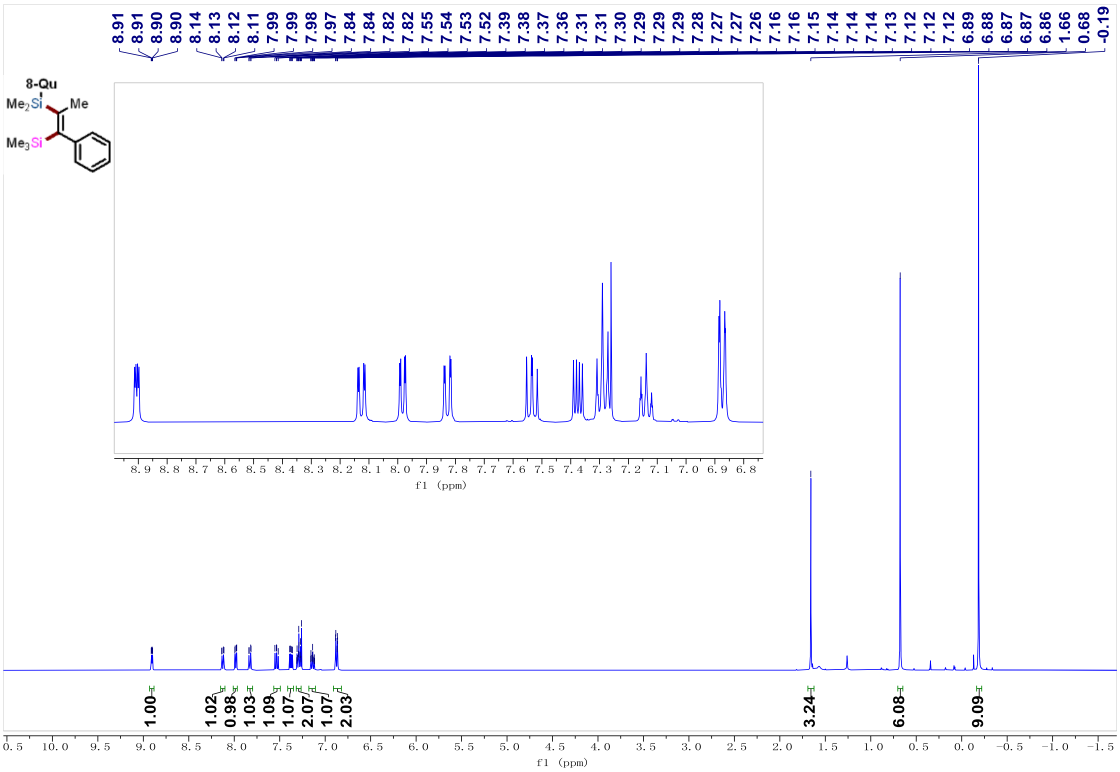

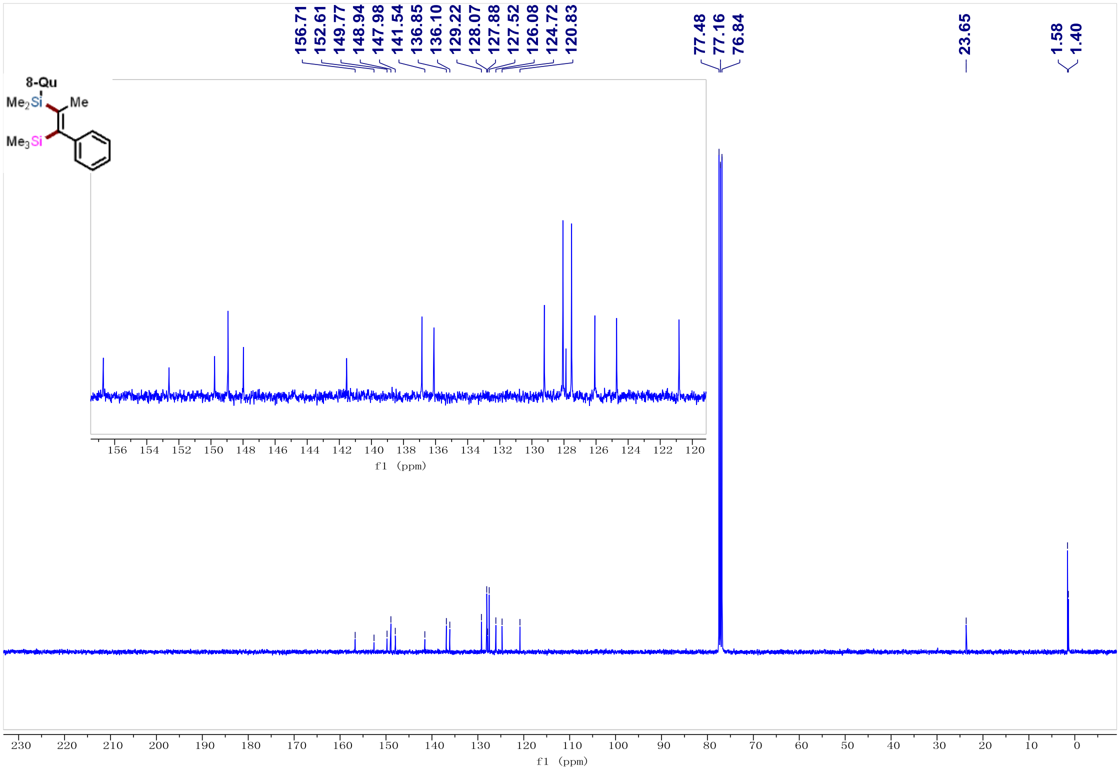

Supplementary Figure 32 ^{1}H and ^{13}C NMR Spectra for compound 3au′

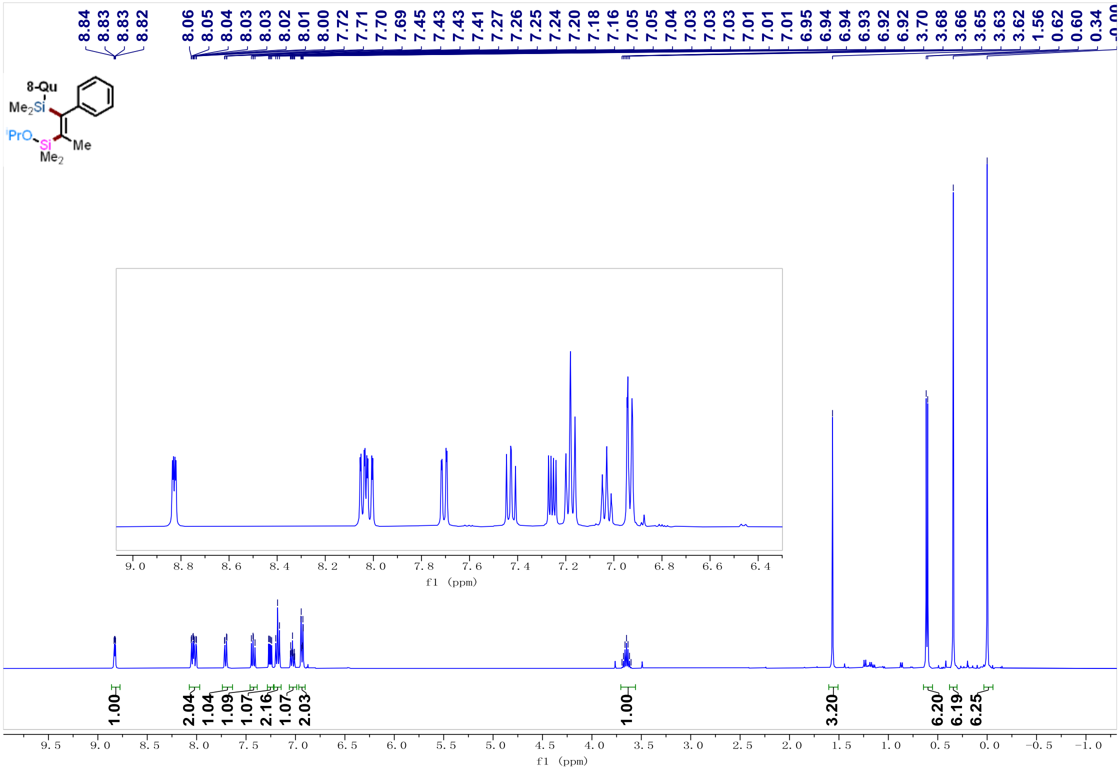

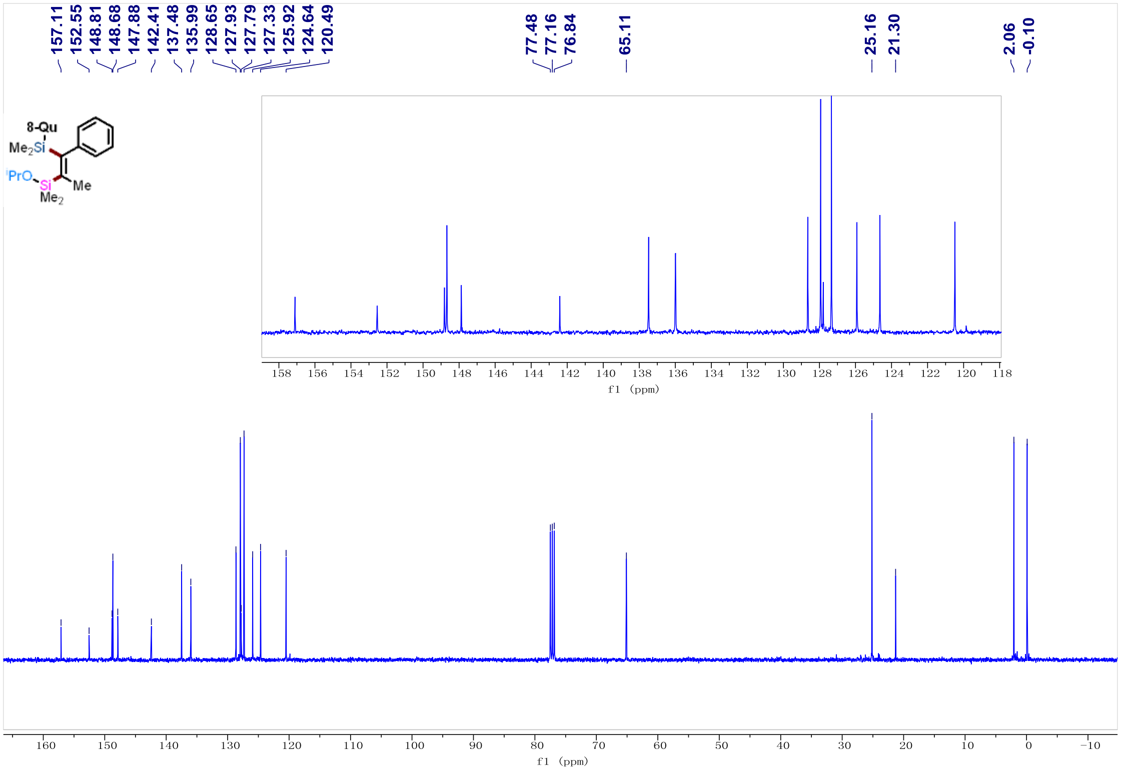

Supplementary Figure 33 ^{1}H and ^{13}C NMR Spectra for compound 3du

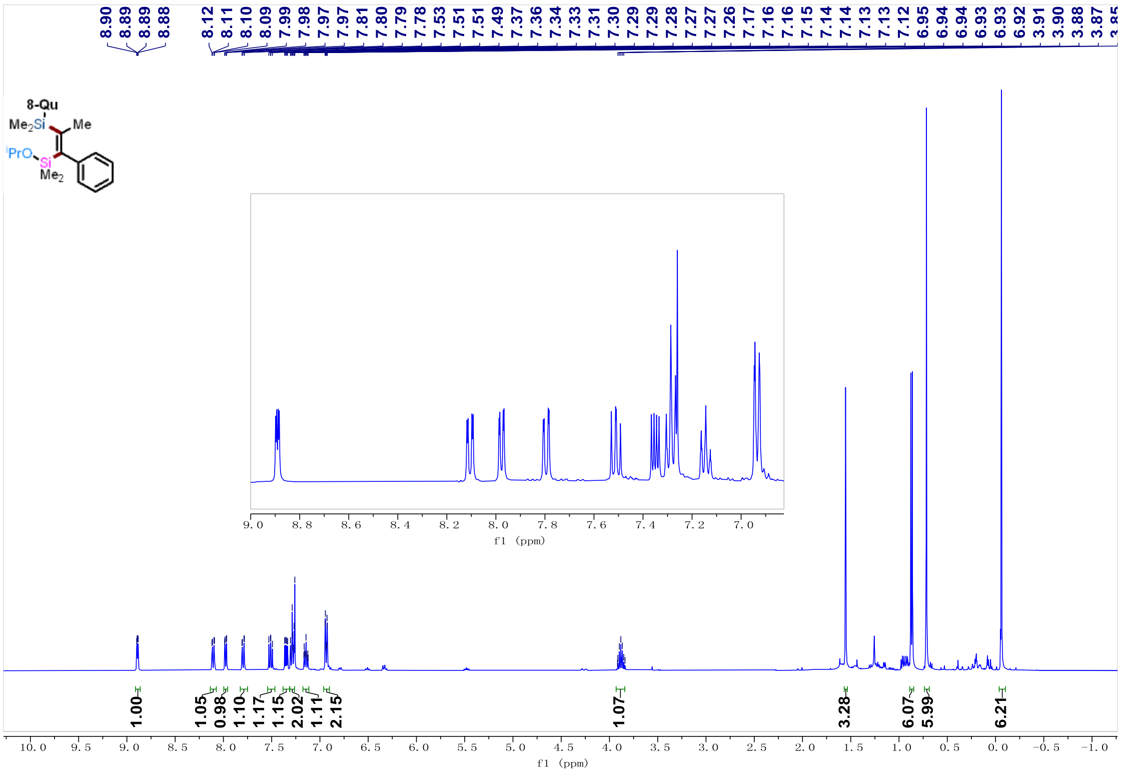

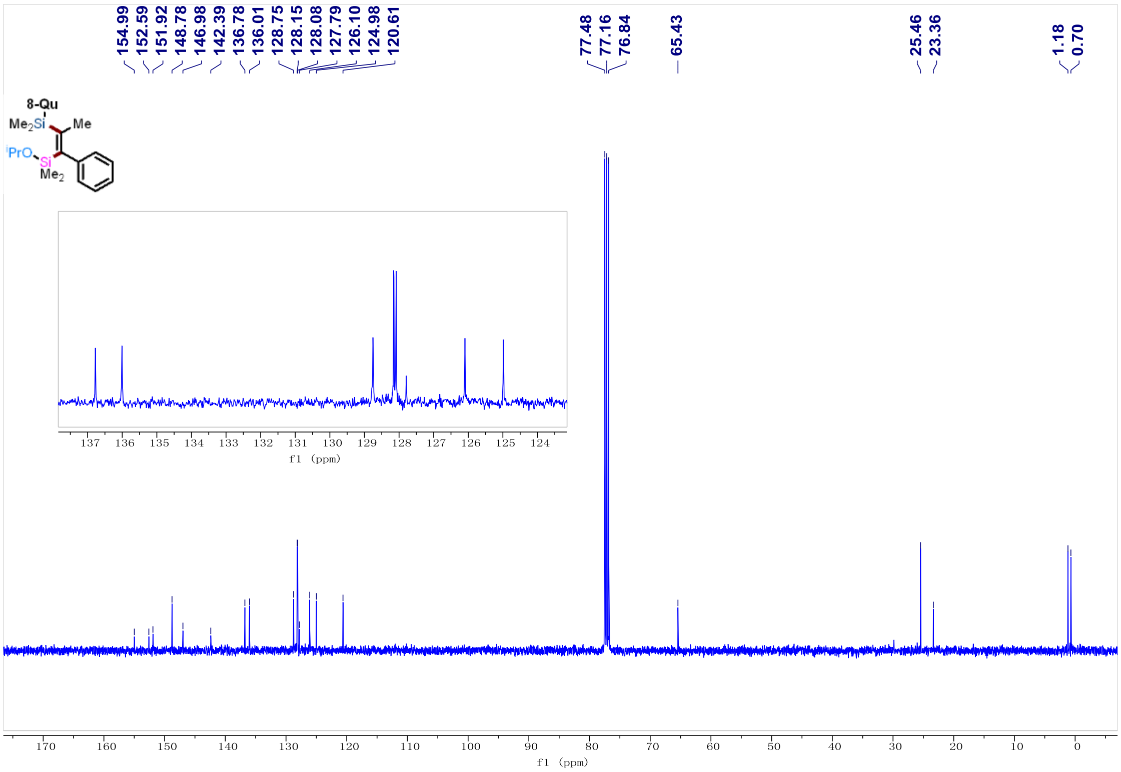

Supplementary Figure 34 ^{1}H and ^{13}C NMR Spectra for compound 3du′

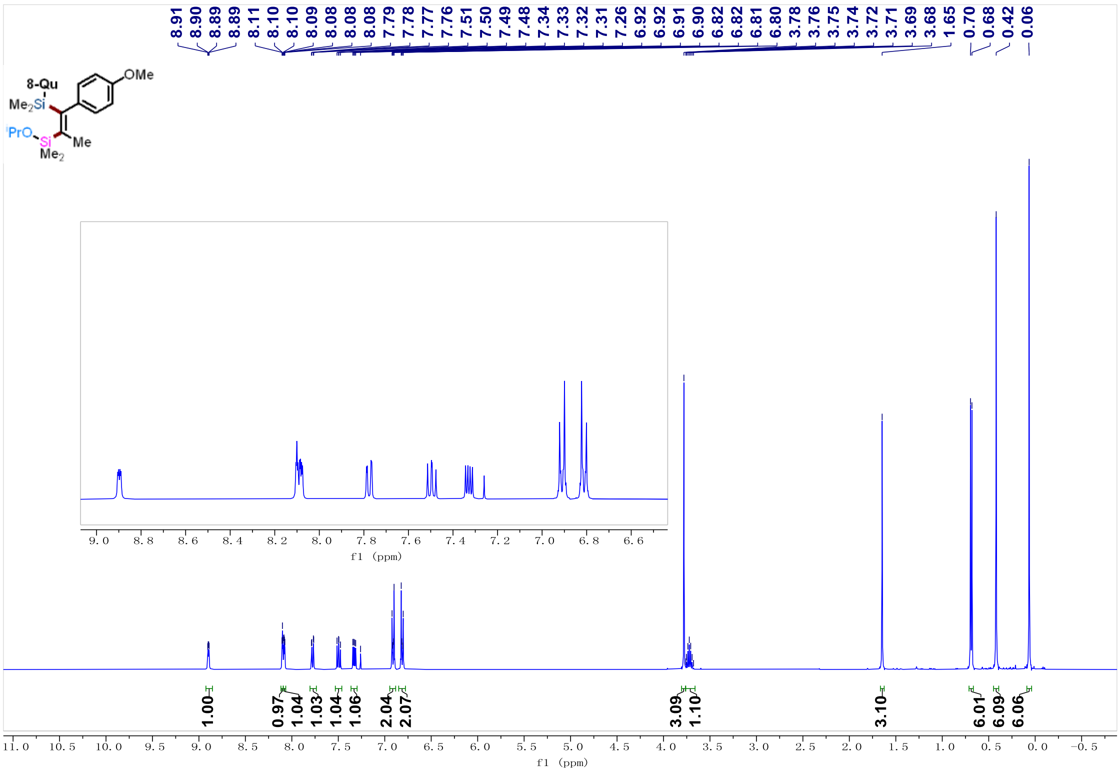

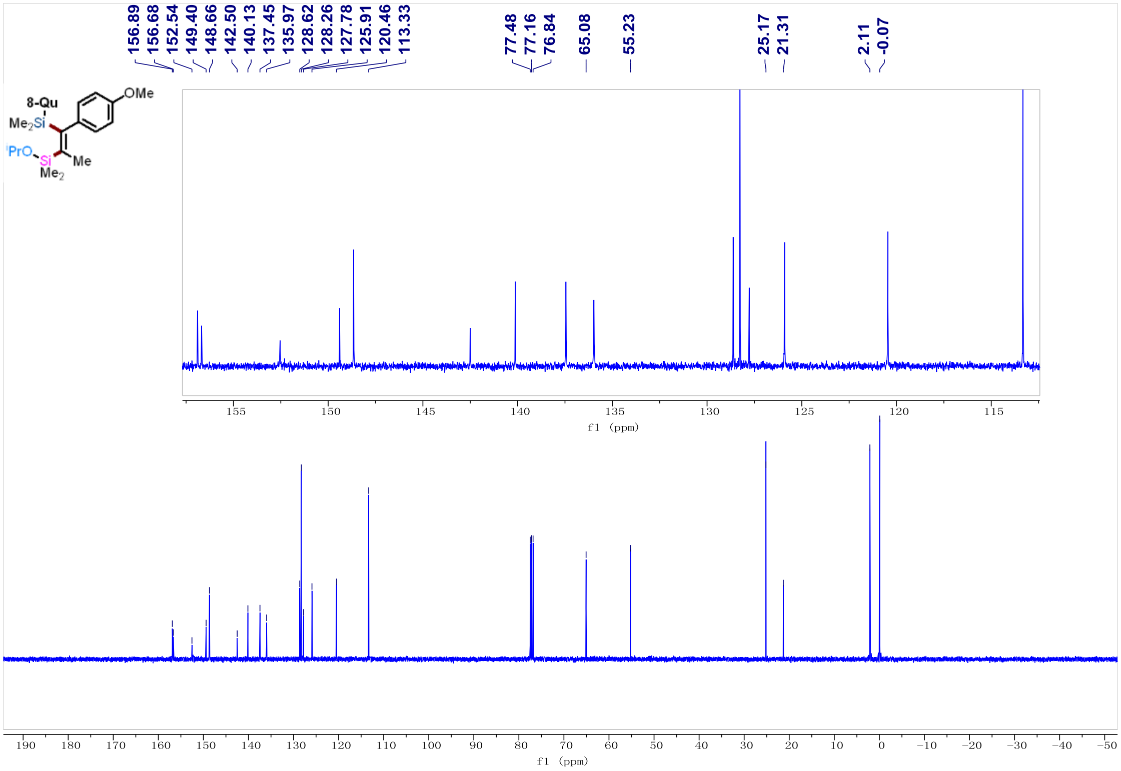

Supplementary Figure 35 ^{1}H and ^{13}C NMR Spectra for compound 3dv

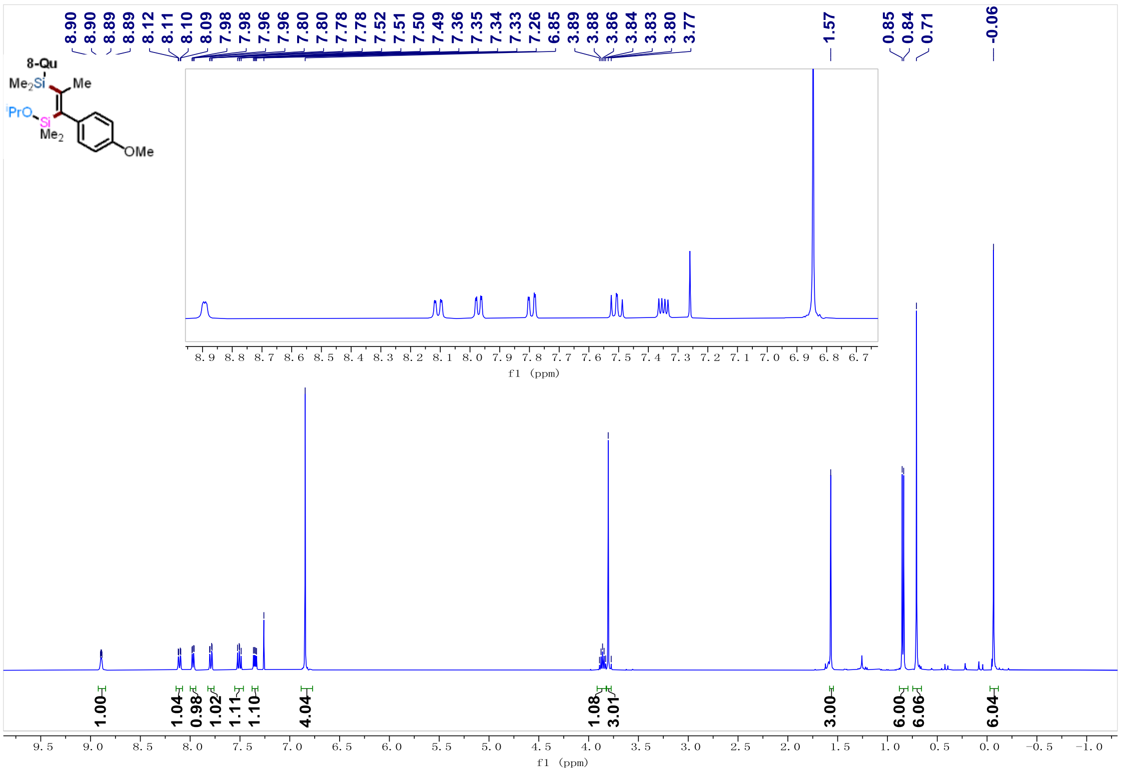

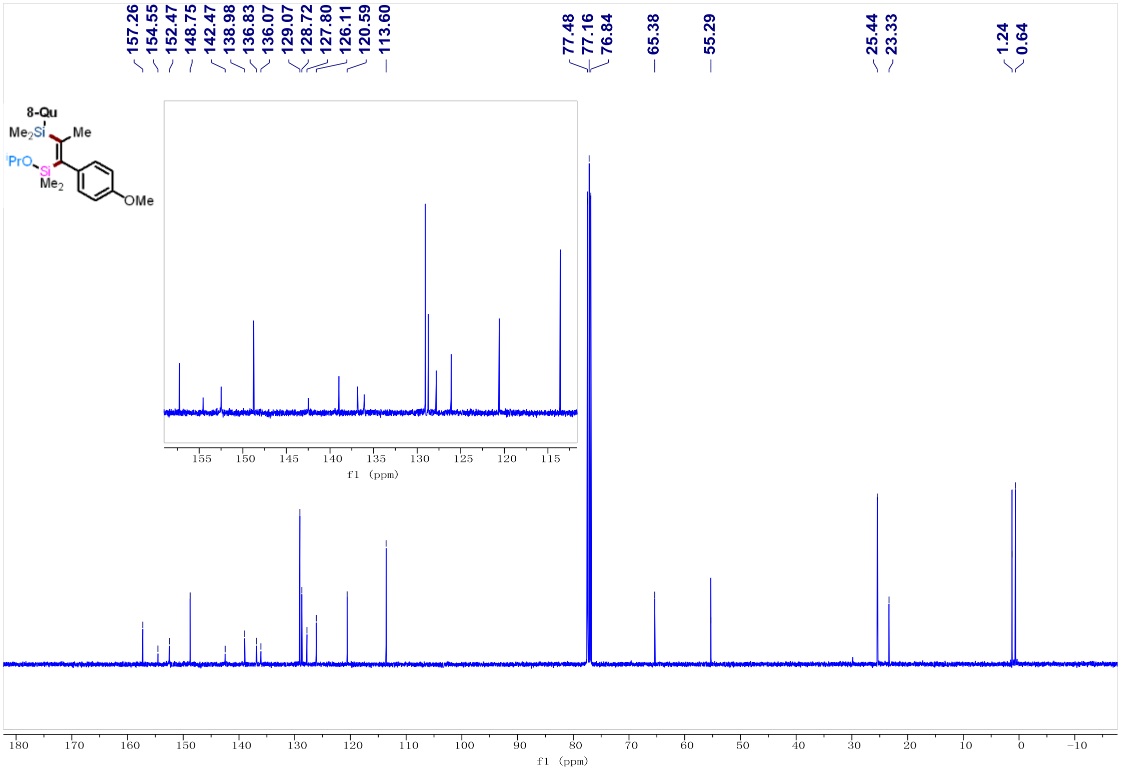

Supplementary Figure 36 ^{1}H and ^{13}C NMR Spectra for compound 3dv′

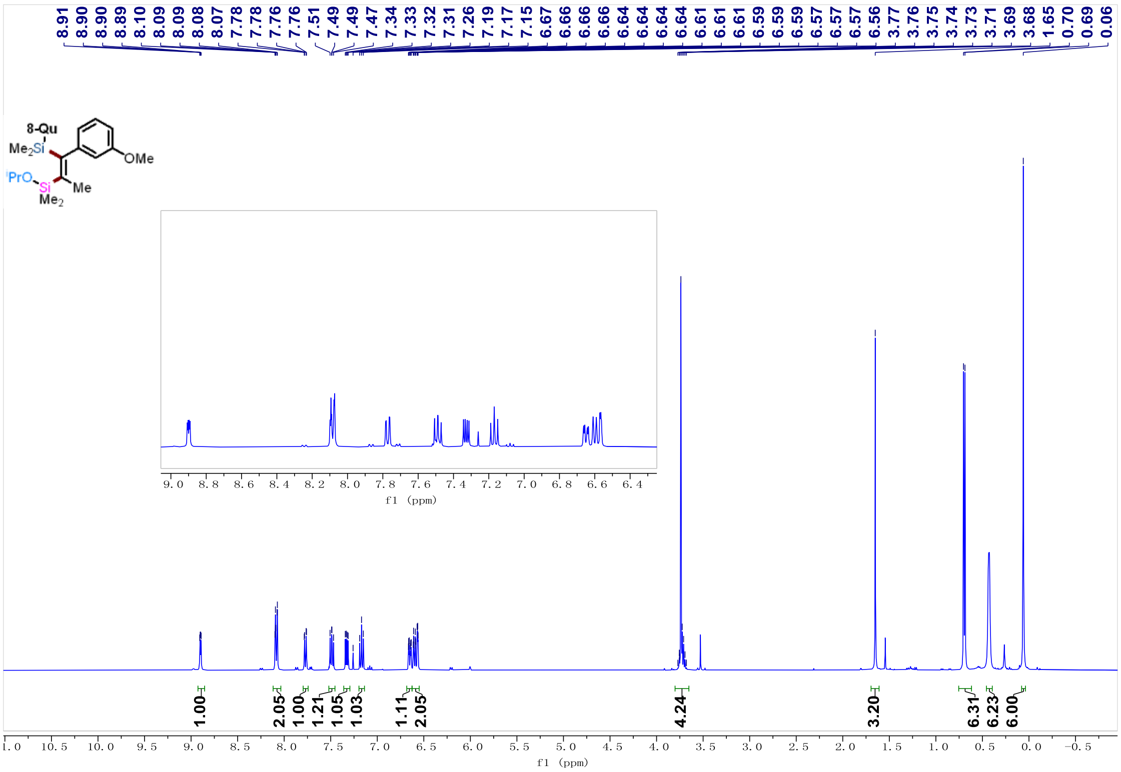

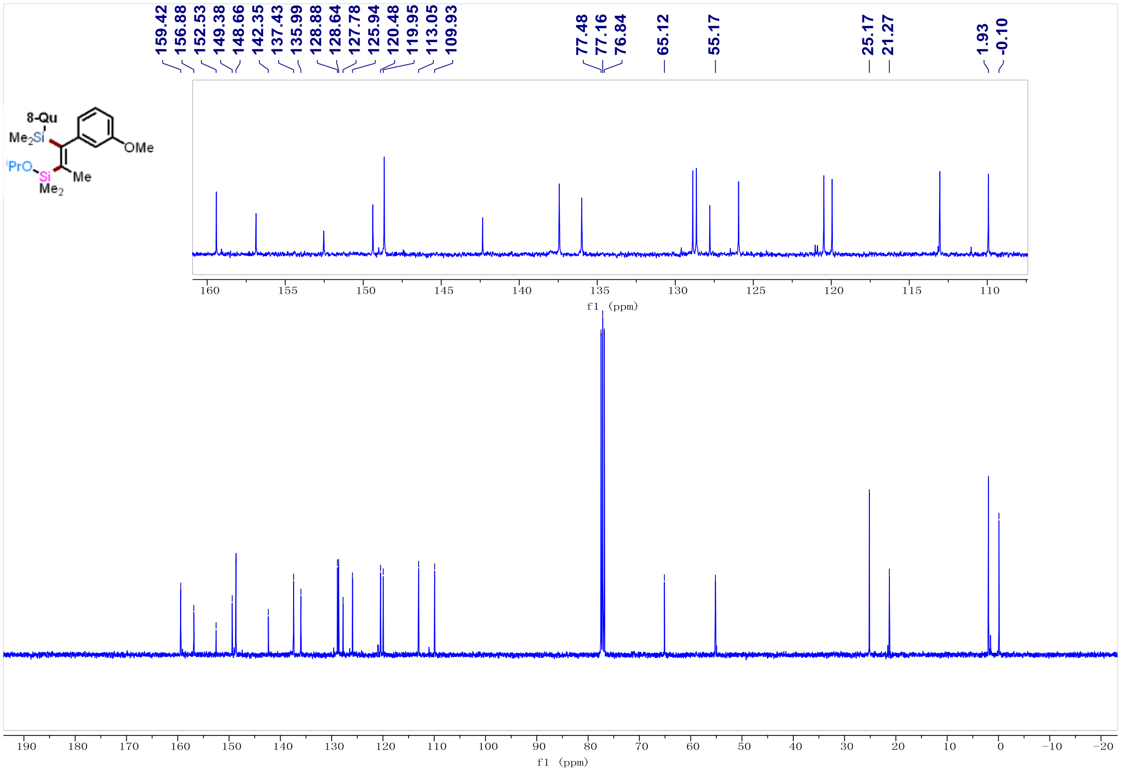

Supplementary Figure 37 ^{1}H and ^{13}C NMR Spectra for compound 3dw

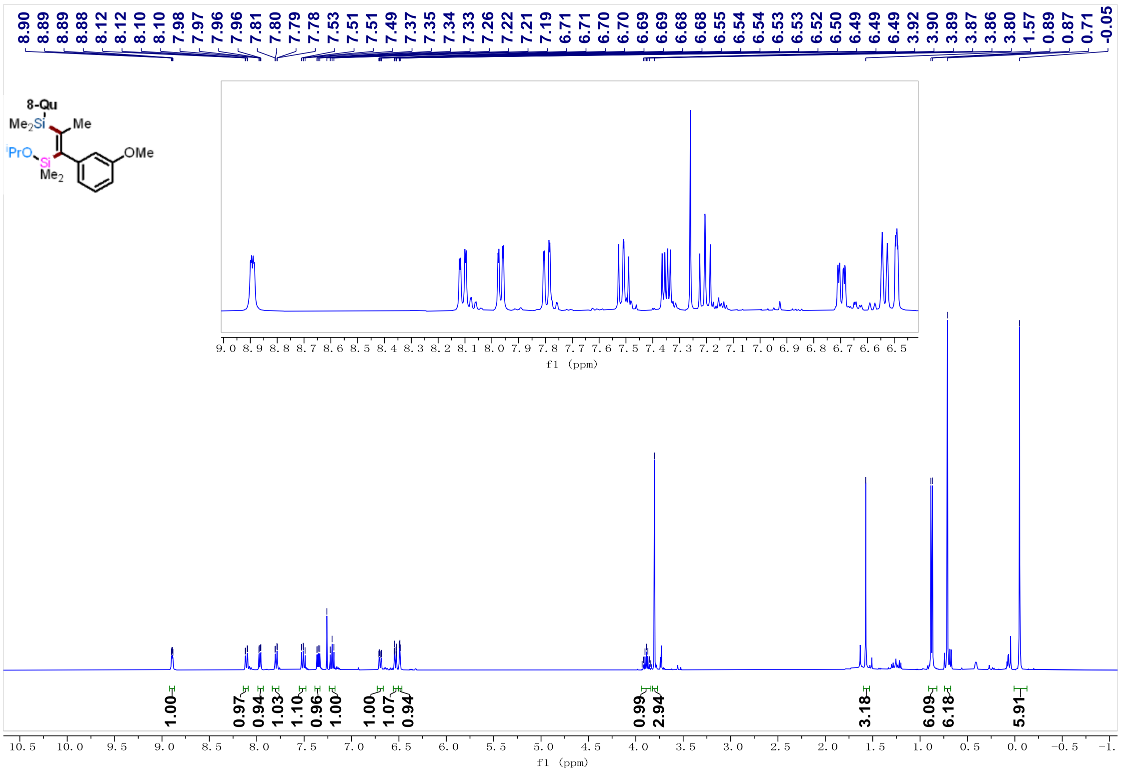

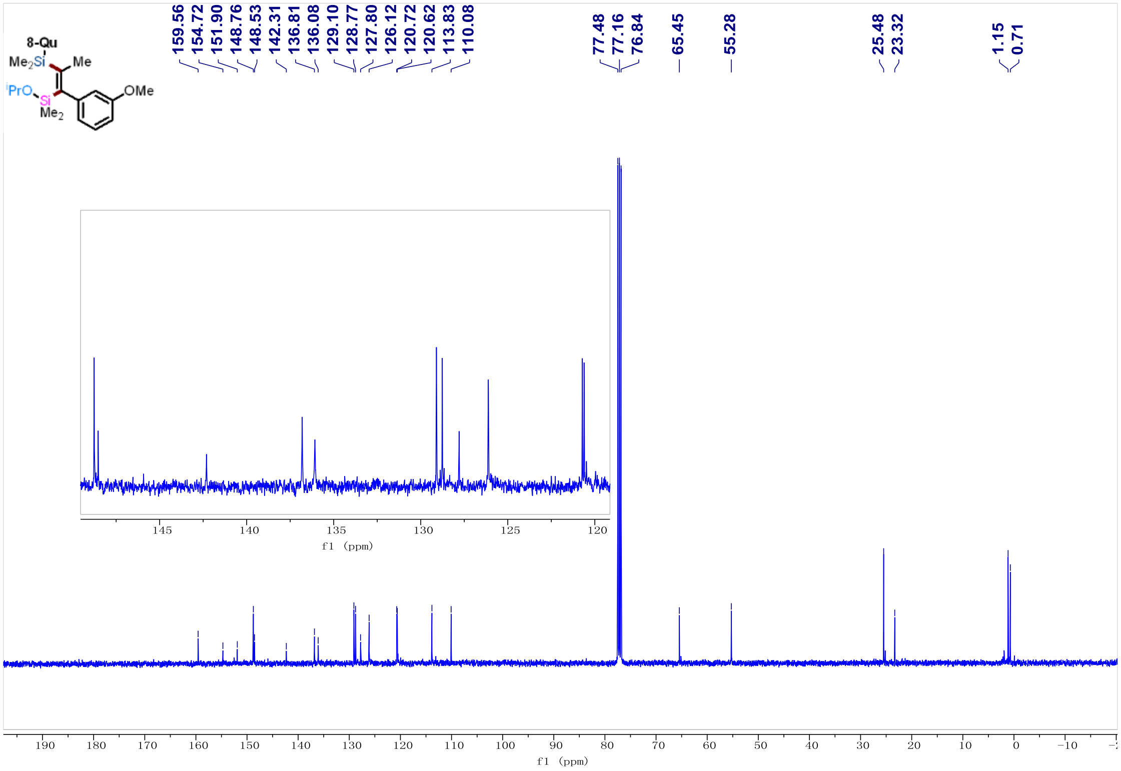

Supplementary Figure 38 ^{1}H and ^{13}C NMR Spectra for compound 3dw′

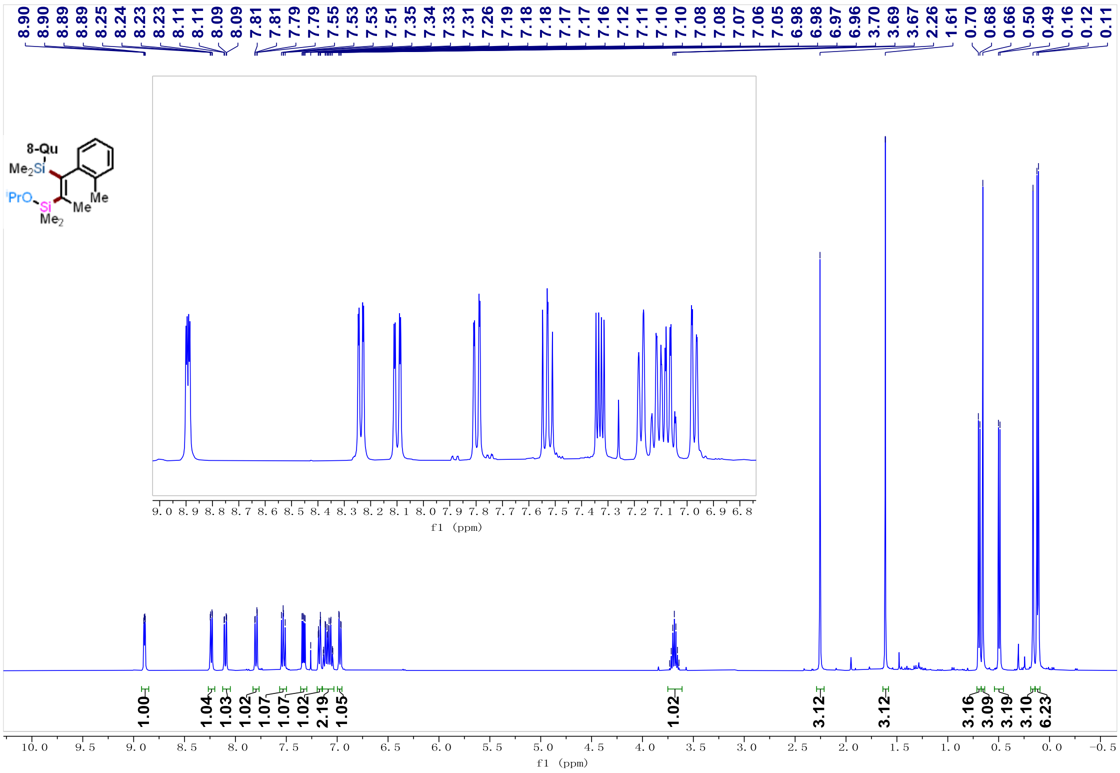

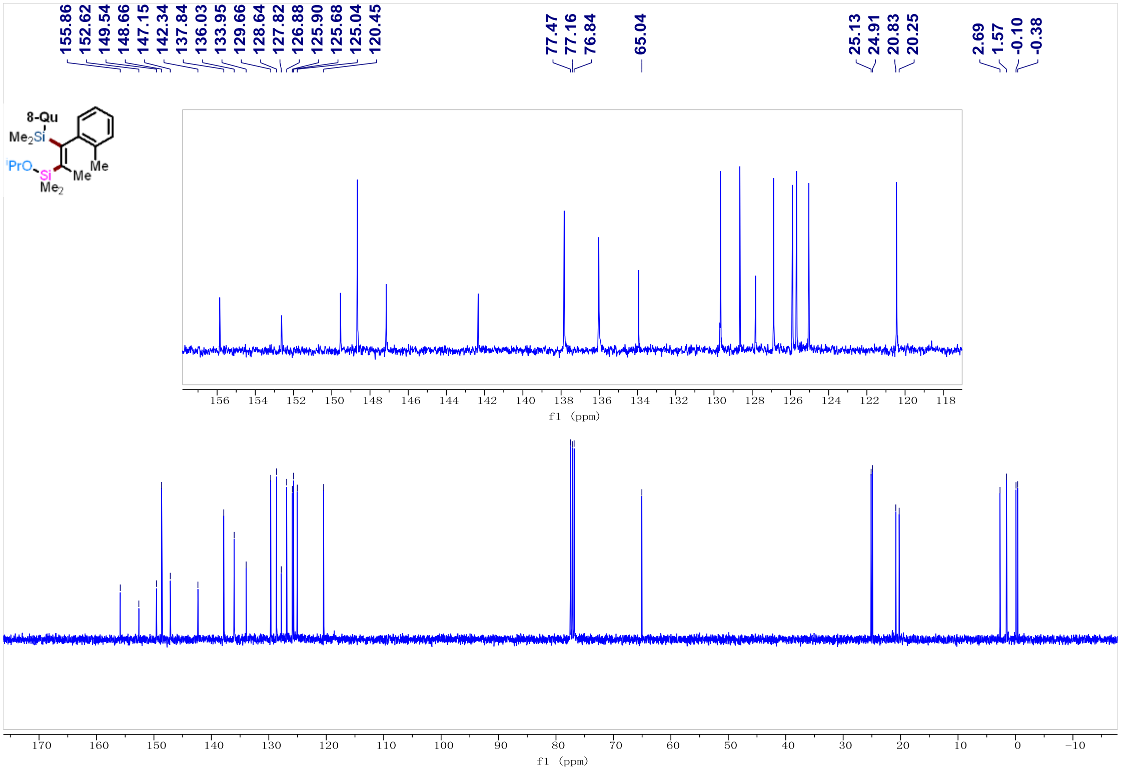

Supplementary Figure 39 ^{1}H and ^{13}C NMR Spectra for compound 3dx

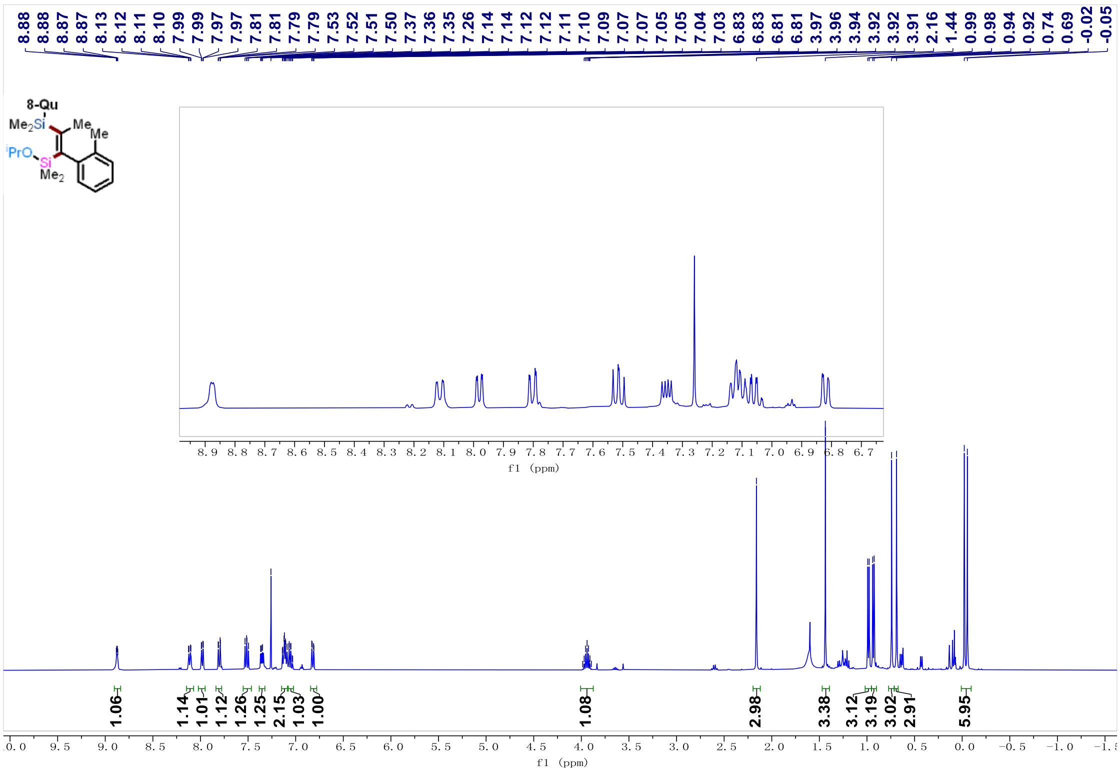

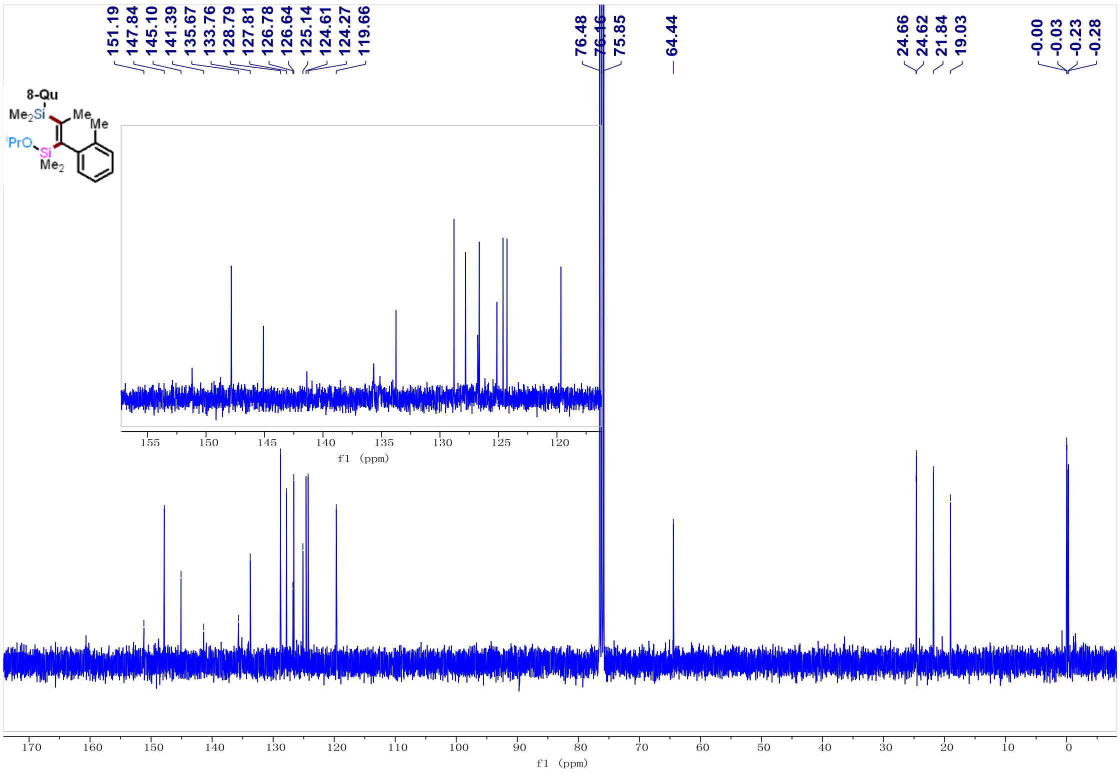

Supplementary Figure 40 ^{1}H and ^{13}C NMR Spectra for compound 3dx′

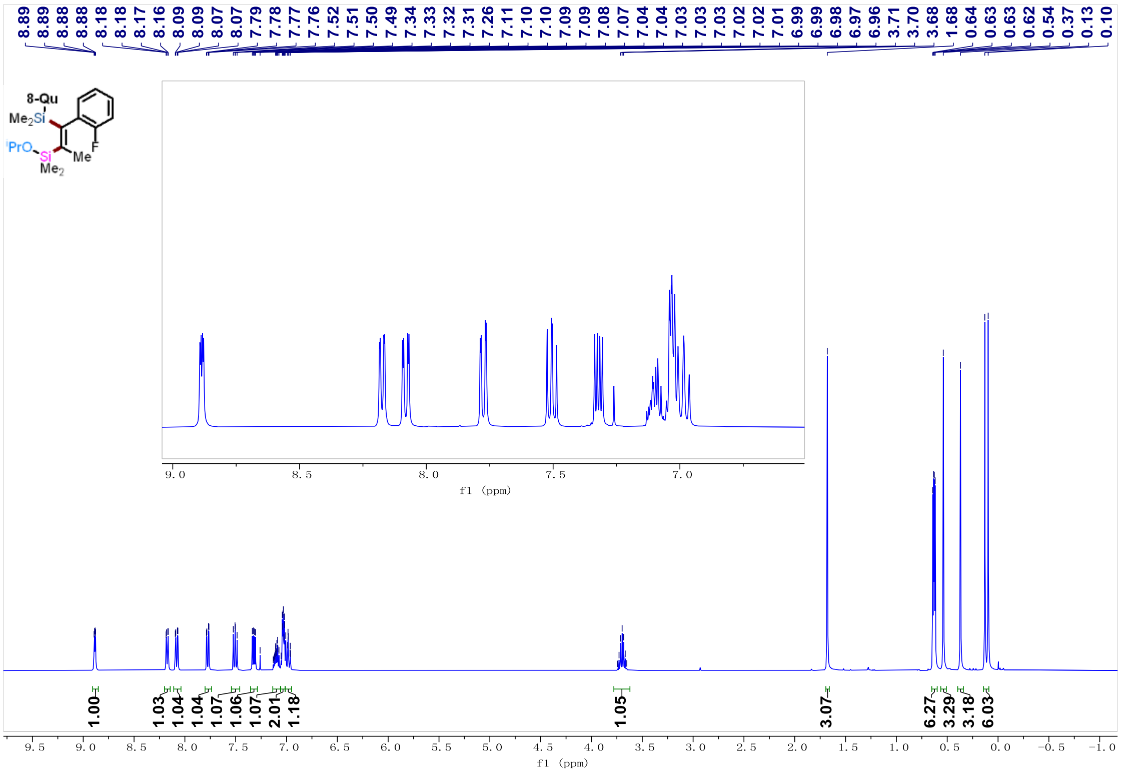
8-Qu
Me2Si
iPrO
Si
Me2
Me
F
f1 (ppm)

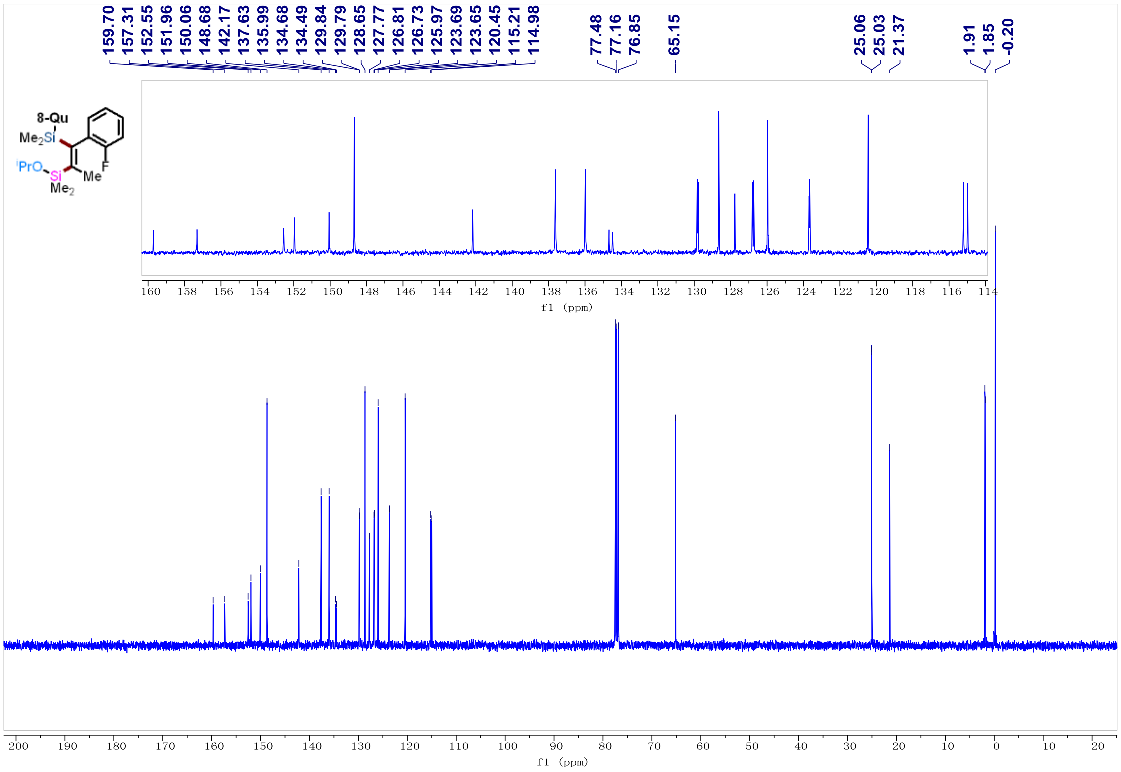
8-Qu
Me2Si
iPrO
Si
Me2
Me
F
f1 (ppm)

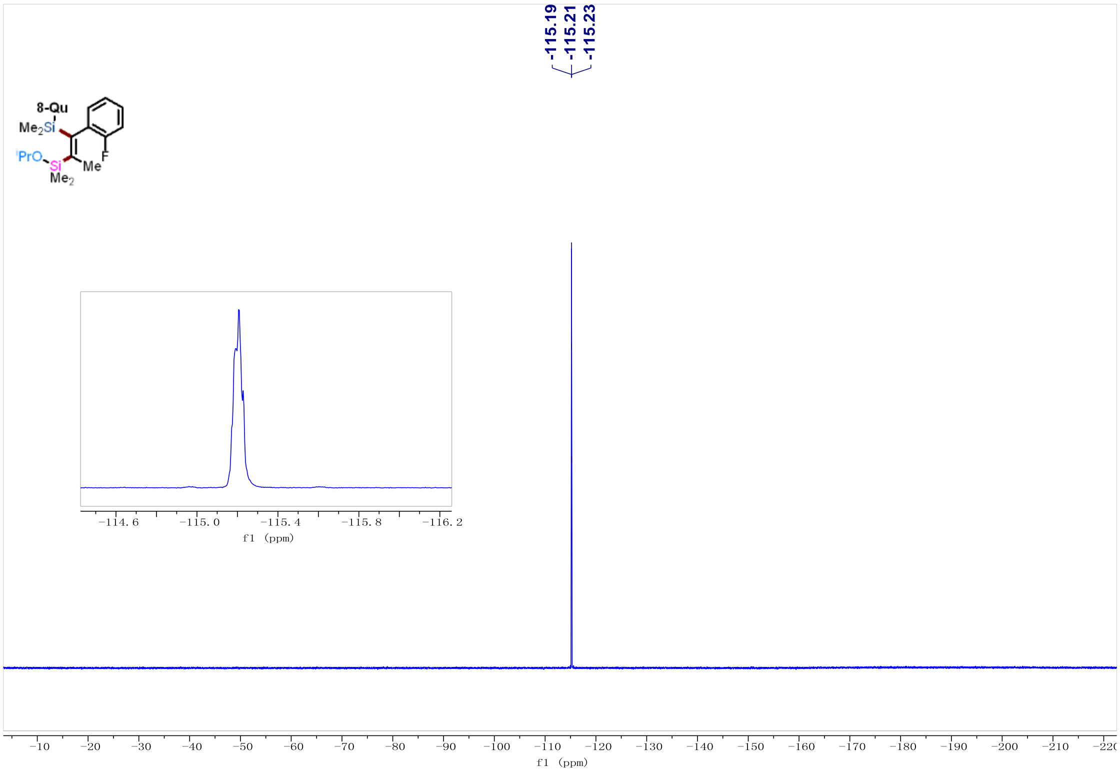

Supplementary Figure 41 ^{1}H, ^{13}C and ^{19}F NMR Spectra for compound 3dy

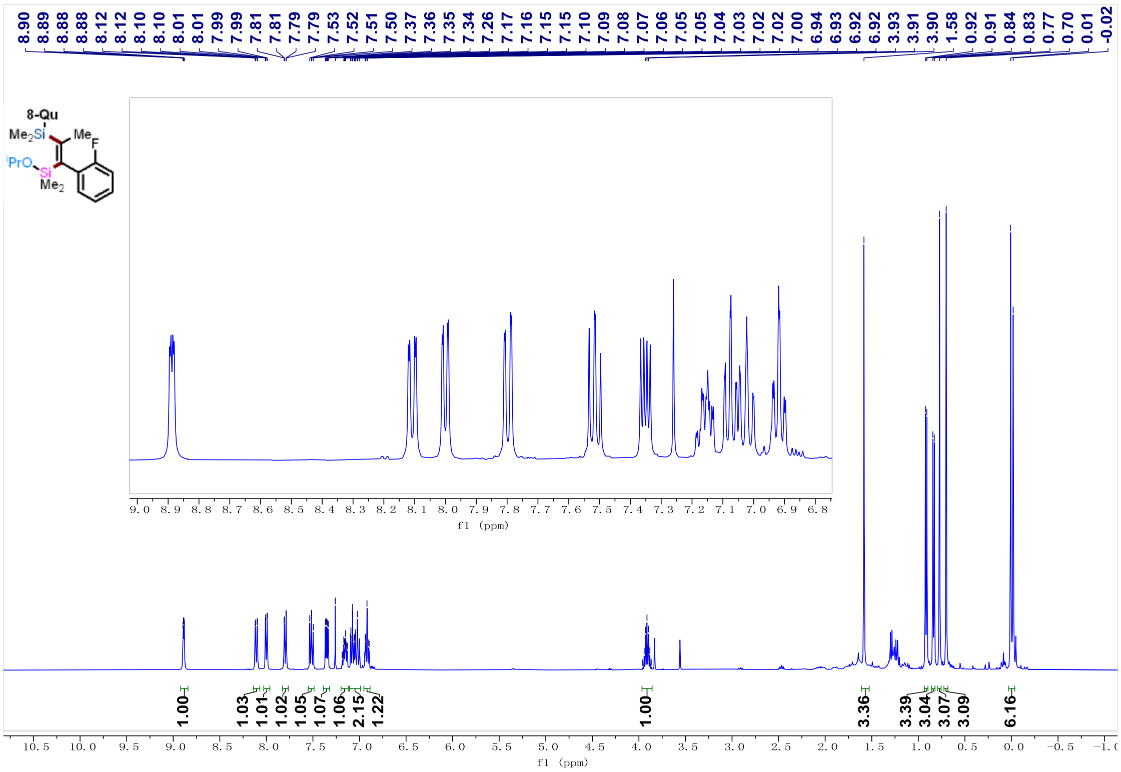

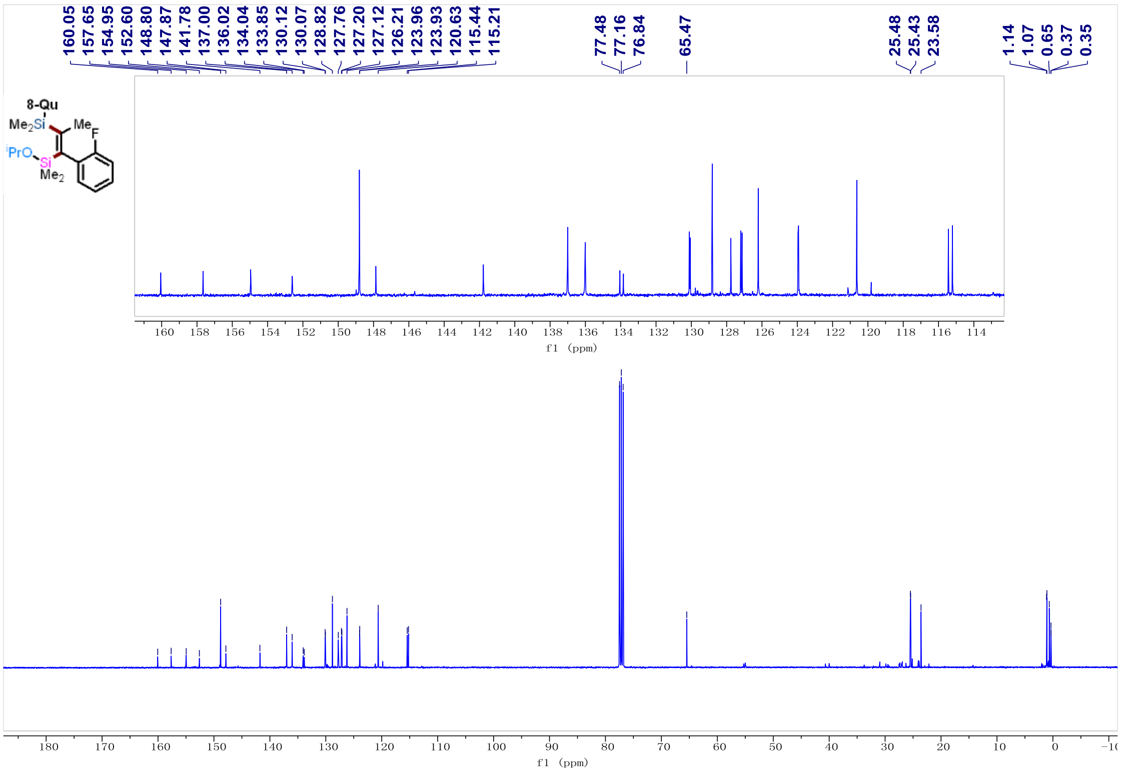

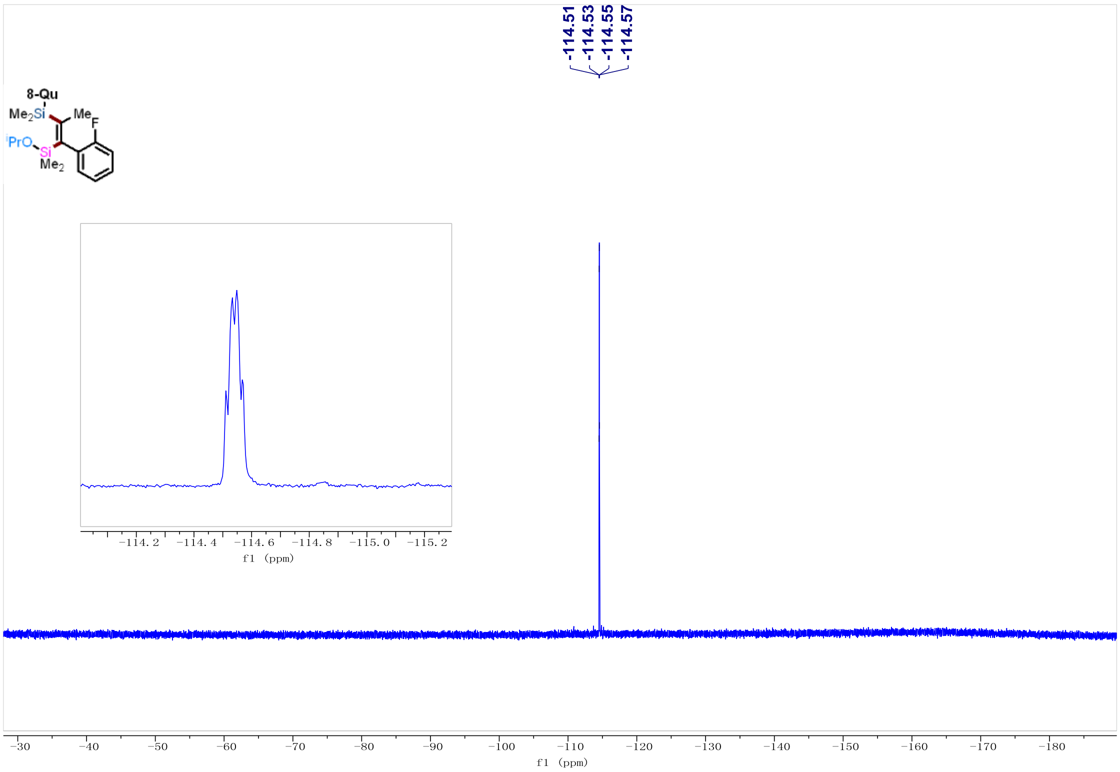

Supplementary Figure 42 ^{1}H, ^{13}C and ^{19}F NMR Spectra for compound 3dy′

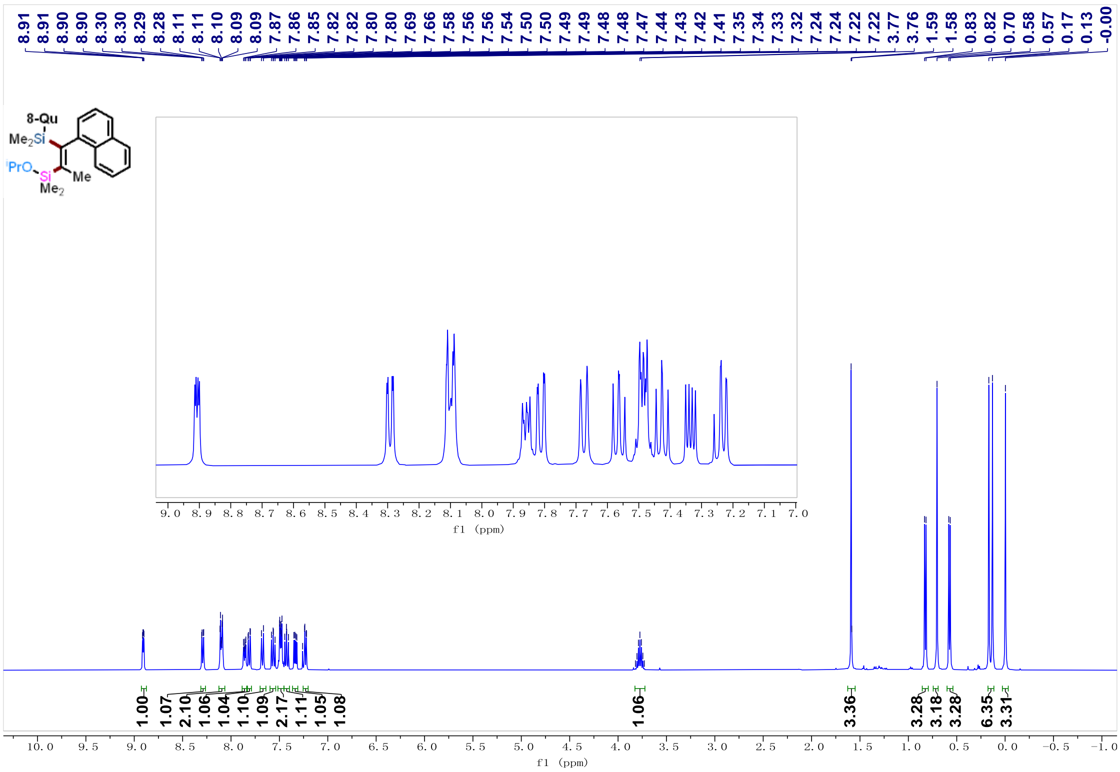

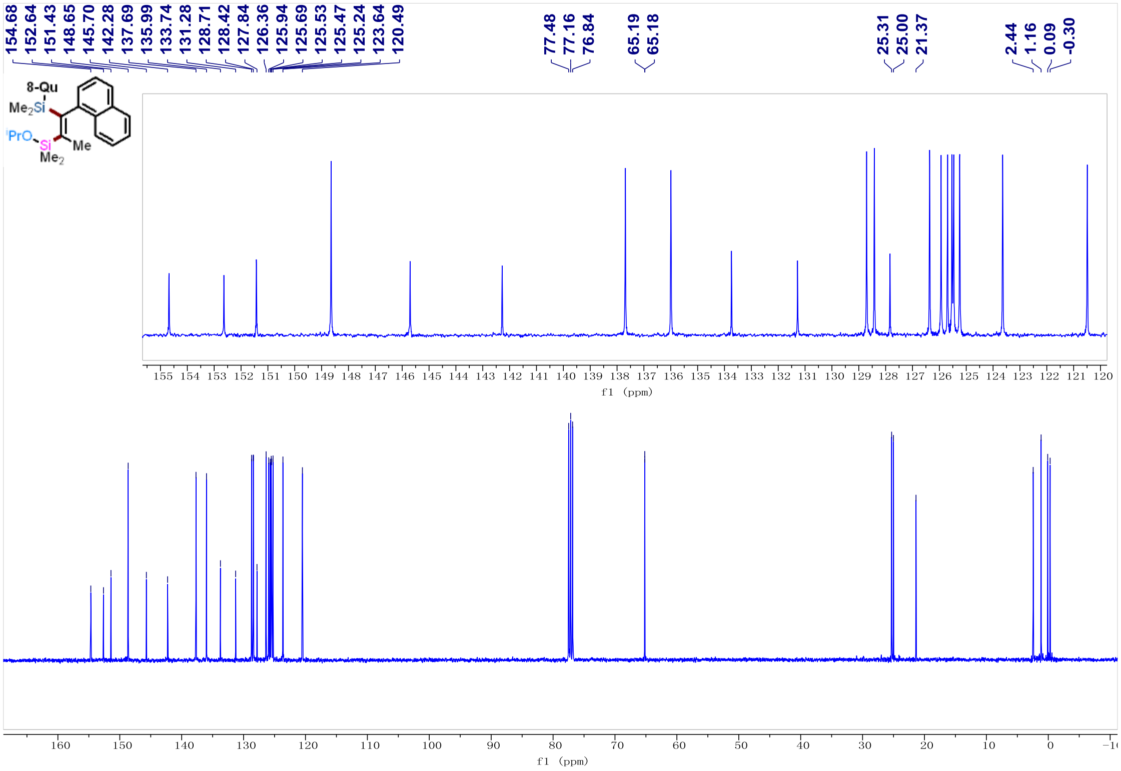

Supplementary Figure 43 ^{1}H and ^{13}C NMR Spectra for compound 3dz

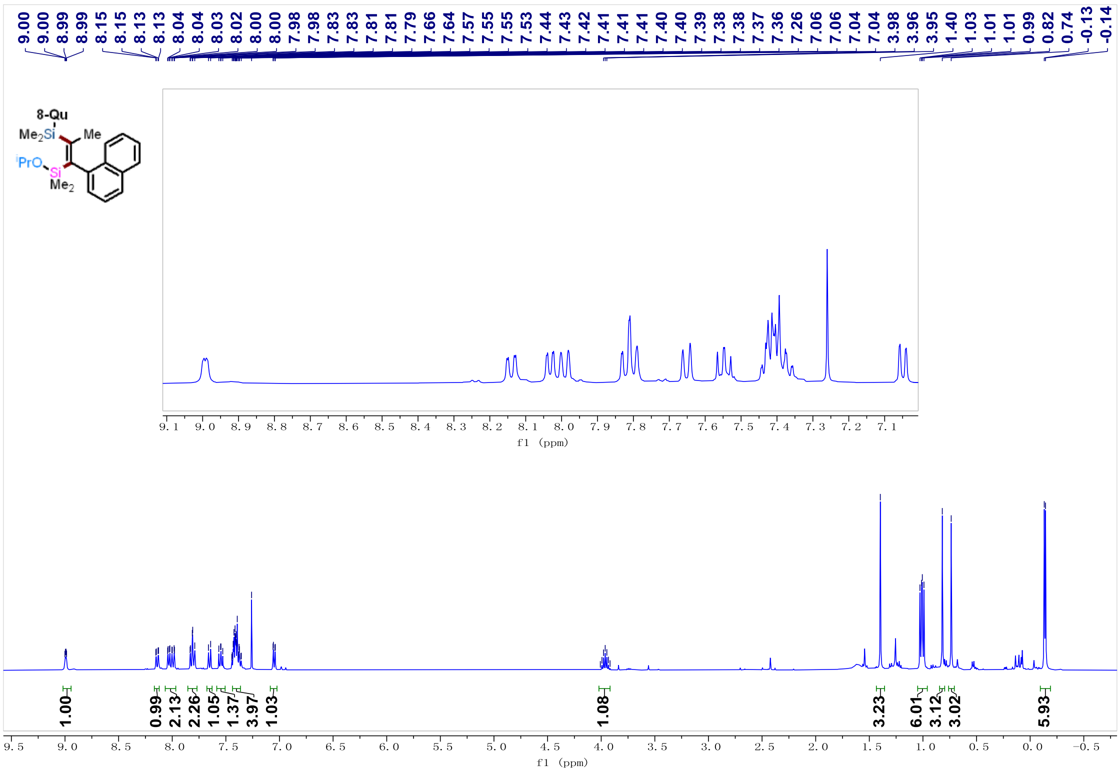

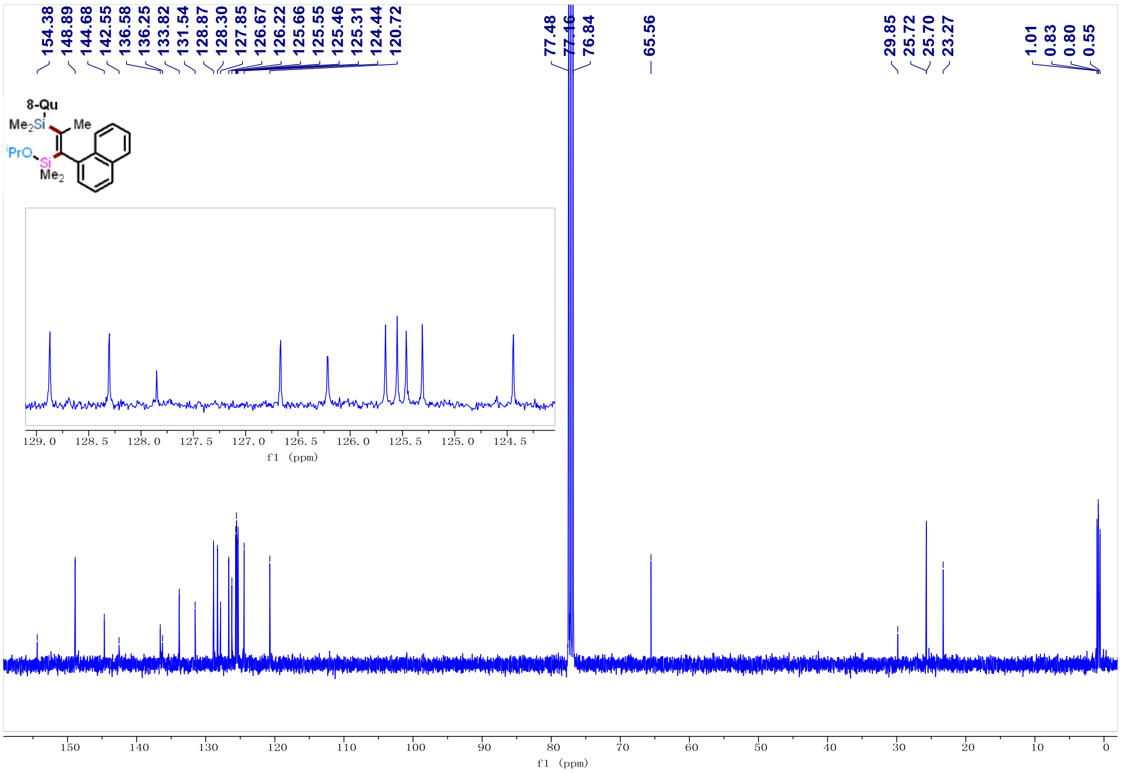

Supplementary Figure 44 ^{1}H and ^{13}C NMR Spectra for compound 3dz′

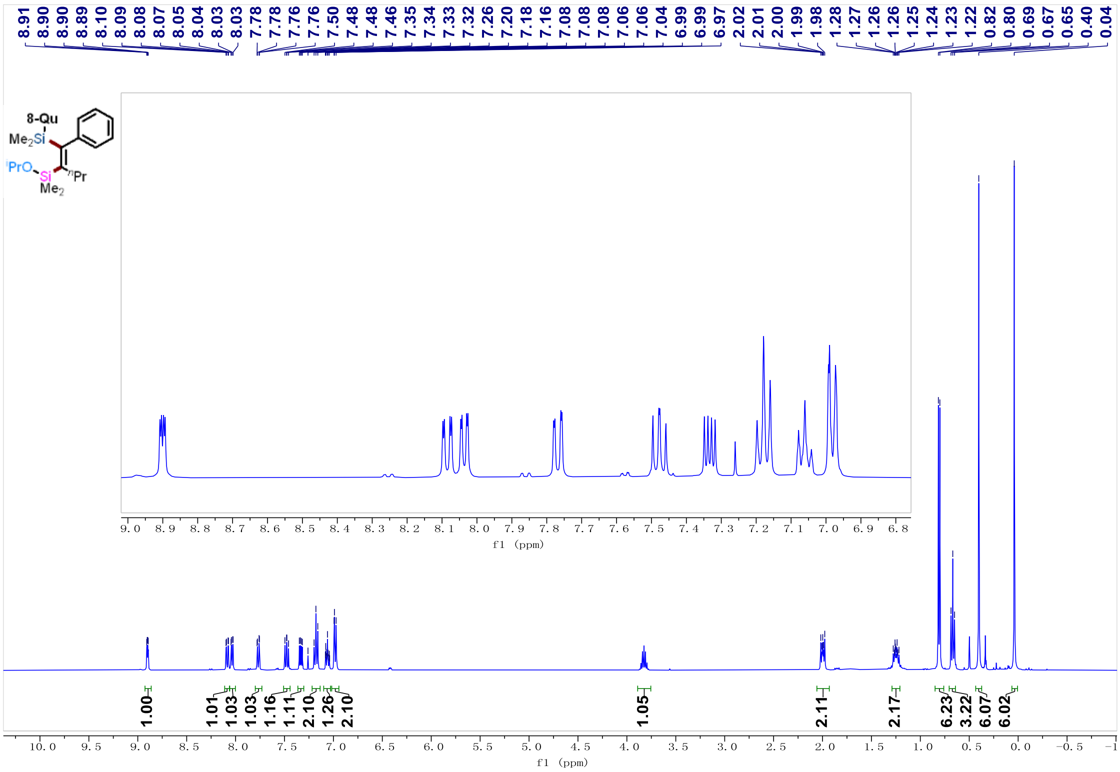

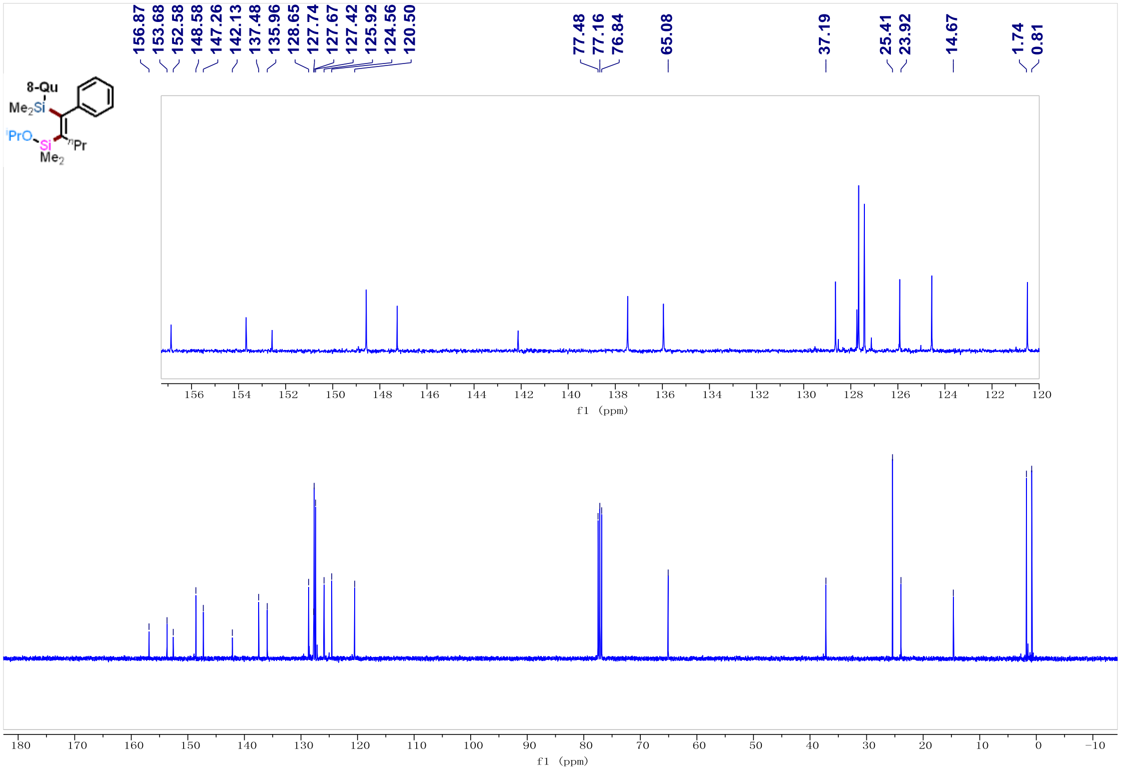

Supplementary Figure 45 ^{1}H and ^{13}C NMR Spectra for compound 3dä

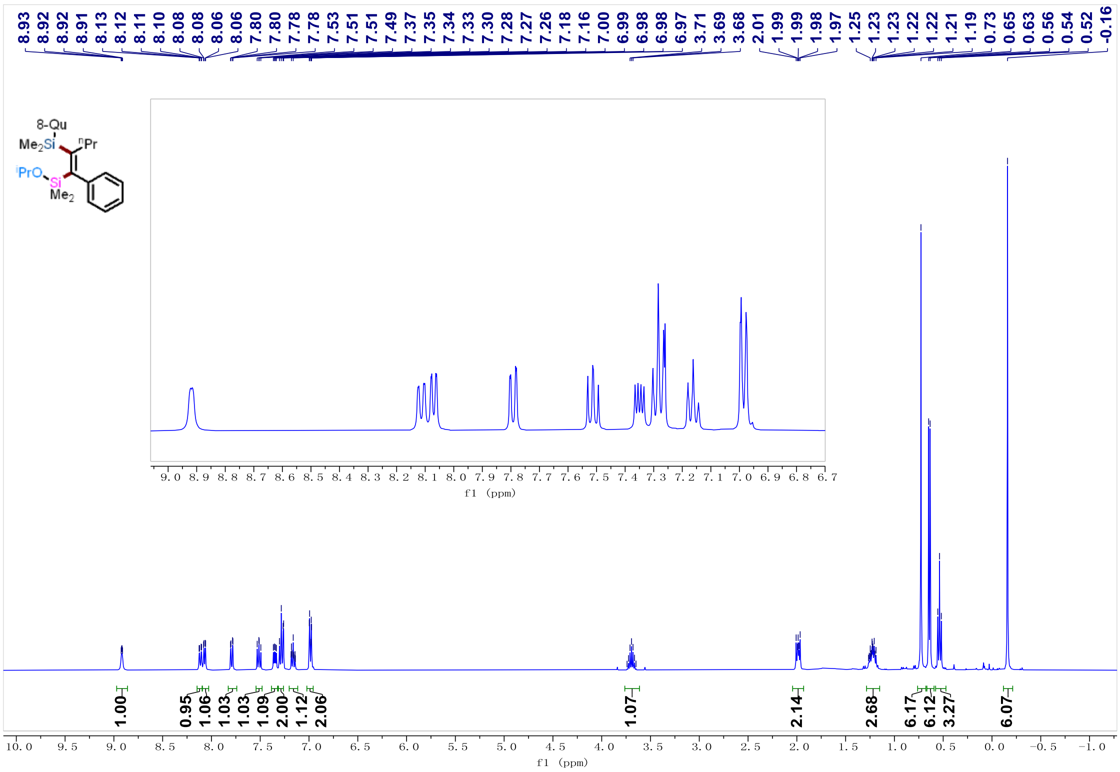

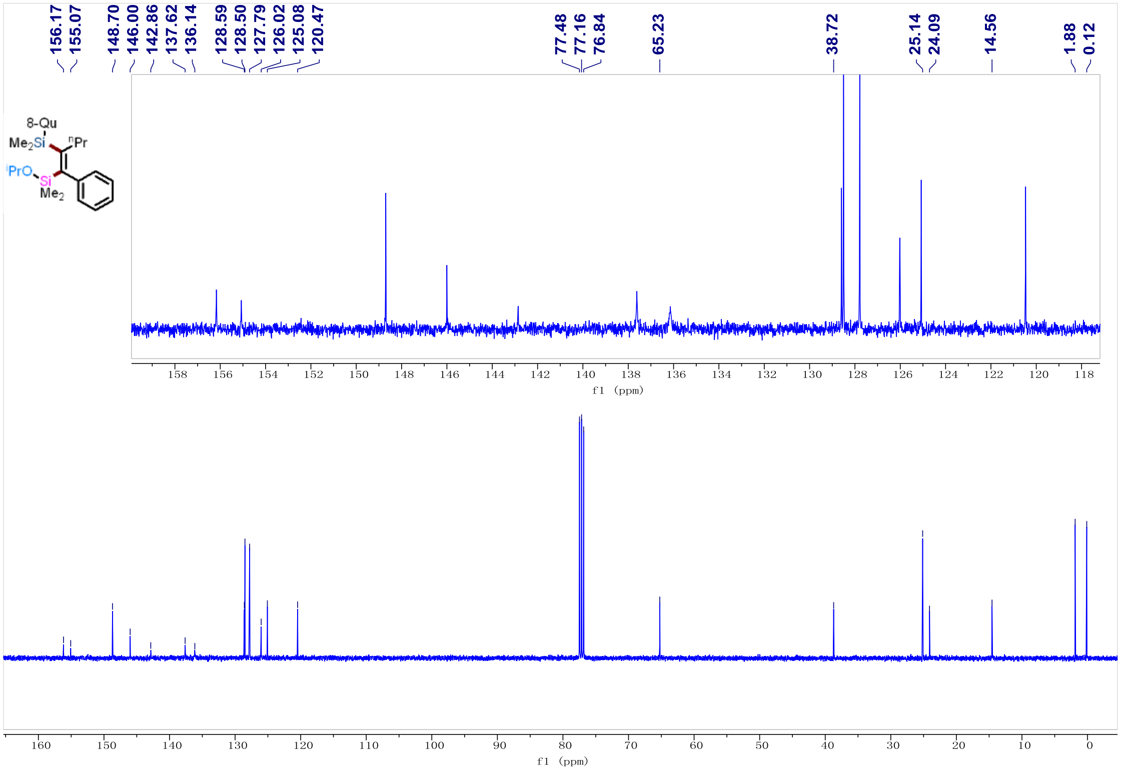

Supplementary Figure 46 ^{1}H and ^{13}C NMR Spectra for compound 3dä′

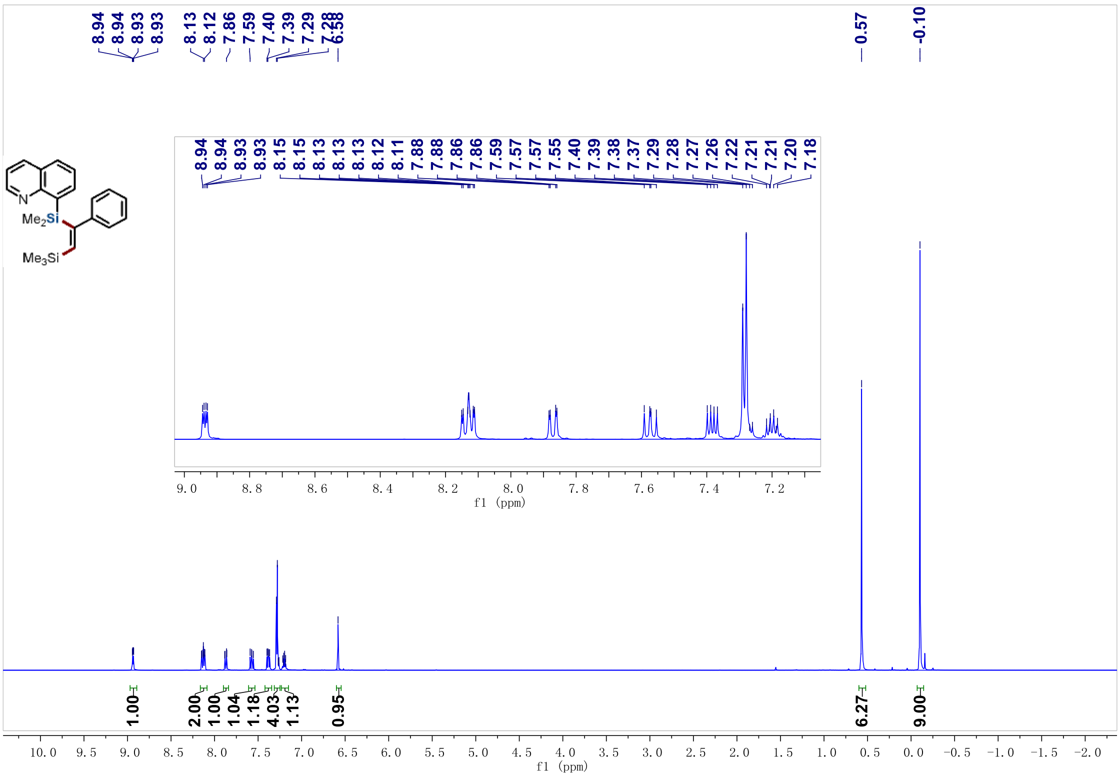

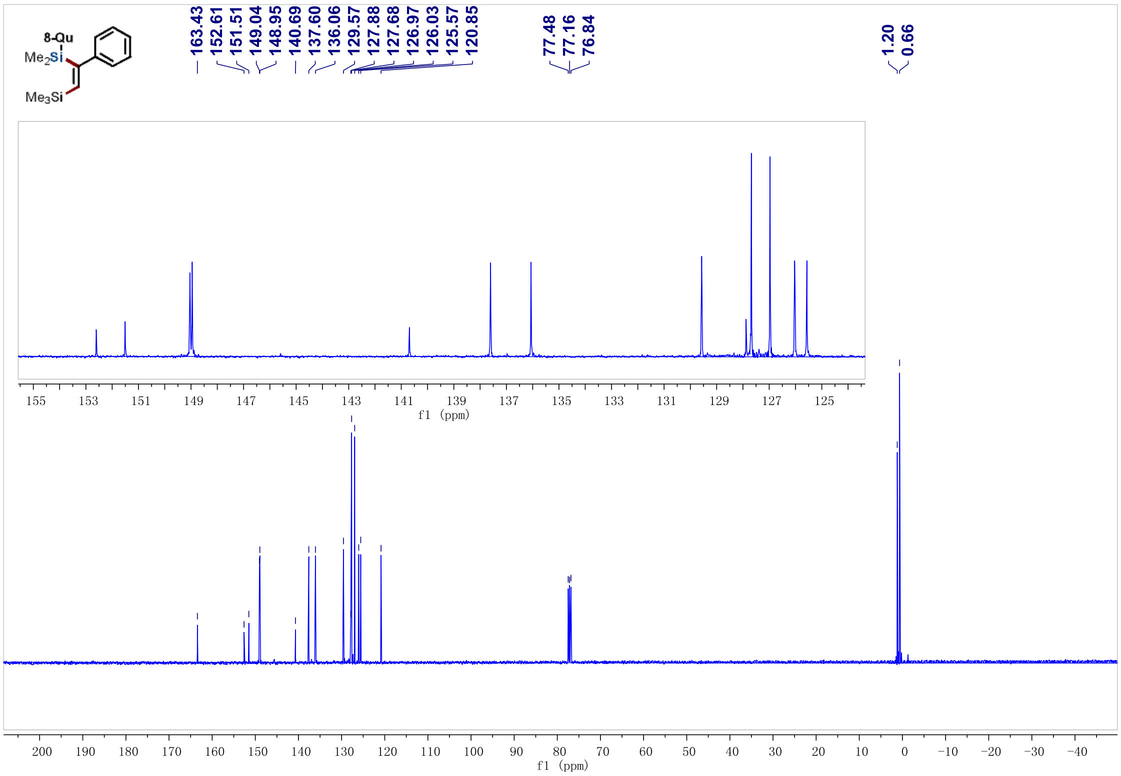

Supplementary Figure 47 ^{1}H and ^{13}C NMR Spectra for compound 4aa

COSY (**4aa**)

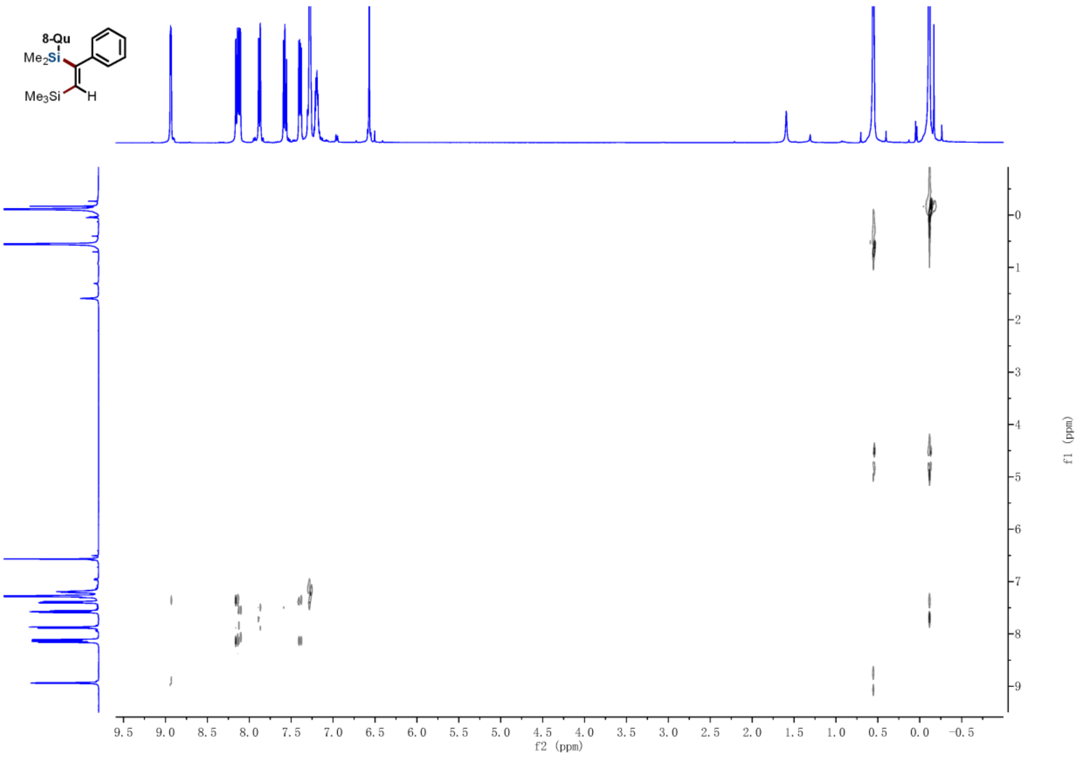

HSQC (**4aa**)

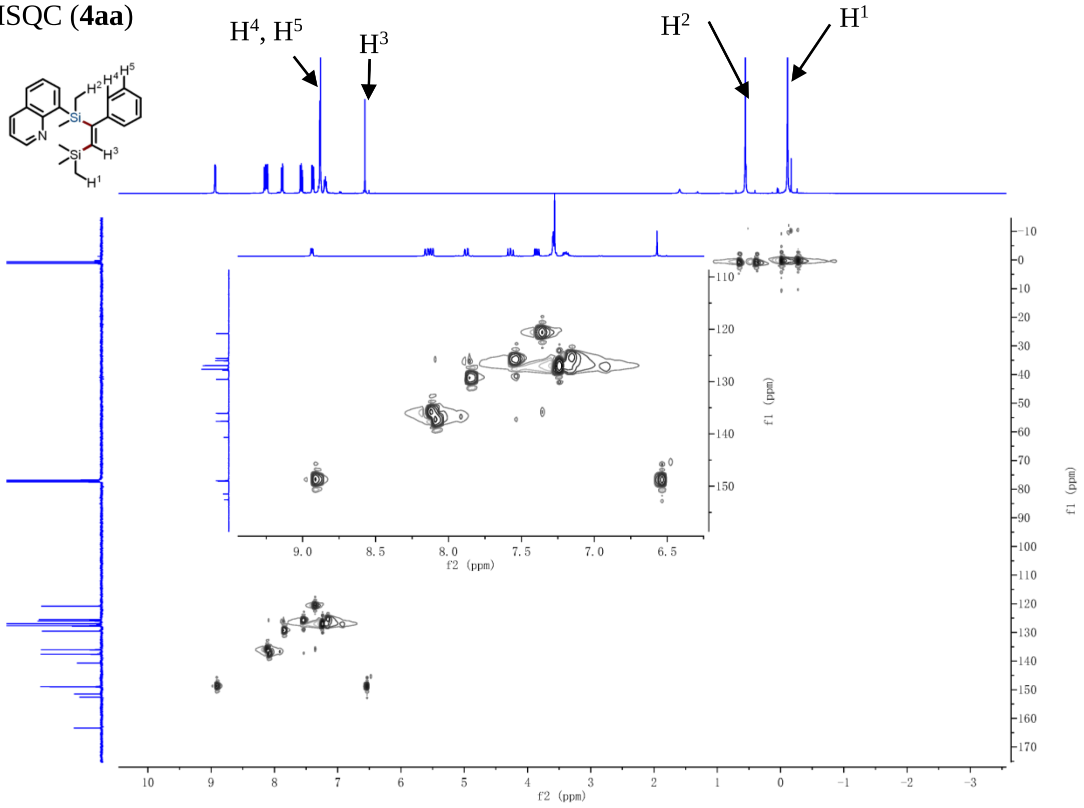

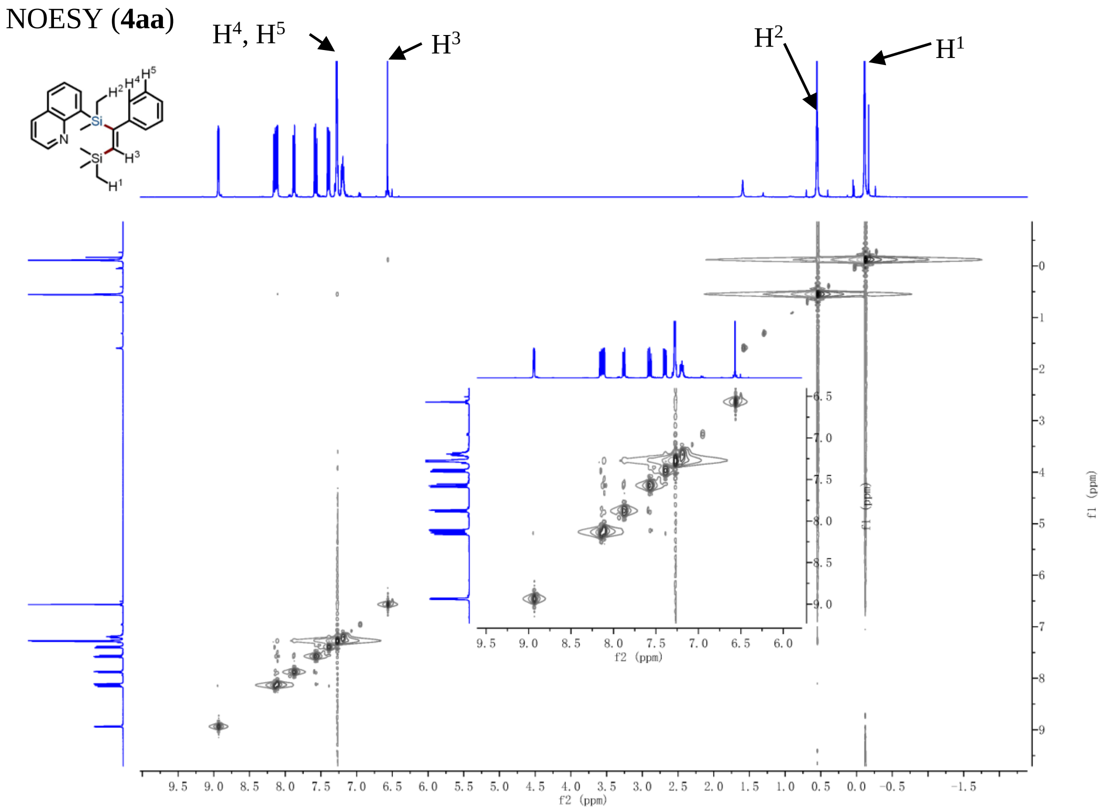

Supplementary Figure 48 COSY, HSQC and NOESY Spectra for compound 4aa

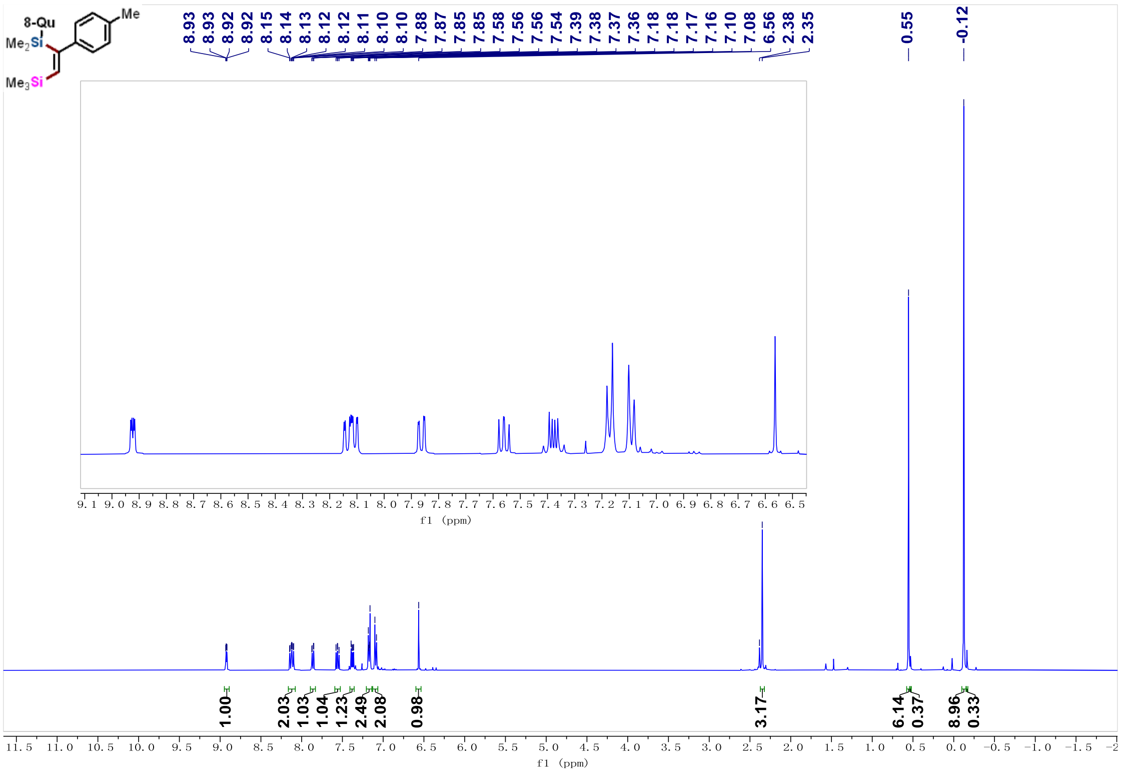

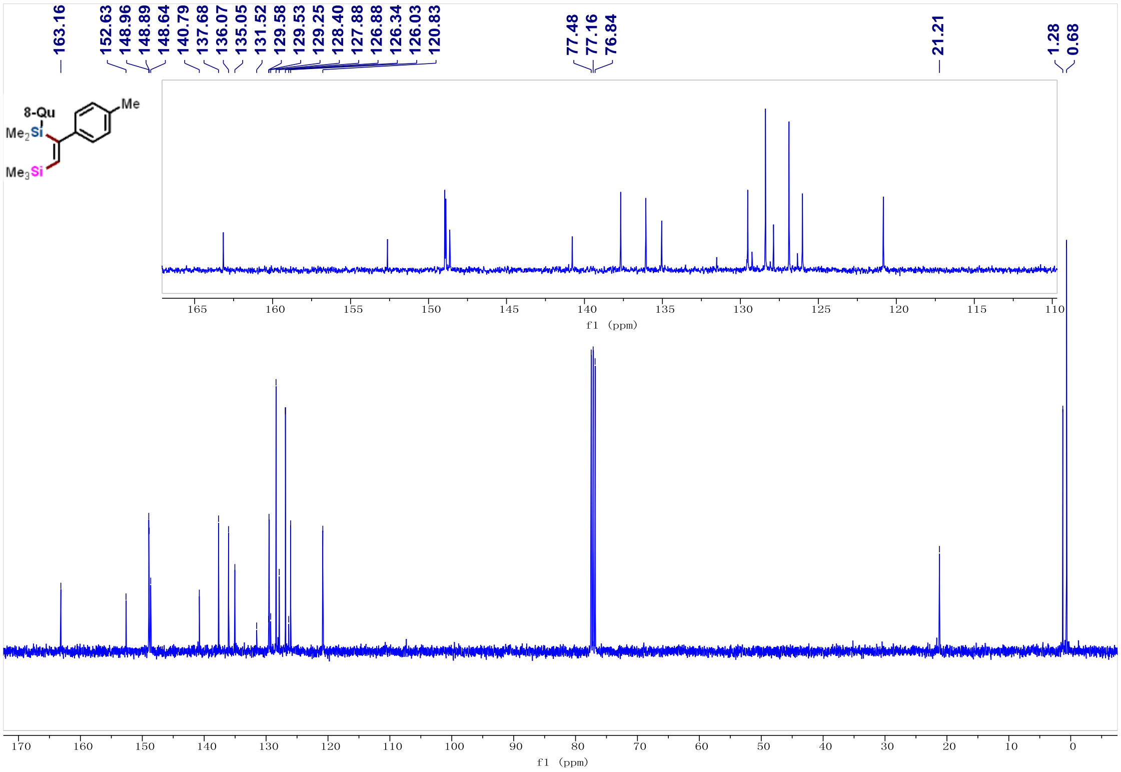

Supplementary Figure 49 ^{1}H and ^{13}C NMR Spectra for compound 4ab

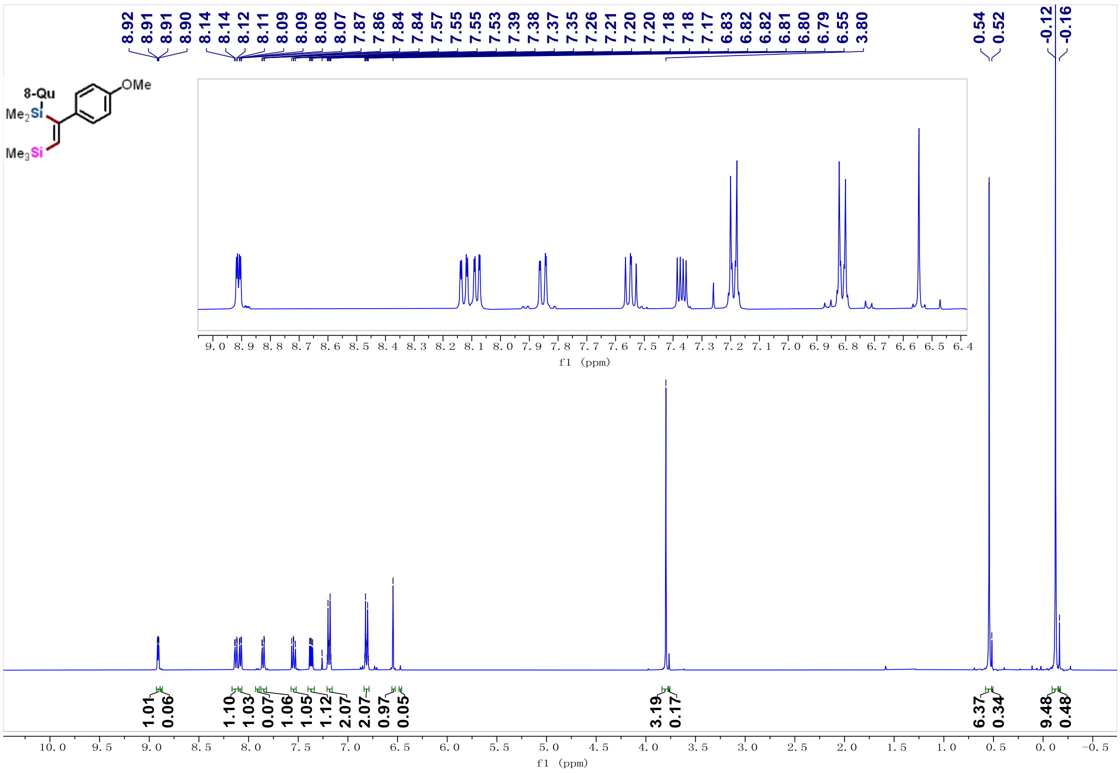

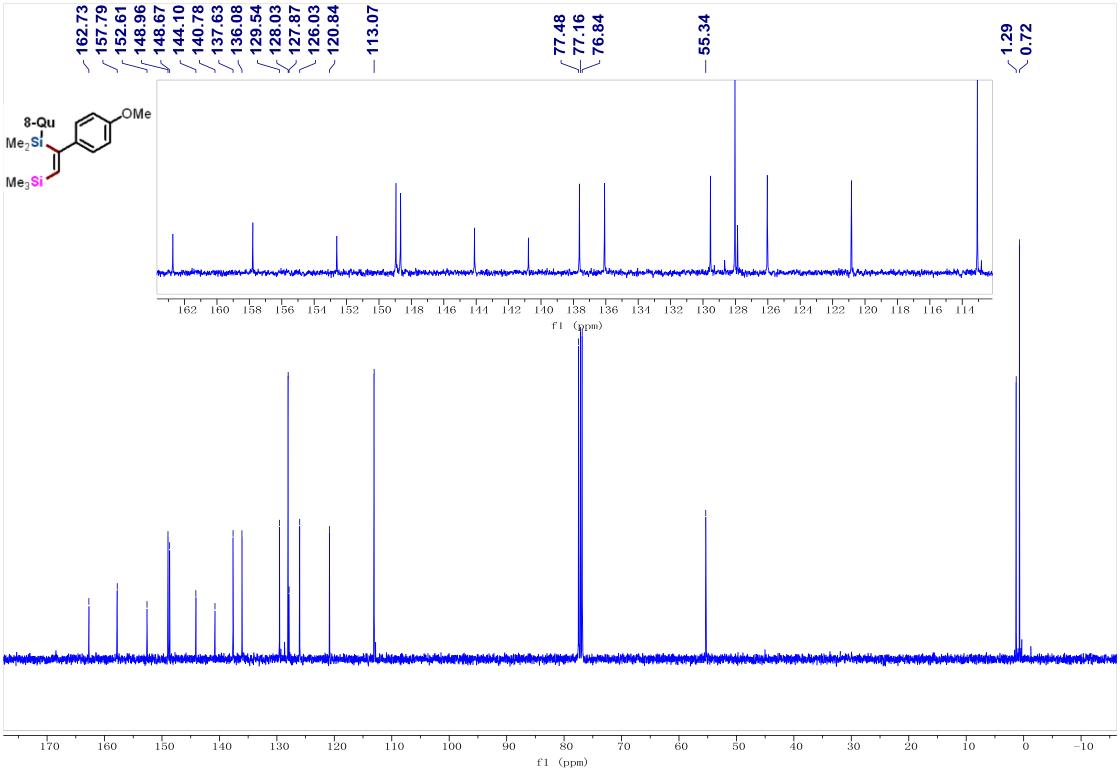

Supplementary Figure 50 ^{1}H and ^{13}C NMR Spectra for compound 4ac

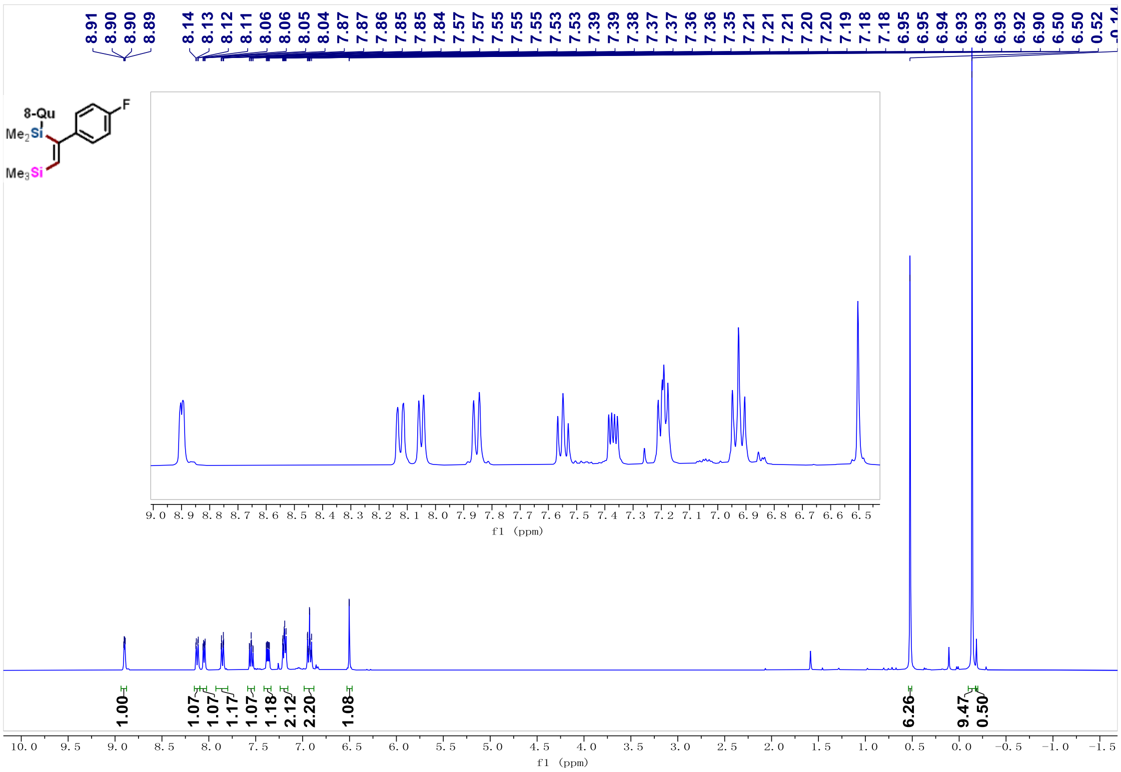

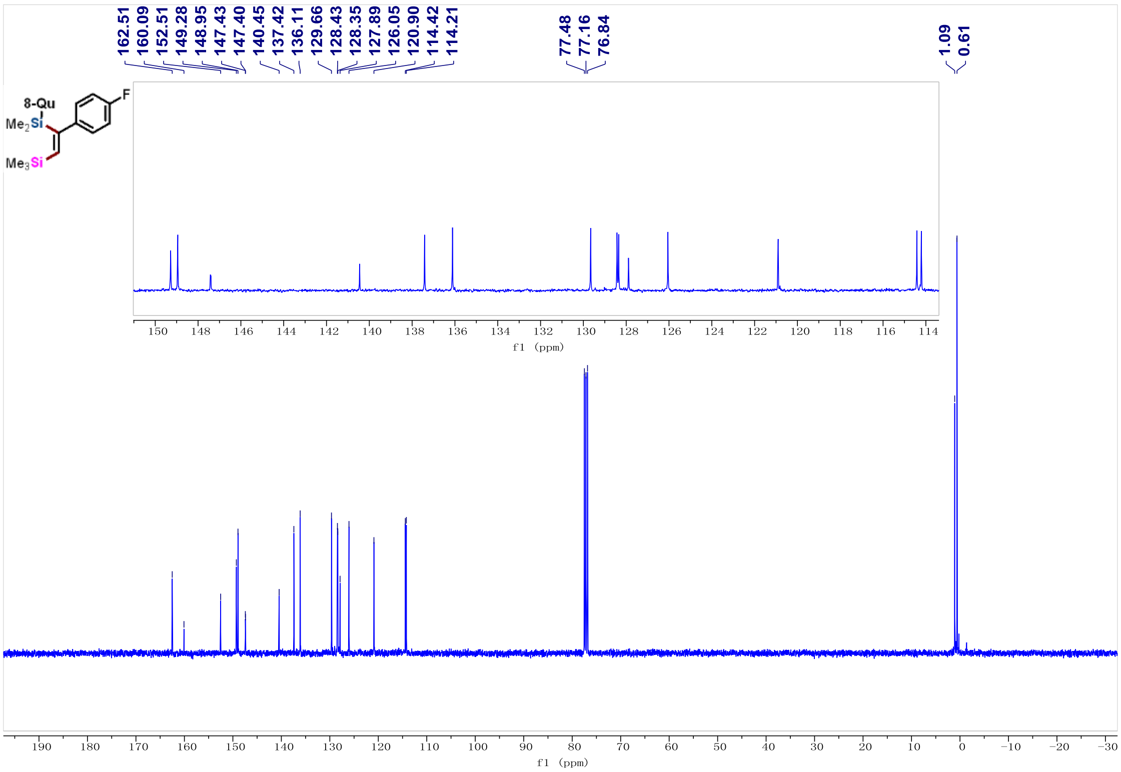

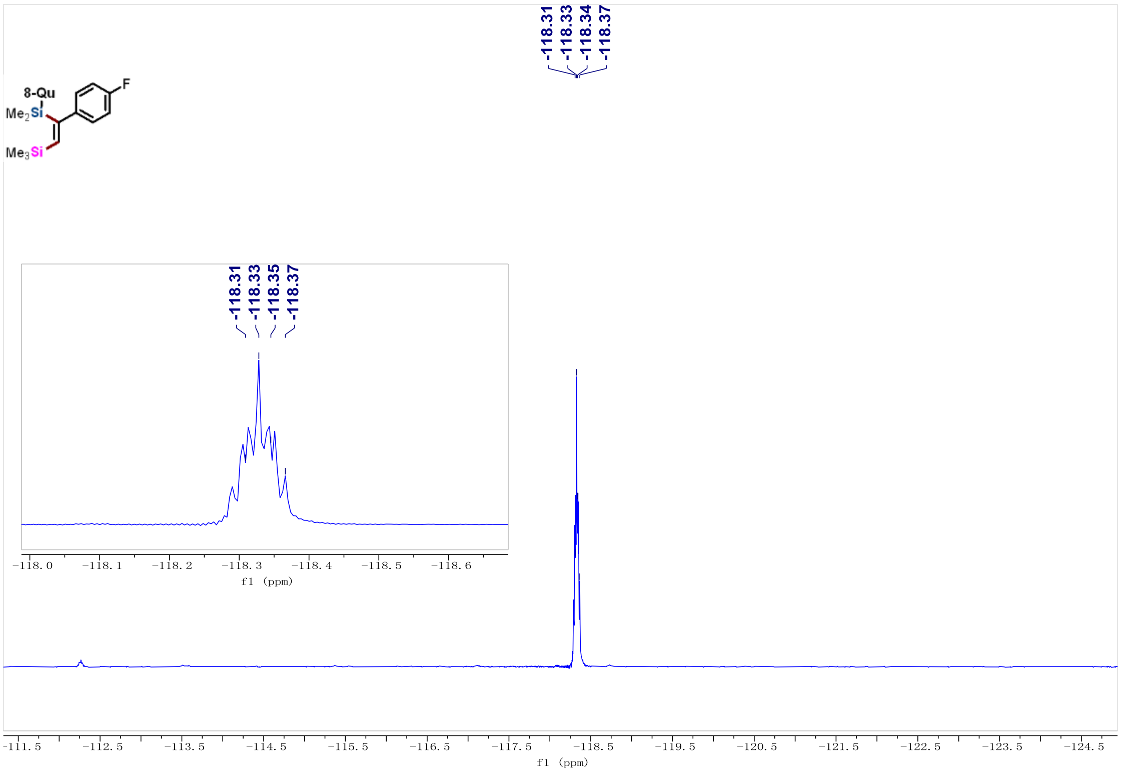

Supplementary Figure 51 ^{1}H, ^{13}C and ^{19}F NMR Spectra for compound 4ad

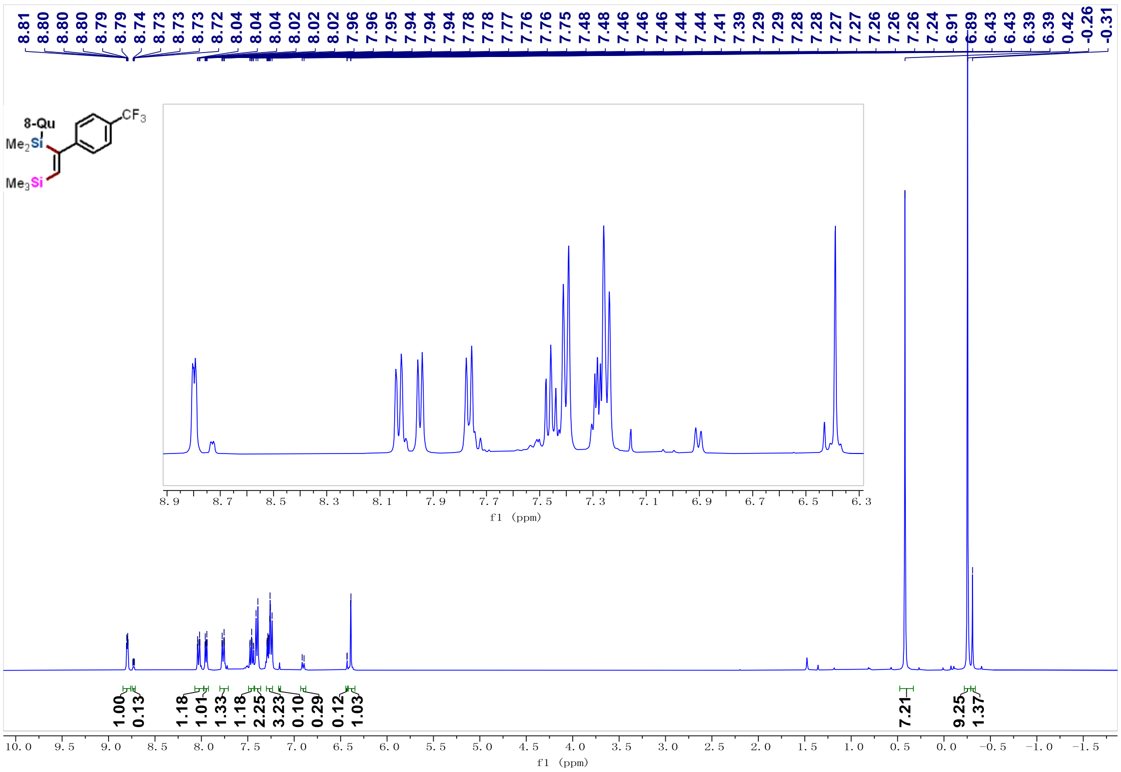

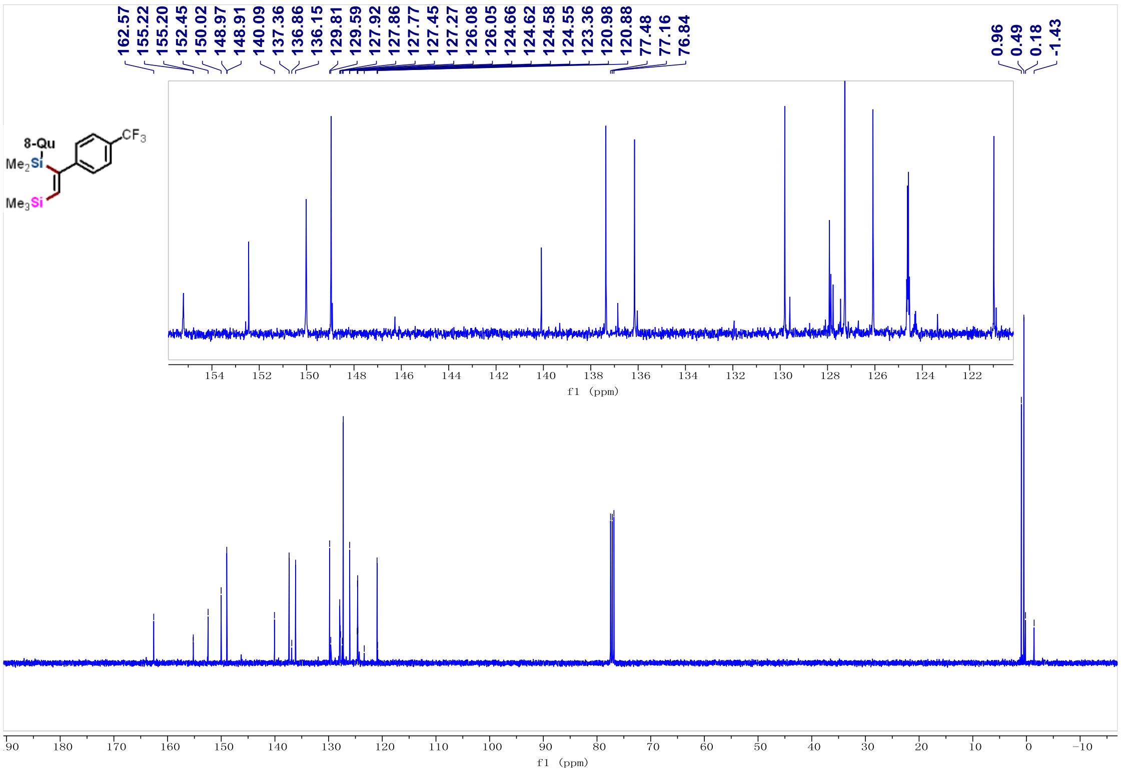

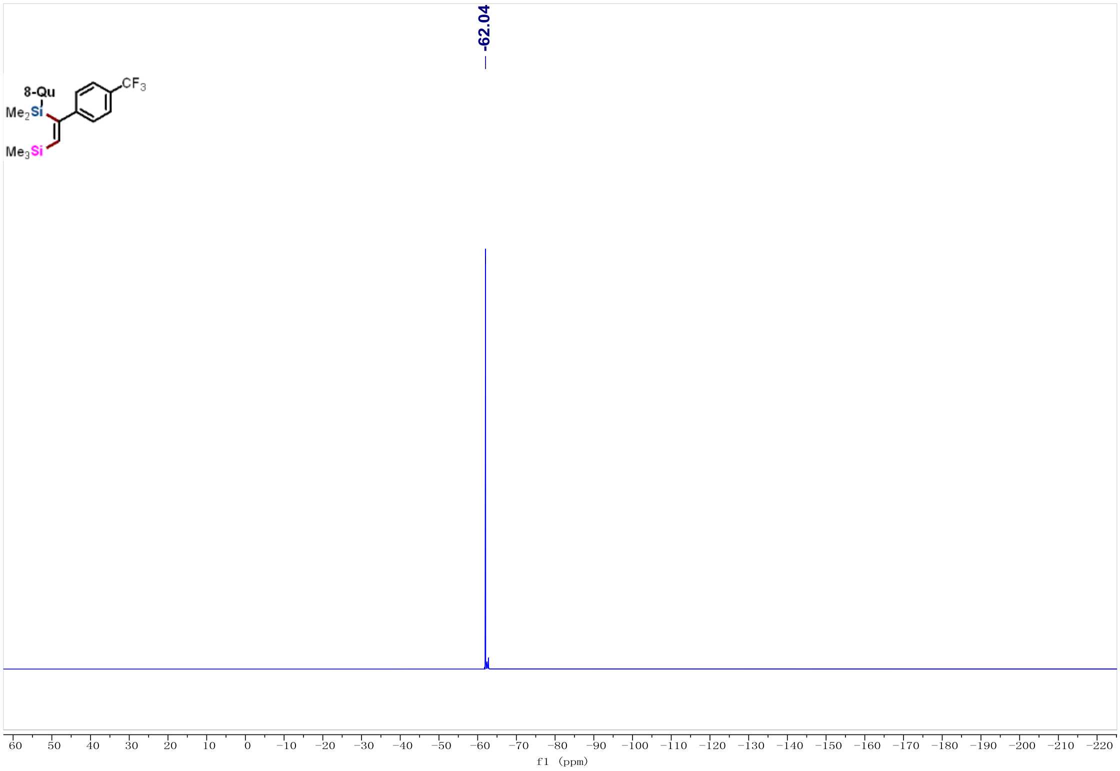

Supplementary Figure 52 ^{1}H, ^{13}C and ^{19}F NMR Spectra for compound 4ae

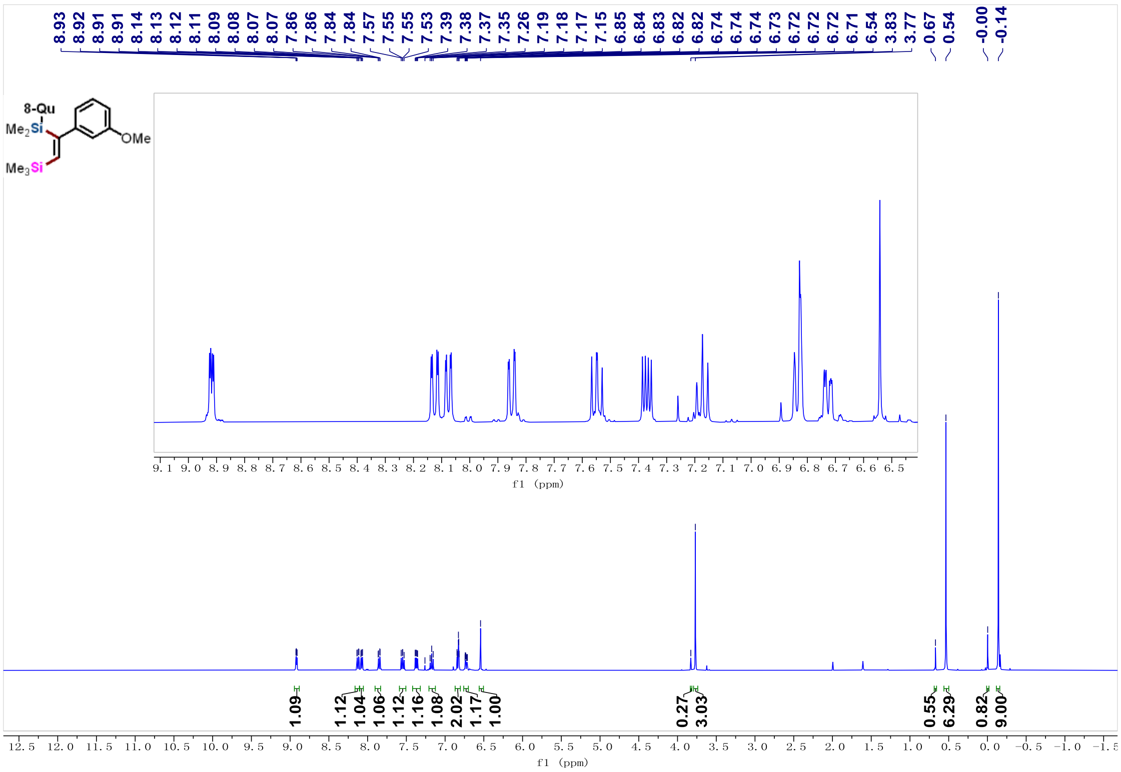

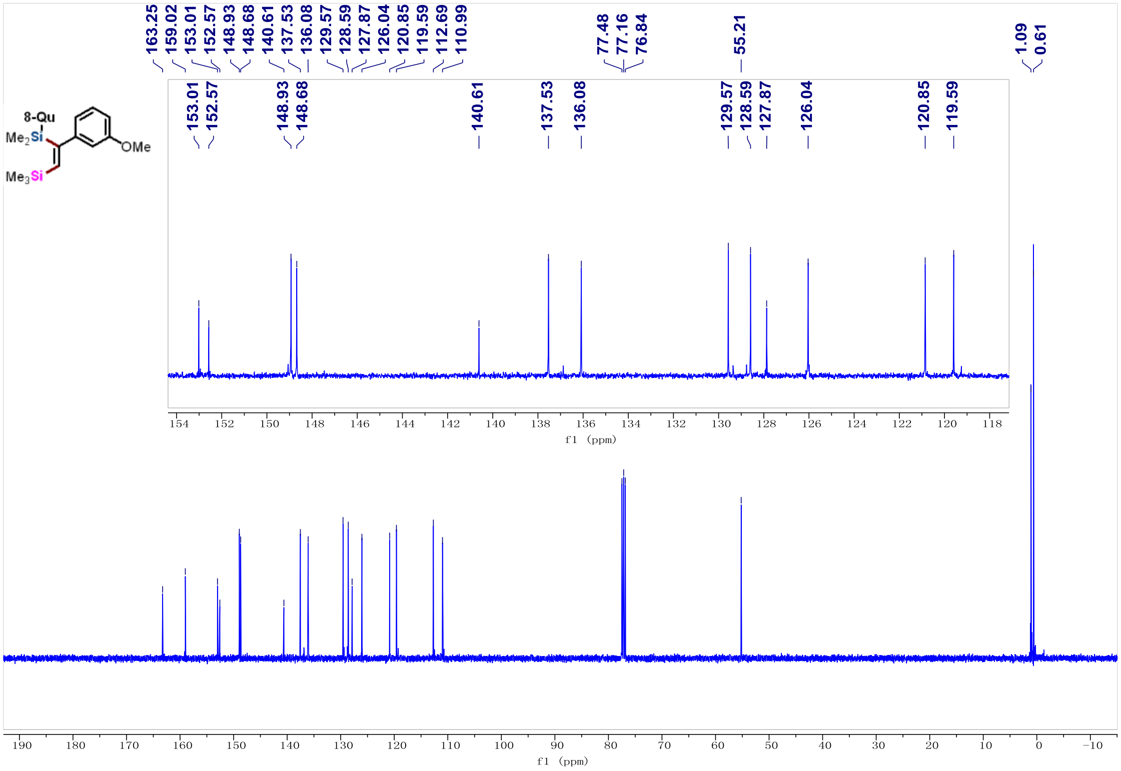

Supplementary Figure 53 ^{1}H and ^{13}C NMR Spectra for compound 4af

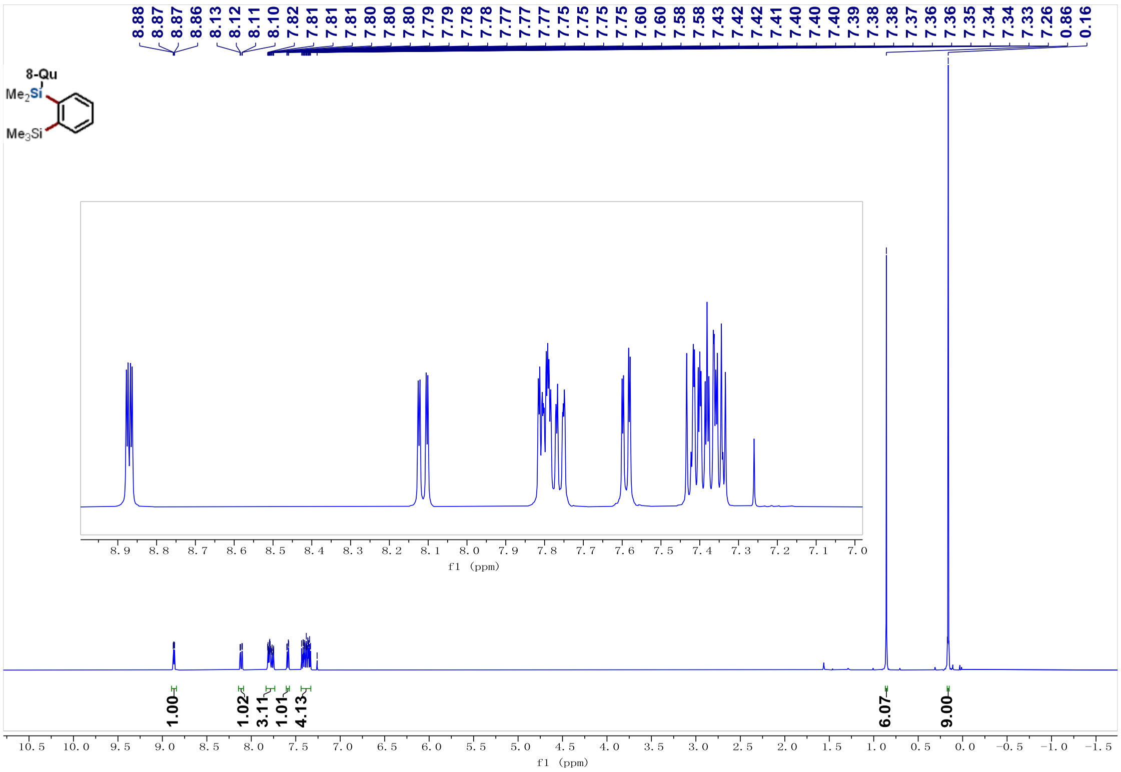

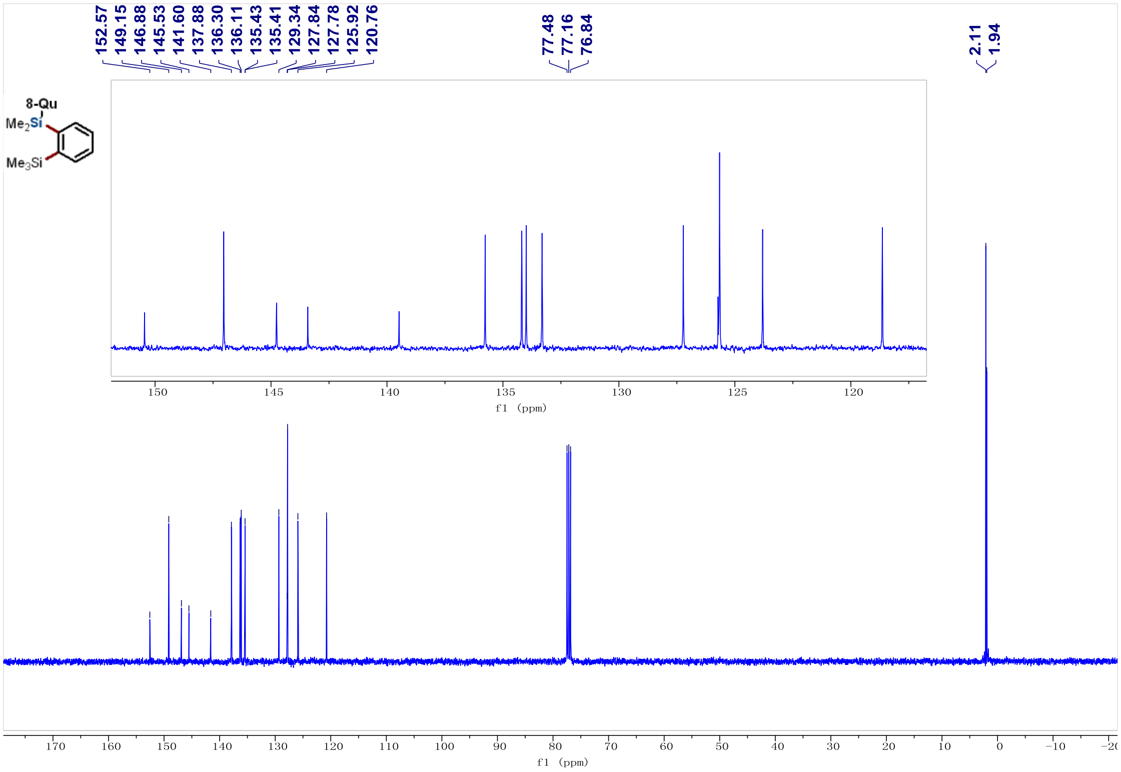

Supplementary Figure 54 ^{1}H and ^{13}C NMR Spectra for compound 6aa

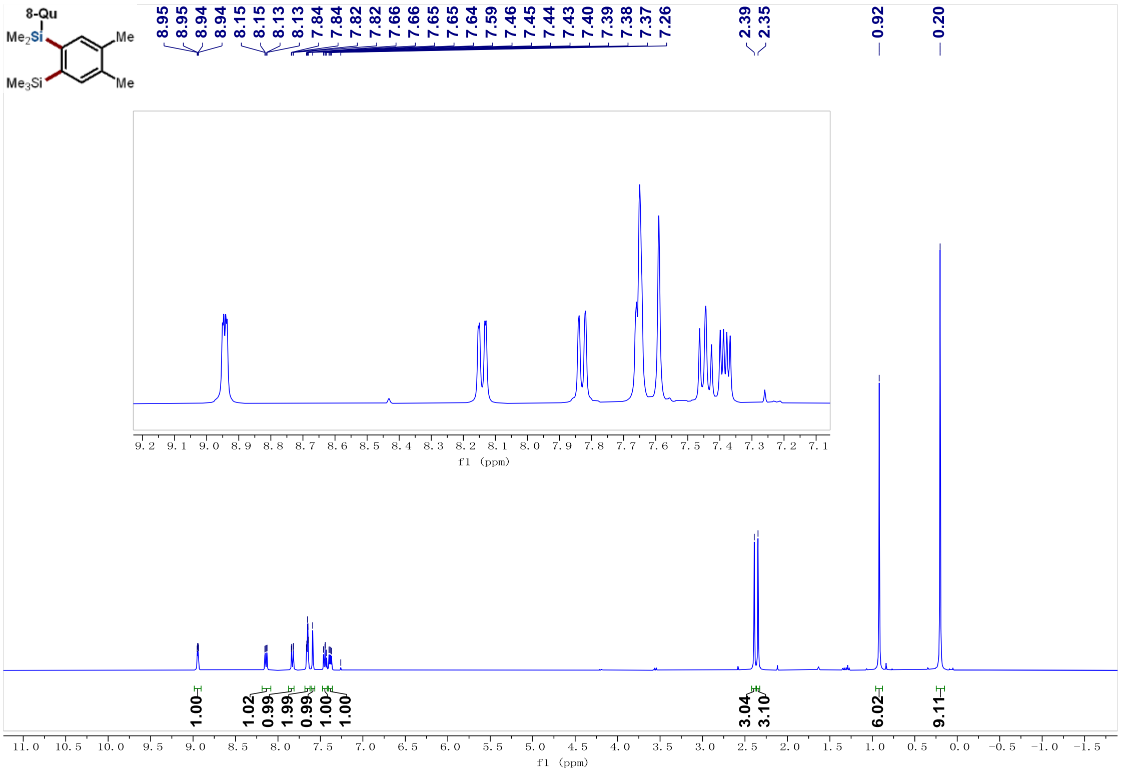

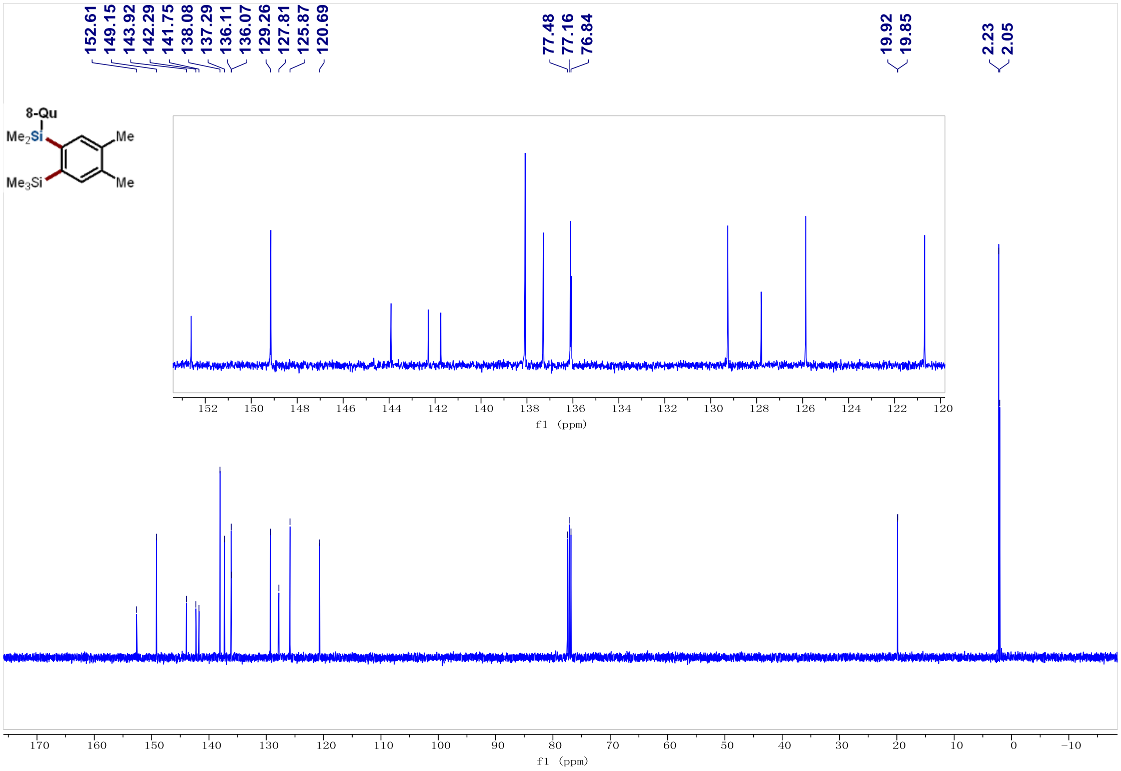

Supplementary Figure 55 ^{1}H and ^{13}C NMR Spectra for compound 6ab

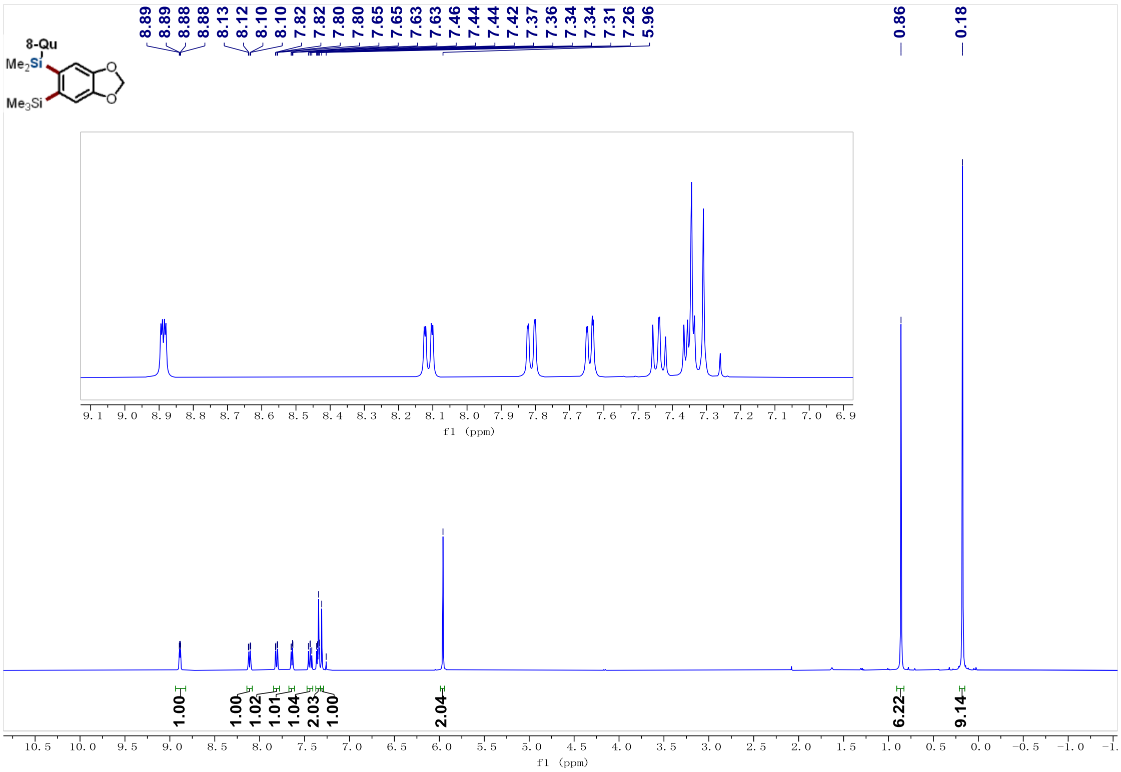

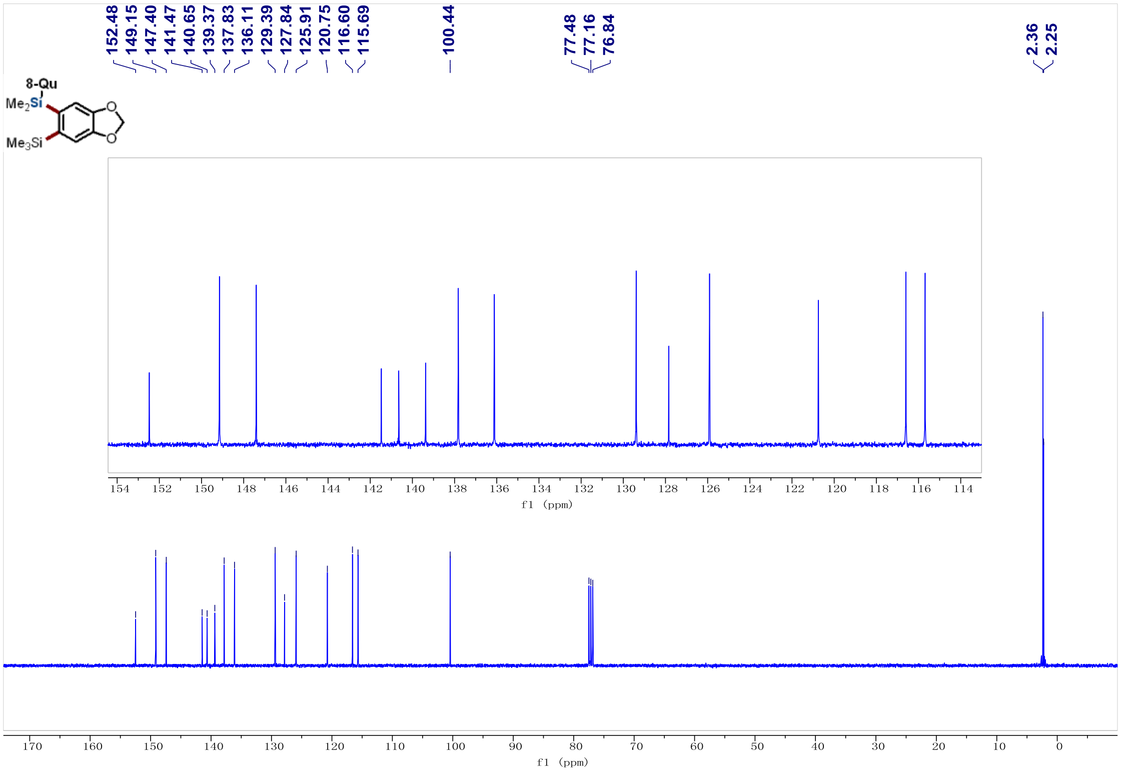

Supplementary Figure 56 ^{1}H and ^{13}C NMR Spectra for compound 6ac

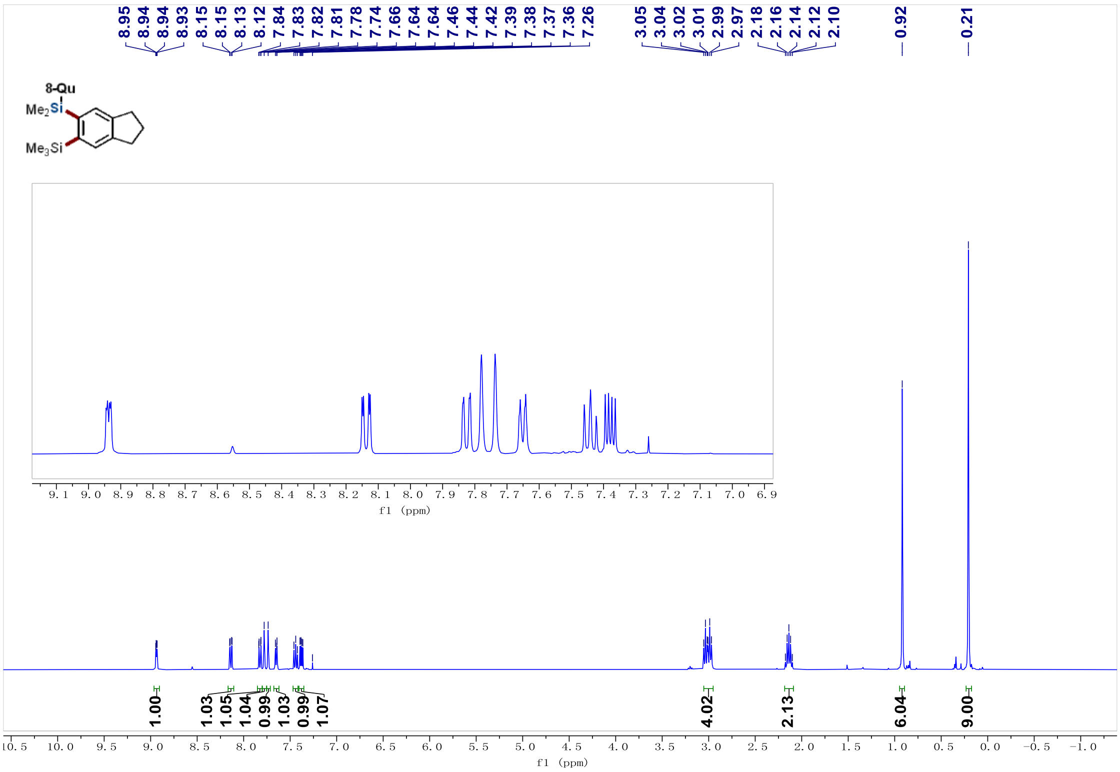

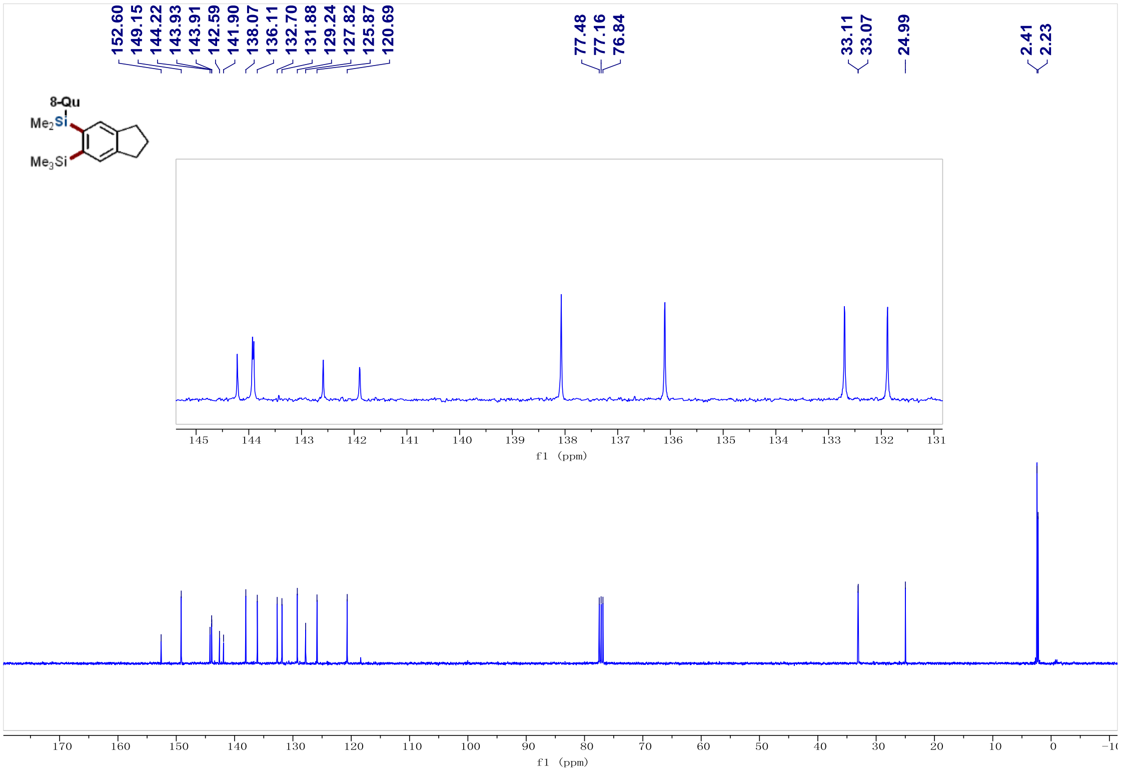

Supplementary Figure 57 ^{1}H and ^{13}C NMR Spectra for compound 6ad

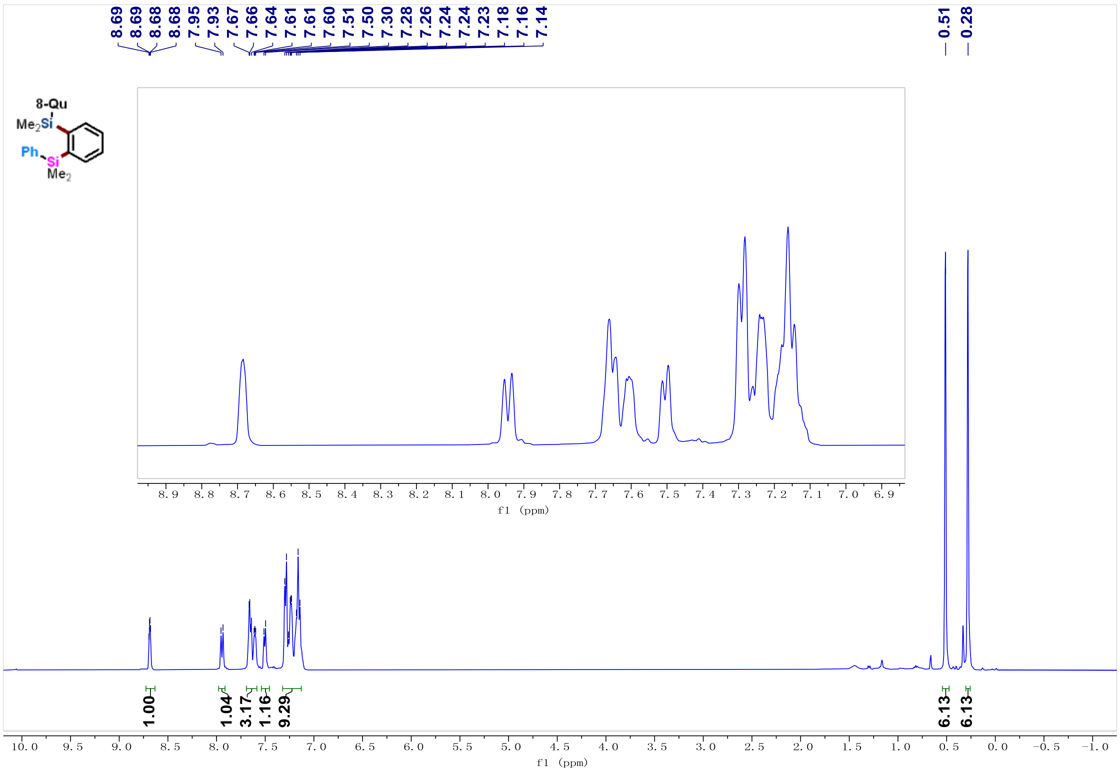

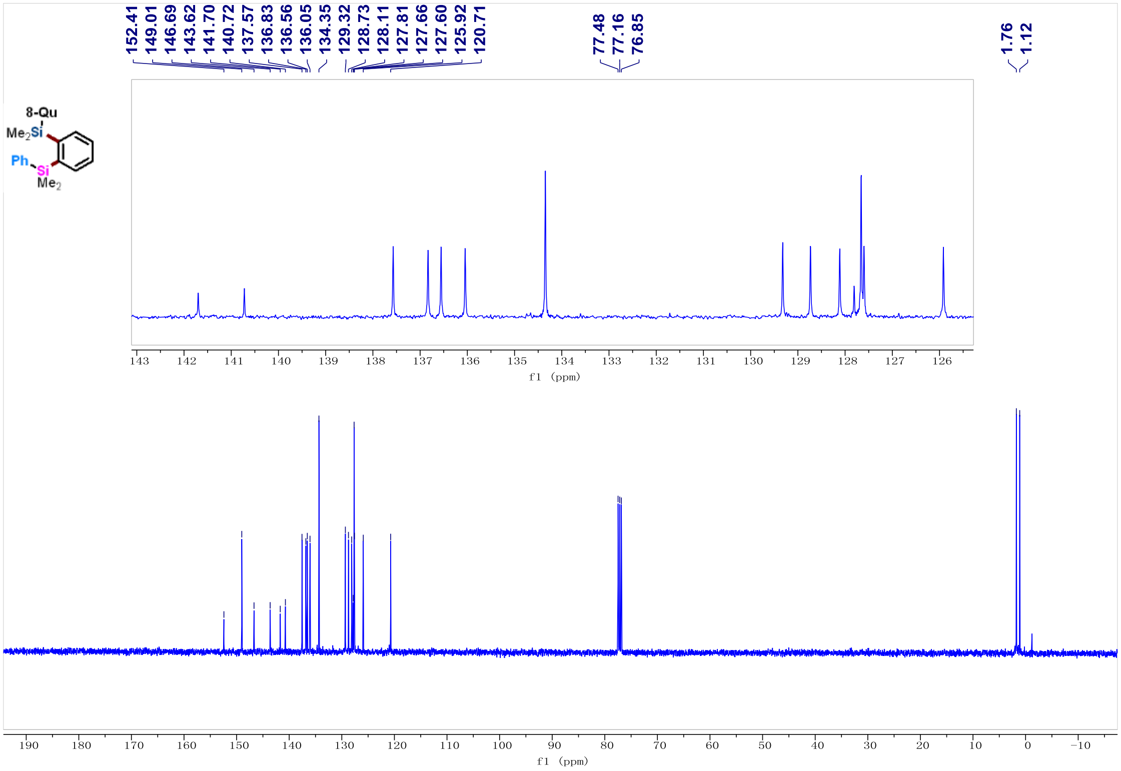

Supplementary Figure 58 ^{1}H and ^{13}C NMR Spectra for compound 6ba

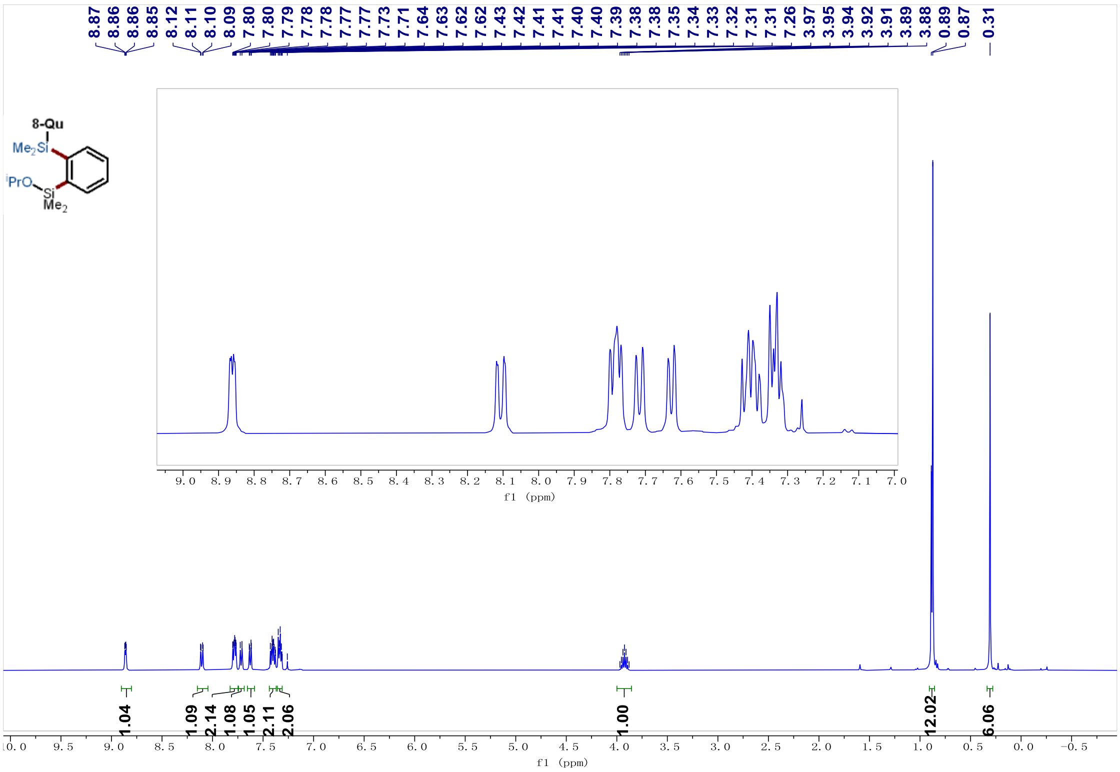

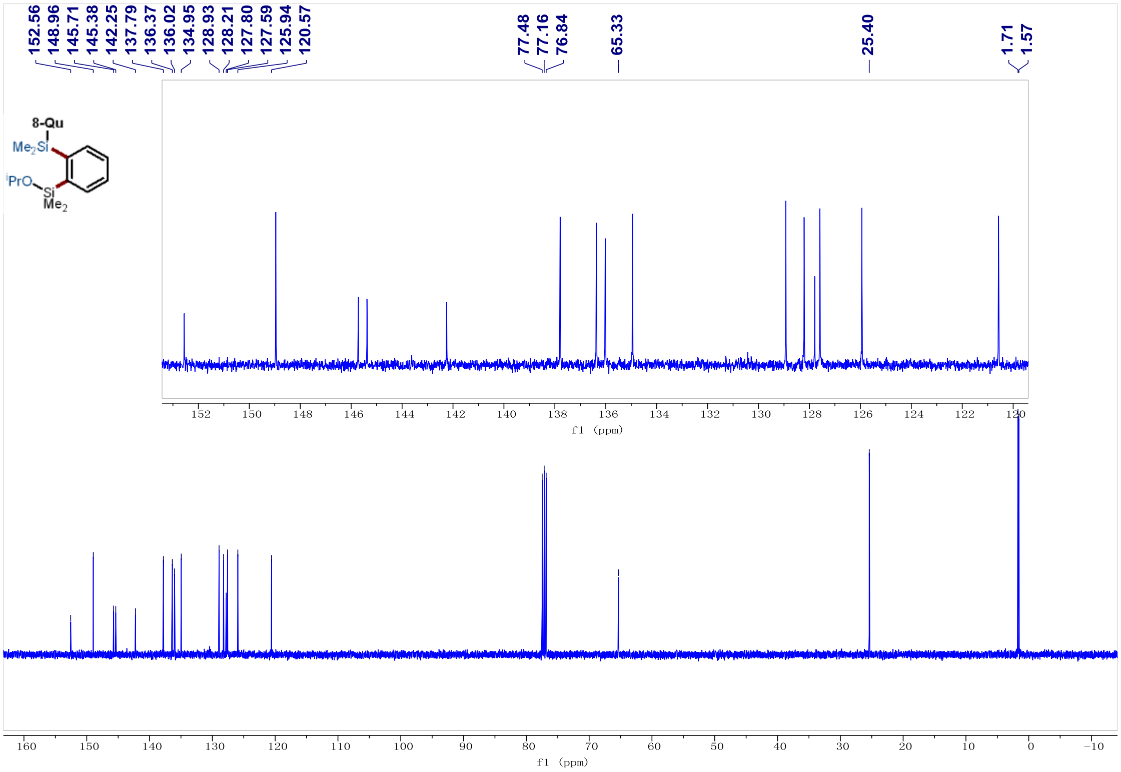

Supplementary Figure 59 ^{1}H and ^{13}C NMR Spectra for compound 6da

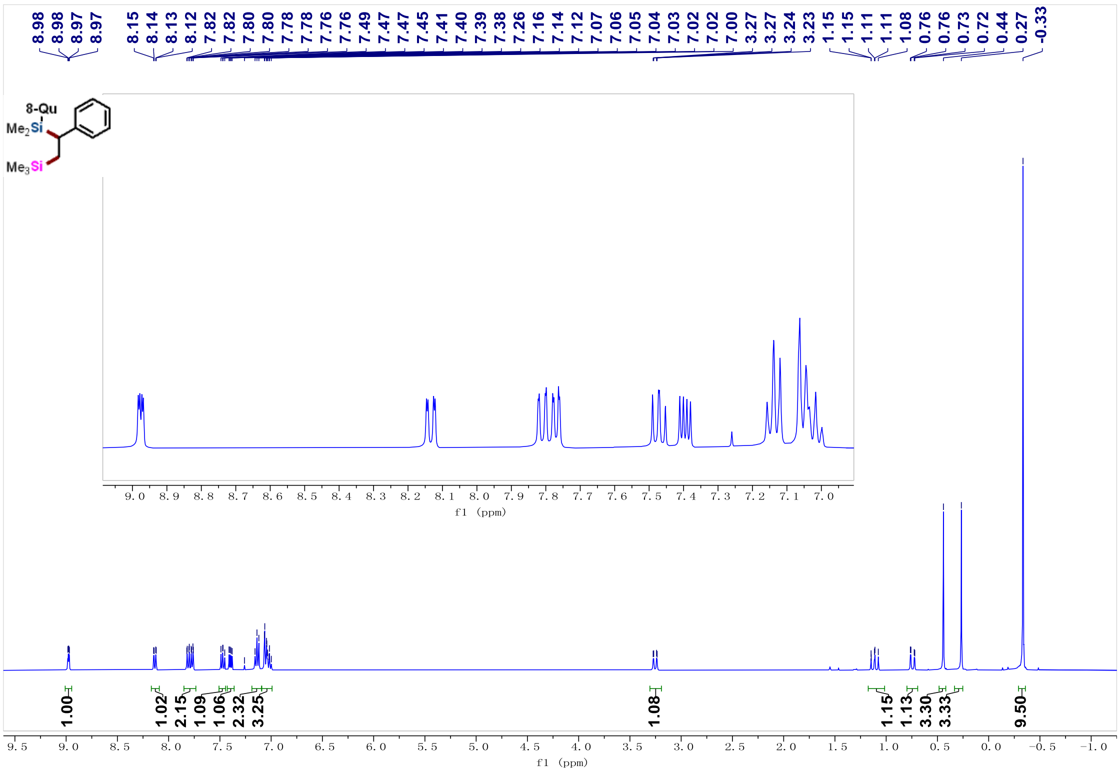

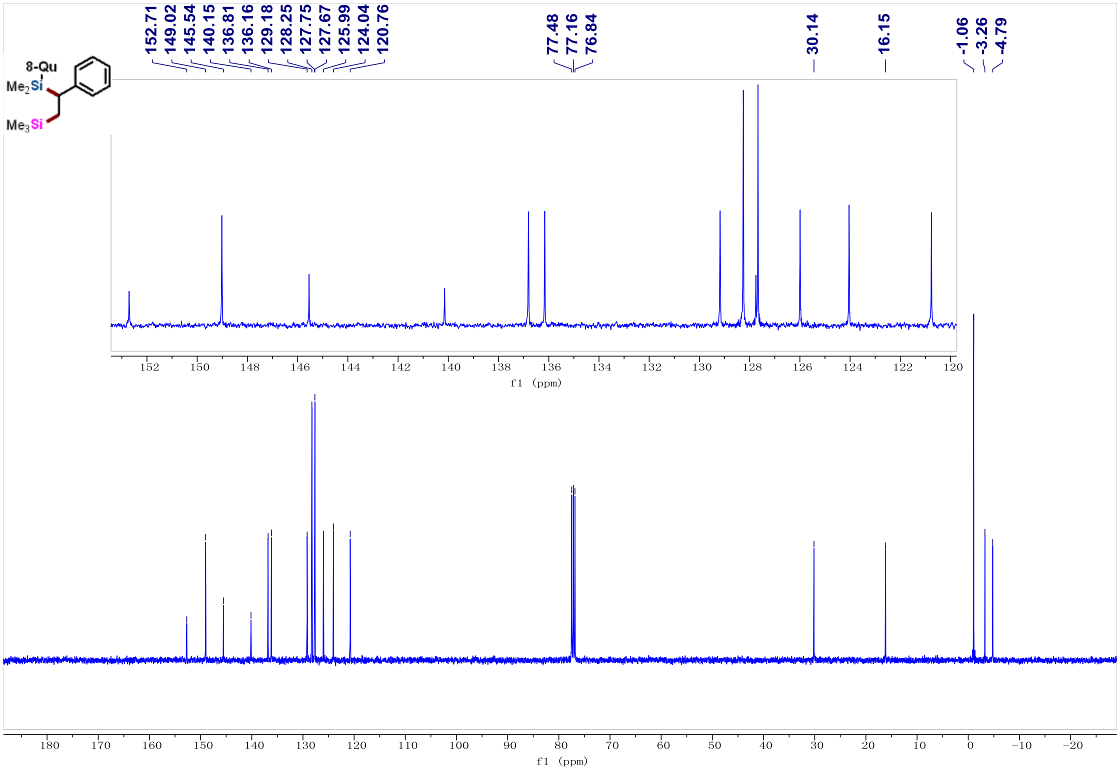

Supplementary Figure 60 ^{1}H and ^{13}C NMR Spectra for compound 8aa

NMR Spectra of 8aa′

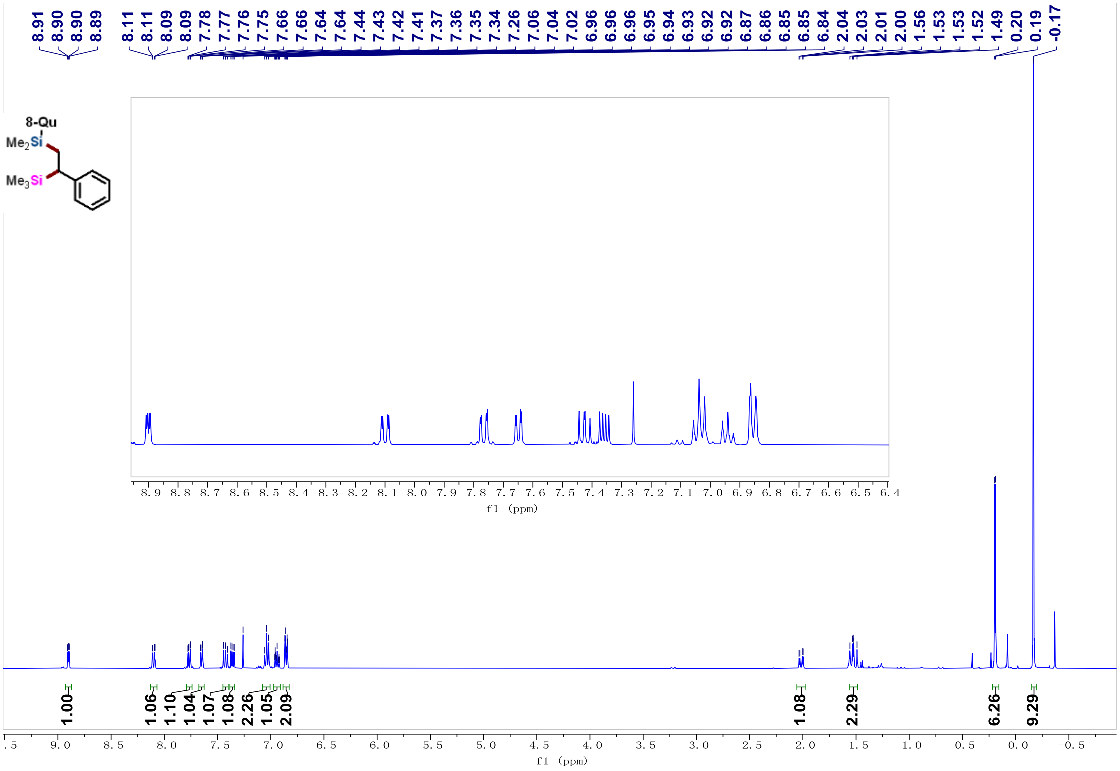

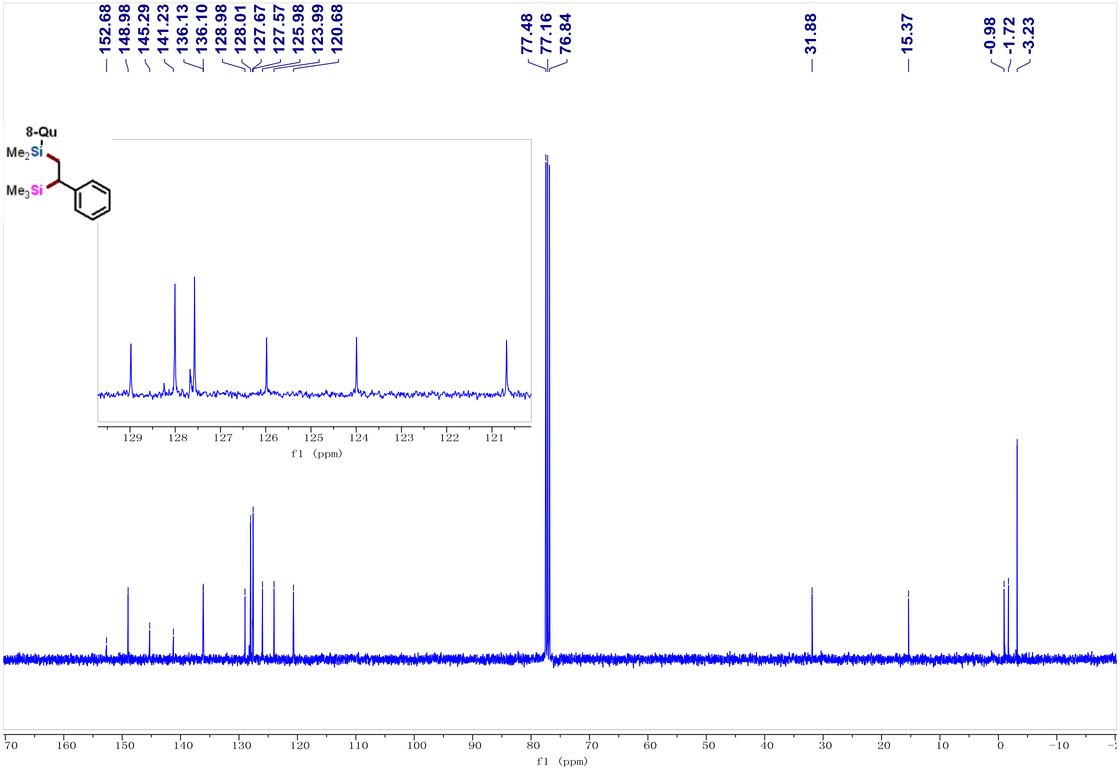

Supplementary Figure 61 ^{1}H and ^{13}C NMR Spectra for compound 8aa′

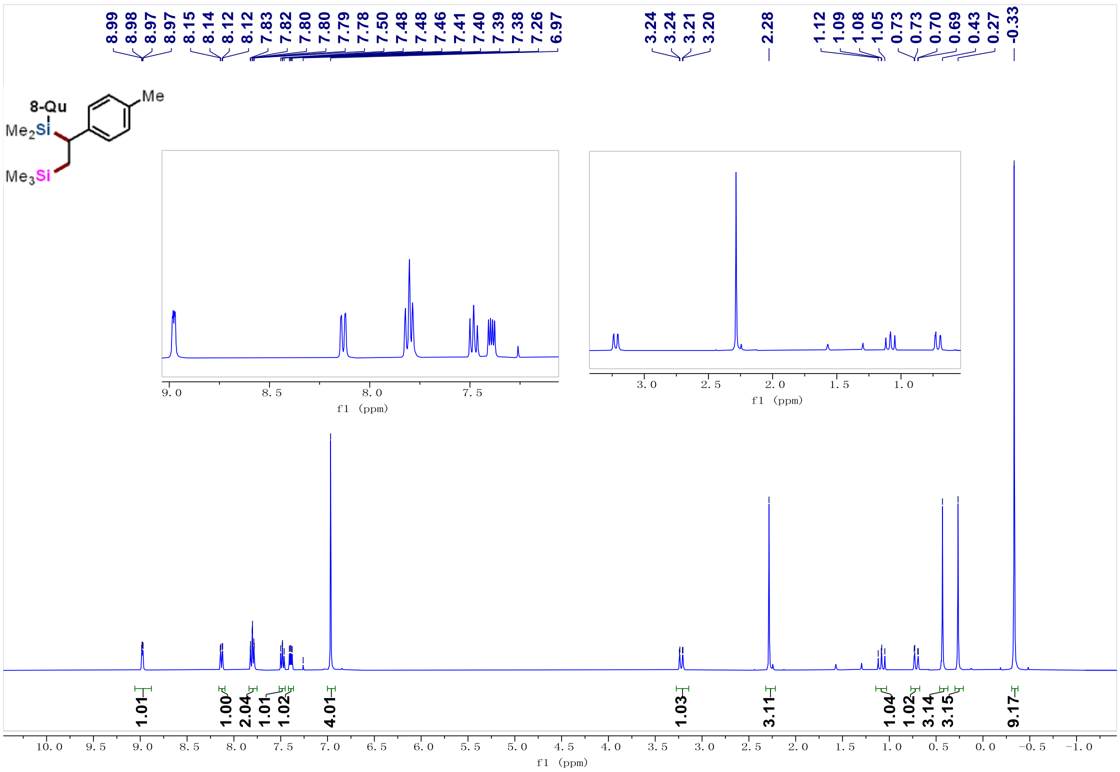

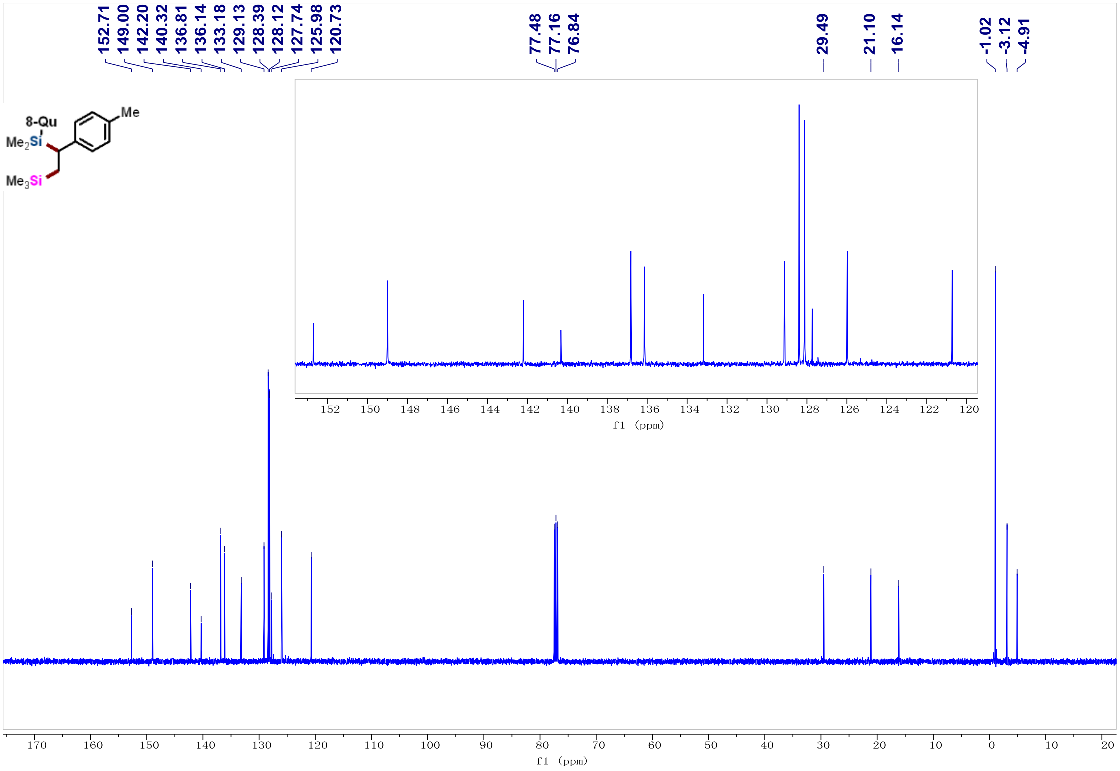

Supplementary Figure 62 ^{1}H and ^{13}C NMR Spectra for compound 8ab

COSY (8ab)

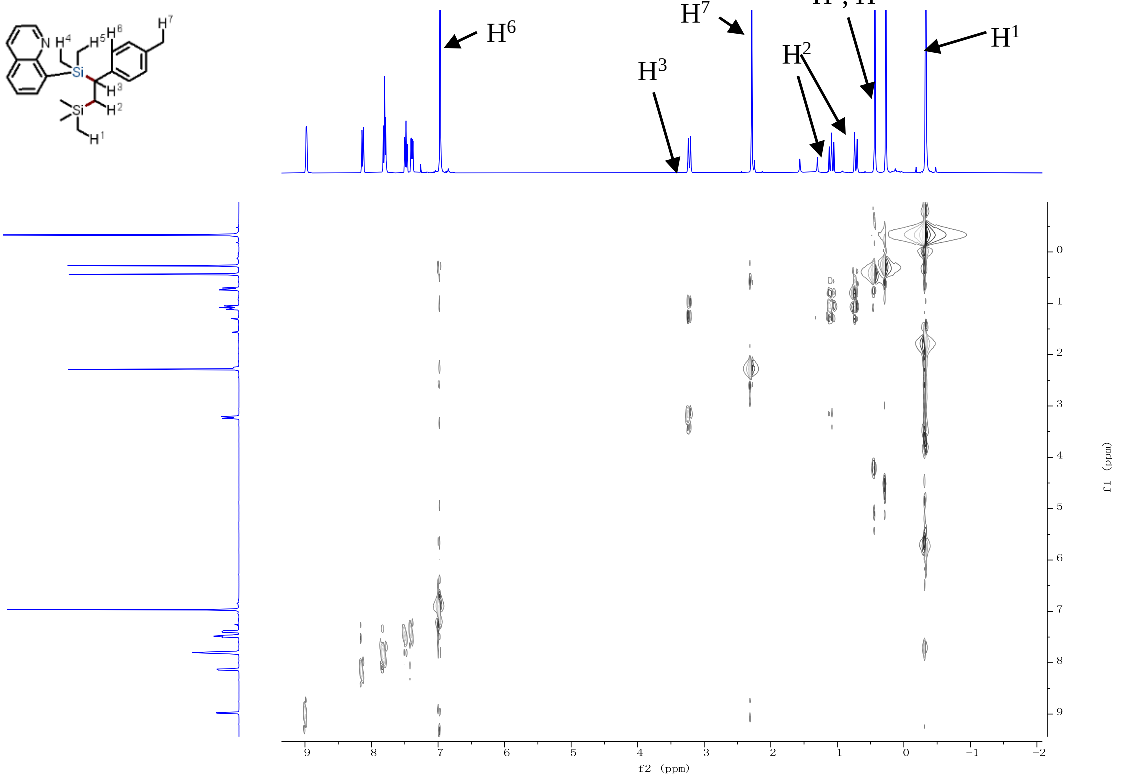

HSQC (8ab)

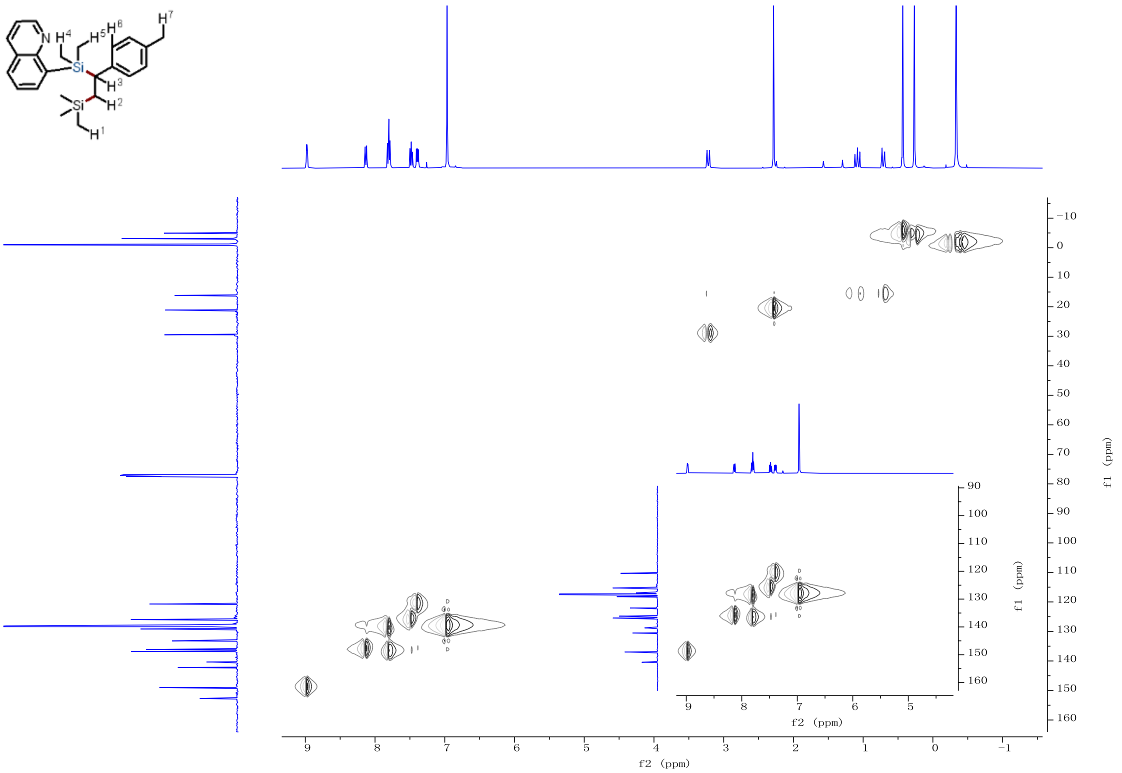

NOESY (8ab)

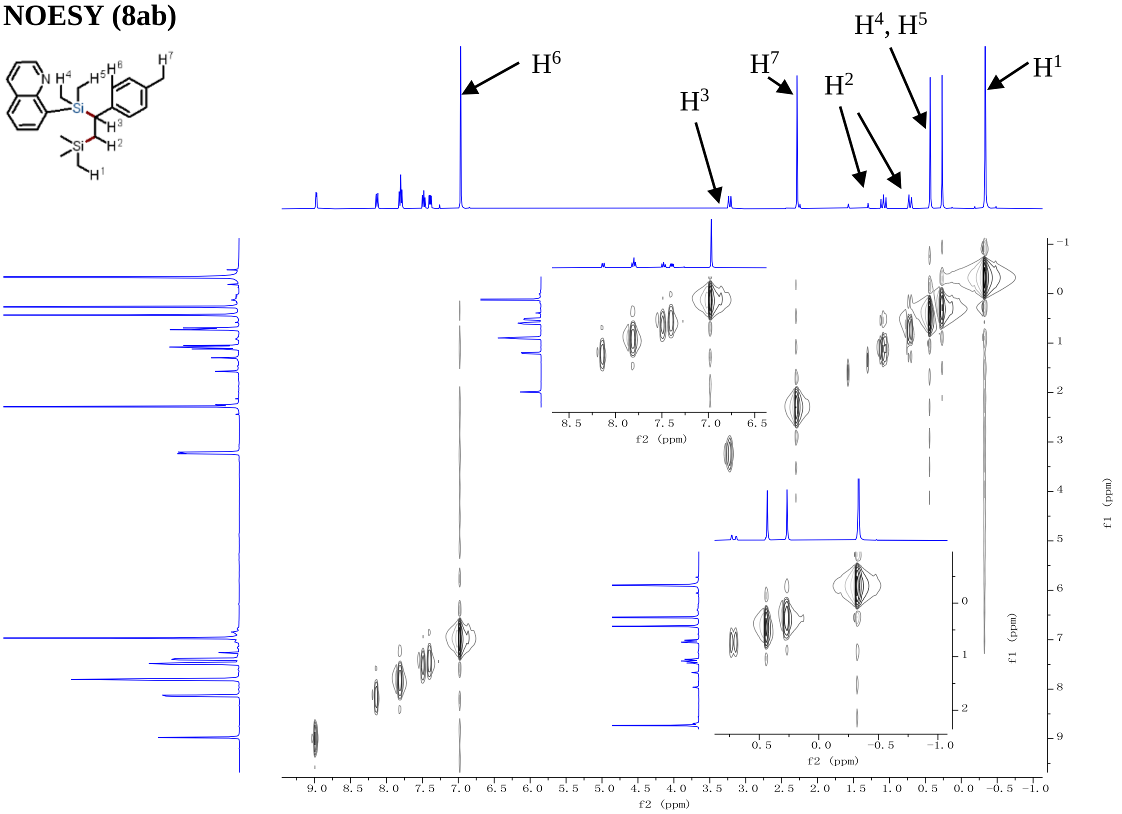

Supplementary Figure 63 COSY, HSQC and NOESY Spectra for compound 8ab

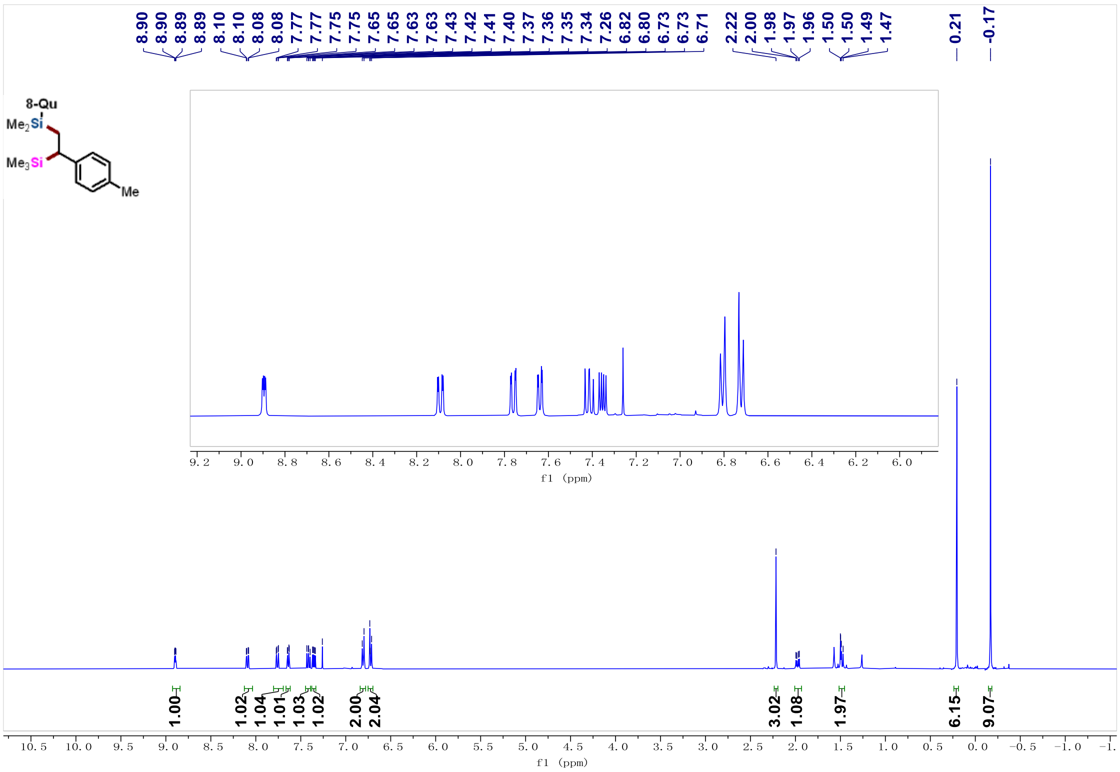

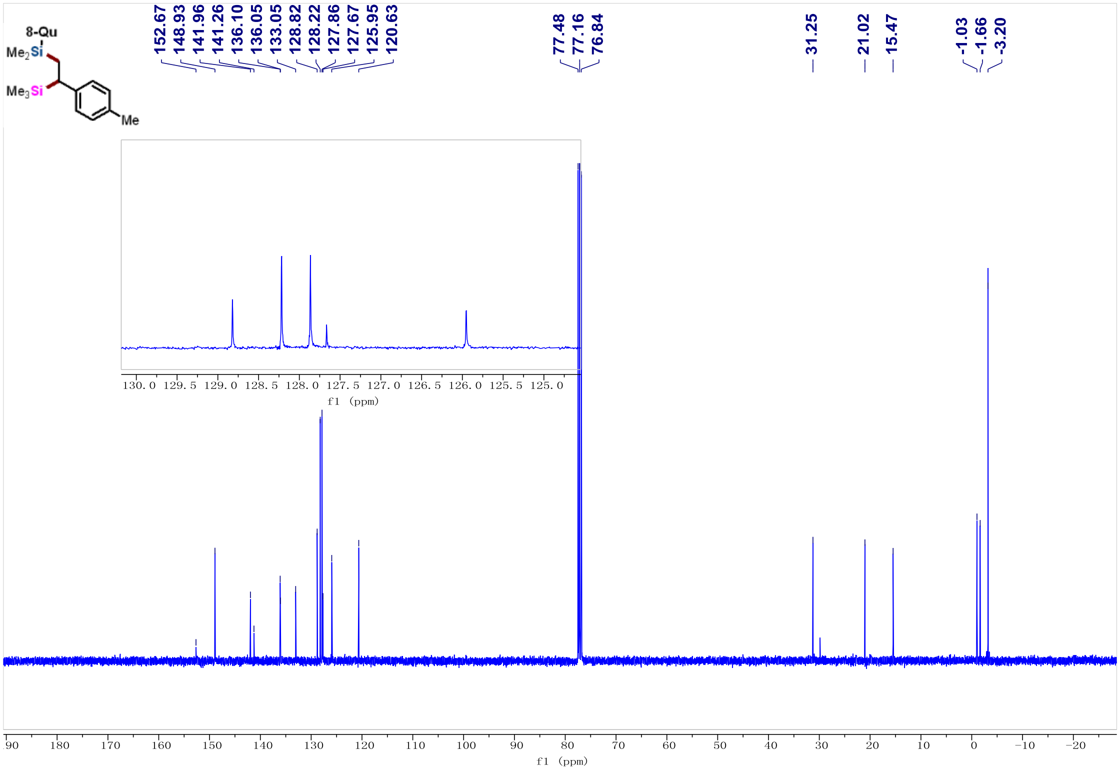

Supplementary Figure 64 ^{1}H and ^{13}C NMR Spectra for compound 8ab′

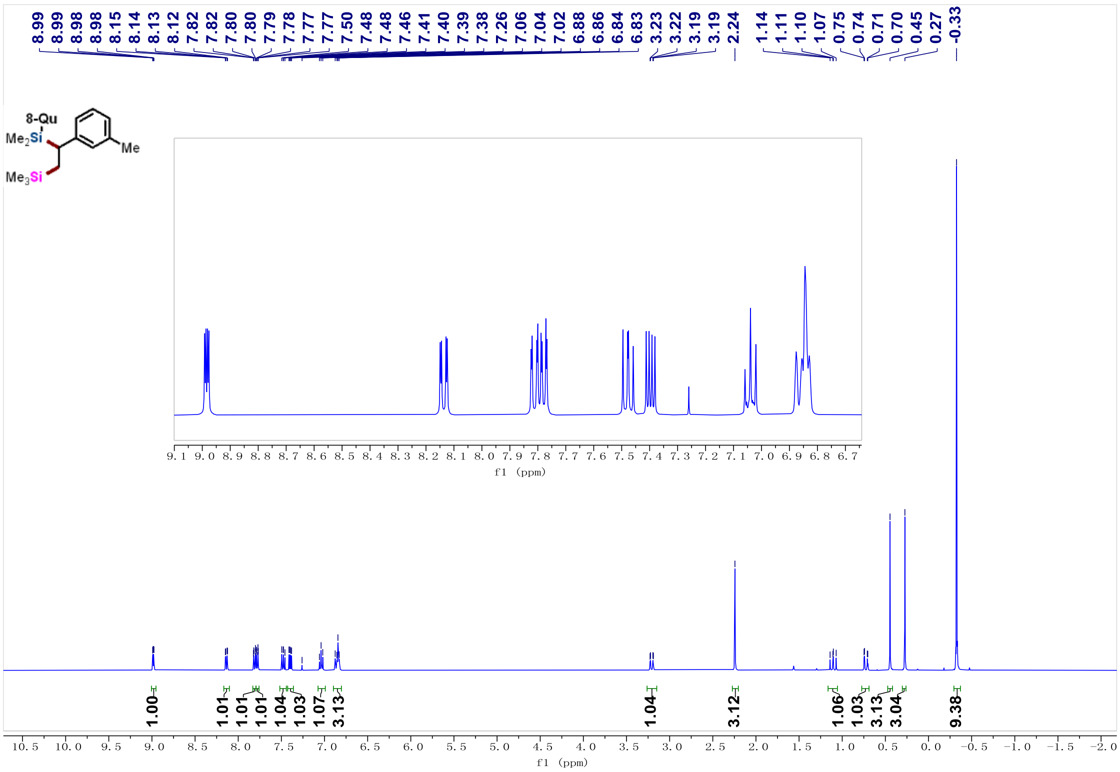

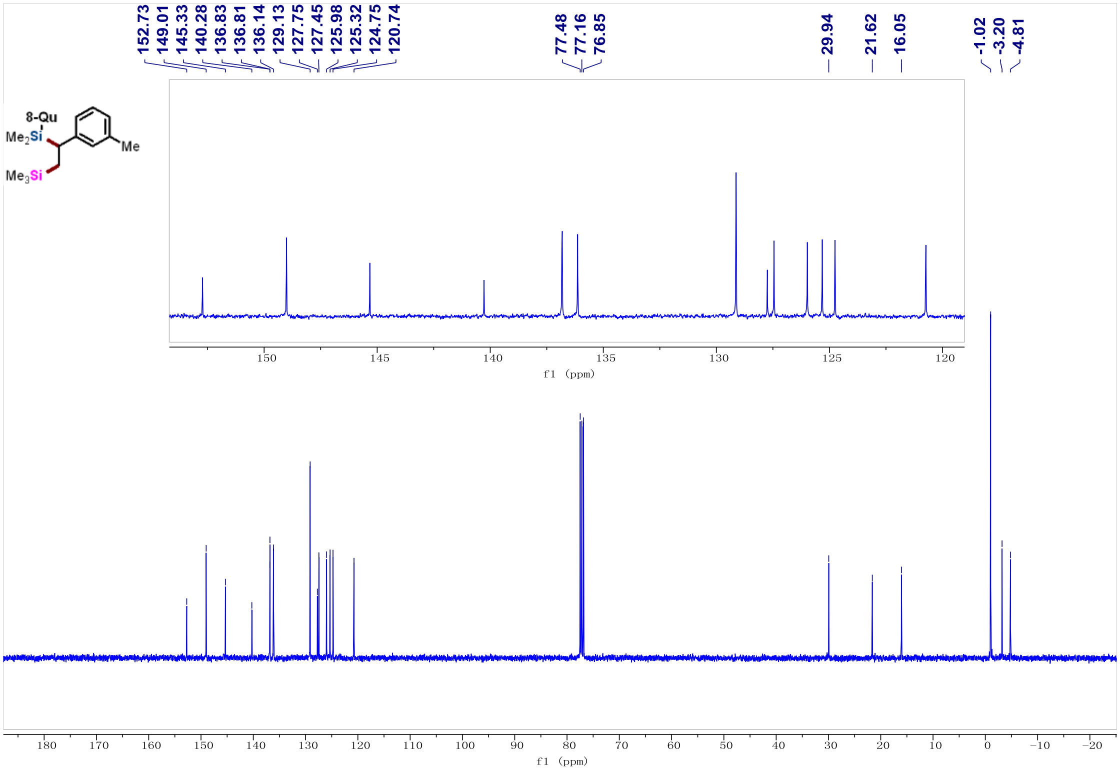

Supplementary Figure 65 ^{1}H and ^{13}C NMR Spectra for compound 8ac

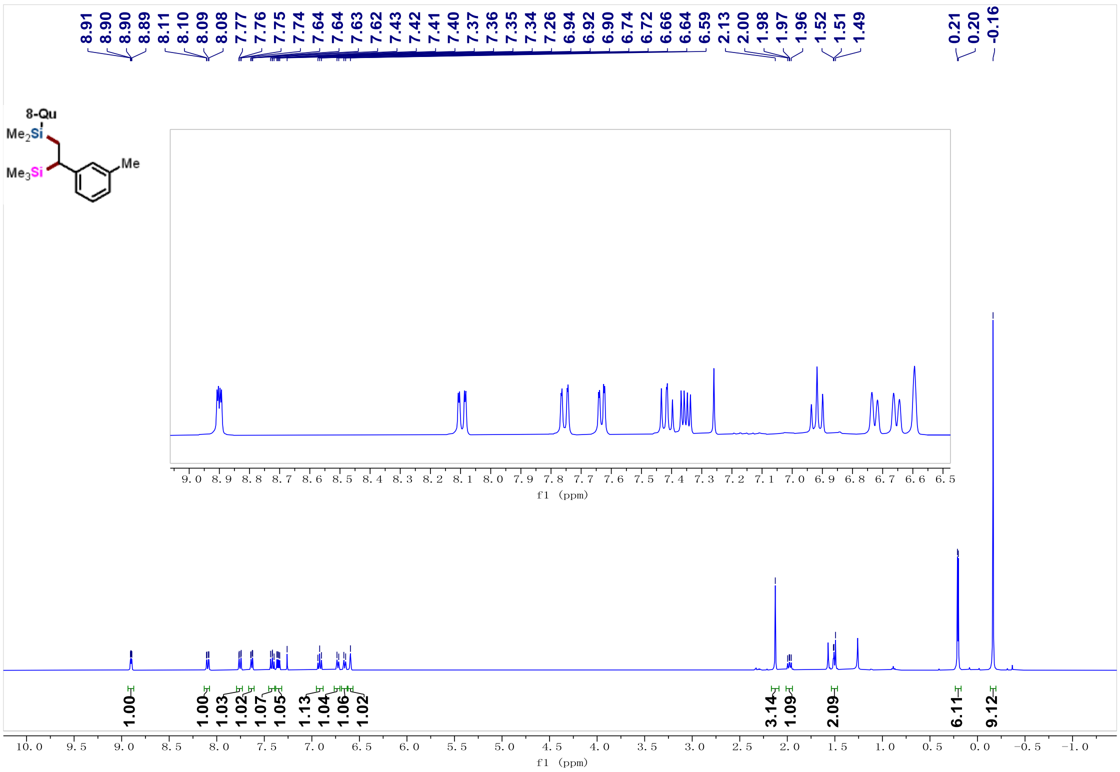

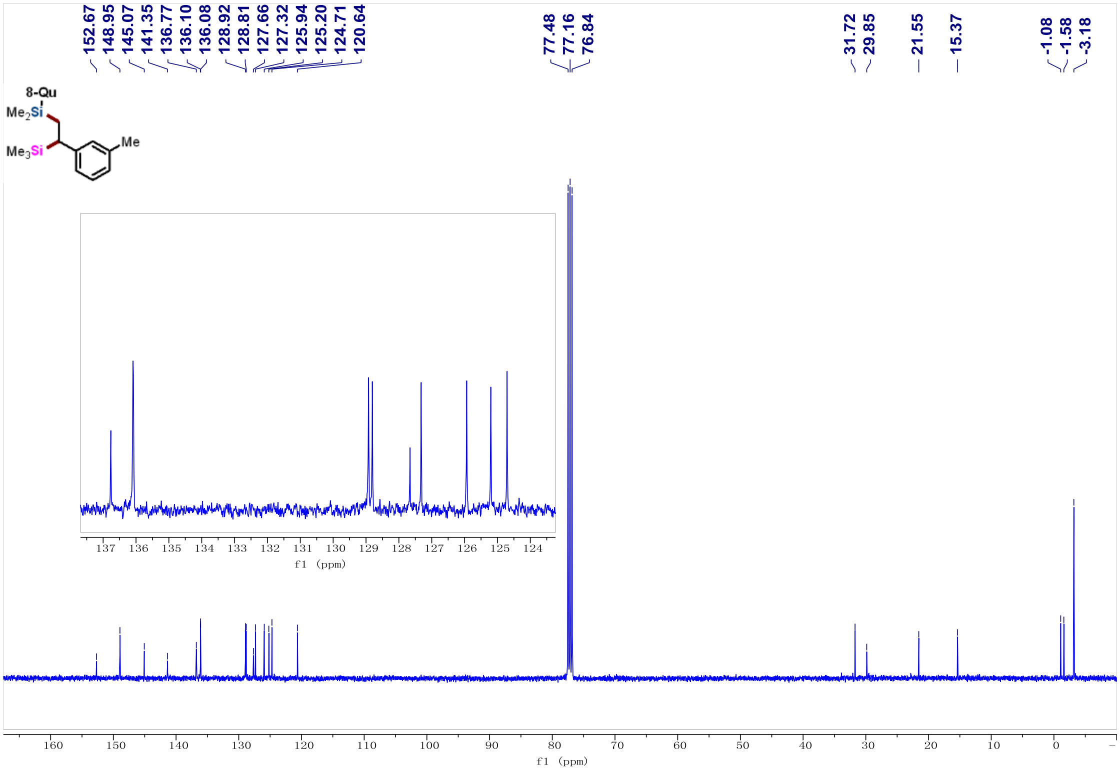

Supplementary Figure 66 ^{1}H and ^{13}C NMR Spectra for compound 8ac′

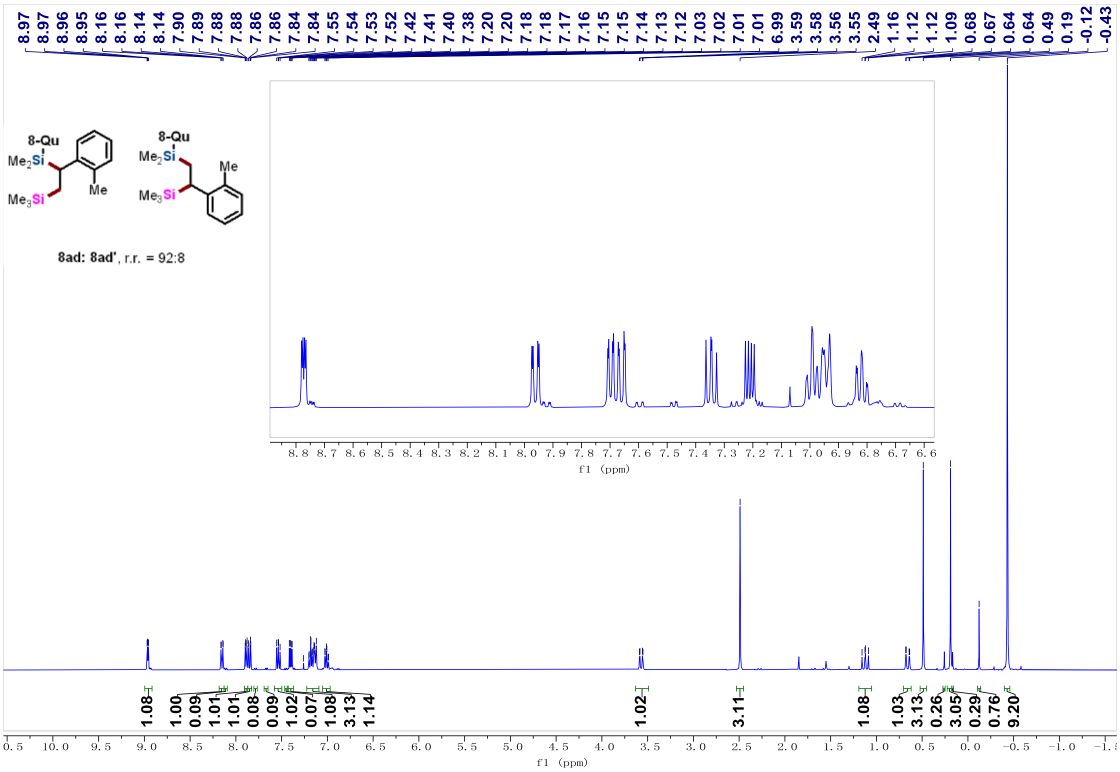

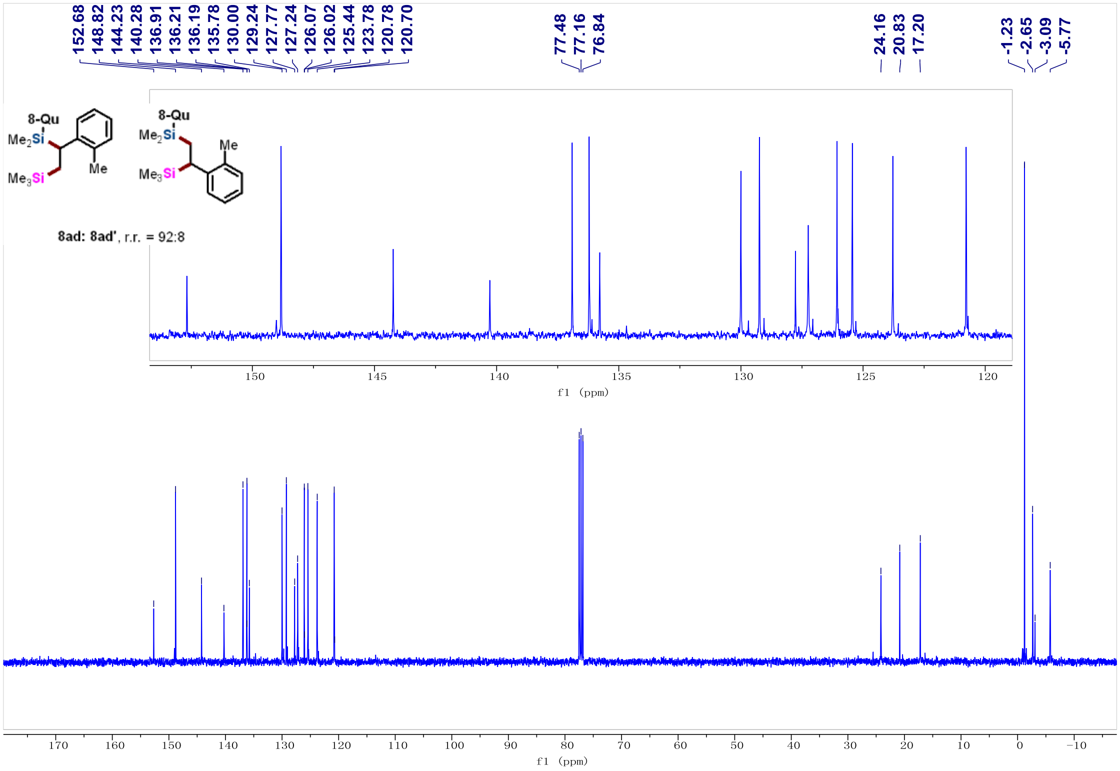

Supplementary Figure 67 ^{1}H and ^{13}C NMR Spectra for compound 8ad

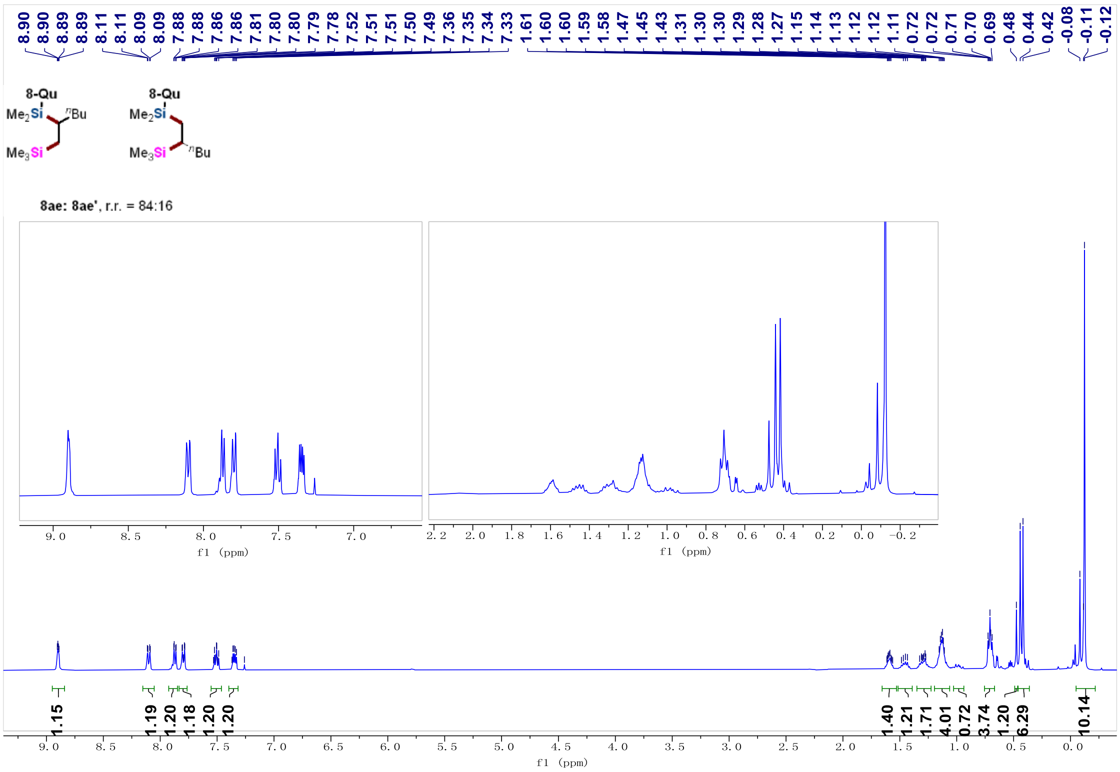

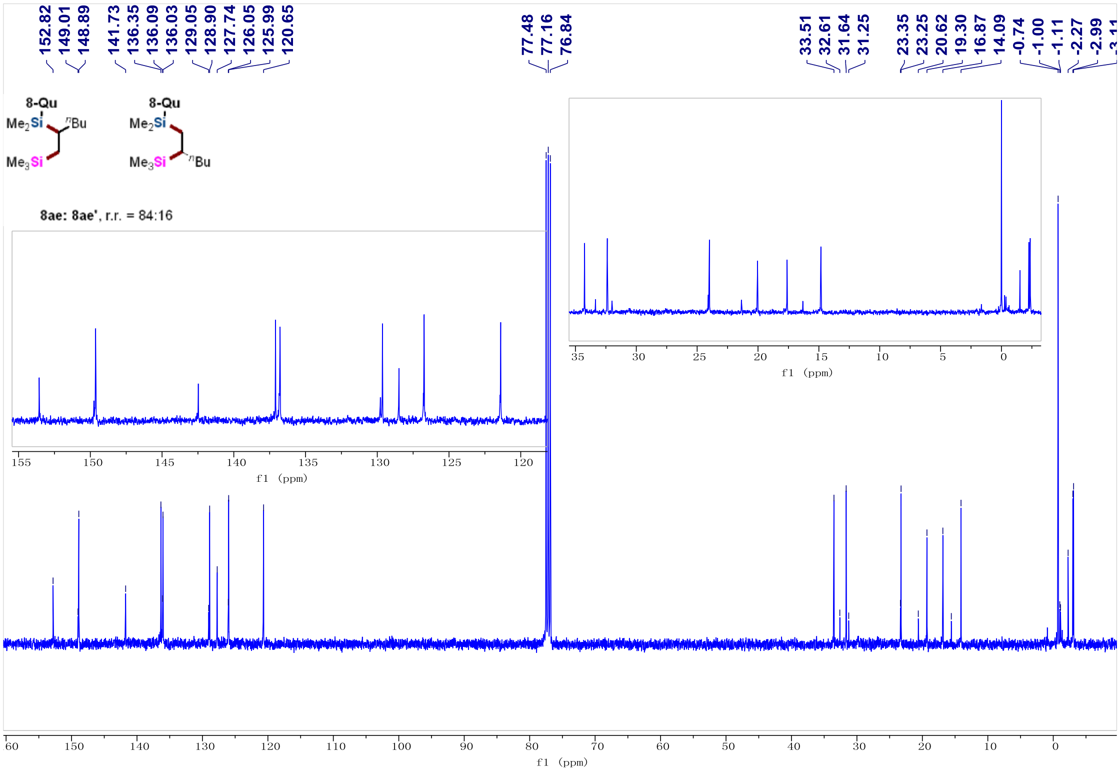

Supplementary Figure 68 ^{1}H and ^{13}C NMR Spectra for compound 8ae

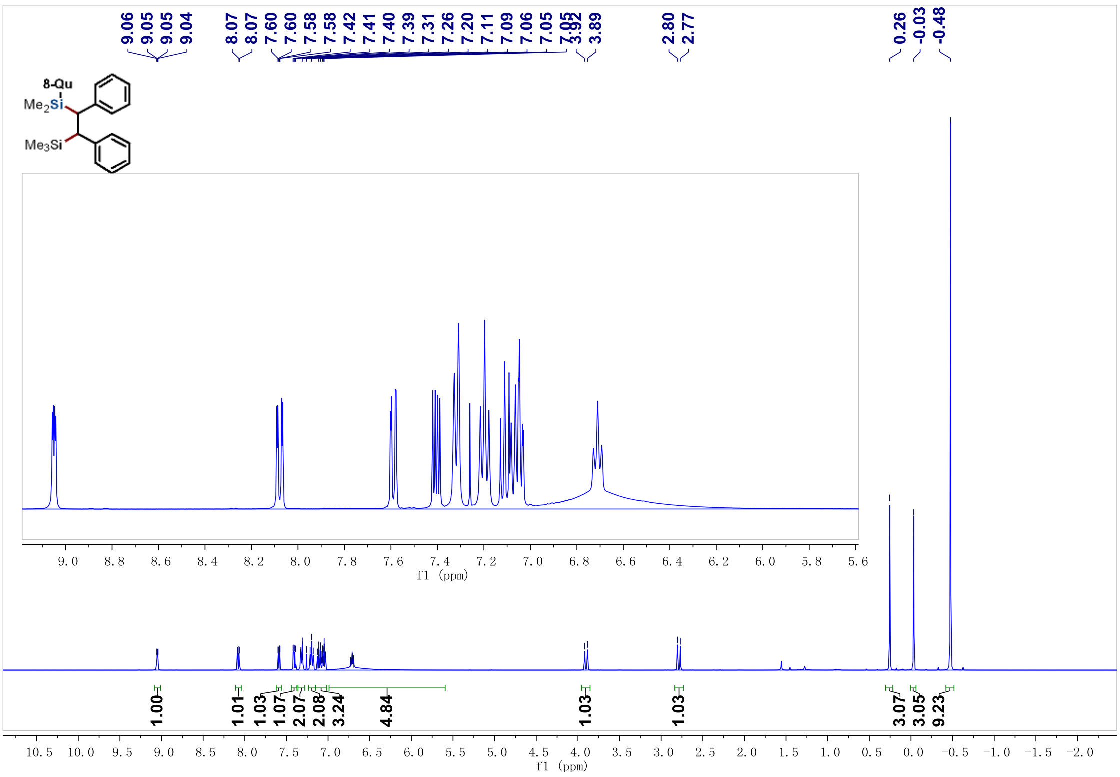

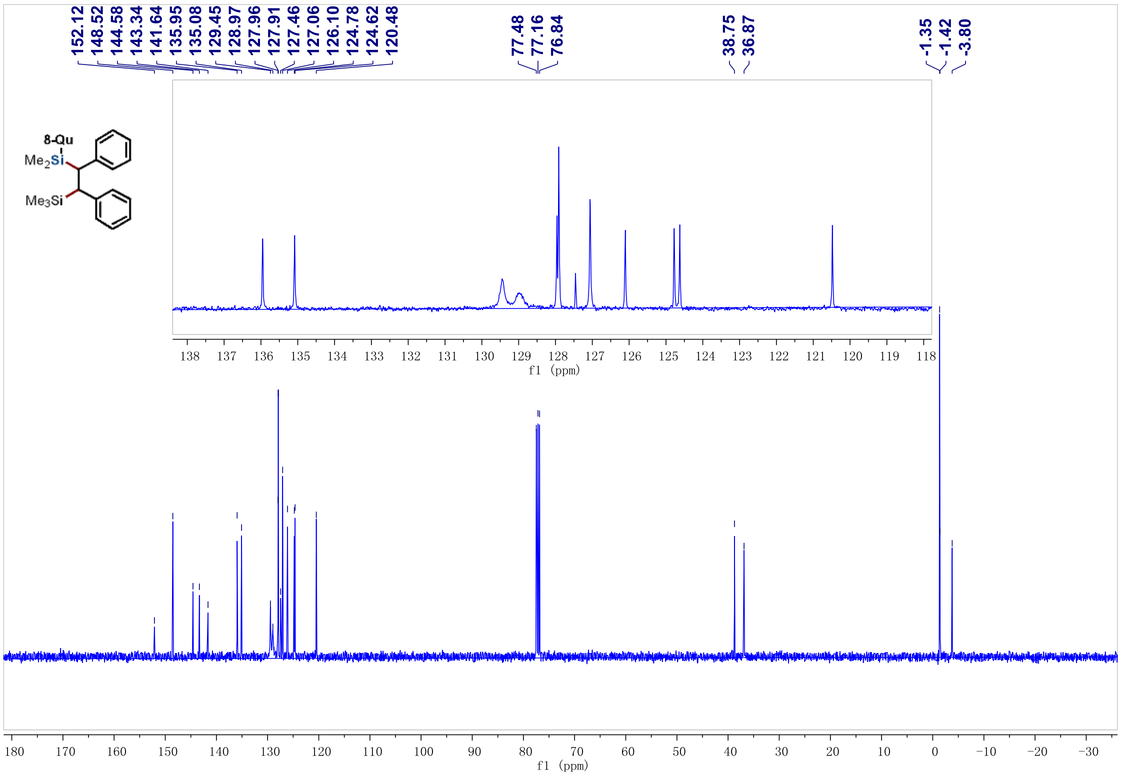

Supplementary Figure 69 ^{1}H and ^{13}C NMR Spectra for compound 8af

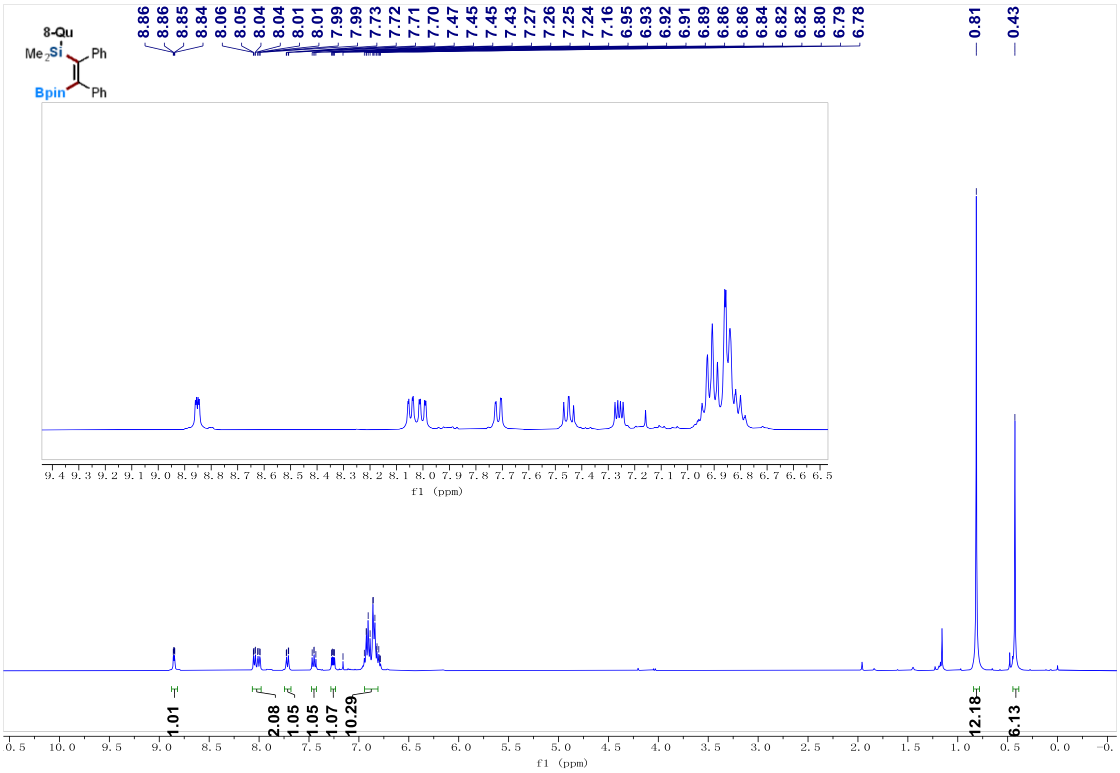

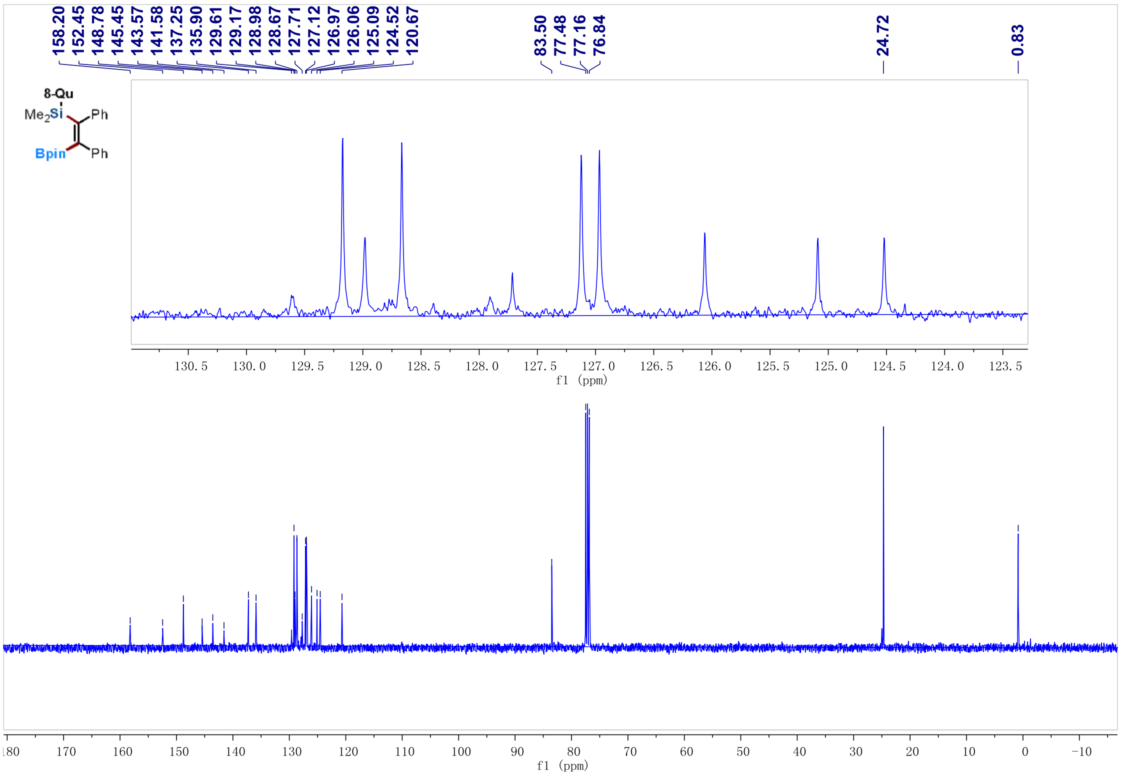

Supplementary Figure 70 ^{1}H and ^{13}C NMR Spectra for compound 9

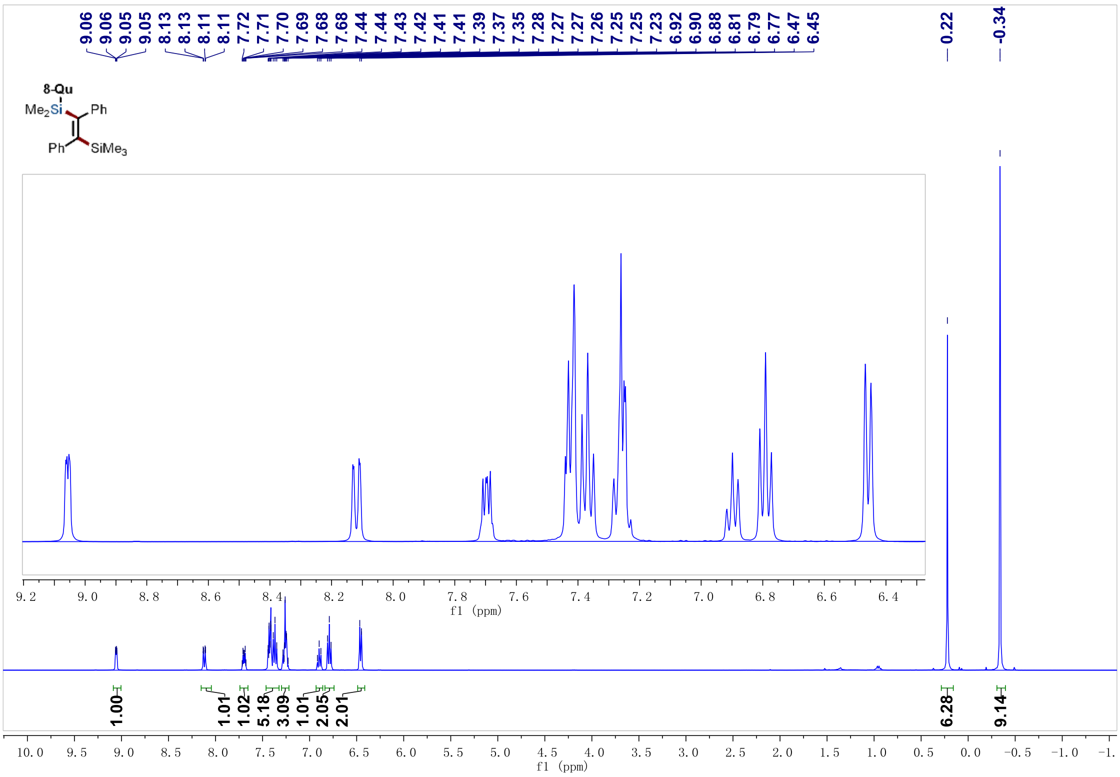

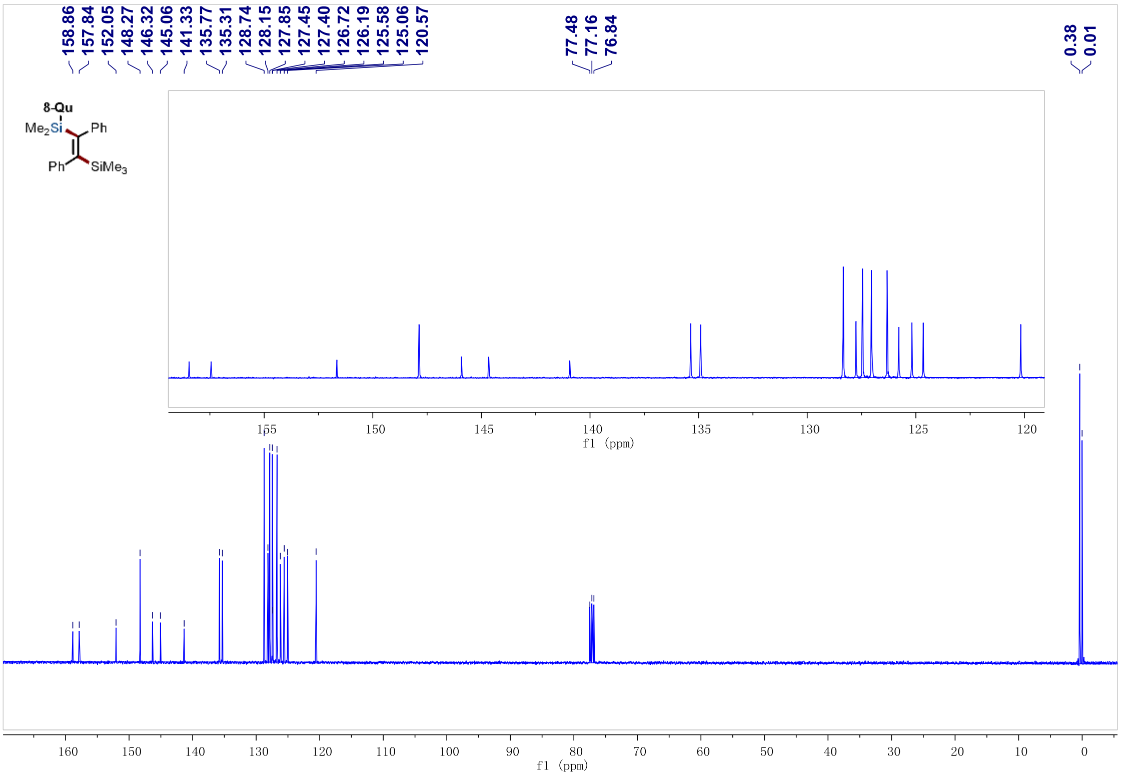

Supplementary Figure 71 ^{1}H and ^{13}C NMR Spectra for compound 10

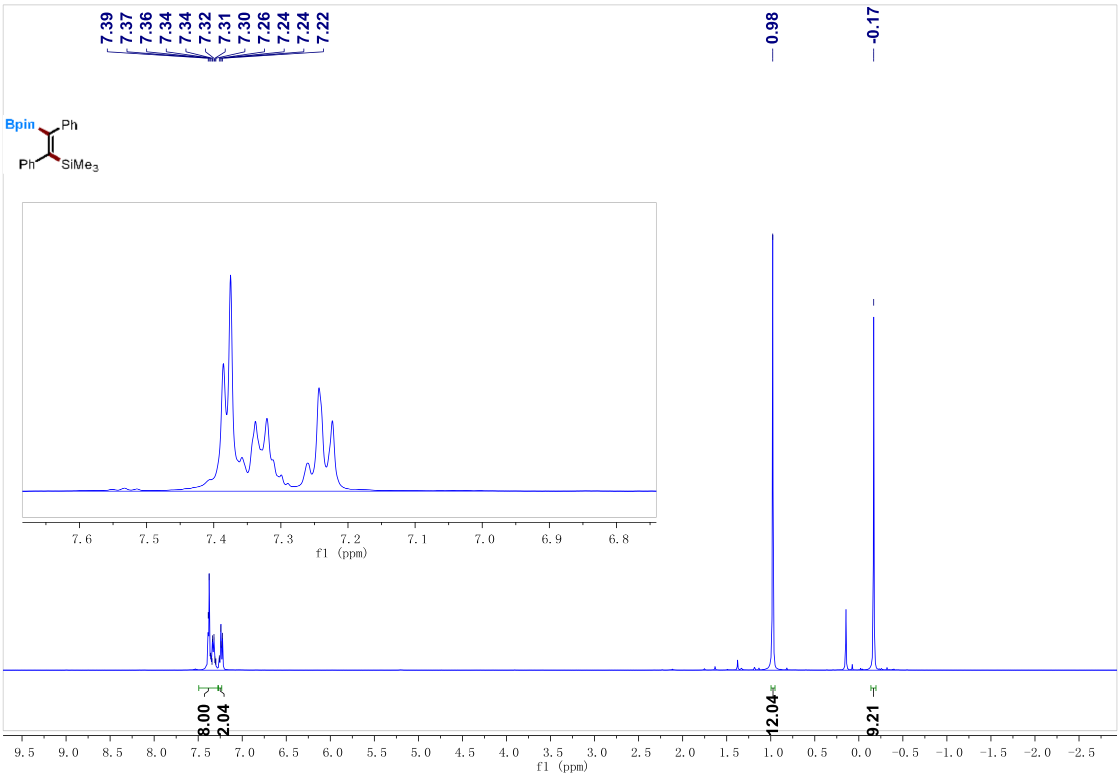

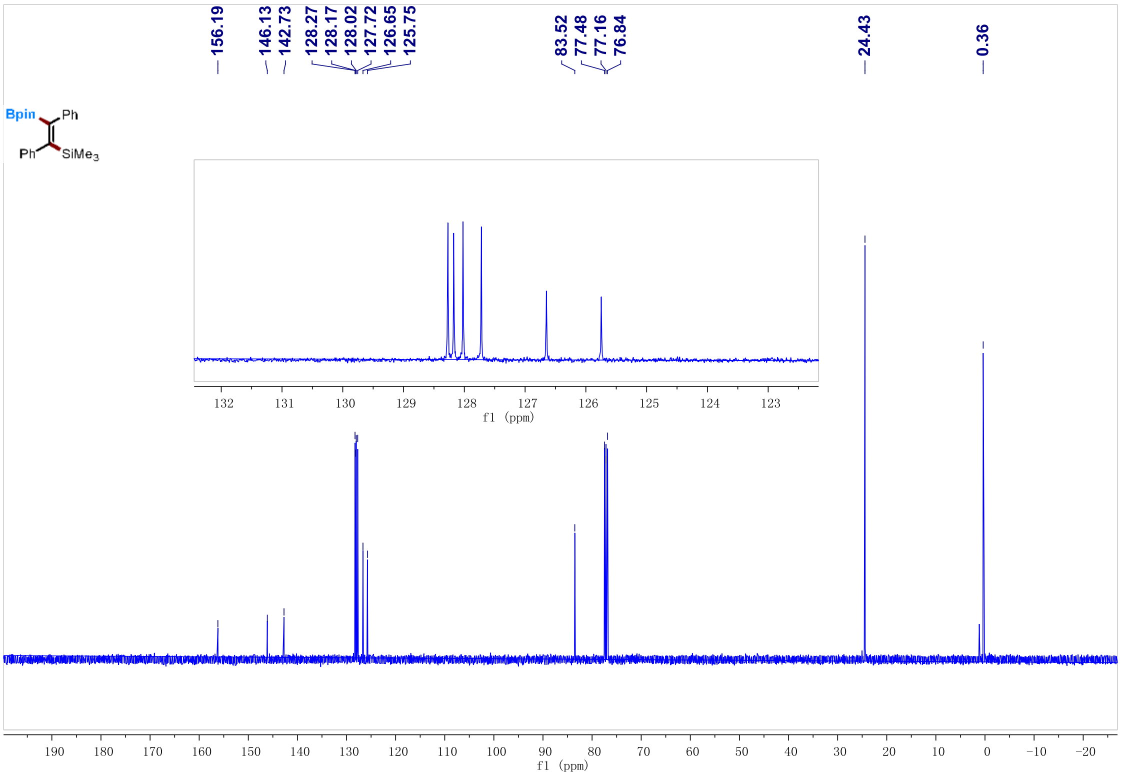

Supplementary Figure 72 ^{1}H and ^{13}C NMR Spectra for compound 11

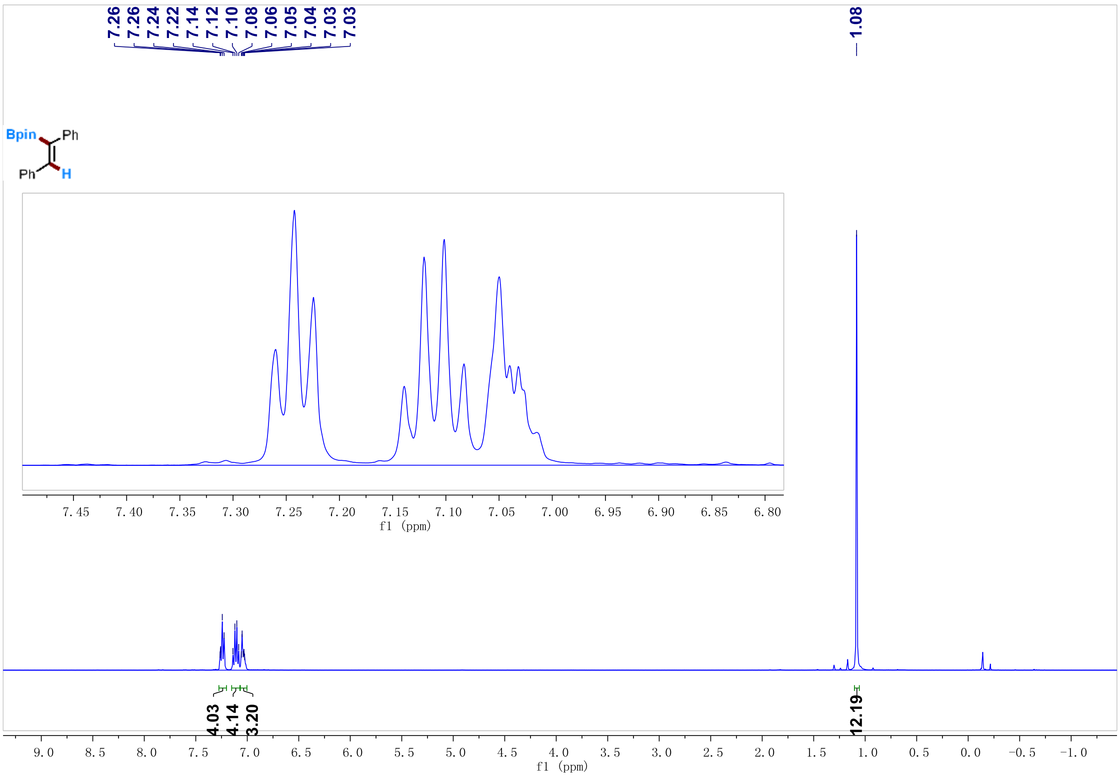

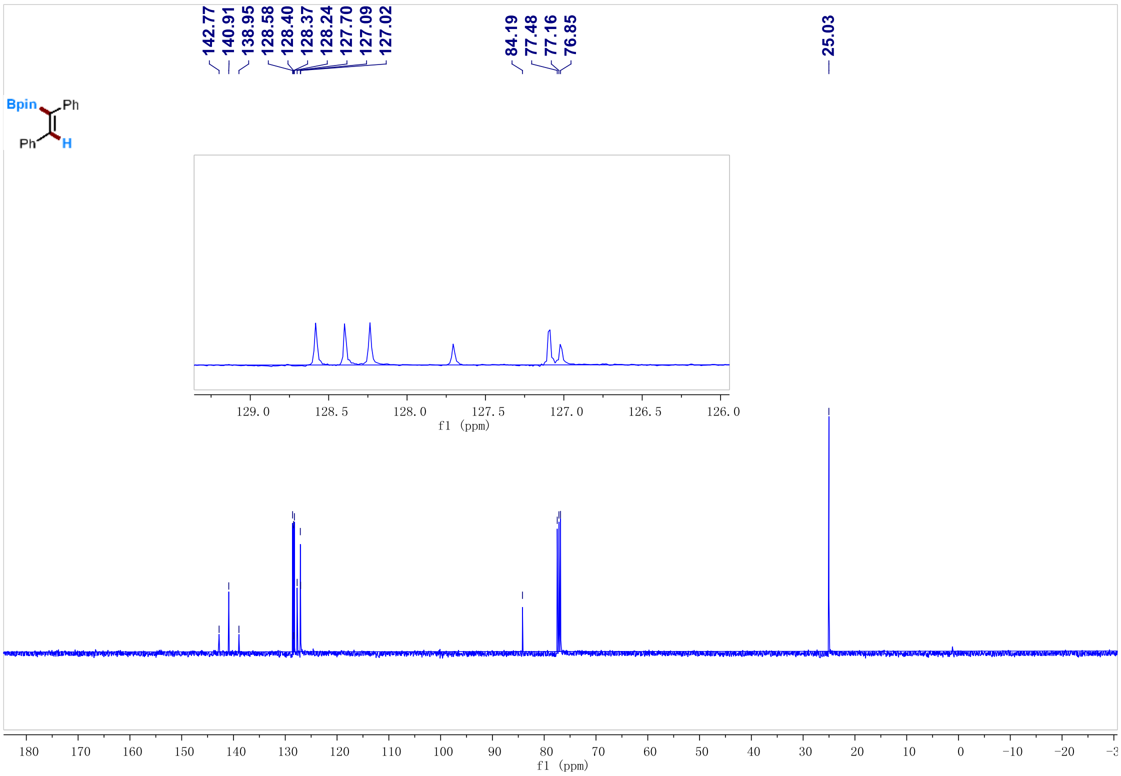

Supplementary Figure 73 ^{1}H and ^{13}C NMR Spectra for compound 12

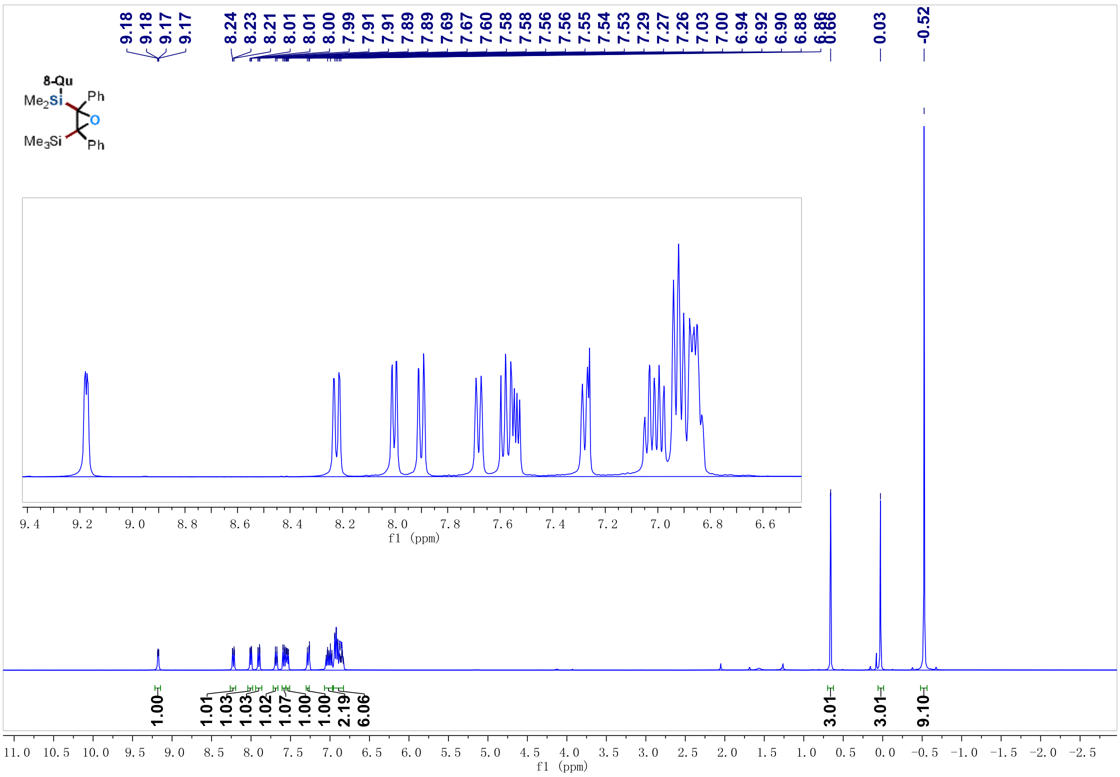

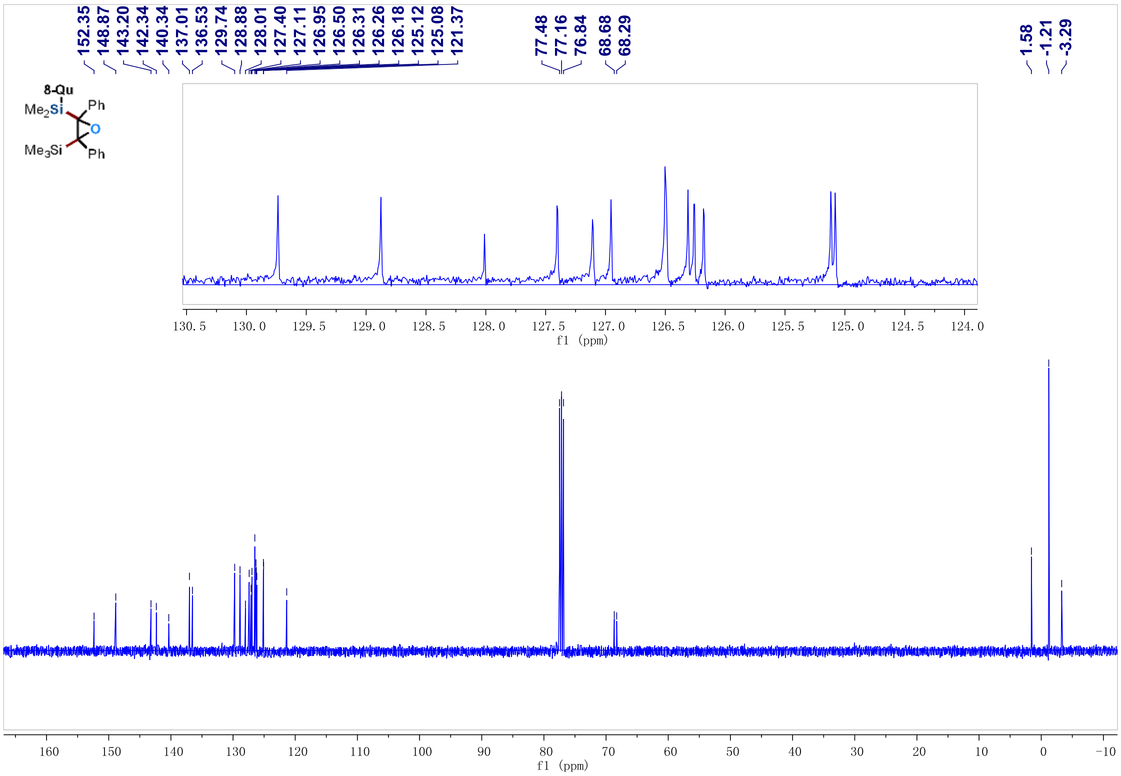

Supplementary Figure 74 ^{1}H and ^{13}C NMR Spectra for compound 13

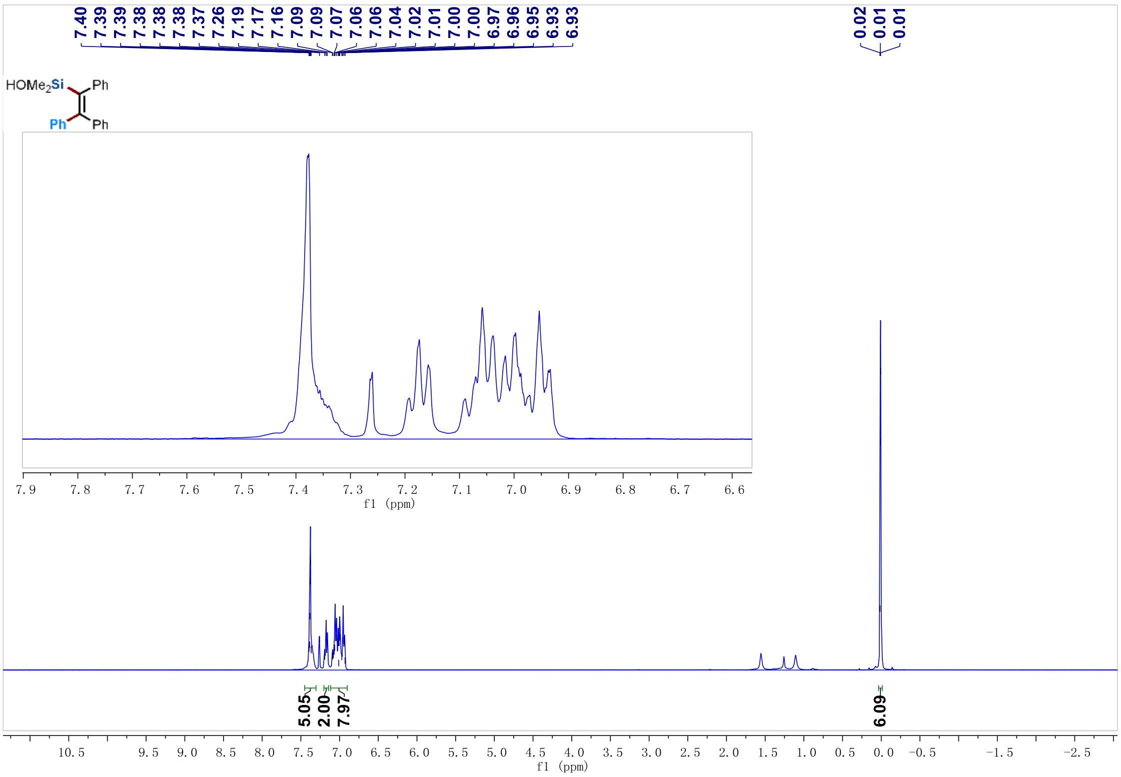

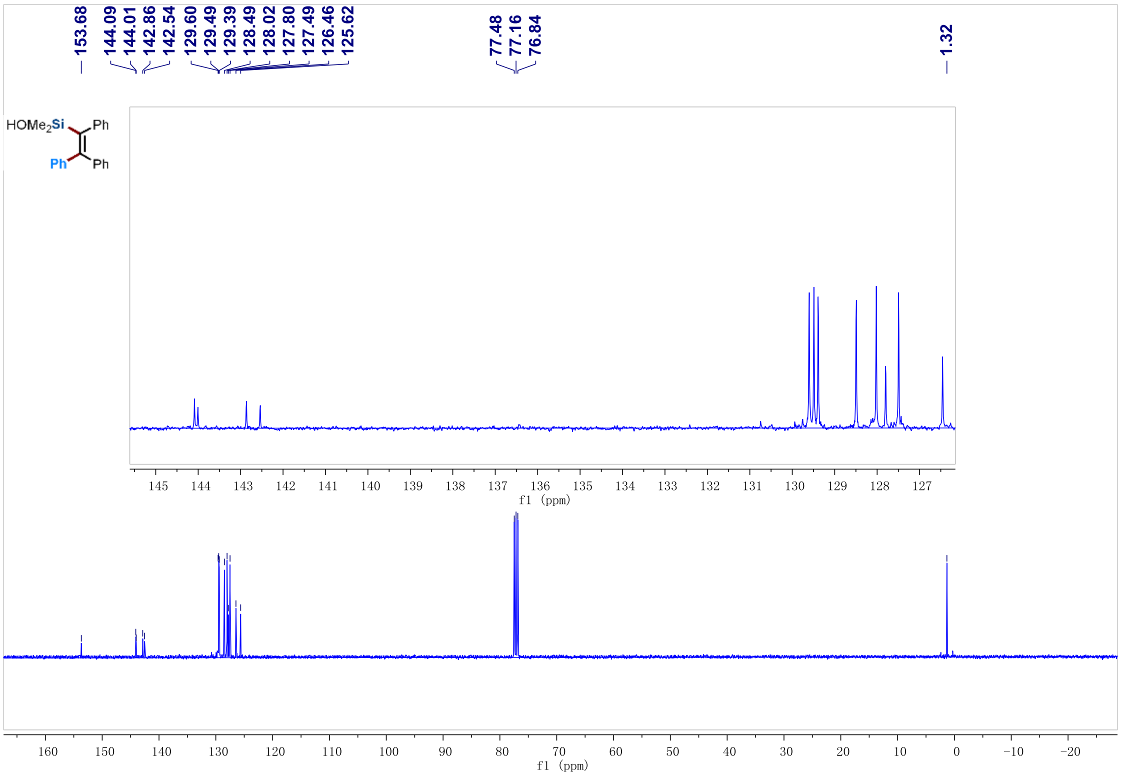

Supplementary Figure 75 ^{1}H and ^{13}C NMR Spectra for compound 14

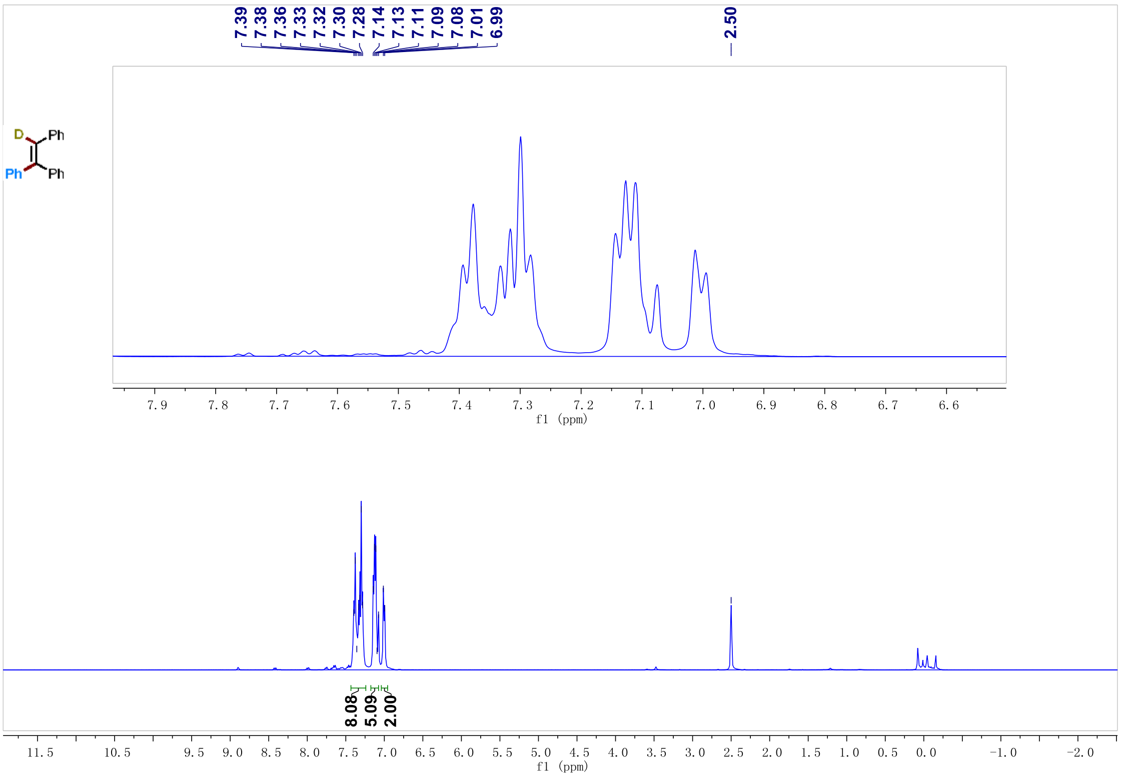

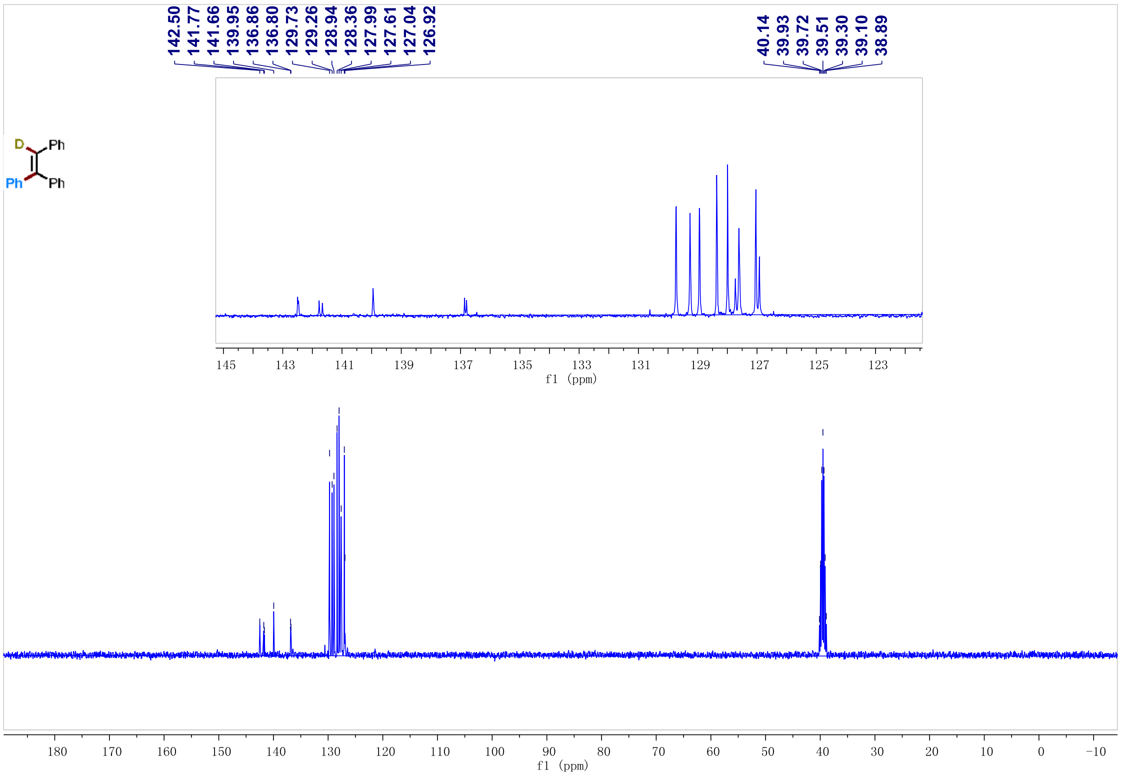

Supplementary Figure 76 ^{1}H and ^{13}C NMR Spectra for compound 15

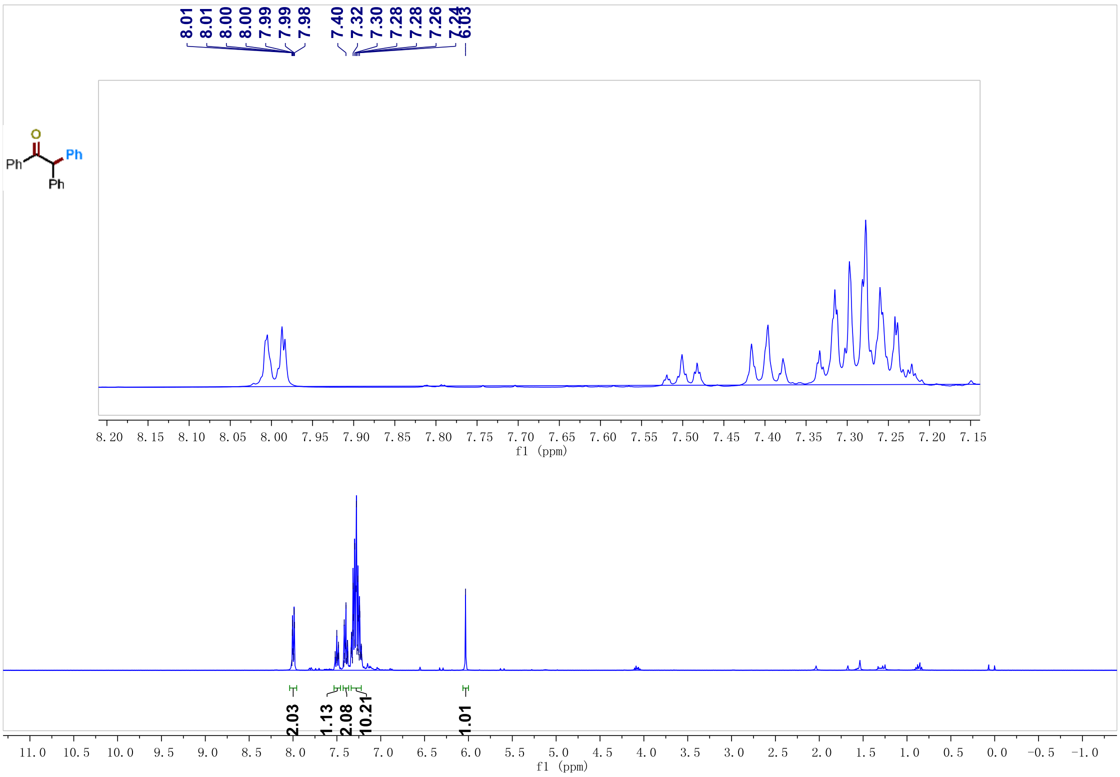

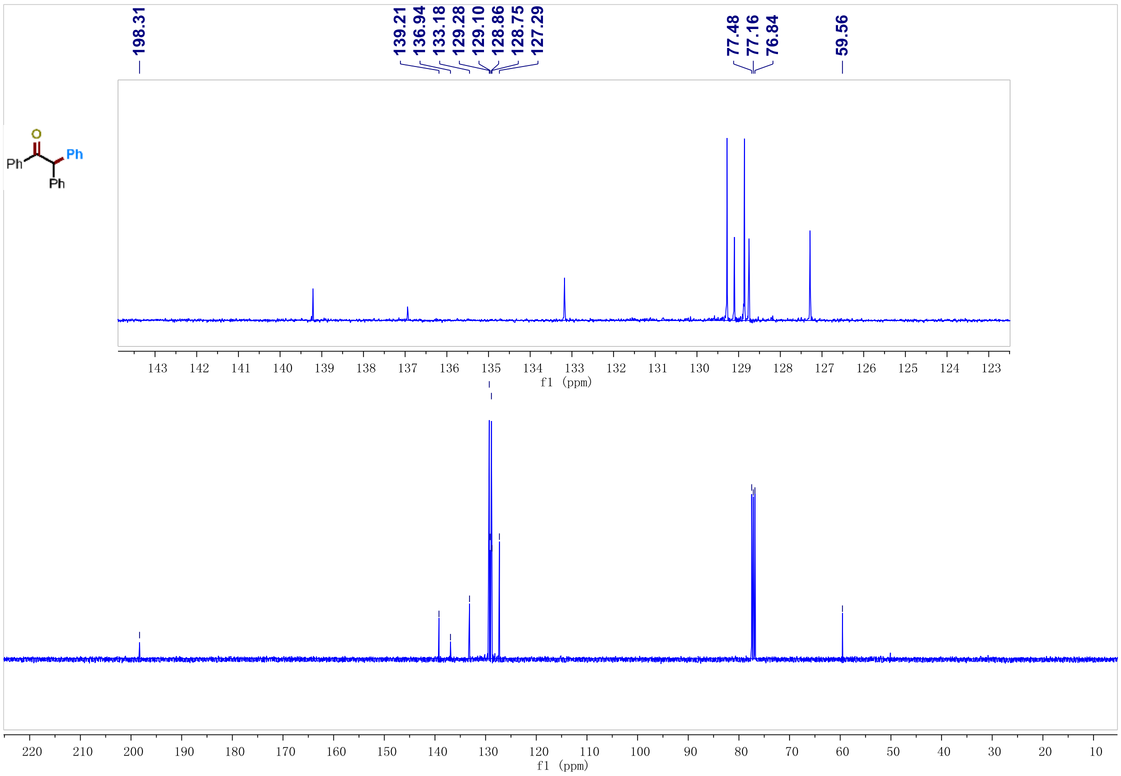

Supplementary Figure 77 ^{1}H and ^{13}C NMR Spectra for compound 16

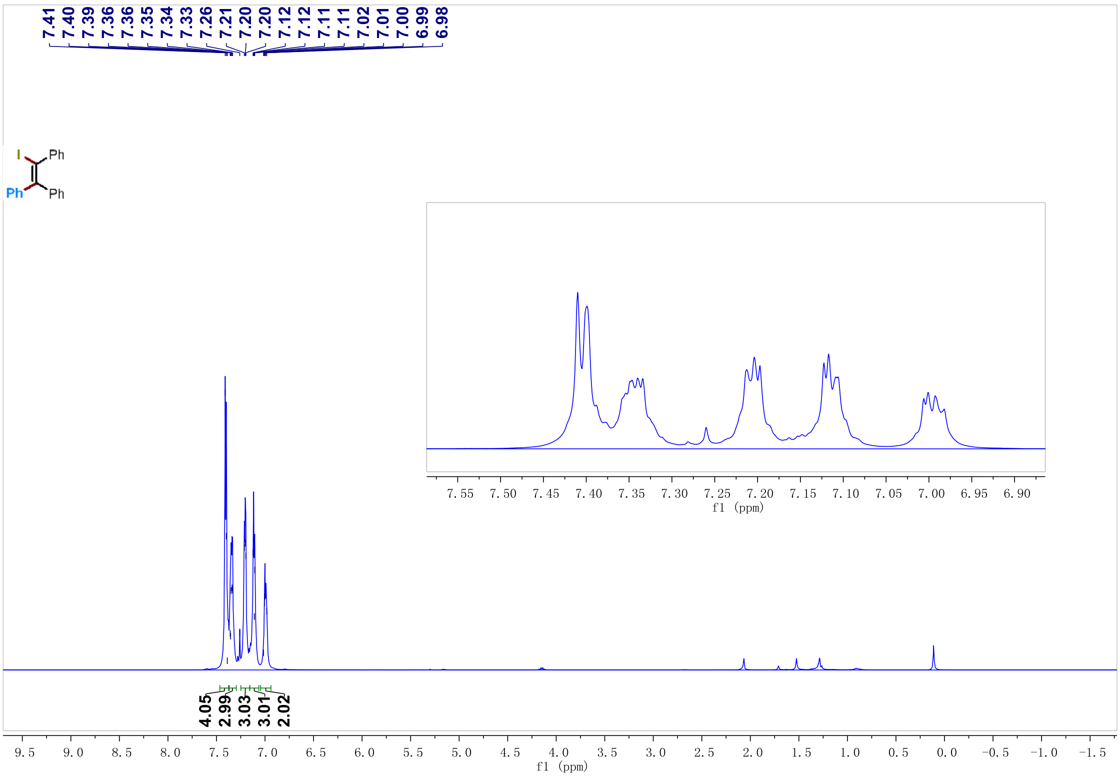

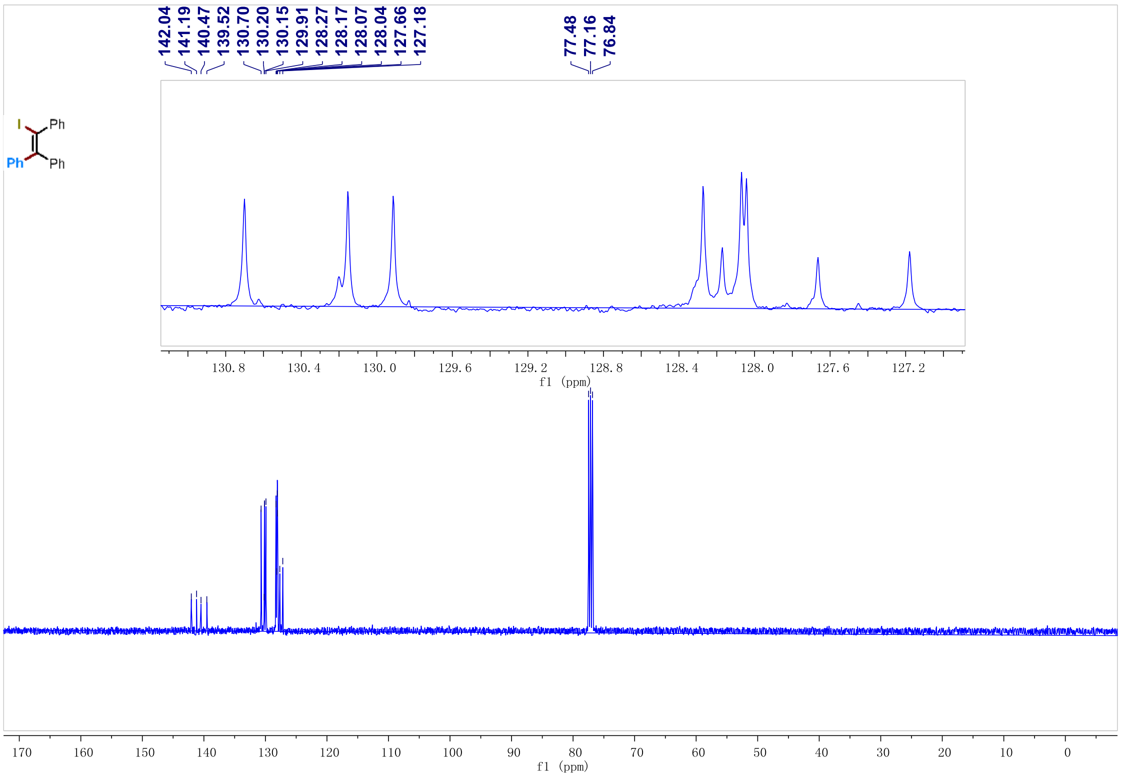

Supplementary Figure 78 ^{1}H and ^{13}C NMR Spectra for compound 17

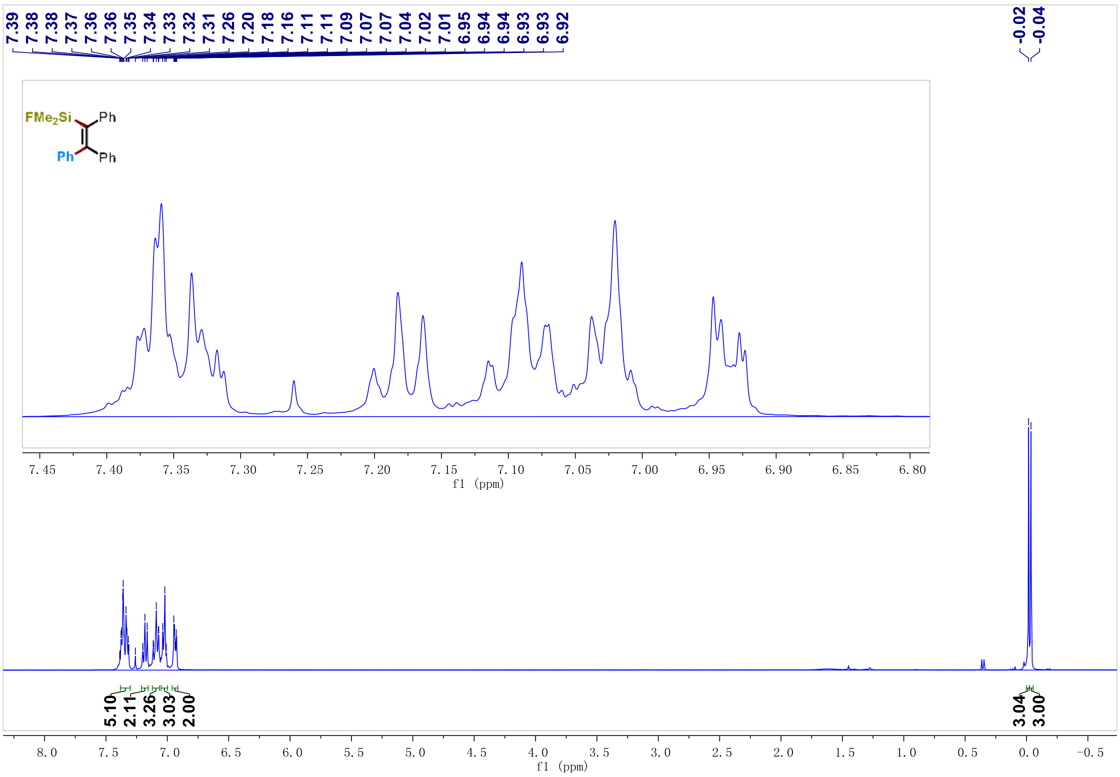
7.39
7.38
7.38
7.37
7.36
7.36
7.35
7.35
7.34
7.33
7.32
7.31
7.26
7.20
7.18
7.16
7.11
7.11
7.09
7.07
7.07
7.04
7.02
7.01
6.95
6.94
6.94
6.93
6.93
6.92
-0.02
-0.04
FMe2Si
Ph
Ph
Ph
5.10
2.11
3.26
3.03
2.00
3.04
3.00
f1 (ppm)

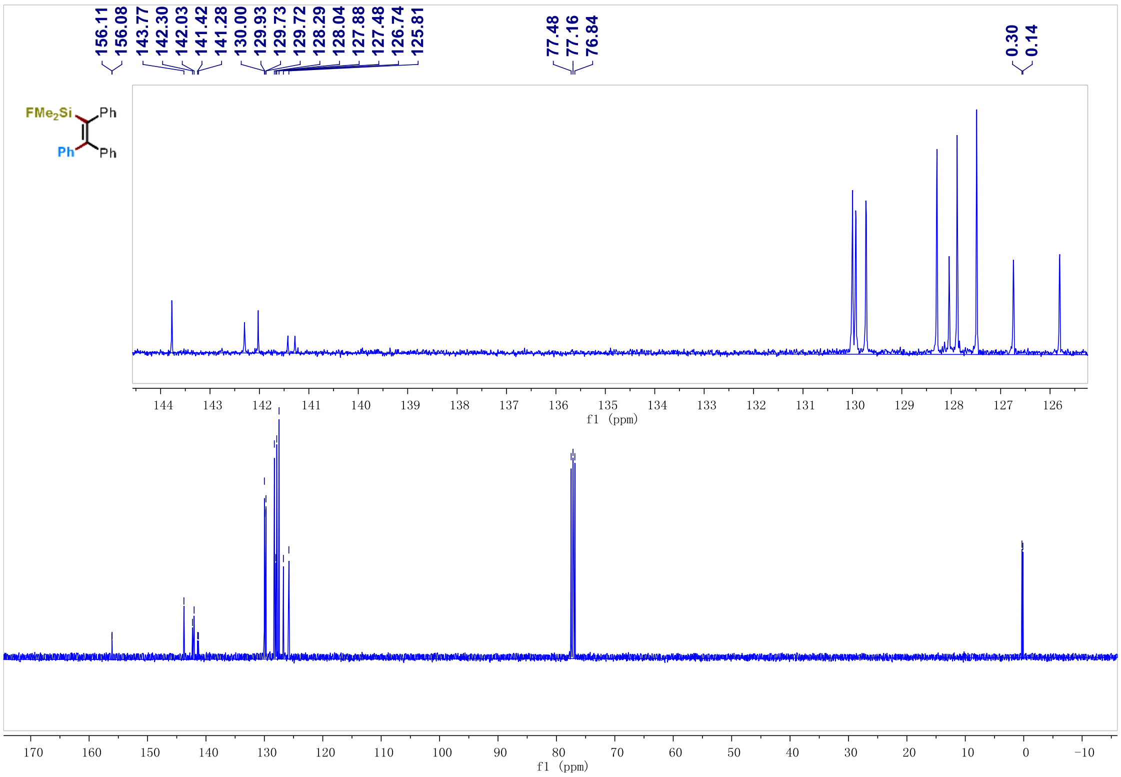
156.11
156.08
143.77
142.30
142.03
141.42
141.28
130.00
129.93
129.73
129.72
128.29
128.04
127.88
127.48
126.74
125.81
77.48
77.16
76.84
0.30
0.14
FMe2Si
Ph
Ph
Ph
f1 (ppm)

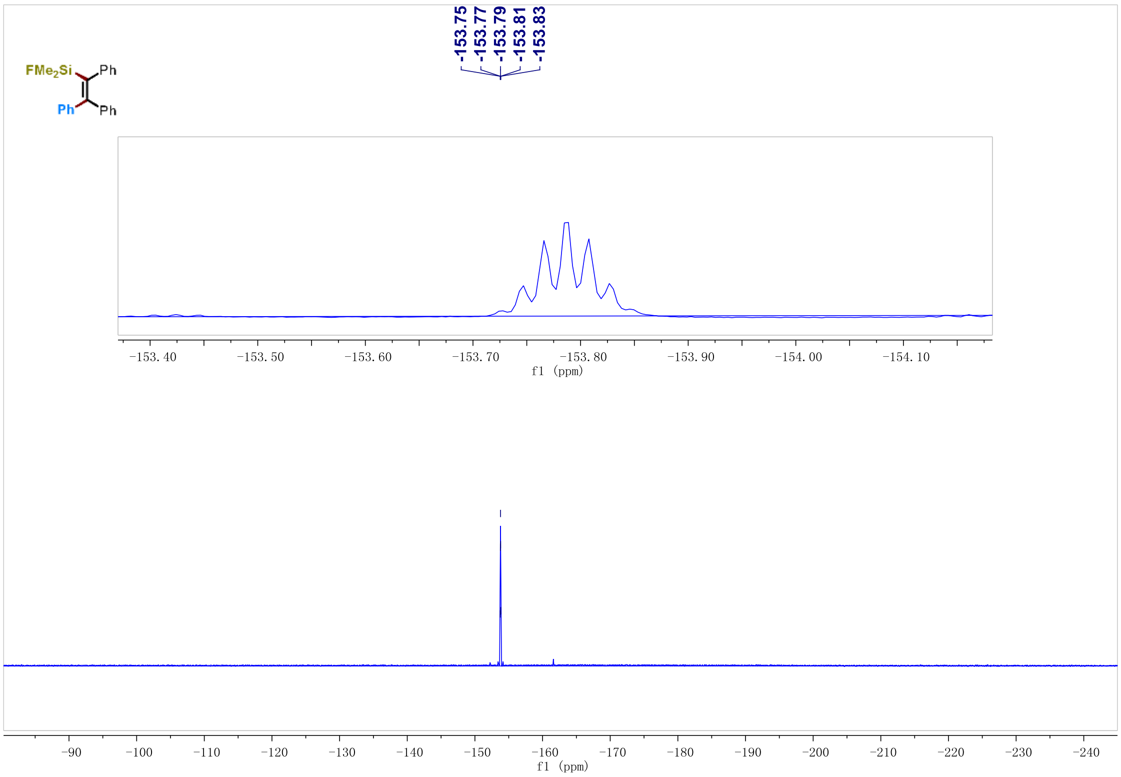

Supplementary Figure 79 ^{1}H, ^{13}C and ^{19}F NMR Spectra for compound 18

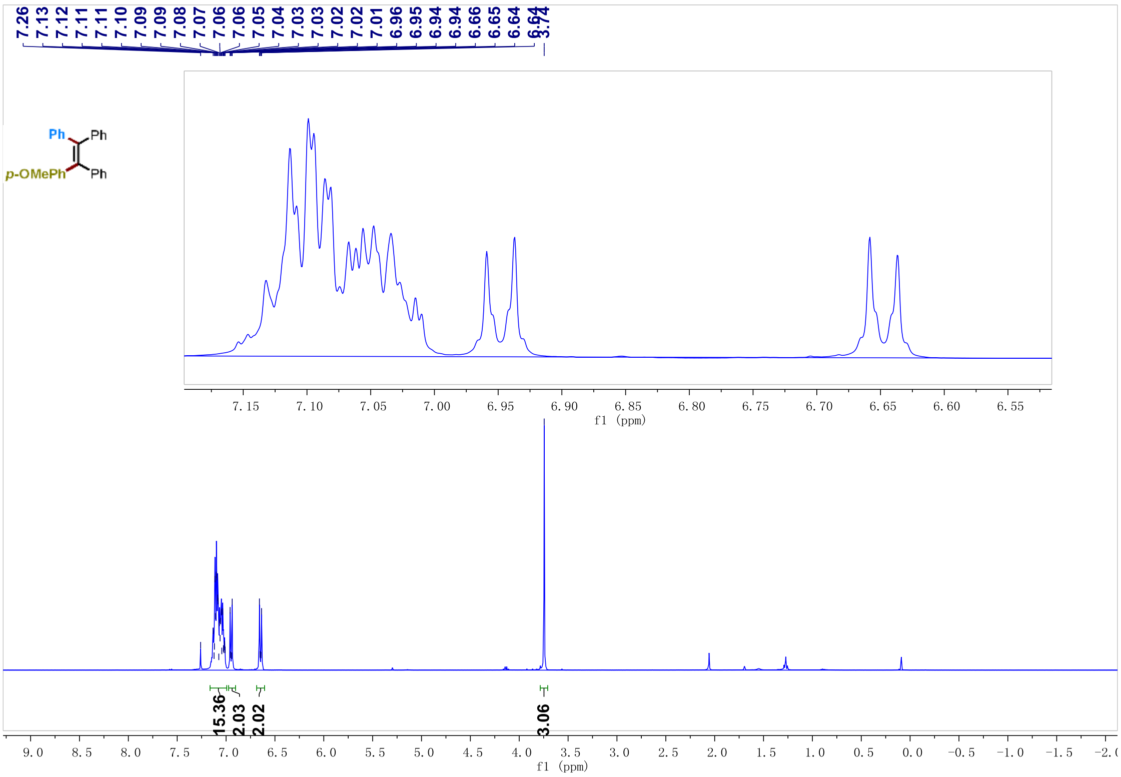

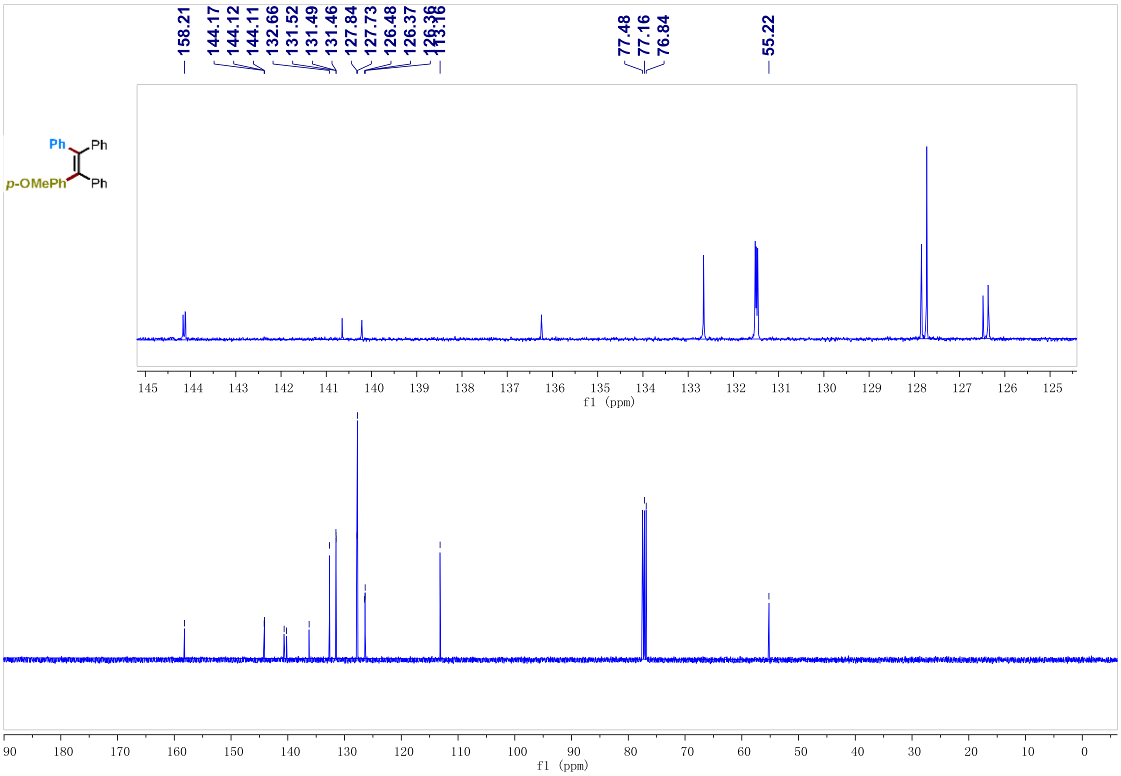

Supplementary Figure 80 ^{1}H and ^{13}C NMR Spectra for compound 19

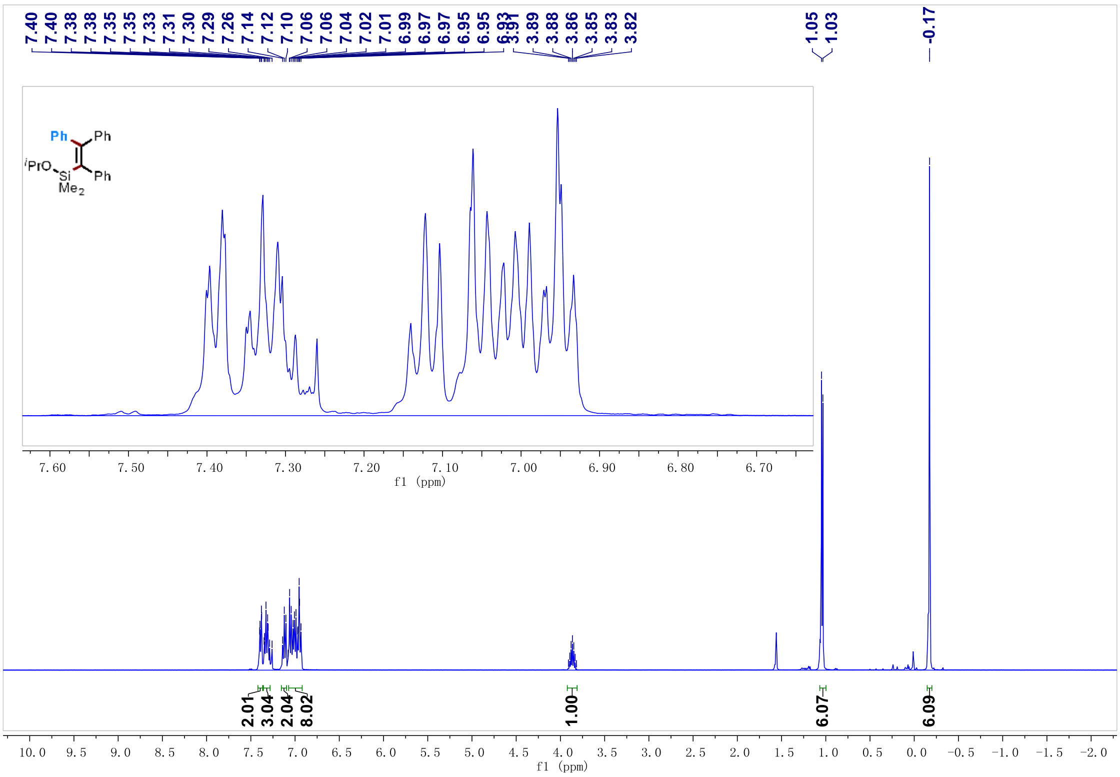

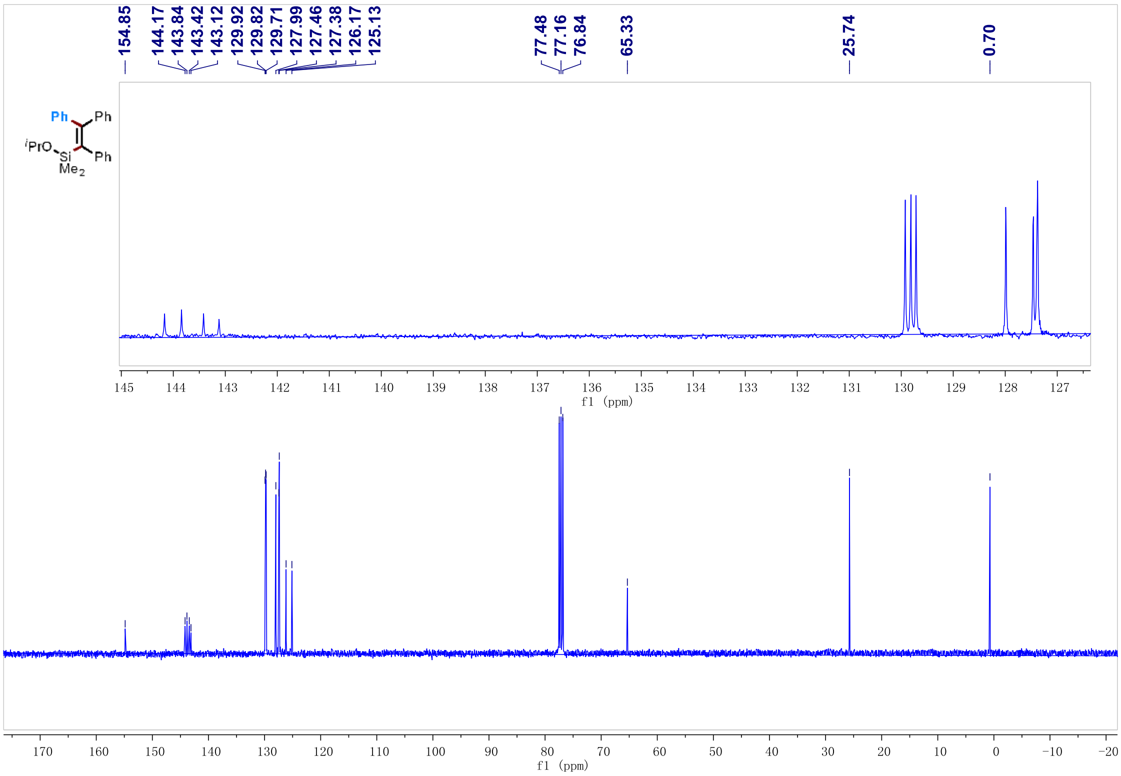

Supplementary Figure 81 ^{1}H and ^{13}C NMR Spectra for compound 20

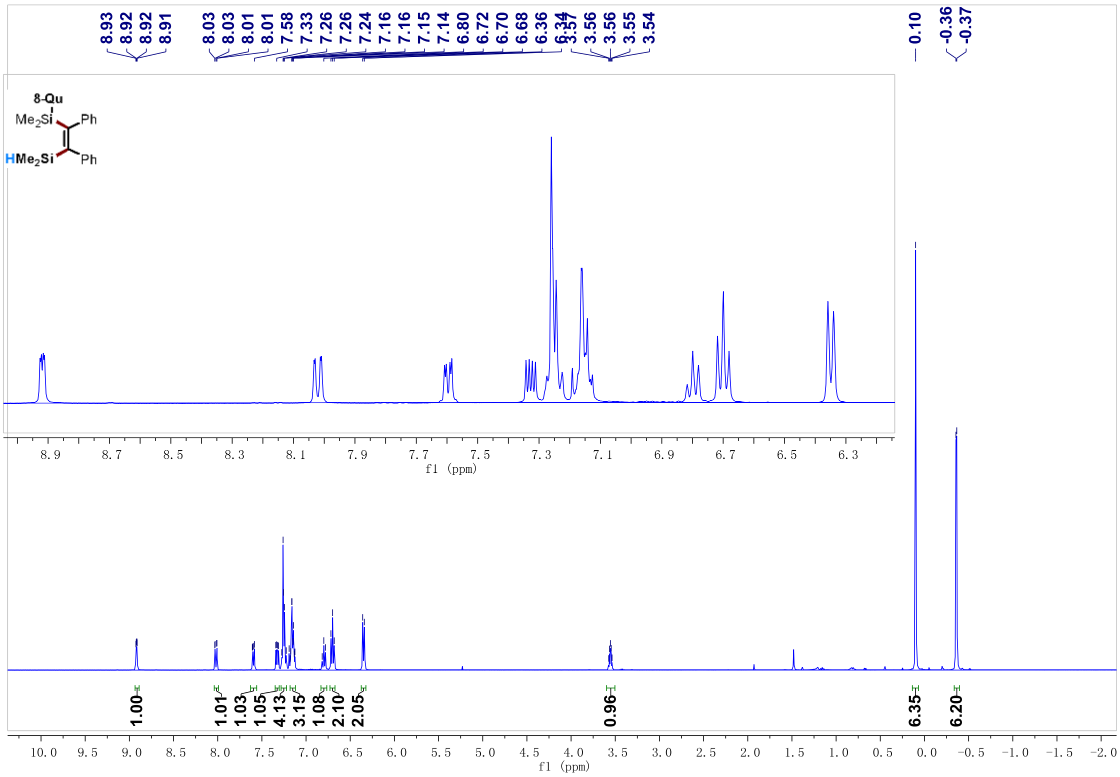

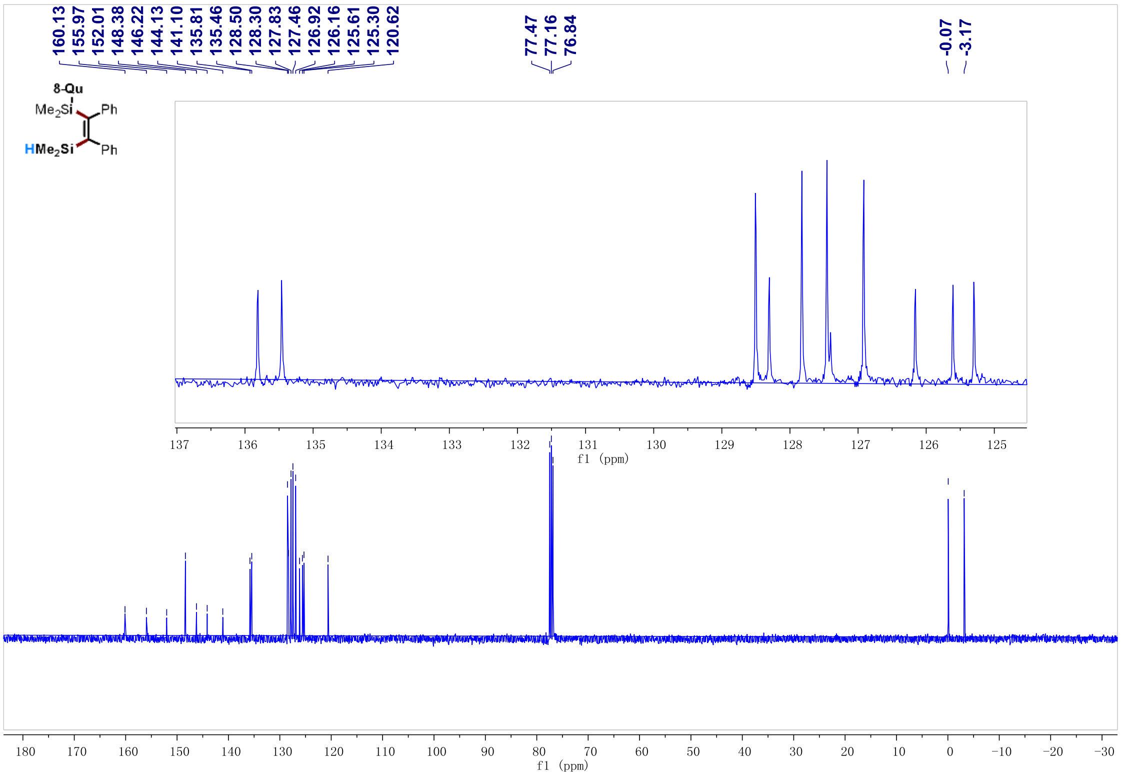

Supplementary Figure 82 ^{1}H and ^{13}C NMR Spectra for compound 21

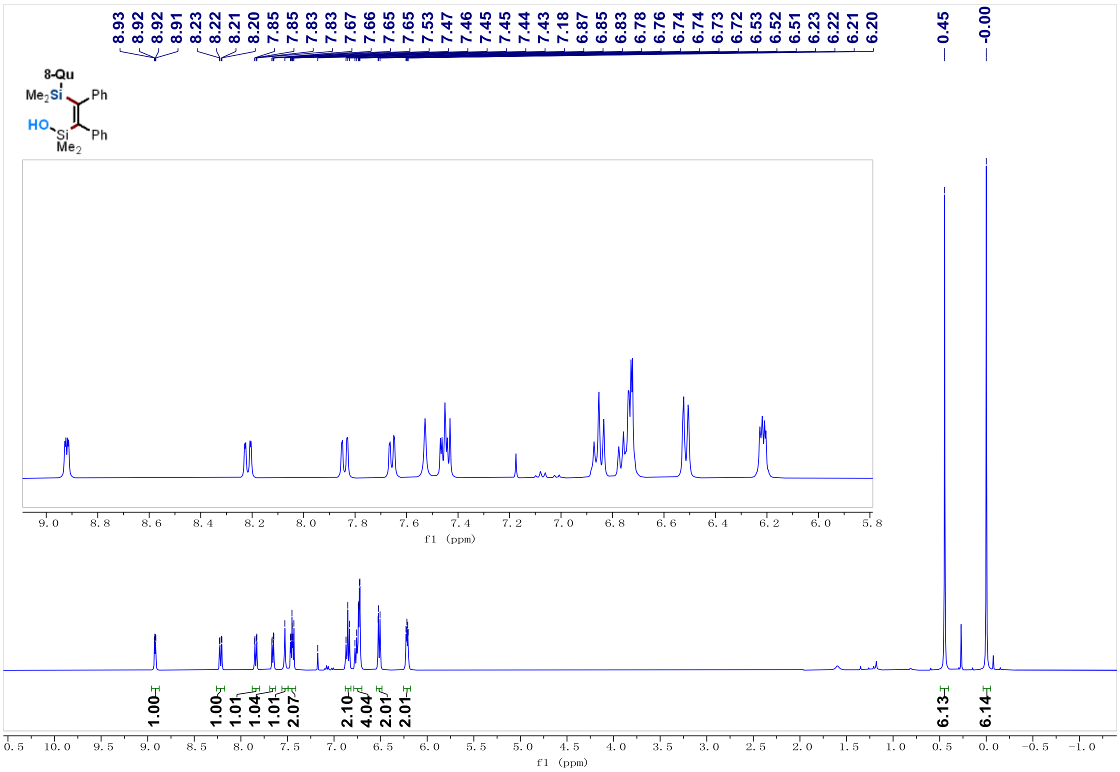

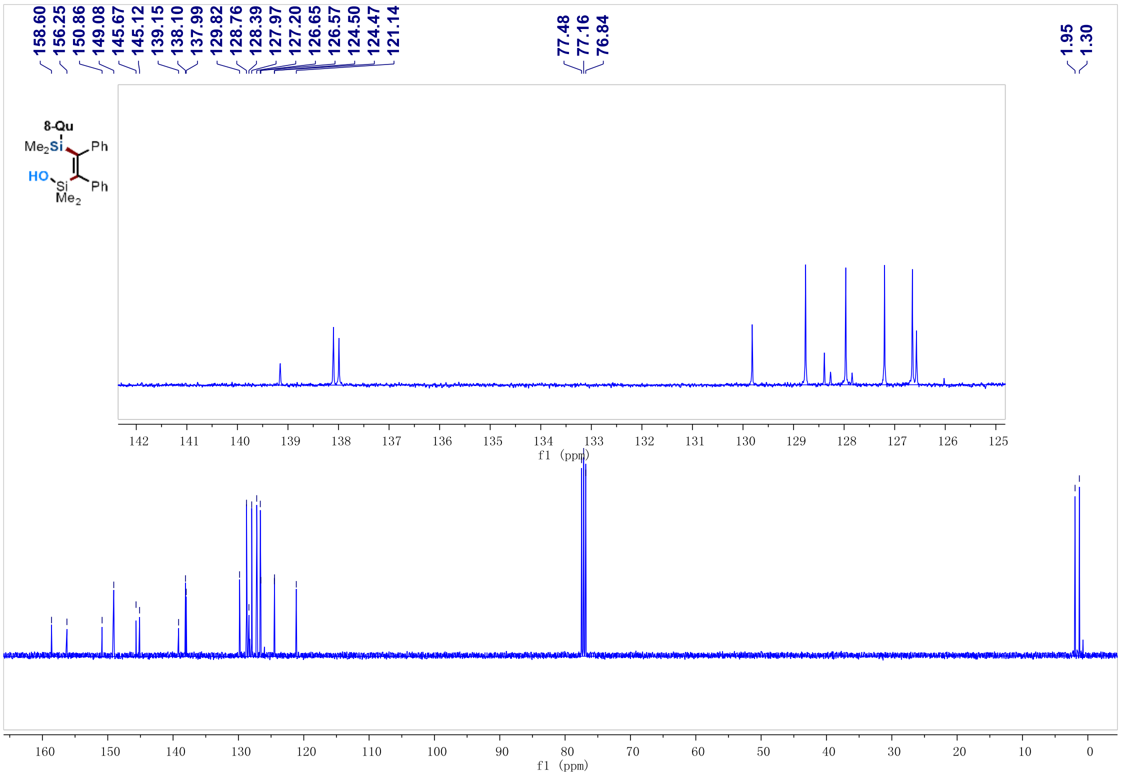

Supplementary Figure 83 ^{1}H and ^{13}C NMR Spectra for compound 22

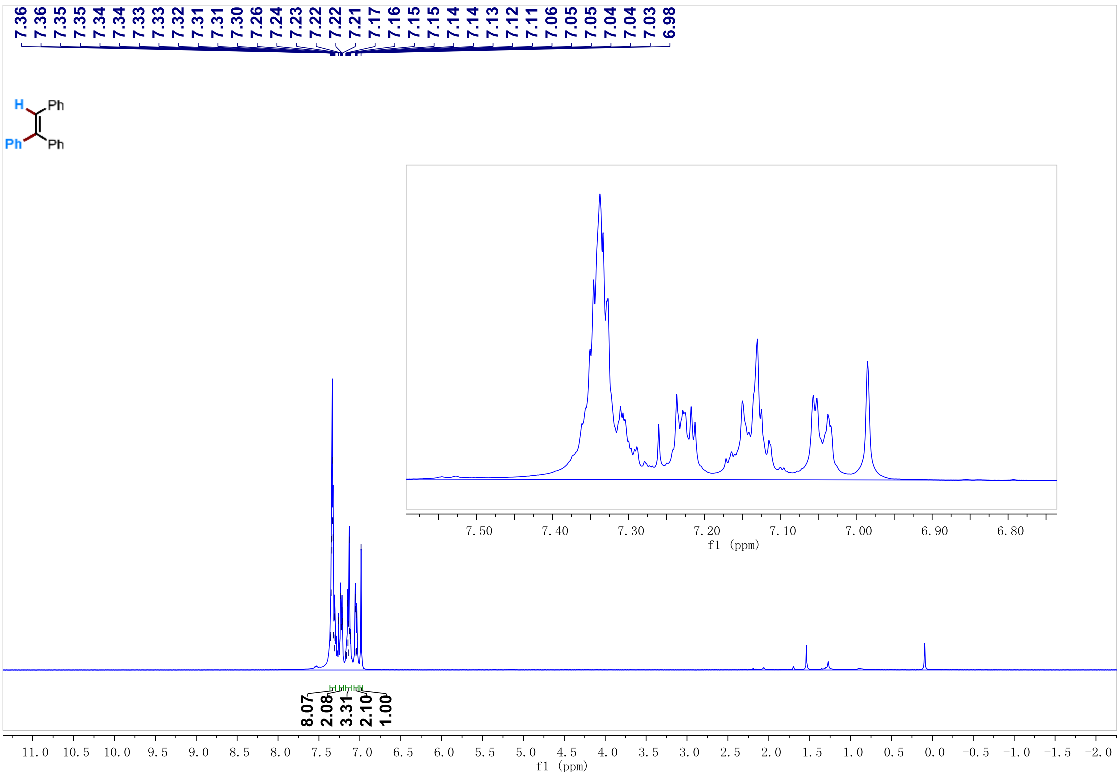

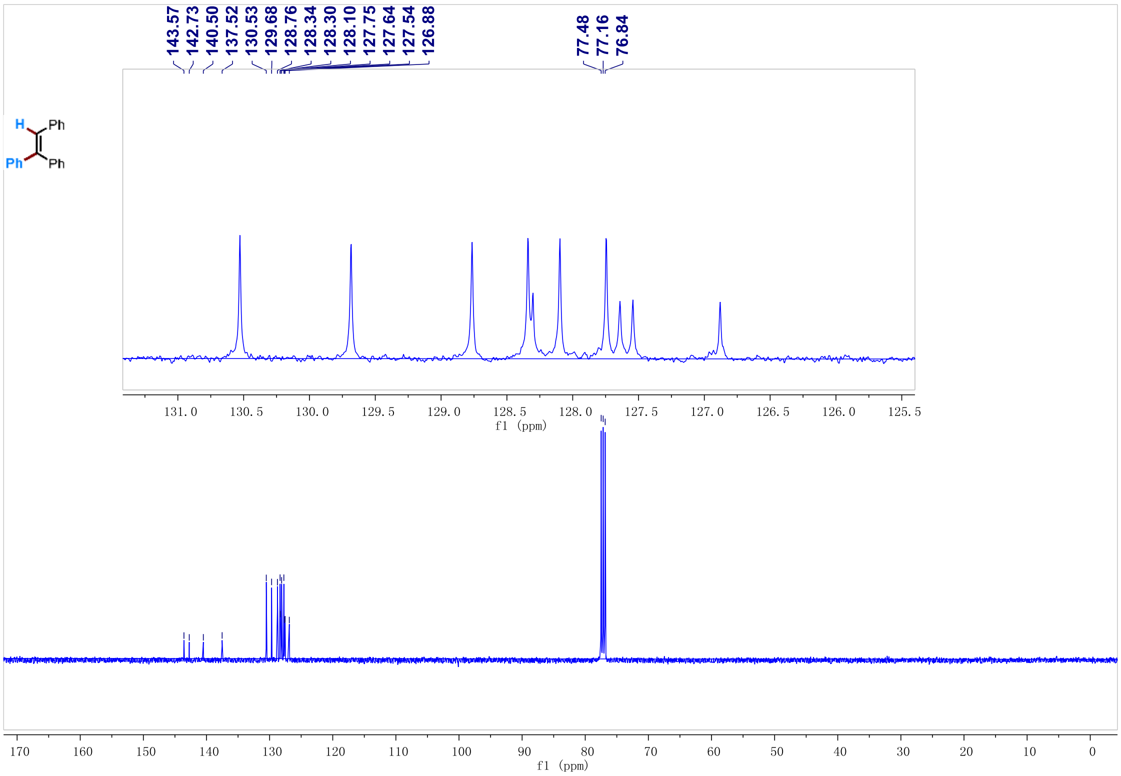

Supplementary Figure 84 ^{1}H and ^{13}C NMR Spectra for compound 23

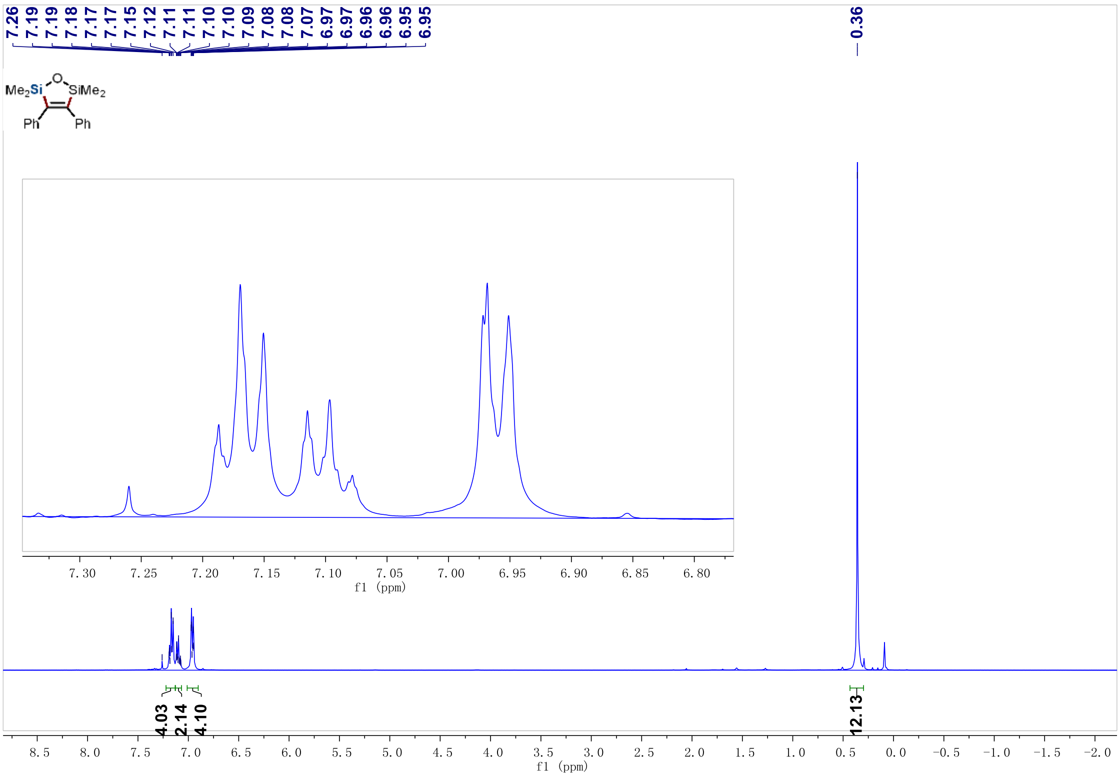

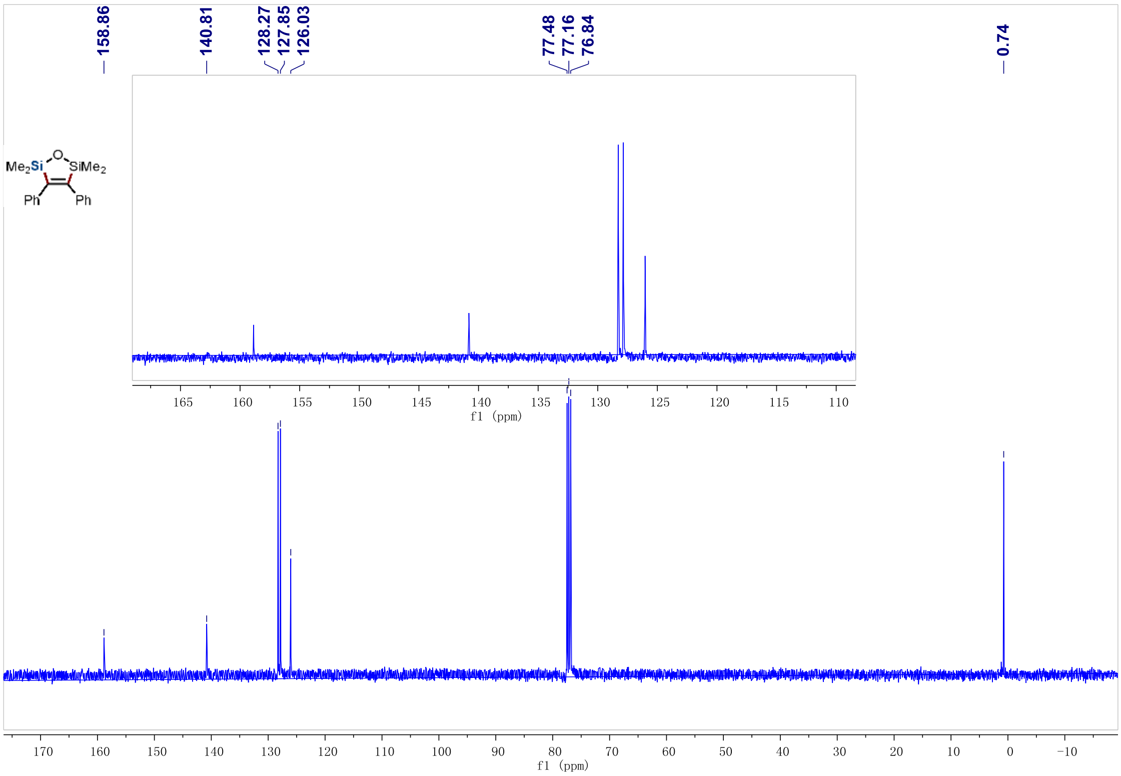

Supplementary Figure 85 ^{1}H and ^{13}C NMR Spectra for compound 24
